# Station-specific thunderstorm forecasting using climate predictors with seasonal decomposition analysis in Bangladesh’s most vulnerable regions

**DOI:** 10.1371/journal.pone.0358795

**Published:** 2026-09-24

**Authors:** Mohammad Mahboob Hussain Khan, Amrin Binte Ahmed, Adisha Dulmini, Rawfun Nahin Kowbi, Dulsaba Azmain, Muhammad Abu Sufian, Md. Mahin Uddin Qureshi, Mustafizur Rahman, Rumana Rois

**Affiliations:** 1 Bangladesh Meteorological Department (BMD), Dhaka, Bangladesh; 2 Department of Statistics and Data Science, Jahangirnagar University, Dhaka, Bangladesh; 3 Department of Business and Law, University of Wollongong, Wollongong, NSW, Australia; 4 School of Business and Economics, North South University, Dhaka, Bangladesh; 5 WTO Branch-3, Ministry of Commerce, Dhaka, Bangladesh; World Health Organization, Regional Office for South-East Asia, INDIA

## Abstract

Thunderstorms pose significant threats to life, agriculture, and infrastructure in Bangladesh, particularly in the northeastern regions of Sylhet, Sreemangal, and Mymensingh. This study develops and rigorously evaluates localized thunderstorm frequency forecasting models for these high-risk stations using 40 years (1985–2024) of monthly data and thirty six modeling approaches encompassing classical time series (SARIMA/SARIMAX, ETS/ETSX), machine learning (ANN/ANNX, SVR/SVRX, XGBoost/XGBoostX), and deep learning (LSTM/LSTMX) architectures, applied to raw and seasonally adjusted (X-11, STL) data under both univariate and exogenous frameworks incorporating five climatic variables (temperature, relative humidity, cloud cover, rainfall, atmospheric pressure). Correlation analysis identified cloud cover (r = 0.83–0.88), atmospheric pressure (r = −0.79 to −0.85), temperature (r = 0.77–0.79), and rainfall (r = 0.74–0.78) as dominant meteorological drivers of thunderstorm activity. Seasonal pre-adjustment using STL substantially improved forecast accuracy, with STL-XGBoost achieving the lowest univariate errors (Sylhet: RMSE = 2.53; Sreemangal: 2.09; Mymensingh: 2.08). Inclusion of exogenous variables dramatically enhanced predictive skill with tree-based and kernel methods: STL-XGBoostX with climate predictors proved optimal for Sylhet (MASE = 0.22), while STL-SVRX with climate predictors was optimal for Sreemangal and Mymensingh (MASE = 0.17), representing over 80% improvement compared to univariate benchmarks. Seasonal forecasts (2025–2028) project peak monsoon activity reaching 100.5 frequency at Sreemangal and identify April–August as the critical preparedness window for these stations. This study provides the Bangladesh Meteorological Department with a validated, operational framework for station-specific thunderstorm frequency forecasting, demonstrating that explicit seasonal decomposition combined with tree-based ensemble and kernel methods offers robust accuracy for early warning systems. The methodology can be extended to additional stations and integrated with socio-economic data to enhance disaster preparedness and climate adaptation strategies for Bangladesh’s most thunderstorm-vulnerable communities.

## 1. Introduction

Thunderstorms are frequent and severe weather events in Bangladesh, predominantly occurring during the pre-monsoon and monsoon seasons, which span from March to May and June to September, respectively. These storms are characterized by heavy rainfall, strong winds, hail, and lightning, often leading to substantial damage to infrastructure, agriculture, and risks to human health [1–3]. In recent decades, the increasing frequency and intensity of thunderstorms, alongside shifts in their spatial distribution, have raised significant concerns due to their deadly impacts and established connections to climate change [4]. In Bangladesh, lightning—an intrinsic component of severe thunderstorms—poses a grave threat to public health and safety, resulting in one of the highest lightning mortality rates globally, with hundreds of deaths annually [5,6]. The severity of this threat prompted the Government of Bangladesh to officially declare lightning a natural disaster in 2016 [7].

The impacts of thunderstorms extend beyond fatal loss of life to cause severe damage to infrastructure, homes, and agriculture. Crop damage, livestock loss, widespread blackouts, transportation disruptions, and building collapses are common consequences [8]. Recent studies highlight the acute vulnerability of specific groups, such ass farmers and fishermen, who have limited knowledge and practices to mitigate lightning risks despite working in high-exposure environments [2]. This vulnerability is exacerbated by a rapidly changing climate. Research confirms that Bangladesh is experiencing significant increases in temperature extremes [9], a key driver of convective instability that fuels more severe thunderstorms. Furthermore, the frequency and intensity of heatwaves, which can precondition the atmosphere for convective events, are also changing across the country [10,11].

Globally, research consistently shows that climate change is amplifying thunderstorm activity. Higher surface temperatures increase atmospheric moisture capacity and convective available potential energy (CAPE), lengthening thunderstorm seasons and boosting the number of severe weather days [12,13]. Projections for South Asia indicate a particularly heightened risk, with CAPE anticipated to increase by 44–106% in major urban areas, fostering environments more favorable for severe convective activity in densely populated regions [14,15]. In Bangladesh, warmer pre-monsoon conditions coupled with higher humidity levels have been linked to increased lightning activity [16]. The Intergovernmental Panel on Climate Change (IPCC) has underscored the increasing intensity and frequency of such extreme weather phenomena as a direct consequence of global warming [17]. Local topographical factors, such as the hilly landscape of the northeastern regions of the Indian subcontinent and Bangladesh—specifically the north-east Himalayan region (21–29°N; 86–94°E) encompassing Sylhet, Sreemangal, and Mymensingh—further catalyze localized convection, while urban heat islands may intensify storms around cities [18]. For instance, the northeastern regions of Bangladesh, such as Sylhet, Sreemangal, and Mymensingh, experience a particularly high frequency of thunderstorms, making them vulnerable to climate-induced weather extremes [19,20].

Accurate forecasting is paramount for mitigating these impacts. Globally, substantial progress has been made using advanced statistical and machine learning (ML) techniques. Methods such as Support Vector Machines (SVM), gradient-boosted decision trees, and Artificial Neural Network (ANN) have demonstrated high predictive accuracy for severe weather [21–23]. In Bangladesh, research has investigated thunderstorms climatology [24–26], lightning forecasting [27], and socio-economic impacts [8]. Numerical models like the Advanced Research Weather Research and Forecasting (ARW-WRF) model have been used in previous studies to simulate storms in Bangladesh [28,29]. Additionally, hybrid approaches like the ensemble empirical mode decomposition (EEMD)-SVM model [30] and statistical methods like SARIMA [31] have been applied to forecast thunderstorms frequency, often using aggregated national data. However, a critical gap remains in developing accurate localized forecasting models that account for the unique climatology of Bangladesh’s most vulnerable regions. While previous studies have identified climatic drivers, they have not systematically quantified the value of integrating these drivers into operational forecasting models at the station level.

This study addresses this methodological gap by providing a rigorous, comparative evaluation of 36 modeling configurations (twelve algorithms encompassing two frameworks, and three preprocessing approaches) for station-specific thunderstorm frequency forecasting. We analyze monthly thunderstorm data over a 40-year period (1985–2024) from the three most thunderstorm-prone regions in northeastern Bangladesh: Sylhet, Sreemangal, and Mymensingh. We move beyond aggregated national models to provide station-specific forecasts using a comprehensive suite of eighteen modeling approaches, including classical time-series, ML and deep learning techniques, applied to raw and seasonally adjusted data using X-11 and Seasonal-Trend decomposition using Loess (STL) methods, to capture the unique temporal and spatial dynamics of thunderstorms to enhance early warning capacity and public safety. A key innovation of our approach is the integration of five exogenous climatic variables—temperature, relative humidity, cloud cover, rainfall, and atmospheric pressure—as predictors alongside historical thunderstorm data, enabling us to capture the meteorological drivers of thunderstorm activity. We further evaluate two modeling frameworks: univariate models (using only thunderstorm history) and exogenous models (incorporating climate predictors), allowing systematic quantification of the value added by meteorological information. Additionally, we rigorously evaluate seasonal adjustment techniques—specifically X-11 and STL—to isolate and model the strong seasonal patterns inherent in thunderstorm data, a step often neglected in conventional forecasting methodologies. Consequently, our research objectives are threefold: (i) to develop and compare accurate localized univariate and exogenous thunderstorm forecasting models for high-risk regions in Bangladesh; (ii) to quantify the improvement in forecast accuracy achieved through explicit seasonal adjustment using X-11 and STL decomposition; and (iii) to assess the contribution of exogenous climatic variables in enhancing predictive skill. By integrating climatology, time-series analysis, and ML methods, this research provides a robust framework for improving thunderstorm early warning systems. The findings are designed to support the operational forecasting of the Bangladesh Meteorological Department (BMD) and to inform evidence-based disaster preparedness and climate adaptation strategies tailored to protect vulnerable communities in Bangladesh’s most thunderstorm-prone regions.

## 2. Materials and methods

### 2.1. Study area and data

Following the BMD Operational Manual [32], a thunderstorm occurrence is officially defined as an observation period during which thunder is audibly heard by a certified meteorological observer at the station, regardless of whether accompanying precipitation occurs. Thunderstorm frequency is defined as the number of thunderstorm occurrences recorded at a given location over a specified temporal scale (e.g., daily, monthly, seasonal, or annual). In the BMD network, thunderstorm observations are made eight times per day at 3‑hour intervals following World Meteorological Organization (WMO) standard synoptic hours. All data underwent rigorous quality control by BMD, including consistency checks and temporal continuity verification, with no major station relocations and consistent adherence to WMO standards throughout 1985–2024. The count of thunderstorm reports within a calendar day is treated as the daily thunderstorm frequency, and monthly thunderstorm frequency for each station is obtained by aggregating the corresponding daily frequencies over the month.

#### 2.1.1. Description of study stations and data.

**Fig 1:**
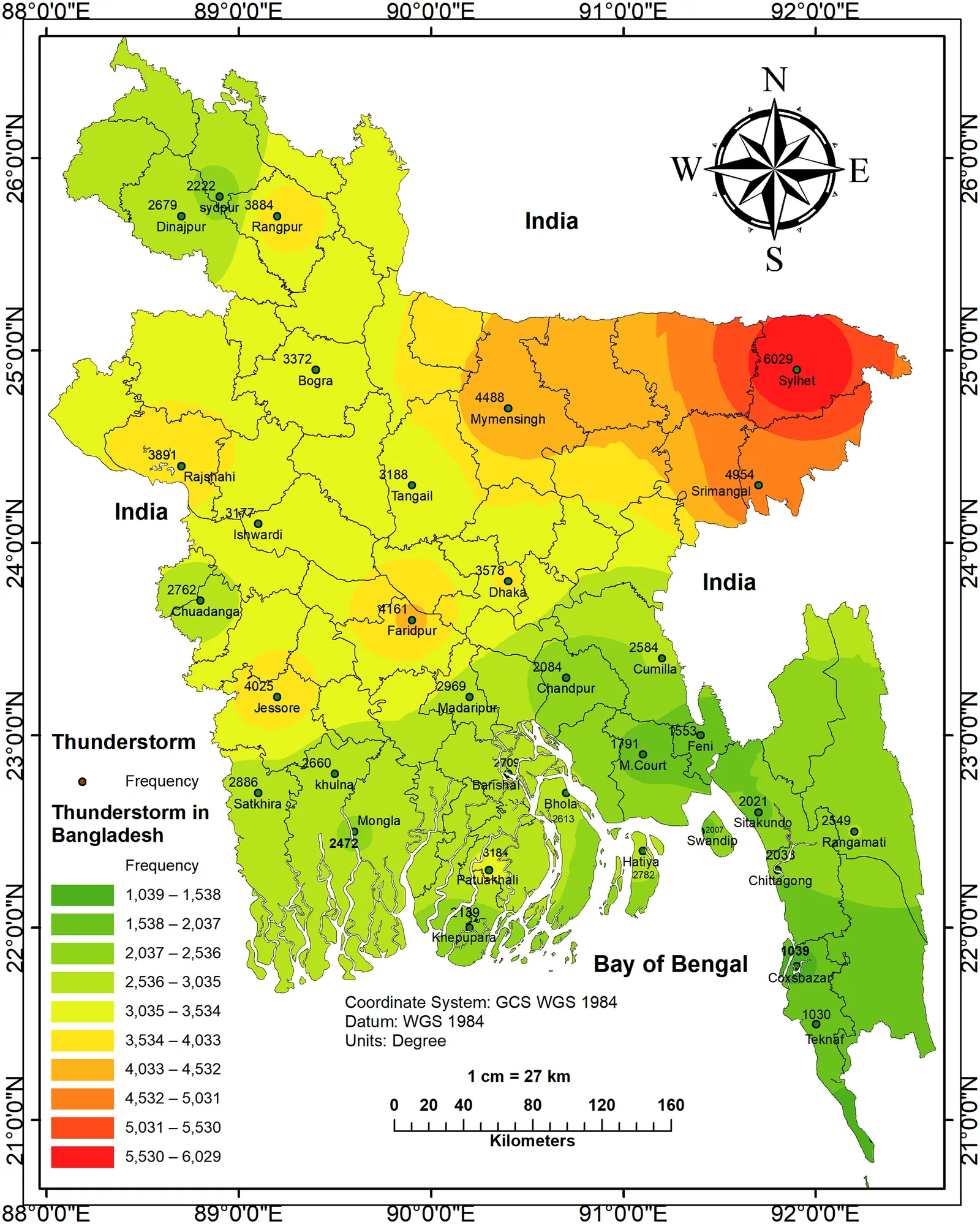
Spatial distribution of thunderstorm frequency across Bangladesh (1985–2024), highlighting the most disaster-prone areas. Created with QGIS Geographic Information System version 3.30’s-Hertogenbosch (https://qgis.org/project/visual-changelogs/visualchangelog330/).

The dataset comprises monthly thunderstorm frequency, monthly average maximum temperature (°C), monthly average relative humidity (%), monthly average cloud cover (oktas), monthly average rainfall (mm), and monthly average atmospheric pressure (hPa) for three BMD meteorological stations: Sylhet (ID: 41891), Sreemangal (ID: 41915), and Mymensingh (ID: 41886). The temporal coverage extends from January 1985 to December 2024, providing 40 complete years (480 months) of observations for each station. All data were obtained directly from the Climate Division of BMD, located in Dhaka. The selected study areas: Sylhet, Sreemangal, and Mymensingh are among the most high-risk meteorological zones in Bangladesh in terms of thunderstorm activity [19,20,33]. These stations were purposefully chosen due to their historically elevated frequency of thunderstorm occurrences, marking them as critical regions for climate-related hazard assessment (Fig 1). These locations are situated in the northeastern region of Bangladesh, which is particularly vulnerable to climatic shifts and extreme weather events.

**Fig 2:**
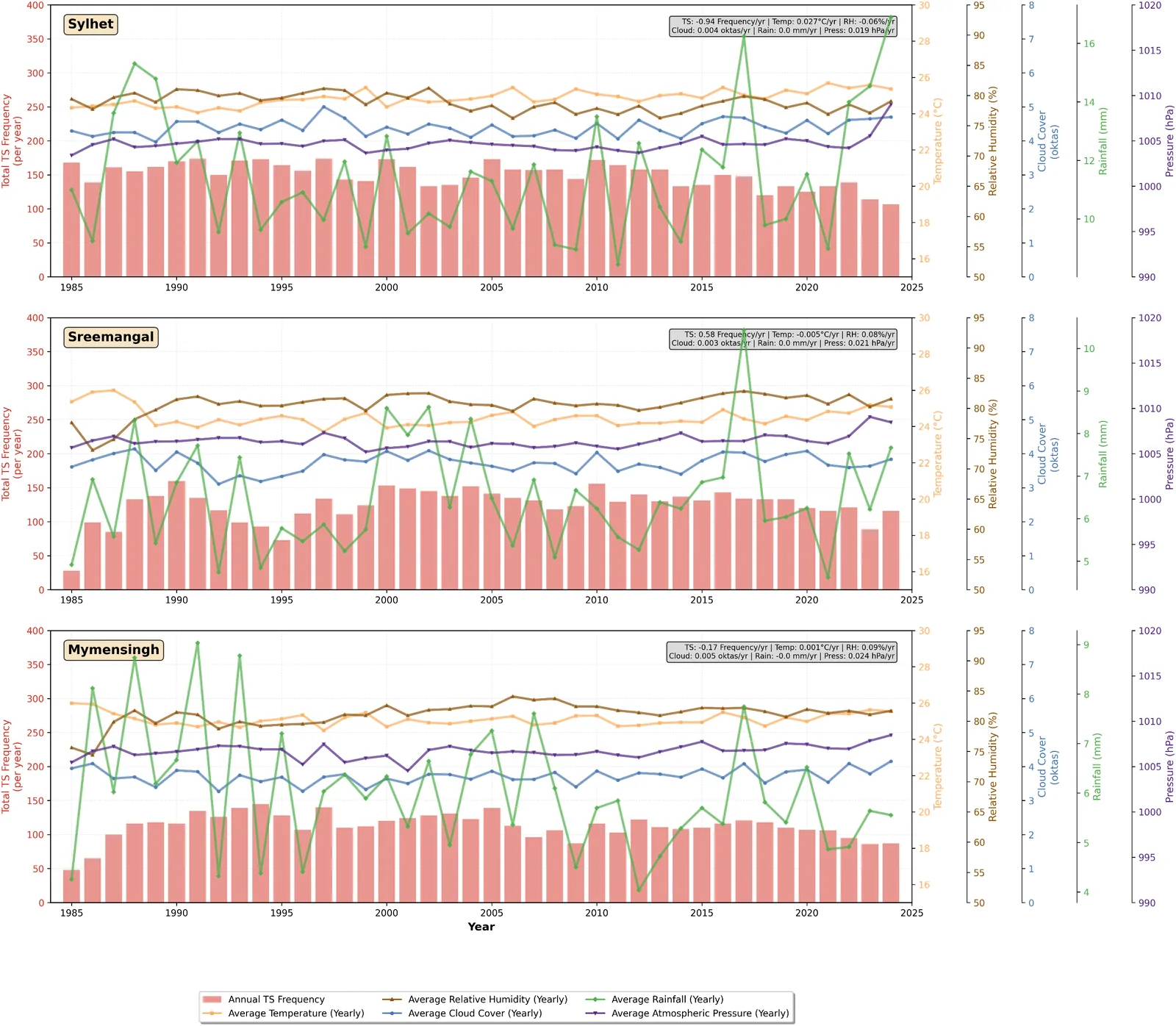
Yearly Climate Trends and Thunderstorm Dynamics in Bangladesh’s Most Vulnerable Regions (1985–2024) for Sylhet (top), Sreemangal (middle), and Mymensingh (bottom).

Table 1 provides the geographical coordinates and a 40-year (1985–2024) climatological summary for three thunderstorm-prone stations in northeastern Bangladesh: Mymensingh, Sylhet, and Sreemangal. Located within a narrow latitudinal range (24.30°N to 24.90°N) and longitudinal band (90.43°E to 91.88°E), the stations exhibit distinct climatological characteristics despite their geographic proximity. Sylhet recorded the highest total thunderstorm frequency (6,029 events) over the study period, followed by Sreemangal (4,954) and Mymensingh (4,488), corresponding to monthly average frequencies of 12.56 ± 9.67, 10.32 ± 8.67, and 9.35 ± 7.94 days, respectively (Table 1). This spatial gradient in thunderstorm activity is consistent with Sylhet’s unique orographic setting, which enhances convective processes. The mean monthly maximum temperature is comparable across all stations, ranging from 24.51 ± 4.15°C in Sreemangal to 25.11 ± 3.94°C in Mymensingh. Relative humidity averages between 78.87 ± 7.27% (Sylhet) and 81.09 ± 5.39% (Mymensingh), indicating persistently moist conditions favorable for thunderstorm development. Cloud cover is highest over Sylhet (4.38 ± 2.21 oktas), while Mymensingh and Sreemangal exhibit similar, lower values (approximately 3.7 oktas) (Table 1). A striking contrast is observed in rainfall, with Sylhet receiving nearly double the monthly average (11.45 ± 11.59 mm) compared to Mymensingh (6.01 ± 6.33 mm) and Sreemangal (6.48 ± 6.28 mm), further underscoring its exposure to intense convective systems. Atmospheric pressure is slightly lower over Sylhet (1004.7 ± 4.74 hPa), consistent with its higher convective activity, compared to Mymensingh (1006.67 ± 5.34 hPa) and Sreemangal (1006.4 ± 4.85 hPa). This numerical summary complements the textual description of the orographic setting (proximity to the Meghalaya and Tripura hills), moisture inflow from the Bay of Bengal, and the known convective environment in these regions (Table 1). Fig 2 shows substantial multi‑decadal variability in annual thunderstorm counts at Sreemangal and Mymensingh, with increases and decreases before ~2010 and an apparent decline thereafter. Because these series are not clearly monotonic, a single linear fit across 1985–2024 can mask more recent trends; therefore, slopes estimated over the full period should be interpreted with caution. Formal trend analysis (Table 1) using the Mann–Kendall test and Sen’s slope on the full 1985–2024 record indicates a statistically significant decreasing trend at Sylhet (Mann-Kendall’s τ = −0.076, p -value = 0.015; Sen’s slope = −0.0037 days per month, 95% CI: −0.0094 to 0.0000), while Sreemangal and Mymensingh shows no significant trends over the full period (Sreemangal: Mann-Kendall’s τ = 0.046, p -value = 0.145; Sen’s slope = 0.0000, 95% CI: 0.0000 to 0.0054. Mymensingh: Mann-Kendall’s τ = −0.0215, p -value = 0.497; Sen’s slope = 0.0000, 95% CI: −0.0027 to 0.0000). Thus, although formal tests on the full record do not detect significant trends at Sreemangal or Mymensingh, the visual evidence of multi‑decadal variability and the post‑2010 decline are noted and may warrant more focused, recent‑period or piecewise trend analyzes in follow‑up work.

**Table 1:** Geographical location, climatological characteristics, and trend analysis of monthly thunderstorm frequency for three thunderstorm-prone stations in Bangladesh (1985–2024).

Station Name	Sylhet (Mean ± SD)	Sreemangal (Mean ± SD)	Mymensingh (Mean ± SD)
Station ID	41891	41915	41886
Latitude	24.90°N	24.30°N	24.72°N
Longitude	91.88°E	91.73°E	90.43°E
Total Thunderstorm Frequency	6,029	4,954	4,488
Monthly Average Thunderstorm Frequency	(12.56 ± 9.67)	(10.32 ± 8.67)	(9.35 ± 7.94)
Monthly Average Maximum Temperature (°C)	(24.86 ± 3.43)	(24.51 ± 4.15)	(25.11 ± 3.94)
Monthly Average Relative Humidity (%)	(78.87 ± 7.27)	(80.64 ± 5.73)	(81.09 ± 5.39)
Monthly Average Cloud Cover (oktas)	(4.38 ± 2.21)	(3.72 ± 2.01)	(3.71 ± 2.15)
Monthly Average Rainfall (mm)	(11.45 ± 11.59)	(6.48 ± 6.28)	(6.01 ± 6.33)
Monthly Average Atmospheric Pressure (hPa)	(1004.7 ± 4.74)	(1006.4 ± 4.85)	(1006.67 ± 5.34)
Mann-Kendall’s τ	−0.076	0.0459	−0.0215
z-statistic	−2.4239	1.4576	−0.6799
p -value	0.0154	0.145	0.4965
Sen’s Slope (frequency/month)	−0.0037	0.0000	0.0000
95% Confidence Interval	(−0.0094, 0.0000)	(0.0000, 0.0054)	(−0.0027, 0.0000)
Trend Interpretation	Statistically significant decreasing trend	No significant trend	No significant trend

The presence of orographic uplift caused by the nearby Meghalaya Plateau and surrounding hills generates strong vertical motions that lift moisture-laden air masses, intensifying convective instability [19,20]. Furthermore, the continuous influx of moisture from the Bay of Bengal increases low-level humidity, supplying ample fuel for thunderstorm development [33]. Regional circulation patterns contribute to these dynamics by converging moist easterlies with westerly disturbances [33]. During the pre-monsoon season (March–May), intense surface heating combined with vertical wind shear facilitates the formation of Nor’wester-type convective storms [34]. These areas also receive some of the highest annual rainfall totals in Bangladesh, often exceeding 4000 mm in Sylhet, fostering conditions favorable for severe storm development [33]. Socio-environmentally, dense rural populations and limited infrastructure resilience heighten vulnerability to hazards such as lightning strikes, crop damage, and power outages, underscoring the importance of these regions for targeted thunderstorm climate research.

Fig 2 presents a comprehensive 40-year (1985–2024) analysis of yearly climate trends and thunderstorm frequency dynamics across three meteorologically vulnerable stations in northeastern Bangladesh—Sylhet (top panel), Sreemangal (middle panel), and Mymensingh (bottom panel). The visualization employs a multi-axis approach where annual thunderstorm frequency is depicted as semi-transparent red bars (left y-axis, ranging 0–400 days), while five complementary climate variables are overlaid as color-coded lines on offset right y-axes: average annual temperature (light orange, 15–30°C), relative humidity (brown, 50–95%), cloud cover (blue, 0–8 oktas), rainfall (green, 4–17 mm), and atmospheric pressure (purple, 990–1020 hPa). All stations exhibit substantial multi-decadal variability in thunderstorm frequency, consistent with the formal trend results reported in Table 1 rather than a uniform directional trend, alongside consistent warming (0.02–0.03°C/year) and pronounced interannual variability in rainfall and cloud cover. Notably, Sylhet records the highest thunderstorm activity with peaks exceeding 150 days, while Sreemangal and Mymensingh show comparatively lower frequencies with notable interannual variability (Fig 2). The synchronized multi-variable display reveals complex interactions where periods of heightened thunderstorm activity often coincide with specific combinations of increased cloud cover, elevated humidity, and pressure fluctuations. Sylhet’s dominant thunderstorm frequency is attributed to its unique physiographical setting [33]. Positioned in a basin bordered by the Meghalaya and Tripura hills, Sylhet fosters enhanced trapping and uplift of moist air masses, which intensifies convective cloud formation. Proximity to the extremely wet highlands of Cherrapunji and Mawsynram ensures a continuous moisture supply, while orographic forcing extends the active convective season and amplifies thunderstorm intensity and variability [33,34]. These combined meteorological mechanisms explain Sylhet’s persistently higher thunderstorm day counts compared to Sreemangal and Mymensingh [26,34].

These findings provide a robust dataset for analyzing temporal patterns and structural changes in thunderstorm frequency over time, which informs the subsequent development of forecasting models. However, a key limitation of this study is the absence of long, station‑level socio‑economic time series (1985–2024) data. As a result, we restrict our quantitative analysis to ground‑based weather observations (thunderstorm frequency, temperature, relative humidity, cloud cover, rainfall, and atmospheric pressure) and discuss socio‑economic vulnerability qualitatively using recent literature rather than as a time‑varying covariate.

#### 2.1.2. Justification for ground-based data.

Although satellite-based datasets such as the Lightning Imaging Sensor onboard the International Space Station (ISS-LIS) offer valuable insights into global lightning and convective activity, they were not utilized in this study due to several limitations. First, the ISS-LIS dataset provides relatively short-term coverage beginning in 2017 [35], whereas the present analysis focuses on long-term patterns over a 40-year period. Second, ground-based observational data from BMD ensures consistent, high-resolution, and locally validated monitoring, which is essential for accurate station-level modeling. In contrast, satellite-based products often suffer from spatial aggregation issues and detection limitations, particularly under complex atmospheric conditions common in South Asia [36,37]. Lastly, the primary objective of this research was to evaluate the temporal dynamics and forecasting of thunderstorm occurrences at meteorological stations using long-term ground-based data. Future efforts should incorporate satellite-derived lightning indices and socio-economic datasets to improve forecasting capabilities.

### 2.2. Modeling framework

We used 18 modeling approaches to forecast monthly thunderstorm frequency in Sylhet, Sreemangal, and Mymensingh, combining traditional time series, machine learning, and deep learning architectures with seasonal decomposition techniques, specifically X-11 [38,39] and STL [40] for seasonal adjustment. Models were developed under two frameworks: univariate (using only historical thunderstorm data) and exogenous (incorporating climatic predictors), enabling systematic evaluation of the value added by meteorological drivers.

#### 2.2.1. Univariate models.

The univariate framework uses only historical thunderstorm frequency as input, serving as a baseline for assessing the contribution of climatic predictors. Six core univariate models were implemented, each applied to raw, X-11 adjusted, and STL adjusted data: ARIMA/SARIMA, ETS, ANN, SVR, Extreme Gradient Boosting (XGBoost), and Long Short-Term Memory (LSTM). ARIMA (p,d,q) [41–43] combines autoregressive (p), differencing (d), and moving average (q) terms, with SARIMA define as ARIMA $\left(p,d,q\right)(P,D,Q{)}_{s}$ [41–43] extending to seasonal components with seasonal period s, seasonal autoregressive (P), seasonal differencing (D), and seasonal moving average (Q) terms. ETS models provide a robust framework for capturing error, trend, and seasonal components, with specifications such as ETS(A,N,A) representing additive error, no trend, and additive seasonality, where smoothing parameters are optimized via maximum likelihood [43–49].

Machine learning approaches capture non-linear patterns: ANNs learn complex relationships through layered neurons [50–54]; SVR uses kernel functions (e.g., RBF) for high-dimensional non-linear regression, robust to overfitting [55–57]; XGBoost ensembles decision trees iteratively on residuals for scalable temporal dynamics [58]; LSTM networks employ memory cells and gates to handle long-term dependencies in sequential data like thunderstorm occurrences [11,58–61]. We used an ANN architecture consisting of four hidden layers with 128, 64, 32, and 16 units (dropout 0.3), while the LSTM architecture comprises three LSTM layers with 128, 64, and 32 units followed by a dense layer of 16 units (dropout 0.3) (Supplementary Tables S1 in S1 File).

#### 2.2.2. Models incorporating climate predictors.

We expanded the univariate framework by adding five exogenous environmental variables: temperature, relative humidity, cloud cover, rainfall, and air pressure to capture the meteorological determinants of thunderstorm activity. These predictors were selected based on their physical relevance to thunderstorm genesis and data availability. The inclusion of exogenous climatic variables in time series models has been widely applied in environmental research [62–64]. The six core algorithms were adapted to accept exogenous inputs, resulting in 18 model configurations. SARIMAX extends SARIMA by including climatic variables as exogenous regressors in a linear regression framework with ARIMA errors, and has been successfully applied to examine associations between climate variables and time series outcomes [62]. ETSX extends ETS by incorporating climatic variables as external regressors within the state space formulation and has been identified as optimal for specific applications when modeling relationships with exogenous climatic factors [62]. ANNX with exogenous inputs [65] includes climatic variables alongside lagged thunderstorm values and seasonal indicators to capture nonlinear interactions between meteorological conditions and thunderstorm occurrence. SVRX with exogenous inputs [63] adds climatic variables to the feature space, with kernel functions capturing nonlinear relationships between meteorological drivers and thunderstorm frequency. XGBoostX with exogenous inputs [66] incorporates climatic variables as additional features in the tree-based ensemble framework, and while widely used in environmental time series analysis, studies show that deep learning approaches can outperform tree-based methods when capturing long-term temporal dependencies [66]. LSTMX with exogenous inputs [64,66] incorporates climatic variables into the input sequence at each time step, allowing the network to learn temporal dependencies between meteorological conditions and thunderstorm occurrence. LSTM networks with embedded climate memory effects have been shown to outperform non-dynamic statistical models for capturing interannual and seasonal variations by effectively learning both short- and long-term dependencies [64,66].

#### 2.2.3. Seasonal decomposition methods.

Two seasonal decomposition methods were applied prior to modeling to isolate seasonal patterns and enhance forecast accuracy: X-11 [38,39] and STL [40]. The X-11 method is a widely used technique for both additive and multiplicative decompositions of quarterly and monthly data, developed from classical decomposition [39,67]. It allows the seasonal component to change gradually and provides trend-cycle estimates for all data points, including endpoints. Additionally, X-11 incorporates advanced strategies for handling trade day variations, holiday effects, and established predictors, and is robust against outliers [39,67]. The Seasonal-Trend decomposition using Loess (STL) applies Loess regressions and moving averages to break down a time series into trend-cycle, seasonal, and irregular components, accommodating both decomposition types [7,40,68]. It features a two-loop process that enhances robustness against outliers while identifying seasonal patterns, as each observation is weighted and regressed on local neighborhoods within the time series [7].

#### 2.2.4. Data Partitioning and cross-validation.

Monthly thunderstorm frequency data from January 1985 to December 2024 were partitioned into training (80%) and test (20%) sets to support rigorous model development and out-of-sample evaluation. Data from January 1985 to December 2016 (384 observations) were used for model development, hyperparameter tuning, and cross-validation. The remaining period from January 2017 to December 2024 (96 observations) was reserved exclusively as an unseen test set for final performance assessment, ensuring that all reported accuracy metrics reflect genuine out-of-sample predictive capability [67,69]. Given the temporal nature of the data, standard k-fold cross-validation is inappropriate as it ignores the sequential dependence structure [70]. We therefore implemented time-series cross-validation with an expanding window and rolling forecasting origin [71,72]. For each fold, models were trained on all available data up to a given cut-off point and then used to forecast subsequent steps ahead (1, 3, 6, and 12 months) to evaluate both short-term and multi-step forecasting capability [73]. The forecast errors from each fold were recorded, and the average multi-step forecast error across all windows served as the primary criterion for hyperparameter tuning and model selection [74]. This expanding window approach maximizes the use of available training data while maintaining temporal consistency and providing robust estimates of model generalizability [75]. After selecting the optimal model configuration for each approach, the final models were re-estimated on the full 1985–2016 dataset and evaluated on the unseen 2017–2024 test set for final out-of-sample performance assessment [76].

Model selection and hyperparameter tuning were performed using grid search combined with expanding window time series cross-validation (5 folds, 12-month forecast horizon). Supplementary Table S1 in S1 File presents the tuning ranges, optimal hyperparameter values, and validation performance (mean RMSE ± standard deviation across cross-validation folds) for all 18 univariate model configurations across the three stations. The same modeling framework and parameter specifications were applied consistently across all stations to ensure comparability of results. Automated selection procedures (AICc for SARIMA and ETS) determined optimal station-specific values within the specified ranges, allowing each model to adapt to the unique characteristics of each station’s thunderstorm time series. The results demonstrate that STL decomposition substantially improves forecast accuracy, with STL-XGBoost achieving the lowest validation errors across all stations (Sylhet: 2.53 ± 0.42; Sreemangal: 2.09 ± 0.35; Mymensingh: 2.08 ± 0.35). Raw LSTM models exhibit poor validation performance (RMSE > 7.5) with high variability, while X11 adjustment markedly improves their stability and accuracy. The hyperparameter tuning values for all exogenous models using climatic predictors are shown in Supplementary Table S2 in S1 File. These models extend the univariate framework by including five climatic variables—temperature, relative humidity, cloud cover, rainfall, and atmospheric pressure—as predictors. The same comprehensive feature engineering pipeline and parameter specifications were applied identically across all three stations to ensure consistency and comparability of model performance. STL-based models first decomposed the series, modeled the seasonally adjusted component, and then reintegrated the seasonal component for final forecasts. The consistent optimal hyperparameters across stations reflect the shared seasonal structure of thunderstorm frequency in northeastern Bangladesh.

Generation of multi-step forecasts (2025–2028): After model selection and hyperparameter tuning, the best-performing models for each station are retrained on the entire historical dataset (1985–2024) to maximize the use of available information. For univariate models, a recursive iterative strategy is employed, where each forecasted thunderstorm value serves as a lagged input for predicting subsequent months [67]. For exogenous models, a two-stage approach is used: first, the five climatic variables (temperature, relative humidity, cloud cover, rainfall, and atmospheric pressure) are forecasted recursively for 2025–2028 using the best-performing univariate models trained on their respective full historical data (1985–2024); second, these forecasted climatic variables are treated as known exogenous inputs and combined with recursively generated lagged thunderstorm values using the optimal exogenous model for each station to produce the final thunderstorm frequency forecasts for 2025–2028.

#### 2.2.5. Evaluation metrics.

Forecast accuracy was assessed using multiple complementary metrics calculated on out-of-sample forecasts (2017–2024). Scale-dependent measures included Mean Square Error (MSE), Root Mean Square Error (RMSE), and Mean Absolute Error (MAE), all expressed in original units [67,77] (thunderstorm frequency per month). The scale-free Mean Absolute Percentage Error (MAPE) and Mean Absolute Scaled Error (MASE) were also considered [67,77]:


$$\begin{array}{c} \text{MSE}=\frac{\text{1}}{n}\sum_{i=1}^{n}{\left(y_{i}-{\hat{y}}_{i}\right)}^{\text{2}}, \end{array}$$ (1)

$$\begin{array}{c} \text{MAE}=\frac{\text{1}}{n}\sum_{i=1}^{n}\left|y_{i}-{\hat{y}}_{i}\right|, \end{array}$$ (2)

$$\begin{array}{c} \text{MAPE}=\frac{\text{1}}{n}\sum_{i=1}^{n}\frac{\left|y_{i}-{\hat{y}}_{i}\right|}{y_{i}}\times 100\%, \end{array}$$ (3)

$$\begin{array}{c} \text{MASE}=\frac{\text{1}}{n}\sum_{i=1}^{n}\left(\frac{\left|y_{i}-{\hat{y}}_{i}\right|}{\frac{\text{1}}{n-m}\sum_{j=m+1}^{n}\left|y_{j}-y_{j-m}\right|}\right), \end{array}$$ (4)

$$\begin{array}{c} \text{RMSE}=\sqrt{\frac{\text{1}}{n}\sum_{i=1}^{n}{\left(y_{i}-{\hat{y}}_{i}\right)}^{\text{2}}}, \end{array}$$ (5)

where *n* indicates the number of observations, *m* is the seasonality value, $y_{i}\;$denotes the actual values, and ${\hat{y}}_{i}\;$represents the predicted values. MAPE was considered but deemed unsuitable as it produces undefined values when actual observations equal zero—a common occurrence in winter months when thunderstorm activity is absent. Additionally, MAPE asymmetrically penalizes errors, potentially introducing bias [77]. The combination of scale-dependent metrics and MASE provides a robust framework for evaluating model performance across Bangladesh’s thunderstorm-prone regions.

**Ethical statement:** Ethical approval was not necessary for this research since the dataset utilized does not include any human or animal participants.

## 3. Results

### 3.1. Long-term trends, seasonal dynamics, and decomposition of thunderstorm frequency

Understanding the fundamental characteristics of thunderstorm variability—its seasonal rhythm, long-term evolution, and underlying components—is essential for developing accurate predictive models in Bangladesh’s most thunderstorm-prone regions. This section presents a comprehensive decomposition analysis of monthly thunderstorm frequency across Sylhet, Sreemangal, and Mymensingh over the four-decade period from 1985 to 2024, examining both the dominant seasonal cycle and evidence of long-term change.

**Fig 3:**
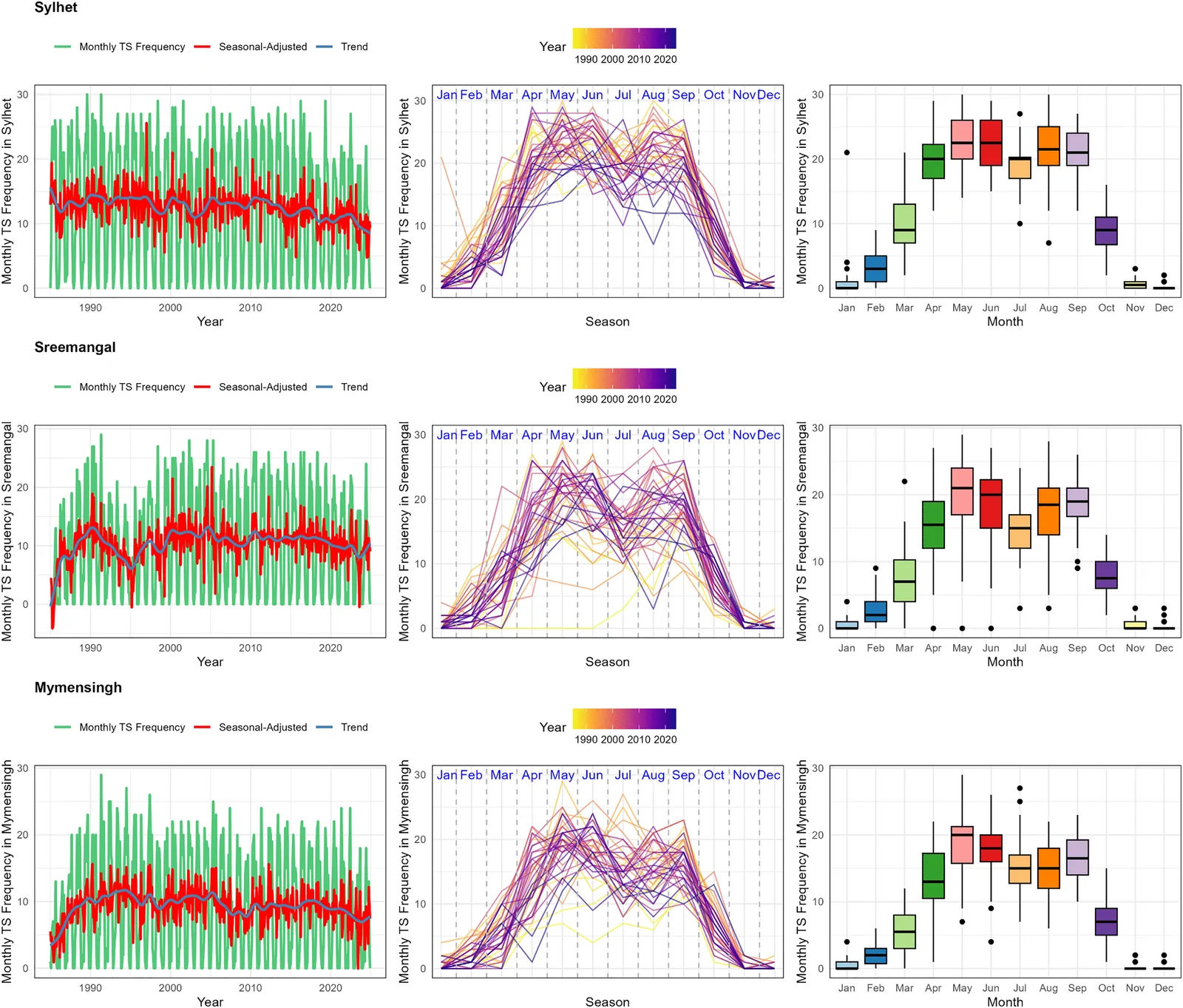
Seasonal decomposition and monthly distribution of thunderstorm frequencies in Sylhet, Sreemangal, and Mymensingh (1985–2024). The left panel for each station displays the original monthly thunderstorm series with an overlaid STL, showing the seasonally adjusted component (red) and the long-term trend (blue). The middle panel presents the raw monthly thunderstorm frequency, with a color gradient representing the progression of years from 1985 (yellow) to 2020 (purple). The right panel shows boxplots summarizing the monthly climatological distribution of thunderstorm frequency, highlighting the core pre-monsoon (March–May) and monsoon (June–September) seasons.

Fig 3 illustrates a multi-faceted view of thunderstorm variability across the three study stations over the four-decade period. A strong and consistent seasonal cycle, governed by Bangladesh’s distinct climatic regimes, is immediately apparent in all locations. Thunderstorm activity begins to rise in March with the onset of pre-monsoon convective conditions, peaks sharply between May and August—spanning the late pre-monsoon and peak monsoon periods—and then declines to a minimum during the post-monsoon and dry winter months (November–February). The boxplots in the right panel quantitatively confirm this bimodal but continuous thunderstorm season, identifying May and June as the absolute peak months across all stations (representing the pre-monsoon/monsoon transition), followed closely by April (pre-monsoon), July, and August (monsoon), while confirming the prolonged quiescent period in boreal winter.

The left panel in Fig 3 presents the STL decomposition of monthly thunderstorm frequency. The blue trend lines reveal distinct regional dynamics: Sylhet exhibits a relatively stable, slowly increasing trend throughout the study period; Sreemangal shows a gradual rise from the late 1980s to the early 2000s followed by a period of stabilization; and Mymensingh displays a stable but more variable interannual pattern, particularly during peak thunderstorm months. The red seasonally adjusted series further clarifies the underlying short-term fluctuations and irregular components by removing the dominant seasonal cycle. The middle panel displays raw monthly thunderstorm frequency with a color gradient transitioning from yellow (1985) to purple (2020), allowing for a visual inspection of interannual variability and long-term change (Fig 3). During the critical pre-monsoon (March–May) and monsoon (June–September) months, the darker purple lines are frequently positioned above the lighter yellow ones, suggesting an increase in thunderstorm frequency over the earlier part of the study period for all stations. Collectively, these visualizations in Fig 3 confirm the dataset’s suitability for advanced time-series analysis and underscore the necessity of incorporating seasonal decomposition techniques—specifically targeting the pre-monsoon and monsoon signals—for improving the interpretability and predictive accuracy of thunderstorm forecasting models in these climatologically vulnerable zones.

### 3.2. Model performance: Univariate framework

Model development was based on monthly thunderstorm frequency data collected between January 1985 and December 2016. We used time-series cross-validation with an expanding window to tune hyperparameters and compare model classes, with average multi-step forecast errors as the selection criterion. The final selected models were then re-estimated on the entire 1985–2016 dataset and tested on an unseen test set from January 2017 to December 2024, which was used solely for final out-of-sample performance evaluation. Table 2 summarizes a comprehensive comparison of forecast accuracy metrics (MSE, RMSE, MAE, MASE) for linear (ARIMA), non-linear (SARIMA, ETS), machine learning (ANN, SVR, XGBoost), and deep learning (LSTM) models, applied both to raw and seasonally-adjusted monthly thunderstorm frequency data across Sylhet, Sreemangal, and Mymensingh. The results consistently demonstrate that seasonal pre-adjustment substantially improves forecast performance. Models incorporating X11 and STL decomposition prior to forecasting systematically outperform their non-adjusted counterparts across all stations. For instance, the raw SARIMA model yields an MASE of 0.74 for Sylhet, while the STL-ARIMA seasonal-adjusted model reduces this to 0.45. Notably, the STL-XGBoost hybrid model achieves the lowest error metrics across all three locations (e.g., Sylhet: MSE = 6.40, RMSE = 2.53, MAE = 2.01, MASE = 0.43), representing a substantial improvement over raw SARIMA (MSE = 25.55) and raw XGBoost (MSE = 16.94) (Table 2). The LSTM models, while powerful in other contexts, exhibit poor performance on raw data (MASE > 1.40 across all stations), though seasonal adjustment via X11 markedly improves LSTM accuracy (e.g., Sylhet MASE reduced from 1.58 to 0.45) (Table 2). These findings underscore that explicitly modeling and removing seasonal components prior to forecasting is critical for accurate thunderstorm prediction, with tree-based ensemble methods (XGBoost) combined with STL decomposition emerging as the most robust approach across Bangladesh’s thunderstorm-prone regions.

**Table 2:** Forecast accuracy metrics for linear, non-linear, machine learning and deep learning models in predicting monthly thunderstorm frequency across Sylhet, Sreemangal, and Mymensingh. Models were applied to raw data and data pre-adjusted with X11 and STL seasonal decomposition methods.

Models	Sylhet				Sreemangal				Mymensingh			
	MSE	RMSE	MAE	MASE	MSE	RMSE	MAE	MASE	MSE	RMSE	MAE	MASE
SARIMA	25.5472	5.0544	3.4680	0.7387	15.5740	3.9464	2.7631	0.5807	12.5247	3.5390	2.5010	0.5425
ETS	18.9346	4.3514	3.1430	0.6695	14.7313	3.8381	2.8895	0.6073	10.9385	3.3073	2.2616	0.4905
ANN	30.2953	5.5041	4.0540	0.8635	23.6362	4.8617	3.3376	0.7015	14.7659	3.8426	2.5913	0.5620
SVR	43.7290	6.6128	4.8787	1.0392	35.0567	5.9209	4.3610	0.9166	31.5332	5.6154	4.2726	0.9267
XGB	16.9382	4.1156	3.2107	0.6839	10.8435	3.2929	2.5064	0.5268	9.1097	3.0182	2.1894	0.4749
LSTM	69.2222	8.3200	7.4217	1.5808	66.7211	8.1683	7.2180	1.5171	55.6320	7.4587	6.4571	1.4005
X11-ARIMA	13.7322	3.7057	3.0628	0.6524	12.8773	3.5885	2.7033	0.5682	8.2837	2.8781	2.1296	0.4619
X11-ETS	11.8912	3.4484	2.7791	0.5920	13.3205	3.6497	2.7525	0.5785	8.7600	2.9597	2.2128	0.4800
X11-ANN	18.2340	4.2701	3.2705	0.6966	20.4008	4.5167	3.4494	0.7250	17.6897	4.2059	3.4219	0.7422
X11-SVR	12.5018	3.5358	2.8496	0.6070	18.8120	4.3373	3.4242	0.7197	8.5546	2.9248	2.1088	0.4574
X11-XGB	11.2661	3.3565	2.5174	0.5362	15.1649	3.8942	2.8711	0.6034	7.6651	2.7686	1.9845	0.4304
X11-LSTM	8.8314	2.9718	2.1257	0.4528	14.1690	3.7642	2.8872	0.6068	21.8389	4.6732	3.5396	0.7677
STL-ARIMA	7.1070	2.6659	2.0895	0.4451	9.3547	3.0585	2.3746	0.4991	9.1116	3.0185	2.3628	0.5125
STL-ETS	7.4189	2.7238	2.1605	0.4602	9.5410	3.0889	2.4356	0.5119	6.6846	2.5855	1.9424	0.4213
STL-ANN	18.2228	4.2688	3.4414	0.7330	12.5561	3.5435	2.7920	0.5868	14.5290	3.8117	3.0633	0.6644
STL-SVR	10.0618	3.1720	2.6312	0.5604	7.8373	2.7995	2.1999	0.4624	7.0963	2.6639	1.9923	0.4321
STL-XGB	**6.3995**	**2.5297**	**2.0060**	**0.4273**	**4.3861**	**2.0943**	**1.5801**	**0.3321**	**4.3265**	**2.0800**	**1.4943**	**0.3241**
STL-LSTM	8.3455	2.8889	2.3106	0.4922	6.7864	2.6051	1.9679	0.4136	7.5838	2.7539	2.1164	0.4590

* Note: The best-performing model for each station and metric is highlighted in bold. Models with an “X11-” or “STL-” prefix were applied to data seasonally adjusted using the X11 or STL method, respectively. XGBoost is defined as XGB.

### 3.3. Influence of exogenous climatic drivers on thunderstorm frequency

**Fig 4:**
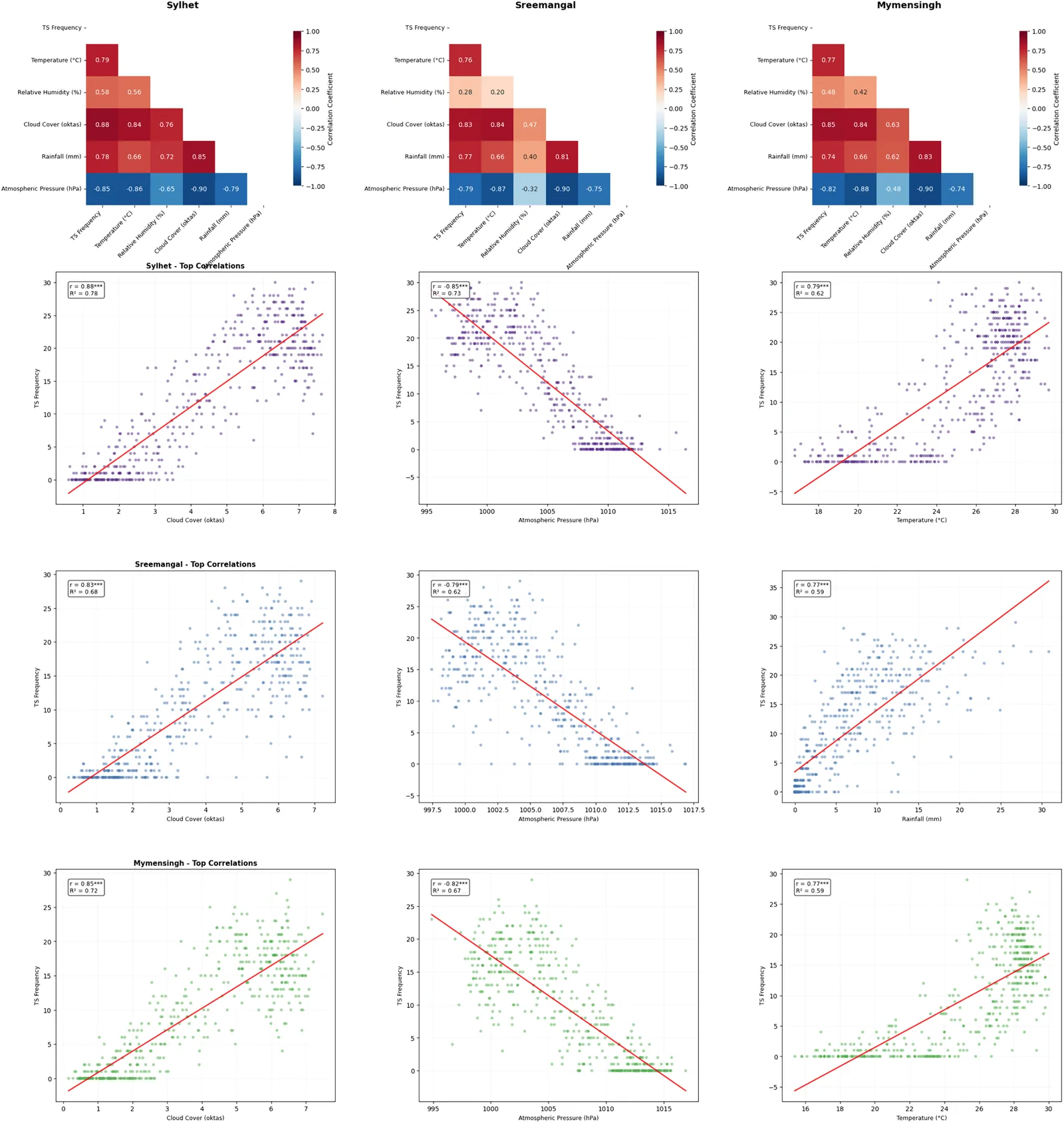
Correlation heatmap illustrating the relationship between monthly thunderstorm frequency and key exogenous climatic variables (temperature, relative humidity, cloud cover, rainfall, and atmospheric pressure) across Sylhet, Sreemangal, and Mymensingh (1985–2024). Cell values represent Pearson correlation coefficients, with color intensity indicating strength of association (blue: positive correlation; red: negative correlation). Scatter plots with regression lines illustrating the relationship between monthly thunderstorm frequency and the three most strongly correlated exogenous climatic variables for each station (1985–2024). For Sylhet (2^nd^ row): cloud cover (oktas), atmospheric pressure (hPa), and temperature (°C). For Sreemangal (3^rd^ row): cloud cover (oktas), atmospheric pressure (hPa), and rainfall (mm). For Mymensingh (4^th^ row): cloud cover (oktas), atmospheric pressure (hPa), and temperature (°C).

We deployed eighteen models, including classical (SARIMAX, ETSX), machine learning (XGBoostX, SVRX, ANNX), and deep learning (LSTMX) architectures, to raw and seasonally adjusted (X11, STL) data to forecast monthly thunderstorm frequency across Sylhet, Sreemangal, and Mymensingh. Each model included five exogenous climatic variables (temperature, relative humidity, cloud cover, rainfall, and atmospheric pressure) as predictors, with their interactions illustrated in Fig 4. Monthly data from January 1985 to December 2016 were used for model development with time-series cross-validation (expanding window) for hyperparameter tuning and model selection based on multi-step forecast errors. The final selected models were re-estimated on the full 1985–2016 dataset and evaluated on an unseen test dataset from January 2017 to December 2024 for out-of-sample performance assessment, with forecasting errors summarized in Table 3.

**Table 3:** Forecast accuracy metrics for linear, non-linear, ML and deep learning models with exogenous variables in predicting monthly thunderstorm frequency across Sylhet, Sreemangal, and Mymensingh. Models were applied to raw data and data pre-adjusted with X11 and STL seasonal decomposition methods.

Models	Sylhet				Sreemangal				Mymensingh			
	MSE	RMSE	MAE	MASE	MSE	RMSE	MAE	MASE	MSE	RMSE	MAE	MASE
SARIMAX	13.8459	3.7210	2.7414	0.4856	13.5547	3.6817	2.5301	0.4969	11.2287	3.3509	2.3860	0.5079
ETSX	20.8409	4.5652	3.4185	0.6055	14.6134	3.8227	2.6169	0.5139	10.1849	3.1914	2.2868	0.4868
XGBX	5.6666	2.3805	1.7895	0.3193	4.2633	2.0648	1.5124	0.2935	3.6136	1.9009	1.2761	0.2696
SVRX	12,0011	3.4643	2.7773	0.4929	4.2707	2.0666	1.5717	0.3023	3.0640	1.7504	1.3718	0.2858
ANNX	10.0264	3.1664	2.2895	0.4066	6.8823	2.6234	1.8666	0.3567	4.7929	2.1893	1.6681	0.3463
LSTMX	20.7164	4.5515	3.3564	0.5946	16.5962	4.0738	3.0107	0.5763	17.9554	4.2374	3.1124	0.6468
X11-ARIMAX	19.8230	4.4523	3.2631	0.5780	15.1788	3.8960	2.7794	0.5459	11.2040	3.3472	2.4569	0.5230
X11-ETSX	21.6599	4.6540	3.3934	0.6011	15.1769	3.8958	2.8020	0.5503	9.5012	3.0824	2.1807	0.4642
X11-XGBX	4.8239	2.1963	1.4540	0.2594	2.7459	1.6571	1.0921	0.2119	2.3263	1.5252	0.9612	0.2031
X11-SVRX	5.5806	2.3623	1.5291	0.2728	2.5462	1.5957	1.0774	0.2090	2.4598	1.5684	0.8522	0.1801
X11-ANNX	4.0103	2.0026	1.4741	0.2630	6.7431	2.5968	1.9388	0.3762	8.8941	2.9823	2.1671	0.4579
X11-LSTMX	3.2859	1.8127	1.3860	0.2473	4.7171	2.1719	1.6095	0.3123	9.4217	3.0695	2.2984	0.4857
STL-ARIMAX	12.3328	3.5118	2.7365	0.4854	12.8272	3.5815	2.5203	0.4811	8.2808	2.8776	2.2006	0.4590
STL-ETSX	15.0044	3.8736	3.2388	0.5745	13.1067	3.6203	2.6703	0.5097	8.8085	2.9679	2.2056	0.4601
STL-XGBX	**3.1910**	**1.7863**	**1.2502**	**0.2214**	2.2318	1.4939	1.0530	0.2015	2.1321	1.4602	0.9818	0.2040
STL-SVRX	4.5291	2.1282	1.6378	0.2901	**2.1521**	**1.4670**	**0.8964**	**0.1715**	**1.5982**	**1.2642**	**0.8346**	**0.1734**
STL-ANNX	3.4650	1.8615	1.3899	0.2462	3.5385	1.8811	1.4406	0.2757	1.6310	1.2771	1.0290	0.3029
STL-LSTMX	13.0507	3.6126	3.0295	0.5409	11.3768	3.3729	2.3762	0.4663	9.0583	3.0097	2.3772	0.4984

* Note: The best-performing model for each station and metric is highlighted in bold. Models with an “X11-” or “STL-” prefix were applied to data seasonally adjusted using the X11 or STL method, respectively. XGBoostX is defined as XGBX.

To understand the underlying meteorological drivers of thunderstorm activity, we examined the correlation between monthly thunderstorm frequency and five exogenous climatic variables: temperature, relative humidity, cloud cover, rainfall, and atmospheric pressure using heatmaps (Fig 4). Temperature exhibits a strong-positive significant correlation with thunderstorm frequency (r≈ 0.77–0.79 across stations), reflecting the role of surface heating in generating convective instability during the pre-monsoon period. Cloud cover (r≈ 0.83–0.88) and rainfall (r≈ 0.74–0.78) also show strong positive associations, consistent with the coupled nature of convective processes where thunderstorm produce extensive cloudiness and precipitation. Relative humidity demonstrates a weak to moderate positive correlation (r≈ 0.28–0.58), highlighting the importance of moisture availability for convective development, particularly during the monsoon season when moisture-laden air masses from the Bay of Bengal prevail. Conversely, atmospheric pressure exhibits a strong negative correlation with thunderstorm frequency (r≈ −0.79–0.85), reflecting the association between thunderstorm activity and low-pressure systems, troughs, and convective instability.

Scatter plots with regression lines for the three most highly associated variables at each station are also shown in Fig 4, which shows both consistent patterns and notable regional variations. Cloud cover (r= 0.88 and p -value < 0.001), atmospheric pressure (r= −0.85 and p -value < 0.001), and temperature (r= 0.79 and p -value < 0.001) were the top three associated factors for Sylhet. For Sreemangal, the main drivers were cloud cover r= 0.83 and p -value < 0.001), atmospheric pressure (r= – 0.79 and p -value < 0.001), and rainfall (r= 0.77 and p -value < 0.001). The top three variables for Mymensingh were similar to those for Sylhet: cloud cover (r= 0.85 and p -value < 0.001), air pressure (r= −0.82 and p -value < 0.001), and temperature (r= 0.77 and p -value < 0.001). These correlation patterns are remarkably consistent across Sylhet, Sreemangal, and Mymensingh, suggesting that the fundamental thermodynamic and dynamic drivers of thunderstorm genesis operate uniformly across northeastern Bangladesh’s thunderstorm-prone regions. The consistency of these correlation patterns—particularly the universal importance of cloud cover and atmospheric pressure—suggests that the fundamental dynamic and thermodynamic drivers of thunderstorm genesis operate uniformly across northeastern Bangladesh’s thunderstorm-prone regions, while station-specific variations in the ranking of temperature versus rainfall reflect local climatological nuances. Building upon the strong associations identified in Fig 4, we incorporated all five exogenous climatic variables as predictors in our forecasting models to assess their contribution to predictive accuracy. Table 3 presents forecast accuracy metrics for different models that include these five exogenous climatic variables, applied both to raw and seasonal-adjusted thunderstorm data.

Table 3 reveals that the inclusion of exogenous variables generally improves forecast performance for many model configurations compared to the univariate models presented in Table 2. For example, the raw XGBoostX model with exogenous inputs achieves substantially lower errors (e.g., Sylhet: MSE = 5.6666, MASE = 0.3193 in Table 3) than its univariate counterpart (MSE = 16.9382, MASE = 0.6839 in Table 2), demonstrating the improved forecast value of incorporating meteorological factors for tree-based methods. Across all stations, seasonal pre-adjustment using STL decomposition consistently outperforms both raw and X11-adjusted approaches for most models, confirming that explicitly modeling the seasonal structure prior to forecasting yields superior predictive accuracy (Table 3). However, the improvement from exogenous variables is not universal. For instance, while ETSX improved over ETS in Sylhet (MASE: 0.6055 vs. 0.6695), STL-LSTMX consistently performed worse than STL-LSTM across all three stations (e.g., Sylhet MASE: 0.5409 vs. 0.4922). Similarly, X11-ARIMAX performed worse than ARIMAX in all cities, indicating that seasonal adjustment via X11 does not always enhance exogenous models.

The most notable improvements emerge when exogenous variables are combined with STL seasonal pre-adjustment. The STL-XGBoostX model with exogenous predictors demonstrates the best performance for Sylhet (MSE = 3.1910, RMSE = 1.7863, MAE = 1.2502, MASE = 0.2214), reflecting over an 80% reduction in MASE compared to the univariate SARIMA model (MASE = 0.7387) and a significant enhancement over the best univariate STL-XGBoost model (MASE = 0.4273) in Table 2. For both Sreemangal and Mymensingh, STL-SVRX achieves the lowest errors overall (e.g., Sreemangal: MSE = 2.1521, RMSE = 1.4670, MAE = 0.8964, MASE = 0.1715; Mymensingh: MSE = 1.5982, RMSE = 1.2642, MAE = 0.8346, MASE = 0.1734), marginally outperforming STL-XGBoostX (e.g., Sreemangal: MSE = 2.2318, RMSE = 1.4939, MAE = 1.0530, MASE = 0.2015; Mymensingh: MSE = 2.1321, RMSE = 1.4602, MAE = 0.9818, MASE = 0.2040). These exceptions suggest that the added value of climatic variables is model-dependent, with tree-based and kernel methods (XGBoostX, SVRX) benefiting more substantially than recurrent architectures (LSTMX).

### 3.4. Projected thunderstorm frequency and seasonal forecasts (2025–2028)

**Fig 5:**
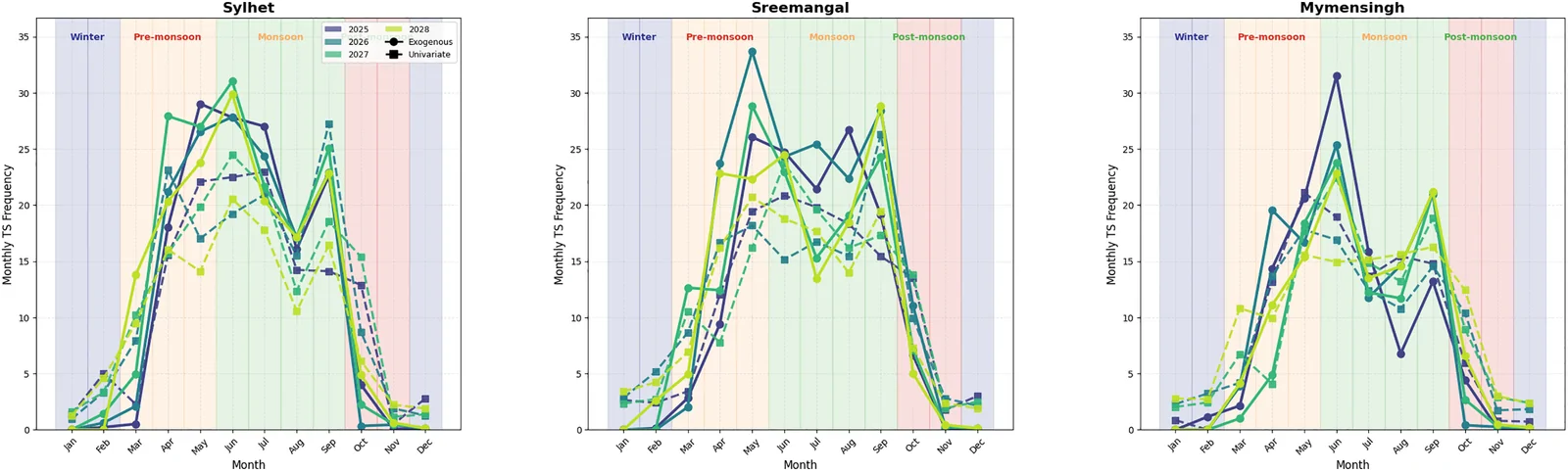
Monthly thunderstorm frequency forecasts for Sylhet, Sreemangal, and Mymensingh (2025–2027) from optimal models incorporating exogenous climatic variables. Solid lines represent forecasts from the best-performing exogenous model for each station (STL-XGBoostX for Sylhet; STL-SVRX for Sreemangal and Mymensingh), while dashed lines show forecasts from the optimal univariate model (STL-XGBoost for all stations).

**Fig 6:**
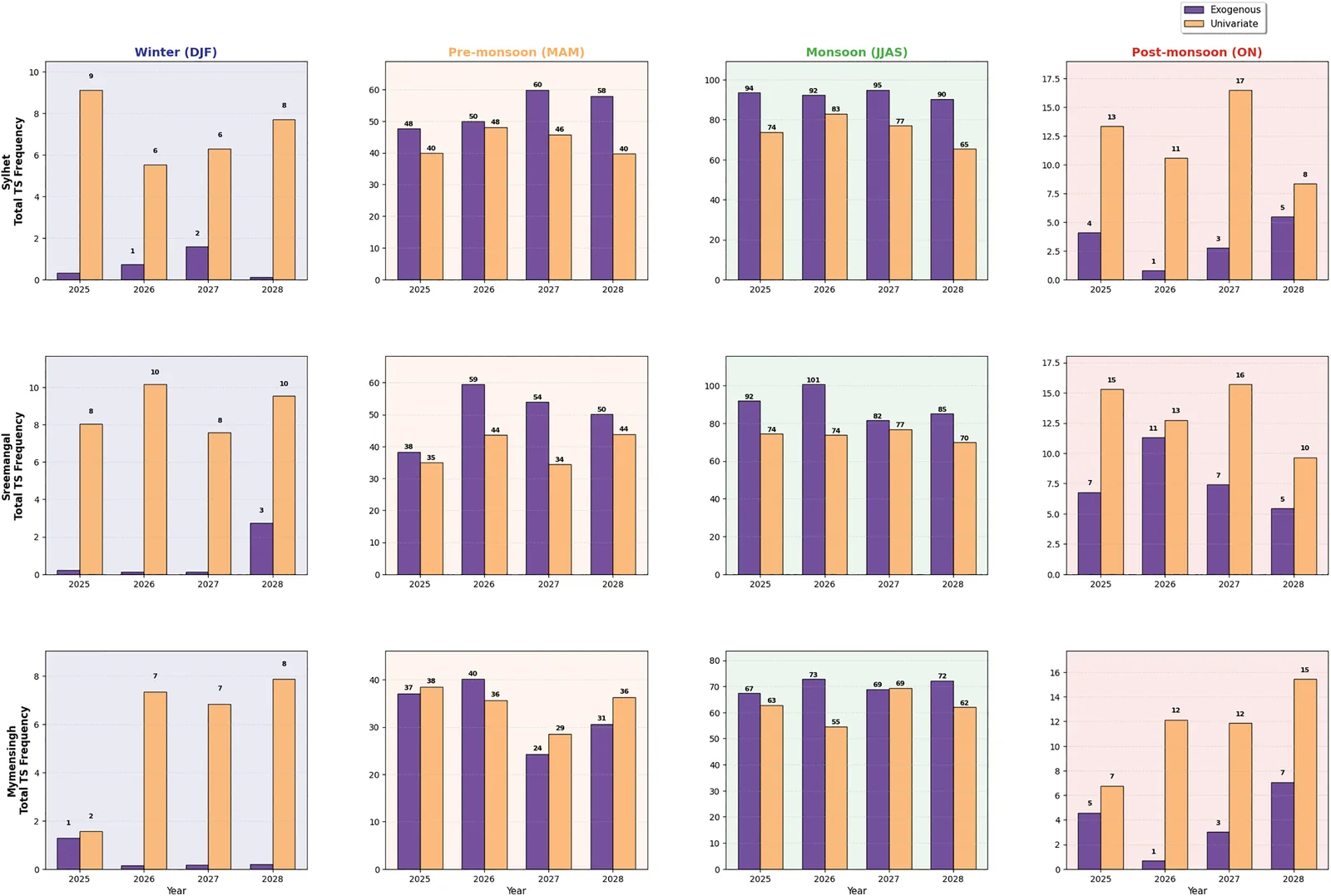
Seasonal aggregate thunderstorm frequency forecasts for Sylhet, Sreemangal, and Mymensingh (2025–2028). Total forecasted thunderstorm frequency were aggregated by meteorological season: Winter (December–February), Pre-monsoon (March–May), Monsoon (June–September), and Post-monsoon (October–November). Light orange bars represent forecasts from the optimal univariate model (STL-XGBoost for all stations); purple bars represent forecasts from the optimal model incorporating exogenous climatic variables (STL-XGBoostX for Sylhet; STL-SVRX for Sreemangal and Mymensingh), demonstrating the value of including meteorological drivers in seasonal thunderstorm prediction.

Table 3 illustrates the optimal forecasting models for the three study stations. For Sylhet, univariate forecasts utilized the STL-XGBoost model, while exogenous forecasts incorporated the STL-XGBoost with climatic variables. In contrast, Sreemangal and Mymensingh followed the univariate approach using STL-XGBoost, but exogenous forecasts adopted STL-SVR with climatic variables. Building on these optimal models, we generated monthly thunderstorm frequency forecasts for 2025–2028 (Fig 5) and aggregated these into seasonal totals for 2025–2028 (Fig 6 and Supplementary Table S3 in S1 File) across all three stations.

Fig 5 presents monthly forecasts from both exogenous (solid lines) and univariate (dashed lines) models, revealing a pronounced and consistent seasonal cycle across all stations. Thunderstorm activity begins escalating in March, peaks during May–September (with June reaching 29.0 frequency), and declines to minimal values during winter. The close agreement between exogenous and univariate forecasts in timing confirms that the seasonal signal is the dominant source of predictability. However, notable differences emerge in magnitude: exogenous models project slightly higher peak values for Sylhet during pre-monsoon months, while for Sreemangal and Mymensingh, the STL-SVRX exogenous forecasts exhibit tighter prediction ranges, reflecting SVR’s regularization properties. Importantly, exogenous models consistently project lower winter and post-monsoon baselines than univariate models, demonstrating that including meteorological drivers better captures near-zero activity during quiescent periods when convective potential is limited by atmospheric conditions.

**Fig 7:**
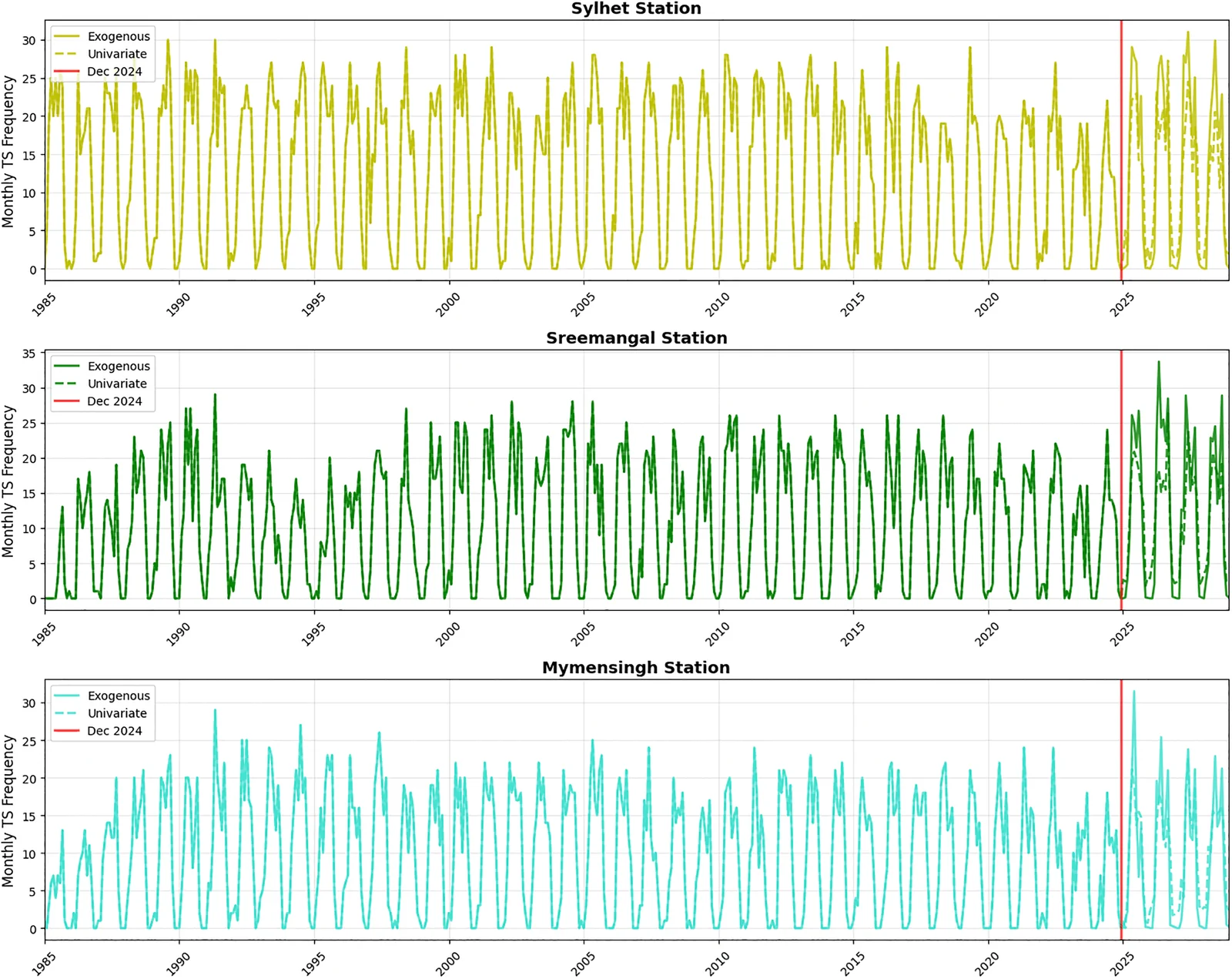
Long-term historical and forecasted monthly thunderstorm frequency trend for Sylhet, Sreemangal, and Mymensingh (1985–2028) using univariate and exogenous model frameworks.

Fig 6 and Supplementary Table S3 in S1 File provide seasonal aggregate forecasts, enabling assessment of interannual variability and model performance across meteorological seasons. During the pre-monsoon season, exogenous forecasts show a progressive increase for Sylhet from 47.6 frequency (2025) to 59.9 frequency (2027), while Sreemangal peaks at 59.4 frequency (2026) and Mymensingh ranges from 24.3–40.1 frequency. The monsoon season accounts for the largest share of annual activity, with Sreemangal reaching 100.5 frequency in 2026—the highest seasonal value across all stations—followed by Sylhet (90.3–94.9 frequency) and Mymensingh (67.4–72.8 frequency). Winter and post-monsoon seasons show minimal activity across all stations, with exogenous forecasts rarely exceeding 1–2 total frequency, confirming the strong seasonal concentration of thunderstorm activity. The long-term annual forecasts presented in Fig 7 support this seasonal analysis by showing a consistent increasing trend, providing essential context for understanding seasonal distribution patterns. However, this forecast skill degrades rapidly with increasing lead time, and these projections are presented as illustrations of model behavior rather than reliable long-term predictions. Notably, in the held-out test period (2017–2024), exogenous models consistently produced lower totals than univariate models during non-peak seasons and higher totals during peak seasons, suggesting that meteorological drivers effectively constrain forecasts when thunderstorm-favorable conditions are absent and amplify them when such conditions are present. The same pattern persists in the 2025–2028 forecasts (Figs 6,7), but these projections are presented as illustrations of model behavior rather than as verified predictions. We note that the apparent contradiction between the historical decreasing trend at Sylhet and the projected increase in later forecast years is a property of the fitted statistical model rather than a demonstrated physical mechanism; we have not established a causal link to warming in this study, and this discrepancy is flagged as an open question for future work combining longer forecast validation with mechanistic climate analysis.

## 4. Discussion

While cyclones and floods have historically dominated climate risk discourse, thunderstorms represent one of the most significant convective hazards in Bangladesh, with documented impacts on public health, agriculture, and infrastructure [8,78,79]. Climate change projections and global studies suggest that warming conditions may intensify convective activity in many regions [12–15], though station-level trend analyses in Bangladesh show heterogeneous patterns that warrant continued monitoring and localized forecasting efforts. Our own analysis reveals a statistically significant decreasing trend at Sylhet, while Sreemangal and Mymensingh show no significant trends over the 1985–2024 period. Despite these heterogeneous trends, the development of accurate station-specific forecasting models remains essential for early warning and disaster preparedness in these vulnerable regions. Thunderstorms are among the most hazardous convective phenomena globally, with an estimated 1,600–2,400 active storms at any moment, totaling nearly 50,000 daily occurrences worldwide [5, 80-81–]. Their rapid onset and destructive impacts pose significant meteorological and societal challenges. In Bangladesh, thunderstorms are increasingly linked to fatalities from lightning strikes, injuries, infrastructure damage, power outages, and adverse effects on agriculture and transportation [79,82]. Despite these growing threats, thunderstorm research in Bangladesh has remained largely focused on climatological description rather than the development and rigorous evaluation of station-specific forecasting tools. This study addresses that gap by developing and validating station-specific forecasting models for three high-risk stations in northeastern Bangladesh—Sylhet, Sreemangal, and Mymensingh—using 40 years (1985–2024) of monthly observational data.

The long-term monthly mean thunderstorm frequency confirm that Sylhet experiences the highest frequency (12.56 frequency per month), followed by Sreemangal (10.32) and Mymensingh (9.35) (Table 1). These differences are consistent with the stations’ varying proximity to the Meghalaya Plateau and associated orographic forcing mechanisms, which enhance convective processes through mechanical lifting of moisture-laden air masses [19,20,33]. Sylhet’s location in a basin bordered by the Meghalaya and Tripura hills fosters enhanced trapping and uplift of moist air, while proximity to the extremely wet highlands of Cherrapunji and Mawsynram ensures a continuous moisture supply [33,34]. Trend analysis using the Mann-Kendall test and Sen’s slope estimator revealed a statistically significant decreasing trend only at Sylhet (τ = −0.076, p -value = 0.015; Sen’s slope = −0.0037 thunderstorm frequency per month, 95% CI: −0.0094 to 0.0000), equivalent to a decline of approximately 1.75 thunderstorm frequency per decade. Sreemangal showed no significant trend (τ = 0.046, p -value = 0.145; Sen’s slope = 0.0000, 95% CI: 0.0000 to 0.0054), and Mymensingh likewise showed no significant trend (τ = −0.0215, p -value = 0.497; Sen’s slope = 0.0000, 95% CI: −0.0027 to 0.0000) (Table 1). These findings demonstrate that claims of ubiquitous thunderstorm intensification in Bangladesh are not supported by station-level occurrence data from these three locations. Contrary to Wahiduzzaman et al. [26], who reported widespread significant increases in thunderstorm frequency across Bangladesh with the largest increases in the northeastern region, our analysis of three key stations in that region—using a longer and more recent dataset (1985–2024) and rigorous trend statistics with confidence intervals—reveals either no change or a modest decline. This discrepancy suggests that earlier claims of systematic thunderstorm intensification may be period-dependent, sensitive to aggregation methods, or no longer hold under recent climate conditions. It underscores the critical importance of disaggregated, station-level analysis rather than regional aggregation, which may mask heterogeneous local patterns or produce statistical artifacts.

Seasonal analysis revealed pronounced and consistent seasonality at all stations, with peak activity concentrated in April–May and June–August (Fig 3). The boxplots in Fig 3 quantitatively confirm May and June as the absolute peak months across all stations, representing the pre-monsoon/monsoon transition, followed closely by April, July, and August. March and September contribute relatively few thunderstorm days compared to the peak months. This is not a correction to established climatology but rather a local-scale operational insight: for these specific locations, enhanced monitoring and preparedness efforts should be concentrated in April–August, with March and September representing transitional periods of lower risk.

The correlation analysis (Fig 4) revealed strong and physically interpretable relationships between thunderstorm frequency and climatic variables. Cloud cover exhibited the strongest positive correlation across all stations (r = 0.83–0.88, p -value < 0.001), reflecting the intrinsic connection between thunderstorm development and extensive convective cloud systems. Atmospheric pressure showed a strong negative correlation (r = −0.79 to −0.85, p -value < 0.001), consistent with the association between thunderstorm activity and low-pressure systems, troughs, and convective instability. Temperature demonstrated strong positive correlations (r = 0.77–0.79, p -value < 0.001) for Sylhet and Mymensingh, underscoring the role of surface heating in generating CAPE during the pre-monsoon season. For Sreemangal, rainfall emerged as the third most strongly correlated variable (r = 0.77, p -value < 0.001), suggesting that thunderstorm activity at this station may be more tightly coupled to moist convective processes and precipitation intensity, consistent with its location in a region renowned for high rainfall totals. These correlation patterns are remarkably consistent across stations, suggesting that the fundamental thermodynamic and dynamic drivers of thunderstorm genesis operate uniformly across northeastern Bangladesh’s thunderstorm-prone regions. The station-specific variations in the ranking of temperature versus rainfall reflect local climatological nuances, with Sylhet and Mymensingh exhibiting stronger thermal forcing while Sreemangal shows greater moisture coupling.

The study’s key contribution is the systematic comparison of thirty six modeling approaches across two frameworks—univariate (SARIMA, ETS, SVR, ANN, XGBoost, LSTM) and exogenous (SARIMAX, ETSX, SVRX, ANNX, XGBoostX, LSTMX) with X-11 and STL decompositions—enabling robust quantification of the value added by meteorological information and seasonal adjustment. Forecasting model comparison was conducted using monthly data from January 1985 to December 2016 for model development, with time-series cross-validation (expanding window) for hyperparameter tuning. The final selected models were re-estimated on the full 1985–2016 dataset and evaluated on an unseen test set from January 2017 to December 2024, reserved exclusively for out-of-sample performance assessment. Among the evaluated methods in the univariate framework, XGBoost consistently outperformed ARIMA, ETS, ANN, SVR, and LSTM on out-of-sample test data. The deep learning model, LSTM, exhibited poor performance (MASE > 1.40), confirming that monthly thunderstorm frequency data lack the sequential complexity or sample size required for deep learning to outperform simpler methods; however, seasonal adjustment via X-11 markedly improved LSTM accuracy (e.g., Sylhet MASE reduced from 1.58 to 0.45), demonstrating that even complex architectures benefit from prior removal of seasonal structure. More importantly, seasonal adjustment using STL decomposition substantially improved forecast accuracy for all models. The STL-XGBoost seasonal-adjusted model achieved 41–45% lower RMSE compared to raw XGBoost across all three stations, reducing RMSE from 4.1156 to 2.5297 at Sylhet, 3.32929 to 2.0943 at Sreemangal, and 3.0182 to 2.0800 at Mymensingh. STL outperformed X-11 because its locally weighted regression approach allows the seasonal component to evolve gradually over time, whereas X-11 estimates a fixed seasonal pattern with more limited flexibility [58]. The STL-XGBoost model consistently outperformed others, reducing MSE by over 65% relative to unadjusted models in univariate framework (Table 2).

The inclusion of five exogenous climatic variables—temperature, relative humidity, cloud cover, rainfall, and atmospheric pressure—dramatically improved forecast performance compared to univariate models. Raw XGBoostX with exogenous inputs achieved substantially lower errors (MASE = 0.3193) for Sylhet than its univariate counterpart (MASE = 0.6839), demonstrating the critical role of meteorological drivers in explaining thunderstorm variability. The most notable improvements emerged when exogenous variables were combined with STL seasonal pre-adjustment, with station-specific optimal configurations reflecting local climatological nuances. For Sylhet, STL-XGBoostX with exogenous predictors achieved the lowest errors (MASE = 0.2214)—an 80% reduction compared to univariate SARIMA (MASE = 0.7387). STL-SVRX with exogenous inputs proved optimal for Sreemangal (MASE = 0.1715) and Mymensingh (MASE = 0.1734), marginally outperforming STL-XGBoostX, which yielded MASE values of 0.2015 and 0.2040, respectively (Table 3). The superiority of SVRX at these stations likely reflects smoother, more regular relationships between meteorological drivers and thunderstorm occurrence, whereas Sylhet’s topographically forced convection benefits from XGBoostX’s ability to capture complex nonlinear interactions. Forecasts for 2025–2028 project a pronounced seasonal cycle with peak activity during May–September, while exogenous models consistently project lower winter and post-monsoon baselines than univariate models, demonstrating that meteorological drivers effectively constrain forecasts during quiescent periods when convective potential is limited. The long-term annual forecast for Sylhet (2025–2049) projects a gradual increasing trend (Fig 7), rising from approximately 62–86 days annually. This extrapolated increase is a statistical projection of the fitted model rather than a validated forecast or a claim about underlying physical drivers, and it should be interpreted with caution given that it runs counter to the significant historical decreasing trend detected at Sylhet over 1985–2024 (Section 3.1). We flag this discrepancy as an open question for future work combining longer forecast validation with mechanistic climate analysis, rather than resolving it here.

Our findings both converge with and diverge from existing literature on thunderstorm frequency forecasting in Bangladesh and South Asia. Previous studies highlighted the efficacy of advanced ML and thermodynamic indicators in predicting pre-monsoon thunderstorm in India and China [24,81]. Locally, research has mainly concentrated on thunderstorm climatology [25,27,29], lightning forecasting [27], and socio-economic impacts on health, agriculture, and fisheries [8]. Large-scale climate drivers such as ENSO and IOD have been implicated in shaping thunderstorm spatiotemporal variability through numerical modeling approaches including ARW-WRF [27–29]. Prior time-series forecasting studies in Bangladesh have either aggregated data across all 34 BMD stations before modeling or applied ARIMA and SARIMA [24,25,31] without systematic comparison to ML alternatives or seasonal adjustment. However, such pooling may mask unique localized patterns and reduce forecast specificity. Islam et al. [24] reported SARIMA-based forecasts for aggregated divisional data; our study demonstrates that station-specific modeling reveals heterogeneous patterns masked by aggregation. The integration of seasonal adjustment techniques with both traditional and contemporary ML models—and their systematic comparison under a rigorous evaluation framework—represents a methodological contribution specific to the Bangladesh thunderstorm forecasting literature and a novel methodological contribution that enhances early warning capabilities and targeted disaster mitigation.

While this study offers valuable insights into thunderstorm forecasting in high-risk regions of Bangladesh, it is important to acknowledge several limitations. We recognize that this study is primarily a model benchmarking exercise, and that advancing mechanistic understanding of thunderstorm processes would require a different analytical approach, including higher-frequency data. Monthly frequency forecasts cannot directly support the imminent threat warnings—minutes to hours—that are most critical for life-saving actions during individual thunderstorm events. However, monthly outlooks serve a complementary function: they inform preparedness planning at weekly to monthly lead times. In the short term, the BMD could implement the STL-XGBoostX and STL-SVRX frameworks in parallel testing mode alongside existing operational forecasts, allowing direct skill comparison and staff training. In the long term, following successful prototype evaluation, the approach could be extended to additional stations and integrated into BMD’s operational forecasting suite. The analysis was spatially limited to three meteorological stations due to data constraints; broader spatial coverage would enable a more comprehensive mapping of thunderstorm risk zones nationwide. Socio-economic data—including population, livelihoods, lightning mortality, infrastructure exposure, and disaster impacts—are not available at the station level or with the temporal continuity required for the 40-year study period. The BMD stations record only meteorological variables; socio-economic data collected by the Bangladesh Bureau of Statistics (BBS) are reported at district or sub-district levels with varying geographic boundaries and temporal coverage that do not align with our station locations or continuous time series. Lightning mortality statistics became systematically available only after lightning was officially declared a natural disaster in 2016; pre-2016 records are incomplete and inconsistently documented. Consequently, we cannot assess the societal impacts of thunderstorm variability or the potential benefits of improved forecasts for specific vulnerable groups. However, observational and media reports confirm that thunderstorm‑associated lightning frequently causes fatalities in our three study regions: multiple deaths have been reported in Sylhet [83] and Moulvibazar (Sreemangal) during individual storm episodes [84], and similar fatal lightning strikes have killed residents in Mymensingh District [85]. Survey‑based research [2] further highlights high lightning mortality among farmers and fishermen in the northeastern districts including Sylhet and Moulvibazar, underscoring the acute human vulnerability in these areas. Hence, the qualitative discussion of socio-economic vulnerability relies on recent literature rather than time-varying covariates. Future discussions of public health, infrastructure resilience, or agricultural adaptation depend on interdisciplinary data collection efforts.

Within these boundaries, this study demonstrates that STL decomposition combined with XGBoostX and SVRX, incorporating exogenous climatic variables, significantly improves monthly thunderstorm forecast accuracy at three high-risk stations in Bangladesh. STL-XGBoostX with climate predictors achieved the lowest errors for Sylhet (MASE = 0.22), while STL-SVRX with climate predictors proved optimal for Sreemangal and Mymensingh (MASE = 0.17), representing over 80% improvement compared to univariate benchmarks. The optimal models identified (STL-XGBoostX for Sylhet; STL-SVRX for Sreemangal and Mymensingh) are computationally lightweight, requiring only a few seconds to execute on a standard desktop computer for the monthly dataset. However, comprehensive benchmarking of computational trade-offs across all models, while beyond the current scope, would be a valuable direction for future work. These findings offer vital opportunities to reduce public health risks and mitigate disaster impacts. Reliable forecasts can facilitate timely community alerts, support infrastructure resilience through informed urban planning, and enhance health preparedness via proactive campaigns [86, 87]. Long-term climate adaptation—including reforestation, wetland restoration, and sustainable agriculture—remains essential for community resilience [88–91]. However, realizing these benefits requires sustained interdisciplinary collaboration and addressing fundamental data limitations.

Future research should focus on hourly thunderstorm occurrence data, expanded spatial coverage to all meteorological stations across Bangladesh, and integration of satellite remote sensing and reanalysis products (e.g., ERA5, GPM, MODIS) to strengthen prediction robustness [92–94]. Investigating large-scale climate drivers (ENSO, IOD, MJO) using hybrid models and incorporating climate change scenarios, e.g., Representative Concentration Pathways (RCPs) and Shared Socioeconomic Pathways (SSPs), would improve long-term trend and vulnerability assessments [95,96]. Critically, future work must integrate meteorological analysis with systematic, spatially referenced socio-economic data collection—including lightning fatalities, infrastructure damage, and population exposure—to translate forecasting improvements into measurable risk reduction outcomes. This requires sustained interdisciplinary collaboration between the BMD, the Department of Disaster Management (DDM), the BBS, and academic researchers. Collaboration with policymakers, NGOs, and the BMD will be vital to translate these scientific advances into practical risk reduction and resilience-building strategies nationwide. Without such integration, forecasting skill remains an academic exercise rather than a tool for societal protection. We offer this study as a reproducible template for station-specific forecast development that can be critiqued, improved, and extended by the research community, with the hope that future interdisciplinary collaborations will build upon this foundation to develop forecasting systems that are both statistically skillful and operationally beneficial for Bangladesh’s most thunderstorm-vulnerable communities.

## 5. Conclusion

This study developed and rigorously evaluated localized thunderstorm forecasting models for three high-risk stations in northeastern Bangladesh—Sylhet, Sreemangal, and Mymensingh—using univariate (SARIMA, ETS, SVR, ANN, XGBoost, LSTM) and exogenous (SARIMAX, ETSX, SVRX, ANNX, XGBoostX, LSTMX) frameworks with X-11 and STL decompositions based on 40 years of monthly data. Correlation analysis identified cloud cover (r = 0.83–0.88), atmospheric pressure (r = −0.79 to −0.85), temperature (r = 0.77–0.79), and rainfall (r = 0.74–0.78) as dominant meteorological drivers. Seasonal pre-adjustment using STL decomposition substantially improved forecast accuracy, with STL-XGBoost achieving the lowest univariate errors (RMSE = 2.08–2.53). Inclusion of exogenous climatic variables dramatically enhanced predictive skill with tree-based and kernel methods: STL-XGBoostX with climate predictors proved optimal for Sylhet (MASE = 0.22), while STL-SVRX with climate predictors was optimal for Sreemangal and Mymensingh (MASE = 0.17), representing over 80% improvement compared to univariate benchmarks.

For decision-makers, these findings have direct operational implications. The BMD can implement the validated STL-XGBoostX and STL-SVRX frameworks to enhance station-specific forecasts, with seasonal projections identifying April–August as the critical preparedness window for targeted resource allocation, public awareness campaigns, and health system preparedness. The demonstrated value of integrating climatic variables—cloud cover, atmospheric pressure, temperature, and rainfall—provides a scientific basis for investing in quality-controlled meteorological data collection to improve forecast accuracy. This study provides BMD with a reproducible, evidence-based pathway to enhance early warning systems, reduce lightning fatalities among vulnerable groups (particularly farmers and fishermen), and inform climate-resilient infrastructure planning. Realizing these benefits requires sustained interdisciplinary collaboration between BMD, disaster management authorities, and academic researchers to translate forecasting improvements into measurable risk reduction outcomes for Bangladesh’s most thunderstorm-vulnerable communities.

## Supporting information

S1 File Supplementary tables for model tuning, validation, and thunderstorm frequency forecasts. (DOCX)

S1 Dataset Monthly thunderstorm frequency and meteorological data for Sylhet, Sreemangal, and Mymensingh. (XLSX)
