# Supplementary material for: Station-specific thunderstorm forecasting using climate predictors with seasonal decomposition analysis in Bangladesh’s most vulnerable regions
Source: PLoS One. 2026 Sep 24;21(9):e0358795. doi: 10.1371/journal.pone.0358795 (PMC13607998; doi:10.1371/journal.pone.0358795)
Supplement: Supplementary file 1 [file pone.0358795.s003.pdf]

Review Report

Abstract:

The abstract should clearly summarize the research manuscript's conclusions. It is currently too general. It should specify the research findings with numerical statistics (if any).

Introduction:

1. Which months are the pre-monsoon and monsoon season? It is not mentioned in the introduction.
2. Please improvise on the introduction section. It does not address recent research studies that could be relevant.

Materials and Methods:

1. Figure 1 is not ideal for the journal paper. In the figure, there is no scale bar, coordinate, and the resolution is too low.
2. Revise the section on the study area and data collection. It seems more relevant to the results section rather than the study area and data collection part. For your example, you can see this paper: 'GIS-based spatial analysis for lightning scenario in Bangladesh'.
3. To model and forecast the monthly frequency of TS days in high-risk locations of Bangladesh specifically, Sylhet, Sreemangal, and Mymensingh, this study employs a diverse set of forecasting techniques, encompassing both traditional time series models and modern machine learning approaches – **irrelevant in the methods section**.
4. ARIMA Model – add citation.
5. In the methods section, better write the full form of the heading of all the model names.
6. Evaluation Metrics – use citation.
7. The datasets have not been compared with other datasets, such as the ISS-LIS dataset. Please provide justification for not using satellite-based data.

Results:

1. Data description is not part of the result. Revise it.
2. Why are Sylhet, Sreemangal, and Mymensingh the most TS-prone regions in Bangladesh? Provide further reasons.
3. Why does Sylhet have the most thunderstorms?
4. The result part is not well structured and described. The findings should be described in the results section very clearly.

Discussion:

1. For Bangladesh, why is the pre-monsoon season only April to May, and also the monsoon season (June – August)? As per the climatic season, March – June is Pre-monsoon and June – September is the Monsoon season.

Conclusion:

This manuscript has no conclusion section.

The authors should maintain a smoother manuscript flow of writing and coherence of each section. In addition, the authors have not explained the reasons for TS occurrences in the specific areas. The current level of description is not deep enough. This manuscript has no page and line numbers, making it difficult for a reviewer to review.
