# Supplementary material for: Station-specific thunderstorm forecasting using climate predictors with seasonal decomposition analysis in Bangladesh’s most vulnerable regions
Source: PLoS One. 2026 Sep 24;21(9):e0358795. doi: 10.1371/journal.pone.0358795 (PMC13607998; doi:10.1371/journal.pone.0358795)
Supplement: Supplementary file 2 [file pone.0358795.s004.pdf]

05 September, 2025

Climate Change and Seasonal Thunderstorm Intensification: Evidence from Bangladesh's Most Vulnerable Regions, PONE-D-25-30212

Dear Editor,

We sincerely thank you and the reviewers for your time and thoughtful comments on our manuscript. We appreciate the opportunity to revise our work and have carefully addressed each concern to enhance the scientific quality, clarity, and structure of the manuscript. Below, we provide a detailed, point-by-point response to all the editorial and reviewer comments.

Best regards,

Authors

Editorial Requirements

When submitting your revision, we need you to address these additional requirements.

1. Please ensure that your manuscript meets PLOS ONE's style requirements, including those for file naming. The PLOS ONE style templates can be found at https://journals.plos.org/plosone/s/file?id=wjVg/PLOSOne_formatting_sample_main_body.pdf and https://journals.plos.org/plosone/s/file?id=ba62/PLOSOne_formatting_sample_title_authors_affiliations.pdf

Authors' response:

Thank you for this reminder. We have carefully reviewed the provided PLOS ONE style templates and confirm that our manuscript now fully adheres to all formatting requirements, including file naming conventions, reference style, and manuscript structure.

2. Please note that PLOS One has specific guidelines on code sharing for submissions in which author-generated code underpins the findings in the manuscript. In these cases, we expect all author-generated code to be made available without restrictions upon publication of the work. Please review our guidelines at https://journals.plos.org/plosone/s/materials-and-software-sharing#loc-sharing-code and ensure that your code is shared in a way that follows best practice and facilitates reproducibility and reuse.

Authors' response:

We will share all author-generated code without restrictions in a public GitHub repository upon manuscript acceptance. The repository will be cited in the manuscript, include a detailed README file, and be archived on Zenodo to provide a permanent DOI. We confirm adherence to PLOS ONE's code sharing guidelines.

3. When completing the data availability statement of the submission form, you indicated that you will make your data available on acceptance. We strongly recommend all authors decide on a data sharing plan before acceptance, as the process can be lengthy and hold up publication timelines. Please note that, though access restrictions are acceptable now, your entire data will need to be made freely accessible if your manuscript is accepted for publication. This policy applies to all data except where public deposition would breach compliance with the protocol approved by your research ethics board. If you are unable to adhere to our open data policy, please kindly revise your statement to explain your reasoning and we will seek the editor's input on an exemption. Please be assured that, once you have provided your new statement, the assessment of your exemption will not hold up the peer review process.

Authors' response:
We will upload the fully anonymized dataset as Supporting Information files upon manuscript acceptance. We confirm our adherence to PLOS ONE's open data policy. The Data Availability statement has been updated accordingly.

4. We note that you have indicated that there are restrictions to data sharing for this study. For studies involving human research participant data or other sensitive data, we encourage authors to share de-identified or anonymized data. However, when data cannot be publicly shared for ethical reasons, we allow authors to make their data sets available upon request. For information on unacceptable data access restrictions, please see http://journals.plos.org/plosone/s/data-availability#loc-unacceptable-data-access-restrictions. Before we proceed with your manuscript, please address the following prompts:

a) If there are ethical or legal restrictions on sharing a de-identified data set, please explain them in detail (e.g., data contain potentially identifying or sensitive patient information, data are owned by a third-party organization, etc.) and who has imposed them (e.g., a Research Ethics Committee or Institutional Review Board, etc.). Please also provide contact information for a data access committee, ethics committee, or other institutional body to which data requests may be sent.

b) If there are no restrictions, please upload the minimal anonymized data set necessary to replicate your study findings to a stable, public repository and provide us with the relevant URLs, DOIs, or accession numbers. Please see http://www.bmj.com/content/340/bmj.c181.long for guidelines on how to de-identify and prepare clinical data for publication. For a list of recommended repositories, please see https://journals.plos.org/plosone/s/recommended-repositories. You also have the option of uploading the data as Supporting Information files, but we would recommend depositing data directly to a data repository if possible.
Please update your Data Availability statement in the submission form accordingly.

Authors' response:

We will upload the fully anonymized dataset as Supporting Information files alongside our manuscript. The Data Availability statement has been updated to reflect this.

5. Please amend your list of authors on the manuscript to ensure that each author is linked to an affiliation. Authors' affiliations should reflect the institution where the work was

done (if authors moved subsequently, you can also list the new affiliation stating "current affiliation:…." as necessary).

Authors' response:

Thank you for this reminder. We have carefully reviewed and amended the author list and affiliations to ensure each author is correctly linked to their respective institution(s).

6. We note that Figure 1 in your submission contain [map/satellite] images which may be copyrighted. All PLOS content is published under the Creative Commons Attribution License (CC BY 4.0), which means that the manuscript, images, and Supporting Information files will be freely available online, and any third party is permitted to access, download, copy, distribute, and use these materials in any way, even commercially, with proper attribution.

Authors' response:

We thank the editor for this important note. We confirm that Figure 1 is an original creation by the authors and does not contain any copyrighted material. The map was created by the authors using the open-source software QGIS (version 3.30), utilizing our study data. The figure caption has been updated to include the required attribution: "Created with QGIS (http://qgis.org)." We hereby grant permission for it to be published under the CC BY 4.0 license.

Reviewer # 1 Comments

Abstract:

1. The abstract should clearly summarize the research manuscript's conclusions. It is currently too general. It should specify the research findings with numerical statistics (if any).

Authors' response:

We sincerely thank the reviewer for this valuable and constructive feedback. We agree that the inclusion of specific numerical results is crucial for a strong and informative abstract. We have thoroughly revised the abstract to explicitly state our key findings with the relevant statistical metrics, as requested. The revised abstract now includes the following specific numerical results:

- Total TS occurrences: The cumulative thunderstorm days for each region over the study period (Sylhet: 6,029; Sreemangal: 4,954; Mymensingh: 4,488).
- Model performance improvement: The precise reduction in Mean Squared Error (MSE) achieved by our best model (over 65%, with a concrete example: from 14.17 to 4.82 MSE at Sylhet).
- Key error metrics: The actual values for the Root Mean Squared Error (RMSE: 2.20 to 1.91) and Mean Absolute Scaled Error (MASE: ~0.30) that demonstrate the high predictive accuracy of our top-performing model.

Thank you again for this suggestion, which has greatly improved our manuscript.

Introduction:

1. Which months are the pre-monsoon and monsoon season? It is not mentioned in the introduction.

Authors' response:

We appreciate the reviewer's helpful comment. In the revised manuscript, we have explicitly defined the time periods for both the pre-monsoon and monsoon seasons in Bangladesh.

2. Please improvise on the introduction section. It does not address recent research studies that could be relevant.

<u>Authors' response:</u>

We sincerely thank the reviewer for this critical and constructive feedback. We agree that incorporating recent, relevant studies is essential for grounding our research in the current scientific discourse and clearly establishing its novelty. In response to this comment, we have thoroughly revised the Introduction section to significantly strengthen its foundation with up-to-date and highly relevant literature.

Materials and Methods:

1. Figure 1 is not ideal for the journal paper. In the figure, there is no scale bar, coordinate, and the resolution is too low.

<u>Authors' response:</u>

We agree with the reviewer and have replaced Figure 1 with a new, high-resolution map that now includes a scale bar, geographic coordinates (latitude/longitude), and a north arrow. This revised figure provides the necessary spatial context and meets the required standards for publication.

2. Revise the section on the study area and data collection. It seems more relevant to the results section rather than the study area and data collection part. For your example, you can see this paper: 'GIS-based spatial analysis for lightning scenario in Bangladesh'.

<u>Authors' response:</u>

We thank the reviewer for this insightful suggestion and for providing a helpful reference. Following the structure of the suggested paper, we have revised the manuscript by renaming the section as "Study Area" and have kept it to the Materials and Methods section. We have also carefully edited the content of this section to focus purely on the description of the selected regions and the source and nature of the dataset, removing any preliminary results or interpretations that were prematurely presented.

3. To model and forecast the monthly frequency of TS days in high-risk locations of Bangladesh specifically, Sylhet, Sreemangal, and Mymensingh, this study employs a diverse set of forecasting techniques, encompassing both traditional time series models and modern machine learning approaches – irrelevant in the methods section.

<u>Authors' response:</u>

Thank you for the valuable suggestion. In response, we have revised the "Modeling Framework" section to focus strictly on procedural steps rather than study objectives. We now describe the implementation of forecasting models and seasonal adjustment techniques in a stepwise and technical manner. The updated section avoids general statements of intent and aligns more appropriately with methodological standards expected in the Materials and Methods section.

4. ARIMA Model – add citation.

<u>Authors' response:</u>

Thank you for your valuable suggestion. We have now added appropriate citations to foundational and widely accepted sources on the ARIMA model in the Materials and Methods section.

5. In the methods section, better write the full form of the heading of all the model names.

Authors' response:

Thank you for your helpful suggestion. In response, we have revised the manuscript to include the full form of each model name in the respective section headings to improve clarity and readability.

6. Evaluation Metrics – use citation.

Authors' response:

Thank you for the suggestion. We have now included appropriate citations for the evaluation metrics used in the Materials and Method section.

7. The datasets have not been compared with other datasets, such as the ISS-LIS dataset. Please provide justification for not using satellite-based data.

Authors' response:

Thank you for this valuable comment. We acknowledge the relevance of satellite-based datasets, such as the Lightning Imaging Sensor onboard the International Space Station (ISS-LIS), for studying global lightning and thunderstorm activity. However, we respectfully note that the ISS-LIS dataset provides relatively short-term coverage beginning in 2017 (Blakeslee et al., 2020), which does not align with the scope of our study covering a 40-year period from 1985 to 2024. Additionally, our objective was to model station-level thunderstorm (TS) activity using temporally consistent and locally validated data. Ground-based observations from the Bangladesh Meteorological Department (BMD) ensure high spatial and temporal resolution suitable for such analysis. Furthermore, satellite-based datasets often suffer from spatial aggregation and reduced detection reliability under specific atmospheric conditions in this region (Abdelmajeed & Juszczak, 2024; *Disadvantages of Satellite: An Extensive Examination (Updated)*, n.d.). We have included this justification in the revised manuscript (Study Area and Data Collection) and have added appropriate citations. Future work will explore integrating satellite-derived lightning indices to complement and expand upon the present findings.

Results:

1. Data description is not part of the result. Revise it.

Authors' response:

Thank you for the valuable comment. We have revised the manuscript by renaming the section to "Regional Thunderstorm Frequency and Characteristics" to clearly distinguish the descriptive overview from the core results. The detailed data description, including study area and data source information, has been retained in the Materials and Methods section to maintain appropriate manuscript structure. This ensures the Results section focuses on the analysis and interpretation of thunderstorm patterns, in accordance with standard scientific reporting practices.

2. Why are Sylhet, Sreemangal, and Mymensingh the most TS-prone regions in Bangladesh? Provide further reasons.

<u>Authors' response:</u>

Thank you for the insightful comment. We have addressed this in the revised manuscript by explaining the climatological and topographical factors contributing to high thunderstorm activity in these regions. Specifically, the orographic uplift from the Meghalaya Plateau, sustained moisture influx from the Bay of Bengal, and the convergence of moist easterlies and westerly disturbances create favorable conditions for thunderstorm formation. This is now clearly described in the "Regional Thunderstorm Frequency and Characteristics" section.

3. Why does Sylhet have the most thunderstorms?

<u>Authors' response:</u>

We appreciate the reviewer's request for clarification. The manuscript now includes a detailed explanation that Sylhet's unique physiography being situated in a basin surrounded by Meghalaya and Tripura hills, enhances moisture trapping and orographic forcing. Its proximity to very wet highland areas like Cherrapunji and Mawsynram provides a continuous moisture supply, lengthening the convective season and increasing thunderstorm frequency. These points are incorporated in the revised "Regional Thunderstorm Frequency and Characteristics" section.

4. The result part is not well structured and described. The findings should be described in the results section very clearly.

<u>Authors' response:.</u>

We thank the reviewer for this feedback. We have thoroughly restructured the Results section to improve its clarity and flow.

Discussion:

1. For Bangladesh, why is the pre-monsoon season only April to May, and also the monsoon season (June – August)? As per the climatic season, March – June is Pre- monsoon and June – September is the Monsoon season.

<u>Authors' response:</u>

We thank the reviewer for pointing out the official seasonal definitions used by the Bangladesh Meteorological Department. While climatologically the pre-monsoon is March–May and the monsoon is June–September, our analysis of storm occurrence data from 1985–2024 shows that thunderstorm activity is most concentrated during April–May (pre-monsoon peak) and June–August (monsoon peak). This data-driven conclusion is supported by the monthly distribution patterns shown in Figures 4–6, which consistently highlight these months as having the highest TS activity across all three stations. We have now clarified this rationale in the Discussion section to avoid confusion and emphasize that our seasonal framing is based on observed TS frequency, not general climatological periods.

Conclusion:

1. This manuscript has no conclusion section.

<u>Authors' response:</u>

Thank you for the comment. We have now added a clear Conclusion section highlighting the novelty of our station-level forecasting approach, the effectiveness of seasonal adjustment methods, and the policy relevance of our findings for improving early warning systems and disaster preparedness in Bangladesh.

Overall Structure and Flow:

1. The authors should maintain a smoother manuscript flow of writing and coherence of each section. In addition, the authors have not explained the reasons for TS occurrences in the specific areas. The current level of description is not deep enough. This manuscript has no page and line numbers, making it difficult for a reviewer to review.

<u>Authors' response:</u>

We deeply appreciate the reviewer's insightful and helpful criticism, which has significantly helped us to improve the manuscript's clarity, depth, and overall quality. We have undertaken a comprehensive revision to address each point raised.

We sincerely apologize for this oversight, which undoubtedly complicated the review process. We have now added page number and continuous line numbers throughout the entire revised manuscript to facilitate a much smoother review.

<u>Reviewer # 2 Comments</u>

Introduction:

1. More accurate and updated references needed and are available. I am not commenting on each reference. We should provide more matching references and credit should go to those really contributed to specific knowledge domain. Background information of this paper is much off the way of its main two research objectives. More accurate and updated context and references about thunderstorm characteristics and impacts should be provided.

1. Rahman, Md. M., Shobuj, I. A., Santo, Md. M. H., Ali, H. M., Hossain, Md. T., Alam, E., & Islam, Md. K. (2025). Knowledge, attitude, and practices towards lightning among vulnerable group: A case study on Agri farmers and fishermen. Sustainable Environment, 11(1). https://doi.org/10.1080/27658511.2025.2464373
2. Akter, M.Y., Islam, A.R.M., Mallick, J., Alam, E., 2024. Temperature extremes Projections over Bangladesh from CMIP6 Multi-model Ensemble. Theor Appl Climatol.. https://doi.org/10.1007/s00704-024-05173-5
3. Rahman, M.M., Mannan, M.A., Sarkar, M.S.K., Sultana, A., Islam, M.M., Akter, M.Y., Alam, E., and Islam, A.R.M.T. 2024. Are hotspots and frequencies of heatwave changing over time? Exploring causes of heatwaves in a tropical country. Plos One, 19 (5): e0300070. https://doi.org/10.1371/journal.pone.0300070
4. Islam, A.R.M.T, Hasanuzzaman, M., Jaman, M., Alam, E., Mallick, J., Alam, G.M.M, Sattar,

M.A., Techato, K., 2021. Assessing farmers perceived typologies concerning environmental issues and climate change for sustainable adaptation strategies in Bangladesh, Climate Journal. Xx. Doi: org/10.3390/cli9120167

Authors' response:

Thank you for this critical and constructive feedback. We agree that incorporating recent, relevant studies is essential for grounding our research in the current scientific discourse and clearly establishing its novelty. In response to this comment, we have thoroughly revised the Introduction section to significantly strengthen its foundation with up-to-date and highly relevant literature. The specific improvements we have made include:

- Incorporation of Suggested References: We have integrated the key recent studies suggested by the reviewer (Rahman et al., 2025; Akter et al., 2024; Rahman et al., 2024; Islam et al., 2021) to precisely address the context of vulnerability, climate trends, and adaptation in Bangladesh. These citations now serve to: reframe the problem, update the climate context, and justify the need for better forecasting.
- The revised "Introduction" now clearly articulates the research gap, stating that prior work in Bangladesh often relies on aggregated national data and a critical gap exists in developing high-resolution, localized forecasting models for vulnerable regions.
- The global context has been simplified, making the overview more relevant to the study's methodology.

Hence, the revised Introduction has significantly improved the introduction by incorporating a more relevant set of references, allowing for a more compelling presentation of the research gap and a clearer justification for the study's objectives.

Data used and methodology:

1. Methods are substantial. A step by step wise data collection, data cleaning and analysis variables, and processes may enhance method section.

Authors' response:

We thank the reviewer for this positive feedback and valuable suggestion. We have thoroughly revised the 'Materials and Methods' section to provide a clearer, step-by-step description of our workflow.

Results:

1. The results are clearly presented.

Authors' response:

Thank you for your positive comment on the Results section. In response to Reviewer 1's suggestions, we have further refined this section to improve clarity and structure. We believe these improvements have strengthened the presentation of our findings.

Discussion:

1. This section is substantial.

Authors' response:

Thank you for your positive feedback on the Discussion section. We have endeavored to provide a comprehensive and detailed analysis to thoroughly interpret our findings in the context of existing literature and practical implications. We hope the depth of discussion enhances the overall quality and clarity of the manuscript.

Conclusion:

1. It is not clear from the analysis how their findings are new and novel from current understanding of the relevant science and how they are contributing to improve current state of art in the discipline. I would encourage to write how the policy sciences can be improved from the results presented in the discipline.

Authors' response:

Thank you for the comment. We have now added a clear Conclusion section highlighting the novelty of our station-level forecasting approach, the effectiveness of seasonal adjustment methods, and the policy relevance of our findings for improving early warning systems and disaster preparedness in Bangladesh.
