# Supplementary material for: Station-specific thunderstorm forecasting using climate predictors with seasonal decomposition analysis in Bangladesh’s most vulnerable regions
Source: PLoS One. 2026 Sep 24;21(9):e0358795. doi: 10.1371/journal.pone.0358795 (PMC13607998; doi:10.1371/journal.pone.0358795)
Supplement: Supplementary file 3 [file pone.0358795.s005.pdf]

General Assessment

This manuscript addresses thunderstorm (TS) occurrences in northeastern Bangladesh and applies several statistical and machine learning models to station data. While the topic is relevant, the study suffers from fundamental flaws in design, data, methods, and interpretation. The work is presented with ambitious claims but lacks the scientific rigor and novelty to support them. The problems are structural and cannot be fixed by revision.

Major issues

1. Lack of definition and data transparency
   - The manuscript never defines what constitutes a “thunderstorm occurrence” in the BMD dataset (observer logs, lightning detection, rainfall with thunder, etc.). Without this, the data cannot be meaningfully interpreted or validated.
   - No discussion of reporting consistency across the 40-year period is provided, raising concerns about data inhomogeneity.

2. Misuse of “High-resolution forecasting”
   - The authors frame a “critical gap” in high-resolution localized forecasting. However, their dataset consists of monthly counts from only three stations, which is the opposite of high-resolution both spatially and temporally.
   - The claim that this study addresses that gap is misleading.

3. Weak and misapplied methods
   - Six models and two seasonal decompositions are tested on a very small dataset (~1,440 monthly points). Applying complex ML models (XGBoost, LSTM) to such limited univariate data is inappropriate and risks overfitting.
   - The methods section is bloated with textbook definitions but omits crucial details (data quality control, training/validation strategy, hyperparameter tuning, uncertainty estimation).

4. Results as filler
   - 8 out of 10 figures and large parts of the text are devoted to basic descriptive climatology of three stations (seasonal cycles, boxplots, radar charts). These simply restate well-known patterns (April-June peaks, Sylhet wetter than others) and add no new major insight.
   - The forecasting results (Table 1 and Figures 9-10) are thin, and the main finding (i.e., seasonal decomposition improves model performance) is trivial and well-established.

5. Unsupported climate change attribution
   - The manuscript repeatedly attributes thunderstorm intensification to climate change, yet no formal trend analysis, attribution methods, or diagnostics (e.g., CAPE, vertical wind shear, ENSO/IOD influences) are performed.
   - Claims of “confirmation” are based only on visual inspection of time series, which is not rigorous.

6. Overstated novelty and impact

- The use of STL decomposition with XGBoost is presented as a methodological breakthrough, but in reality this is a routine time-series preprocessing step.
- The authors extrapolate forecasts to 2025-2028 and present them as reliable. With such limited data and simplistic models, these projections are scientifically meaningless.
- Claims that the work informs “early warning systems, climate-resilient infrastructure, and public health” are vastly overstated given the coarse dataset and limited results.

7. Contradictions in discussion

- The authors acknowledge major limitations (monthly aggregation, only three stations, univariate models without covariates), which in fact invalidate their main conclusions.
- Despite these admissions, they still make strong claims about climate change impacts and operational utility, creating a clear disconnect between evidence and conclusions.

8. Conclusion

- The conclusion is awkwardly brief, merely repeating methods and results without genuine synthesis, which reinforces the manuscript’s imbalance and lack of depth.

Summary

This is a classic case of overselling: the manuscript makes ambitious claims about filling critical gaps and supporting climate resilience, but the actual results are limited to descriptive plots and small error reductions in basic forecasting models. The disconnect between claims and evidence, combined with missing definitions, filler content, and inappropriate methodology, makes the manuscript unsuitable for publication.
