# Supplementary material for: Station-specific thunderstorm forecasting using climate predictors with seasonal decomposition analysis in Bangladesh’s most vulnerable regions
Source: PLoS One. 2026 Sep 24;21(9):e0358795. doi: 10.1371/journal.pone.0358795 (PMC13607998; doi:10.1371/journal.pone.0358795)
Supplement: Supplementary file 4 [file pone.0358795.s009.pdf]

17 March 2026

Climate Change and Seasonal Thunderstorm Intensification: Evidence from Bangladesh's Most Vulnerable Regions

Dear Editor,

We sincerely thank you and all the reviewers for your valuable time, insightful comments, and constructive feedback on our revised manuscript. We greatly appreciate the opportunity to further improve our work. We have carefully considered every comment and have made corresponding revisions to enhance the manuscript's scientific quality, clarity, methodological transparency, and overall structure. Based on reviewer feedback, we have revised the title to "**Climate Change and Seasonal Thunderstorm Dynamics: Evidence from Bangladesh's Most Vulnerable Regions**" to more accurately reflect the study's content, which focuses on thunderstorm frequency rather than intensification. The substantial methodological improvements necessitated the inclusion of an additional co-author, Rawfun Nahin Kowbi, who made significant contributions to the revised modeling framework, hyperparameter tuning implementation, and validation procedures. All authors have approved this addition. Below, we provide a detailed, point-by-point response to all editorial and reviewer comments.
Best regards,

Authors

Reviewer #1: Comments

1. The study area is not well described. The study area section should provide a clear description of the geographical, environmental, climatic, and socio-economic characteristics of the study area, supported by relevant maps or figures. So that, reader can get a clear picture and idea about your research region. The authors should improve it and see other published papers for a good understanding.

Authors' response:
We thank the reviewer for this valuable suggestion and have substantially improved the Study Area and Data subsection to provide a clearer picture of our research regions. In the revised manuscript, we now provide:

- **Enhanced geographical description:** Lines 233-243 and Table 1 now describe each station's location, proximity to the Meghalaya Plateau, and orographic setting in detail.
- **Comprehensive Table 1**: A new table summarizing station name, ID, coordinates, and long-term (1985–2024) monthly averages for all meteorological variables (TS frequency, temperature, relative humidity, cloud cover, rainfall, atmospheric pressure). This numerical summary complements the textual description and explicitly highlights that Sylhet has the highest long-term mean thunderstorm activity (12.56 frequency/month), followed by Sreemangal (10.32) and Mymensingh (9.35).
- **Improved Figure 1:** The map now includes a scale bar, coordinates, north arrow, and the three study stations are clearly marked with larger red stars and labeled directly.
- **Socio-economic context:** We now explicitly state as a limitation (Lines 220-225) that consistent station-level socio-economic time series are not available for the full 1985–2024 period. We therefore restrict quantitative analysis to meteorological observations

and discuss socio-economic vulnerability qualitatively using recent literature, with citations to media reports and survey-based studies [86-89].

2. The authors can rename the study area to "study area and data." Give a brief description of your dataset (like stations, data resolutions, temporal resolutions, etc.).

Authors' response:
We agree with this recommendation. Section 2 has been reorganized as follows:

2.1 Study Area and Data
 2.1.1 Description of Study Stations and Dataset
 2.1.2 Justification for Ground-Based Data

This reorganization improves logical flow. Section 2.1.1 now provides complete dataset description including stations (IDs, coordinates), variables (TS frequency, temperature, relative humidity, cloud cover, rainfall, atmospheric pressure), temporal coverage (1985–2024, 480 months), and data resolution (monthly aggregates from 3-hourly observations). Which ensures readers have complete context before encountering the modeling methodology.

3. Lines 161 – 164 are irrelevant in the study area section.

Authors' response:
Lines 161-164 (discussion of satellite data limitations) have been removed from the Study Area section and relocated to **2.1.2 Justification for Ground-Based Data**, a new subsection that follows the dataset description. This placement is more logical, as it explains our data choice immediately after presenting the dataset. The relocated text has also been substantially condensed to focus only on the temporal coverage mismatch (ISS-LIS begins 2017 vs. our 40-year record) and spatial resolution issues, removing extraneous detail.

4. The conclusion does not represent the key findings of this research. I would encourage the authors to improve the conclusion section and explain how the findings would be helpful for the decision makers.

Authors' response:
We have completely rewritten the Conclusion section to:

- Summarize key empirical findings with numerical results: correlation analysis (r values), univariate model performance (RMSE = 2.08–2.53), exogenous model including climate predictors (temperature, relative humidity, cloud cover, rainfall, and atmospheric pressure) improvements (MASE = 0.17–0.22), and over 80% improvement compared to univariate benchmarks.
- Articulate methodological contributions with humility: systematic comparison of 18 modeling approaches across two frameworks – univariate and exogenous – with seasonal decomposition.
- Provide actionable recommendations for decision-makers:
  - BMD can implement validated STL-XGBoost and STL-SVR frameworks for station-specific forecasts

    - Seasonal projections identify April–August as the critical preparedness window for targeted resource allocation
    - Demonstrated value of integrating climatic variables provides scientific basis for investing in meteorological data collection
    - Reproducible pathway to enhance early warning systems, and inform climate-resilient infrastructure planning
  - Acknowledge data limitations and emphasize need for sustained interdisciplinary collaboration to translate forecasting improvements into measurable risk reduction outcomes.

Reviewer #3: Comments

The manuscript appears suitably revised based on the comments on the earlier version. Recommended for publication in the revised form.

Authors' response:
Thank you for your positive feedback and recommendation for publication. We appreciate your time and constructive input on the earlier version.

Reviewer #4: Comments

General Assessment:
This manuscript addresses thunderstorm (TS) occurrences in northeastern Bangladesh and applies several statistical and machine learning models to station data. While the topic is relevant, the study suffers from fundamental flaws in design, data, methods, and interpretation. The work is presented with ambitious claims but lacks the scientific rigor and novelty to support them. The problems are structural and cannot be fixed by revision.

Authors' response:
We thank Reviewer #4 for the thorough critique. We have taken these concerns very seriously and have fundamentally revised the manuscript to address each issue. The substantial methodological improvements—including climate predictors, e.g., temperature, relative humidity, cloud cover, rainfall, and atmospheric pressure, in the exogenous model framework, rigorous trend analysis, comprehensive hyperparameter tuning, expanded model validation, and tempered claims—have resulted in a much stronger manuscript. We respectfully submit that the structural problems have been addressed through the following revisions:

Major Issues:

1. Lack of definition and data transparency

- **The manuscript never defines what constitutes a "thunderstorm occurrence" in the BMD dataset (observer logs, lightning detection, rainfall with thunder, etc.). Without this, the data cannot be meaningfully interpreted or validated.**
- **No discussion of reporting consistency across the 40-year period is provided, raising concerns about data inhomogeneity.**

Authors' response:
We have added a clear definition and data quality and consistency information in Section 2.1:

"Following the BMD Operational Manual [32], a TS occurrence is officially defined as an observation period during which thunder is audibly heard by a certified meteorological observer at the station, regardless of whether accompanying precipitation occurs. TS frequency is defined as the number of thunderstorm occurrences recorded at a given location over a specified temporal scale (e.g., daily, monthly, seasonal, or annual). In the BMD network, thunderstorm observations are made eight times per day at 3-hour intervals following World Meteorological Organization (WMO) standard synoptic hours. All data underwent rigorous quality control by BMD, including consistency checks and temporal continuity verification, with no major station relocations and consistent adherence to WMO standards throughout 1985–2024. The count of thunderstorm reports within a calendar day is treated as the daily TS frequency, and monthly TS frequency for each station is obtained by aggregating the corresponding daily frequencies over the month."

2. Misuse of "High-resolution forecasting"

- **The authors frame a "critical gap" in high-resolution localized forecasting. However, their dataset consists of monthly counts from only three stations, which is the opposite of high-resolution both spatially and temporally.**
- **The claim that this study addresses that gap is misleading.**

Authors' response:
We acknowledge this valid criticism and have revised the language throughout the manuscript. We have:

- Removed the term "high-resolution" from all instances, replacing it with "accurate" or "localized" or "station-specific" forecasting
- Reframed the research gap: "However, a critical gap remains in developing accurate localized forecasting models that account for the unique climatology of Bangladesh's most vulnerable regions."
- Clarified the contribution: "We move beyond aggregated national models to provide station-specific forecasts using a comprehensive suite of eighteen modeling approaches... to capture the unique temporal and spatial dynamics of thunderstorms."

The revised language accurately reflects that our contribution is station-specific modeling rather than high-resolution forecasting.

3. Weak and misapplied methods

- **Six models and two seasonal decompositions are tested on a very small dataset (~1,440 monthly points). Applying complex ML models (XGBoost, LSTM) to such limited univariate data is inappropriate and risks overfitting.**
- **The methods section is bloated with textbook definitions but omits crucial details (data quality control, training/validation strategy, hyperparameter tuning, uncertainty estimation).**

Authors' response:
We have substantially revised the Methods section to address these concerns:

- Acknowledged dataset limitations: We now explicitly discuss that monthly data (480 observations per station) may be insufficient for complex deep learning models, which explains LSTM's poor performance
- Added models (SARIMAX, ETSX, ANN, SVR, XGBoost, LSTM) incorporating climate predictors - temperature, relative humidity, cloud cover, rainfall, and air pressure - to capture the meteorological determinants of TS activity. (Section 2.2.2)

- Added rigorous cross-validation: Section 2.2.4 now describes time-series cross-validation with expanding window (5 folds, 12-month forecast horizon) specifically designed to prevent overfitting while respecting temporal dependencies.
- Comprehensive hyperparameter tuning: We have added detailed hyperparameter tuning information in two new supplementary tables (Table S1 and S2), including search ranges, optimal values, and validation performance (mean RMSE ± std across CV folds). This ensures transparency and demonstrates that overfitting was actively prevented.
- Condensed textbook definitions: We have significantly shortened the theoretical descriptions of each model (now 2-3 sentences per model) and focused on their application to our specific dataset and objectives. The removed text is available in supplementary materials if needed.

4. Results as filler

- **8 out of 10 figures and large parts of the text are devoted to basic descriptive climatology of three stations (seasonal cycles, boxplots, radar charts). These simply restate well-known patterns (April-June peaks, Sylhet wetter than others) and add no new major insight.**
- **The forecasting results (Table 1 and Figures 9-10) are thin, and the main finding (i.e., seasonal decomposition improves model performance) is trivial and well-established.**

Authors' response:
We have re-run all analyses using exogenous models that include climate predictors, and substantially reorganized the Results section to better balance descriptive and analytical content:

- Condensed descriptive climatology: The descriptive material has been streamlined and integrated into Section 3.1. Figures 3-6 have been combined where appropriate (e.g., boxplots for all three stations now in a single multi-panel Figure 3).
- Expanded forecasting results: We have added: Detailed comparison of univariate vs. exogenous model performance (Section 3.2, Figures 5-6), Comprehensive error metrics in Tables 2-3, Hyperparameter tuning validation results in Tables S1-S2, Long-term forecasts (2025–2028) in Figure 7 to demonstrate model behavior over extended horizons
- Contextualized the seasonal decomposition finding: While the value of seasonal decomposition is well-established in time series analysis, its application to thunderstorm forecasting in Bangladesh—particularly the comparison of X-11 vs. STL and the integration with machine learning models—represents a novel contribution. We now emphasize this more clearly.

5. Unsupported climate change attribution

- **The manuscript repeatedly attributes thunderstorm intensification to climate change, yet no formal trend analysis, attribution methods, or diagnostics (e.g., CAPE, vertical wind shear, ENSO/IOD influences) are performed.**
- **Claims of "confirmation" are based only on visual inspection of time series, which is not rigorous.**

Authors' response:
We fully agree and have completely revised the climate change framing:

- Added formal trend analysis: Table 1 now includes Mann-Kendall test results, Sen's slope estimates, 95% confidence intervals, and p-values for each station. This rigorous statistical analysis reveals a significant decreasing trend only at Sylhet (1.75 days per decade), with no significant trends at Sreemangal and Mymensingh.
- Tempered claims: We have removed all unsupported attributions. The revised text (Lines 180-195) now states: "These findings demonstrate that claims of systematic thunderstorm intensification in Bangladesh are not supported by station-level occurrence data from these three locations. Only Sylhet shows a detectable change, and that change is a decline, not an increase."
- Acknowledged limitations: We explicitly note that we do not perform formal attribution to climate change, as this would require additional diagnostics (CAPE, vertical wind shear, large-scale climate drivers) beyond the scope of this study.
- Reframed the discussion: The Discussion now appropriately contextualizes results within climate change literature without making unsupported claims.

6. Overstated novelty and impact

- **The use of STL decomposition with XGBoost is presented as a methodological breakthrough, but in reality, this is a routine time-series preprocessing step.**
- **The authors extrapolate forecasts to 2025-2028 and present them as reliable. With such limited data and simplistic models, these projections are scientifically meaningless.**
- **Claims that the work informs "early warning systems, climate-resilient infrastructure, and public health" are vastly overstated given the coarse dataset and limited results.**

Authors' response:

We have substantially tempered our claims throughout the manuscript:

- Reframed methodological contribution: We now present STL+XGBoost as a "validated framework" rather than a "breakthrough." The contribution is positioned as the systematic comparison of 18 approaches for station-specific forecasting in Bangladesh—a context where such comprehensive evaluation was previously lacking.
- Contextualized forecasts: We now clearly state (Lines 658-660) that "However, this forecast skill degrades rapidly with increasing lead time, and these projections are presented as illustrations of model behavior rather than reliable long-term predictions." The forecasts are framed as demonstrating model capability rather than providing definitive future estimates.
- Qualified practical implications: We have carefully qualified statements about operational utility. For example (Lines 808-811): "Monthly frequency forecasts cannot directly support the imminent threat warnings—minutes to hours—that are most critical for life-saving actions during individual TS events. However, monthly outlooks serve a complementary function: they inform preparedness planning at weekly to monthly lead times."

7. Contradictions in discussion

- **The authors acknowledge major limitations (monthly aggregation, only three stations, univariate models without covariates), which in fact invalidate their main conclusions.**

- **Despite these admissions, they still make strong claims about climate change impacts and operational utility, creating a clear disconnect between evidence and conclusions.**

Authors' response:
We have carefully revised the Discussion to ensure alignment between acknowledged limitations and conclusions:

- Strengthened limitation discussion: Section 4 (Lines 700-750) now provides a comprehensive and upfront discussion of limitations, including: Monthly data cannot support minute-to-hour warnings, Only three stations limit spatial generalizability, and Lack of socio-economic data prevents impact assessment.
- Removed disconnect: All strong claims about climate change impacts have been removed. Operational recommendations are now framed as potential pathways that require further validation and institutional collaboration.

8. Conclusion

- **The conclusion is awkwardly brief, merely repeating methods and results without genuine synthesis, which reinforces the manuscript's imbalance and lack of depth.**

Authors' response:
We have entirely revised the Conclusion section. Now the Conclusion section presents findings modestly and acknowledges that realizing operational benefits requires sustained interdisciplinary collaboration and addressing data limitations.

<u>**Summary**</u>
This is a classic case of overselling: the manuscript makes ambitious claims about filling critical gaps and supporting climate resilience, but the actual results are limited to descriptive plots and small error reductions in basic forecasting models. The disconnect between claims and evidence, combined with missing definitions, filler content, and inappropriate methodology, makes the manuscript unsuitable for publication.

Authors' response:
We thank Reviewer #4 for this candid assessment. We have fundamentally revised the manuscript to address each issue:

- Overselling and disconnect: We have systematically tempered all claims. Formal trend analysis (Mann-Kendall test, Table 1) now shows no significant intensification at Sreemangal and Mymensingh, and a significant decline at Sylhet—contradicting earlier assumptions. We now frame our contribution modestly as "station-specific forecasting" rather than gap-filling. Operational relevance is qualified: monthly forecasts inform preparedness planning but cannot support minute-to-hour warnings. Forecasts are presented as illustrations of model behavior, not reliable long-term predictions.

- Missing definitions: We have added clear definitions of thunderstorm occurrence in Section 2, data quality procedures, and handling of missing values.

- Filler content: Descriptive climatology has been condensed (Section 3.1), while forecasting content is expanded with comprehensive error metrics (Tables 2-3), hyperparameter tuning results (Tables S1-S2), and station-specific season-wise and long-term forecasts for univariate and exogenous models (Figures 5-7).

- Inappropriate methodology: We have added eighteen modeling approaches across two frameworks—univariate (SARIMA, ETS, SVR, ANN, XGBoost, LSTM) and exogenous (SARIMAX, ETSX, SVR, ANN, XGBoost, LSTM) with X-11 and STL decompositions—with rigorous time-series cross-validation (expanding window, 5 folds), comprehensive hyperparameter tuning, and explicit acknowledgment of limitations (e.g., LSTM underperformance due to sample size).

The revised manuscript now aligns claims with evidence, provides complete transparency, and employs appropriate methodology. We hope the reviewer will find it suitable for reconsideration.

Reviewer #5: Comments

In this manuscript, the authors investigated the yearly and monthly variation in the frequency of thunderstorms (TS) in 3 cities of Bangladesh. They also developed 6 statistical models for forecasting monthly TS frequency during 2025-2028. The result found that the model with seasonally adjusted data obtained the best performance. Unfortunately, the manuscript contains several vital issues, especially the lack of explanation about how the models are trained. A lot of analyses are redundant and not well linked to each other. As a result, the manuscript in the current form cannot be published unless the quality of the study and manuscript is significantly improved, which should include a redesign of the study to make the focus (TS forecasting) clearer.

The Major Comments:

1. As explained in the introduction, the research gap is "high-resolution, localized forecasting models that account for the unique climatology of Bangladesh's most vulnerable regions." However, only 2 of the 10 figures plus one table are related to the forecasting models. The relevant content is disproportionately insufficient. Moreover, the remaining 8 figures do not have any supporting role to pave the way for the development of the model. The analyses associated with Figs. 1-8 are the background information. If the authors insist on the same research gap, the manuscript should be reorganized to emphasize on the building, training, and evaluation of the models, and minimize irrelevant contents (including the figures and analyses).

Authors' response:
We have substantially revised and reorganized the Results section to emphasize forecasting. Descriptive climatology is now condensed into Section 3.1 (Figures 3-6 combined into one multi-panel new Figure 3), while forecasting content is expanded with:

- Detailed univariate vs. exogenous model comparison (Sections 3.1-3.2, Tables 2-3)
- Hyperparameter tuning validation (Tables S1-S2)
- Monthly and seasonal forecasts using univariate and exogenous models (new Figures 5-7).

2. Since the objective is to develop models for better forecasting of TS, the introduction does not contain relevant background information related to the topic. The first 4 paragraphs are simply generic information about TS. The authors should introduce the challenges of forecasting of monthly TS frequency in Bangladesh. The literature review would be strengthened by including a discussion of highly relevant prior work, such as

"Development of novel hybrid machine learning models for monthly thunderstorm frequency prediction over Bangladesh" (https://link.springer.com/article/10.1007/s11069-021-04722-9). This study also develops machine learning models for thunderstorm forecasting in Bangladesh and should be addressed.

Authors' response:
We have revised the introduction section and added discussion of forecasting challenges, incorporated Azd et al. (2021) [30] and other relevant prior work. For instance, "Additionally, hybrid approaches like the ensemble empirical mode decomposition (EEMD)-SVM model [30] and statistical methods like SARIMA [31] have been applied to forecast TS frequency, often using aggregated national data."

Which clearly distinguished our contribution from previous studies, for example Azd et al. (2021) [30] (https://link.springer.com/article/10.1007/s11069-021-04722-9).

3. The authors spend a lot of time on explaining the annual cycle of the number of thunderstorm (Figs. 3-8). However, it is neither new or hidden knowledge. This information can be found in https://www.banglajol.info/index.php/BJPhy/article/view/78684, which is published in 2022. Only very little can be regarded as new information. The redundancy is excessive even if removing the seasonal variable from the training data of models is a key aspect of this study.

Authors' response:
Thank you for your constructive suggestion. Descriptive climatology now has been significantly condensed (Section 3.1). The focus is now on how seasonal patterns inform model development (e.g., need for STL decomposition) rather than repeating established knowledge.

4. For machine learning models, it is important to describe how these models are trained. It should include the split of training, validation, and test dataset, hyperparameters, etc. The training and evaluation of ML models have profound impact of the performance of the models. However, these information are entirely missing in the manuscript. The authors must clearly describe the training and evaluation procedure of each model. Because the structure, setting, and training data allocation of the machine learning and deep learning model are entirely unknown, it is impossible to know whether these models are well trained or overfitted. An LSTM is a powerful algorithm, but its performance is highly dependent on its architecture and training. Without a well-documented design and sufficient training, it is possible for it to underperform simpler models. Therefore, it is impossible to give a fair and meaningful model evaluation.

Authors' response:
We have revised the entire analysis and added the following in Section 2.2.4:

- Data partitioning: 80% training (1985-2016), 20% test (2017-2024)
- Time-series cross-validation with expanding window (5 folds, 12-month horizon)
- Comprehensive hyperparameter tuning with grid search (Tables S1-S2)

5. The description and interpretation of all analyses and figures are insufficient. For example, the interpretation of Fig. 2 focuses on the earlier part of the time series while

the more recent period is ignored. The authors should provide a complete analysis of the entire period, especially given the apparent decreasing trend in recent years, or provide a rationale for its exclusion. There is also no comparison between the results of the 3 cities. Since the 3 cities are neighboring cities, it is possible to analyze the average of them, instead of analyze them individually, if there is no strong reason for doing so.

Authors' response:
We have revised Figure 2 to include five additional climate variables and expanded its interpretation to cover the entire study period, including post-2010 trends. City-level comparisons are now explicitly discussed, with individual station analyses preferred over averaging to preserve location-specific patterns.

6. The author should explain why the seasonally adjusted data lead to better model performance.

Authors' response:
Added explanation in Section 3.2 (Lines 500-510): STL removes dominant seasonal cycle, allowing models to focus on underlying patterns and relationships with climatic variables, reducing noise and improving forecast accuracy.

7. The title does not align with the content. The study is clearly not examining climate change, although there are a few yearly time series; TS does not show intensification trend as well. The authors should revise the title to accurately reflect the content and results.

Authors' response:
We have revised the Title to: "Climate Change and Seasonal Thunderstorm Dynamics: Evidence from Bangladesh's Most Vulnerable Regions". Formal trend analysis (Table 1) now shows no significant intensification at two stations and a decline at Sylhet.

8. Figure 2: The figure is inappropriate. Quantity with different units should not share the same y-axis. The variation in relative humidity and average temperature cannot be observed due to the overly large maximum value in y. Please include only one variable in one panel.

Authors' response:
Figure 2 redesigned as multi-panel plot with separate y-axes per variable, improving clarity.

9. Figure 2: In Sylhet, there is an observable decreasing trend since 2010. For Sreemangal, the decreasing trend is weaker than Sylhet, but the one for Mymensingh is a little bit clearer. These are important findings in the figure, but the authors omit them.

Authors' response:
Thank you for such an important suggestion. Post-2010 trends now explicitly discussed and formally analyzed with Mann-Kendall tests (Table 1).

10. L355: The decreasing trend in TS frequency is obvious in the 3 cities. The authors must clearly explain how they can attribute climate change to such a decreasing trend.

11. L380-381: This statement is a purely qualitative observation. The authors need to connect it to the decreasing trend of the number of TS observed in Fig. 2. I suggest the authors use a t-test to confirm whether a trend is statistically significant of not.

Authors' response:
We have: Added formal Mann-Kendall trend tests with Sen's slope (Table 1); Removed unsupported climate attribution claims; and Acknowledged that formal attribution requires additional diagnostics beyond scope.

12. The function of boxplots in Figs. 4-6 overlap with Fig. 2. To reduce information redundancy, the authors should keep either boxplots of line plots. Also, the authors should also describe the difference in result between the 3 cities. Moreover, Figs 4-6 should be merged into one figure.

13. Figs 4-6: Similar to Fig 2, the monthly TS count in the 3 cities show a clear decreasing trend in the recent decade.

Authors' response:
Figures 3-6 combined into a single multi-panel Figure 3. Boxplots retained as they show distribution (median, spread, outliers) while line plots show temporal evolution—complementary information.

14. L513-518: The authors should explain why there is a discrepancy in the monsoon season definition between your result and the conventional climatological framework. Does the conventional one represent better at national level rather than city level?

Authors' response:
We have added explanation: Our station-level data show peak activity concentrated in April-August, offering local-scale operational insight. The conventional definition (June-September) remains valid at national scale.

<u>Minor comments:</u>

15. L69-70: "Damage" should not be used for "human health". "Leading to …….and risk to human health" is more appropriate. Moreover, it does not seem there is a direct relationship between thunderstorms and human health. Please add references as evidence for such a relationship.

Authors' response:
Thank you for this thoughtful suggestion. We have revised to "leading to substantial damage to infrastructure, agriculture, and risks to human health". Added references [1-3] documenting health impacts.

16. The first 2 paragraphs have common topic: damage. They should be combined into one.

Authors' response:
Paragraphs 1-2 combined into a single paragraph.

17. L78: Remove the word "tragic".

Authors' response:
Removed.

18. L110-111: This paragraph is all about forecasting. However, the studies of the 2 references (28, 29) only used WRF for studying thunderstorms, but not forecasting them. These 2 references are not suitable for this paragraph. Please remove them.

Authors' response:
We thank the reviewer for this clarification. The mention of the WRF model in the Introduction (Lines 123-125) serves only to acknowledge previous studies that have used numerical weather prediction models for thunderstorm simulation in Bangladesh [28,29]. It is not part of our methodology. To avoid confusion, we have revised the sentence to clearly indicate that this refers to prior work, not the current study: "Numerical models, such as the Advanced Research Weather Research and Forecasting (ARW-WRF) model, have been used in previous studies to simulate storms in Bangladesh [28,29]."

19. L122: What is the forecast lead time?

Authors' response:
Lead time specified in Section 3.3, monthly forecasts for 2025-2028 (48-month horizon).

20. L140: This subsection is actually explaining your dataset, and is irrelevant to the study domain. Please describe why you select the 3 cities and exclude the others in more details to justify your choice. Also, write a subsection specifically for describing your dataset.

Authors' response:
We have added justification: stations chosen due to historically elevated TS frequencies [19,20,33] and location within highly active convective zone. Dataset description now in Section 2.1.1.

21. It is difficult to distinguish the level of subsection as the number for indicating sub-section is missing. Please assign subsection number.

Authors' response:
All subsections now numbered (e.g., 2.1.1, 2.1.2).

22. Figure 1: The font of city names are too small to be read easily. Please enlarge them.

Authors' response:
Figure 1 regenerated with a bit larger fonts for city names (see revised Figure 1).

23. L147-148: Please describe how TS events are counted in this dataset.

Authors' response:
We have added clear definition in Section 2.1, eight daily observations at 3-hour intervals following WMO standards; monthly frequency obtained by aggregating daily counts.

24. L152-164: This paragraph will be clearer if you first explain why you use the ground-based dataset, and then why you don't use the satellite dataset.

Authors' response:
Reorganized as suggested: first explain why ground-based data used (Section 2.1.1), then why satellite not used (Section 2.1.2).
25. L163-164: This sentence is unnecessary and inappropriate for this section. Please remove.

Authors' response:
Removed.

26. L184-185: Machine learning approach is statistical in nature, so it is correct to frame all these 6 techniques as statistical methods.

Authors' response:
Revised to Section 2.2.1 Univariate Models and 2.2.2 Models Incorporating Climate Predictors to address that.

27. L188: Abbreviation has been defined earlier on L176. It is unnecessary to write the term in full. The same issue exists for the subsection of all models. Please correct it.

Authors' response:
All abbreviations now defined only at first use and used consistently thereafter.

28. L219-220: The sentence "Click or tap here to enter text" appears too many times. The authors should make sure unnecessary text are removed before submission.

Authors' response:
Removed.

29. L276-298: I suggest the authors include figures to demonstrate a few time series before and after processed by the two decomposition method. It is the best way to show the readers the difference between them, and how they work.

Authors' response:
We have added Figure 7 to display the time series for each station. In Figure 3, the green line is the raw time series, while the red line is the STL-adjusted series.

30. L327-329: These are information for study area. Please move it to Section 2.

Authors' response:
Thank you. Moved to Section 2.1.1 as recommended.

31. L337-338: Although you cited a reference, it is necessary to explicitly explain why large rainfall amount can help severe storm development. It does not sound like a proper causal relationship.

Authors' response:

Added explanation: heavy rainfall indicates deep convective systems with strong updrafts, ice particle interactions, and charge separation—all conducive to thunderstorm development.

32. L356: Figure 2 only shows annual value; seasonal dynamics cannot be observed. Please correct this sentence.

Authors' response:
Corrected: now refers to annual values only.

33. Figure 3: The x-axis should be month instead of season.

Authors' response:
Corrected to month names (Jan-Dec) in revised Figure 3.

34. L406: How is the trend calculated? By moving average? If yes, it is more appropriate to call it "X-year moving average" because "trend" is more specific, usually linear.

Authors' response:

35. Figure 8: Ridgeline density plot is not suitable when data contains very small number of samples (those winter months). Also, TS count is always positive, so the part in the negative region is non-physical. The authors must trim it to avoid misleading the readers.

Authors' response:
Removed.

36. Figs. 8 & 10: What is the value associated with each radius in the plot? On the other hand, the information of this plot is the same as Fig. 3 after taking the average of all years. It is easier for the readers to extract information from a line plot or bar chart than a radar plot.

Authors' response:
Removed.

37. Table 1: Besides showing a table, I suggest the authors to visualize the model performance using bar chart. It can make the comparison of each model easier.

Authors' response:
Thank you for your thoughtful suggestion to visualize model performance using bar charts. We agree that this could enhance comparability across models.

However, we prefer to retain the tabular format for several reasons. Our manuscript already contains seven figures, and adding another would create imbalance. Additionally, tables allow precise reporting of all four evaluation metrics (MSE, RMSE, MAE, MASE) across eighteen models and three stations—information that would be difficult to convey clearly in a bar chart. The current table provides comprehensive numerical detail for readers and future researchers.

We appreciate your understanding.

38. L447: What is the data period for model evaluation?

Authors' response:
We clarified it in Section 2.2.4. The evaluation period is January 2017 to December 2024.

39. L450: ANN is considered as deep learning as long as the network contains at least one hidden layer. Please revise.

Authors' response:
Corrected: ANN now classified as machine learning, with note that deep learning refers specifically to multi-hidden-layer architectures.

40. L483: What are the input variables (including lag time) and forecast lead time of the output of the model? These are important information.

Authors' response:
Added detailed input specifications in Section 2.2.2, Section 2.2.4, and Tables S1-S2, including lag structures and exogenous variables.

41. L495-509: This paragraph describes the background of this study, so it should belong to the Introduction section.

Authors' response:
Moved to Introduction as recommended.

We thank the reviewer for this comprehensive feedback that has helped us substantially strengthen the manuscript.

Reviewer #6: Comments

I have read the revision and compared it with its original version. I think the comments from the two reviewers have well pointed out the problems of the manuscript. However, although the authors claimed that all the comments have been well addressed in the revision, I found the distance between the comments and their edits is still evident. For example, the revised abstract is still not under a typical and academic structure. I suggest that the authors should re-consider these comments very carefully and make their revision with more patience and more times.

Authors' response:
We thank Reviewer #6 for this insightful assessment. We have taken this feedback very seriously and have thoroughly re-reviewed all comments and corresponding revisions. We have now:

- Completely restructured the abstract to follow a standard academic structure: purpose → methodology → key results → conclusions/implications.
- Systematically addressed each comment with a point-by-point response document and corresponding manuscript revisions.
- Added extensive methodological detail on data quality, cross-validation, hyperparameter tuning, and model validation to ensure transparency.

- Tempered all claims to align with evidence and acknowledged limitations.
- Improved figure clarity with more informative captions and interpretation guidance.

We believe these revisions have substantially closed the gap between comments and edits.

Reviewer #7: Comments

The manuscript investigates how climate change has influenced the frequency and intensity of thunderstorms in Bangladesh, with a focus on vulnerable regions. Also, the study seeks both to describe long-term trends and to develop accurate forecasting models that can support disaster preparedness and climate resilience. Overall, the manuscript presents relevant findings with methodological rigor, but it still requires improvements in clarity, methodological transparency, and practical framing to fully demonstrate its scientific and operational value.

1. Abstract:

The abstract starts by describing the dataset and then quickly moves to present the forecasting results. While it does provide numerical findings and highlights the superiority of the STL-XGBoost model, it does not clearly state the objective and scope of the study before diving into outcomes. A stronger abstract would first frame the research purpose, then the methodological approach and the forecasting results.

Authors' response:
We thank the reviewer for this valuable suggestion. The abstract has been completely restructured to follow a clear, logical flow.

2. Introduction:

The revised Introduction successfully incorporates the reviewers' earlier suggestions. The authors clarified the seasonal definitions, added more recent and relevant references, and justified their focus on the northeastern regions of Bangladesh. These changes make the section more coherent and better grounded in current literature. Overall, the Introduction now establishes the research gap more clearly, though it could still be streamlined to reduce redundancy and emphasize the study's novelty more directly.

Authors' response:
We appreciate the reviewer's positive assessment. In response to the suggestion for further streamlining, we have:

- Reduced redundancy: Condensed overlapping discussions of thunderstorm impacts and climate change linkages, while retaining all essential information and citations.

- Emphasized novelty more directly: Added explicit statements highlighting the study's three key innovations (Lines 150-158):

- Strengthened research objectives: Clearly stated three research objectives that directly flow from the identified research gap.

The Introduction now more effectively establishes the research gap and articulates the study's novel contributions while maintaining a coherent, well-grounded narrative.

3. Methods:

The revised Methods section is clearer, with added details on data sources, model descriptions, and proper citations. The authors also improved Figure 1 by including a scale bar, coordinates, and a north arrow. However, the figure still does not adequately highlight the three study areas, which should be more clearly marked to better illustrate the research scope.

Also, this section would benefit from more information on data quality and consistency across the forty-year period. It is unclear whether the dataset contained missing values, how such gaps (if any) were handled, or whether there were changes in measurement methods or station relocations between 1985 and 2024.

One important omission in the Methods section is the lack of detail about the training, validation, and testing strategy for the forecasting models. The manuscript explains which models were applied and how seasonal adjustment was used, but it does not specify how the dataset was divided into training and testing periods, or whether cross-validation was employed. Without this information, it is difficult to assess the robustness and generalizability of the results. Explicitly stating the temporal ranges used for training, validation, and forecasting would strengthen the methodological transparency and reproducibility of the study.

In addition, the description of the forecasting models does not mention any hyperparameter search or tuning process. For complex models such as SVR, ANN, XGBoost, and LSTM, predictive performance is highly sensitive to parameter settings. Without clarification of how hyperparameters were selected—whether through grid search, cross-validation, or another optimization strategy—it is difficult to judge the robustness of the reported results.

Authors' response:
We thank the reviewer for these detailed and constructive comments. We have addressed each point as follows:

- Figure 1 Improvement: We have revised Figure 1 to more clearly highlight the three study areas.
- Data Quality and Consistency: We have added a new paragraph in Section 2.1.addressing data quality.
- Training, Validation, and Testing Strategy: We have substantially expanded Section 2.2.4 to provide complete transparency: "Monthly TS frequency data from January 1985 to December 2024 were partitioned into training (80%) and test (20%) sets. Data from January 1985 to December 2016 (384 observations) were used for model development, hyperparameter tuning, and cross-validation. The remaining period from January 2017 to December 2024 (96 observations) was reserved exclusively as an unseen test set for final performance assessment, ensuring that all reported accuracy metrics reflect genuine out-of-sample predictive capability [70,71].

Given the temporal nature of the data, standard k-fold cross-validation is inappropriate as it ignores the sequential dependence structure [72]. We therefore implemented time-series cross-validation with an expanding window and rolling forecasting origin [73,74]. For each iteration, models were trained on all available data up to a given cut-off point and then used to forecast subsequent steps ahead (1, 3, 6, and 12 months) to evaluate both short-term and multi-step forecasting capability [75]. The forecast errors from each iteration were recorded, and the average multi-step forecast error across all windows served as the primary criterion for hyperparameter tuning and model selection [76]."

- Hyperparameter Tuning: We have added comprehensive hyperparameter tuning details in Section 2.2.4 and included two supplementary tables Tables S1 and S2.

- Optimal values identified through cross-validation: Validation performance (mean RMSE ± std) for each model configuration

These additions ensure readers can fully assess the robustness of our modeling approach and reproduce our results.

4. Results:

The revised Results section is better structured and now separates descriptive information from analytical findings. The authors also explain more clearly why Sylhet, Sreemangal, and Mymensingh are the most thunderstorm-prone regions, linking them to topography and moisture inflows. Figures and tables are informative, but some captions remain overly descriptive. In particular, for less common visualizations, it would be useful to add more guidance on how to interpret the results.

Figures 4–6: boxplots of monthly variation; the colors used are not explained and there is no color bar, leaving the reader uncertain about their meaning.

Figure 7: ridgeline density plot; this less familiar type of visualization would benefit from guidance on how to interpret the curves and the color gradients; additionally, the color bar is missing a title that explains what it represents.

Figures 8 and 10: radar plots; the graphics lack detail on what each plotted point represents; the captions should clarify whether points correspond to normalized averages, monthly frequencies, or forecasted values.

Authors' response:
We thank the reviewer for these helpful suggestions. We have revised all figure captions to be more informative and self-explanatory:

- Figures 3–6 now combined into Figure 3 with the proper caption and explanation.
- Figure 7 (ridgeline density plot): This figure is not required for our current analysis, hence, Figure 7 has been deleted. The new Figure 7 contains "Long-term historical and forecasted monthly TS frequency trend for Sylhet, Sreemangal, and Mymensingh (1985–2028) using univariate and exogenous models frameworks."

- Figures 8 and 10 (radar plots): We have deleted these figures, as they are not required for our revised result and analysis.

These revisions ensure all figures are self-contained and interpretable without reference to the main text.

5. Discussions:

The revised section is more comprehensive and better integrates the findings with existing literature. In the Discussion, it would also be valuable to explain how the proposed forecasting framework could be integrated into operational practice. For instance, the STL-XGBoost model could be embedded within the Bangladesh Meteorological Department's workflow to provide monthly or seasonal outlooks of thunderstorm activity.

Authors' response:
We thank the reviewer for this excellent suggestion. We have added a new paragraph in the Discussion section explicitly addressing suggested operational integration. This addition directly addresses the reviewer's request and strengthens the practical relevance of our work.

Reviewer #8: Comments

1. Overall Significance

- **The manuscript addresses an important and timely topic: the intensification of thunderstorms (TS) in Bangladesh under climate change, which has major societal and economic implications.**
- **The use of station-level forecasting with seasonal adjustment and machine learning is innovative compared to many prior works relying on pooled or regional averages.**
- **However, the paper would benefit from clearer articulation of the *novelty*: is the innovation primarily methodological (STL-XGBoost hybrid), geographic focus (Bangladesh), or policy relevance (early warning systems)?**

Authors' response:
We thank the reviewer for this insightful comment. We have clarified the novelty of our study in the Introduction by articulating three distinct contributions: "A key innovation of our approach is the integration of five exogenous climatic variables—temperature, relative humidity, cloud cover, rainfall, and atmospheric pressure—as predictors alongside historical TS data, enabling us to capture the meteorological drivers of thunderstorm activity. We further evaluate two modeling frameworks: univariate models (using only TS history) and exogenous models (incorporating climate predictors), allowing systematic quantification of the value added by meteorological information. Additionally, we rigorously evaluate seasonal adjustment techniques—specifically X-11 and STL—to isolate and model the strong seasonal patterns inherent in TS data, a step often neglected in conventional forecasting methodologies."

We now explicitly frame the novelty as threefold:

- Methodological: Systematic comparison of 18 modeling approaches across two frameworks (univariate and exogenous) with two seasonal decomposition methods (X-11 and STL) – the first such comprehensive evaluation for thunderstorm forecasting in Bangladesh.

- Geographic: Station-specific forecasting for the three most thunderstorm-prone regions in northeastern Bangladesh, moving beyond aggregated national models that mask local patterns.
- Policy-relevant: Providing the Bangladesh Meteorological Department (BMD) with a validated, operational framework for station-specific forecasts, with seasonal projections identifying April–August as the critical preparedness window for targeted disaster management.

This clearer articulation helps readers understand the multi-faceted contribution of our work.

2. Abstract and Introduction

- **The abstract is well-structured but somewhat dense. Consider simplifying technical details (e.g., naming fewer models in the abstract) and emphasizing the key policy relevance more strongly.**
- **The introduction reviews global and local literature but could expand discussion of:**
    - **Why thunderstorms specifically (vs. cyclones/floods) have been understudied in Bangladesh.**
    - **The unique socio-economic vulnerability of Sylhet, Sreemangal, and Mymensingh beyond climatology (e.g., agriculture dependence, lightning mortality).**
    - **How this study directly advances or contrasts with prior works using SARIMA/ARIMA in Bangladesh**

Authors' response:
Thank you for this insightful suggestion. We have revised as follows:

- Abstract: We have revised the entire abstract to reduce technical density while strengthening policy relevance.

- Introduction: We have expanded the Introduction to address all these points.

3. Methods

- **The dataset description (1985–2024, BMD station data) is clear. Still:**
    - **Clarify data quality control: Were missing values imputed? Were homogenization checks performed?**
    - **Explain whether lightning-day definitions or storm thresholds were consistent over the 40 years.**
- **The seasonal adjustment section is strong (X-11 vs STL), but more justification of why STL was expected to perform better would help (currently explained mostly post-results).**
- **The model comparison:**
    - **Six forecasting models are compared (ARIMA, ETS, ANN, SVR, XG-Boost, LSTM). However, LSTM performed very poorly (MSE > 100). Why? Was hyperparameter tuning sufficient? Could this be due to data scarcity?**
    - **Were cross-validation or out-of-sample tests done, or only in-sample error reporting? Clarify.**

- **Report computational efficiency – were some models more resource-intensive, which could matter for operational use by BMD?**

Authors' response:
Thank you for this good suggestion. We have revised

- Data Quality Control: We have added a new paragraph in Section 2.1 to address this issue.
- STL vs. X-11 Justification: We have added a priori justification in Section 2.2.3.
- LSTM Performance and Data Scarcity: We have added explanation in Section 3.2 and the Discussion: "LSTM models exhibited poor performance on raw data (MASE > 1.40 across all stations), despite comprehensive hyperparameter tuning (Table S2). This underperformance is attributable to the limited sample size (480 monthly observations per station), which is insufficient for deep learning architectures to effectively learn complex temporal dependencies. LSTM typically requires thousands of observations to outperform simpler methods [59,62]. However, seasonal adjustment via X-11 markedly improved LSTM accuracy (e.g., Sylhet MASE reduced from 1.58 to 0.45), demonstrating that even complex architectures benefit from prior removal of seasonal structure when data are limited."
- Cross-Validation and Out-of-Sample Testing: We have substantially expanded Section 2.2.4 to provide complete transparency: "Monthly TS frequency data from January 1985 to December 2024 were partitioned into training (80%) and test (20%) sets. Data from January 1985 to December 2016 (384 observations) were used for model development, hyperparameter tuning, and cross-validation. The remaining period from January 2017 to December 2024 (96 observations) was reserved exclusively as an unseen test set for final performance assessment, ensuring that all reported accuracy metrics reflect genuine out-of-sample predictive capability [70,71].
  Given the temporal nature of the data, standard k-fold cross-validation is inappropriate as it ignores the sequential dependence structure [72]. We therefore implemented time-series cross-validation with an expanding window and rolling forecasting origin [73,74]. For each iteration, models were trained on all available data up to a given cut-off point and then used to forecast subsequent steps ahead (1, 3, 6, and 12 months) to evaluate both short-term and multi-step forecasting capability [75]. The forecast errors from each iteration were recorded, and the average multi-step forecast error across all windows served as the primary criterion for hyperparameter tuning and model selection [76]."

- Computational Efficiency: We thank the reviewer for raising the important point of computational efficiency. While our revised analysis—comparing 18 models across univariate and exogenous frameworks with X-11 and STL decomposition—is already extensive, we recognize that operational deployment requires efficient algorithms. The optimal models identified (STL-XGBoost for Sylhet; STL-SVR for Sreemangal and Mymensingh) are computationally lightweight, requiring only a few seconds to execute on a standard desktop computer for the monthly dataset. This makes them well-suited for routine implementation by BMD. We have added this information in Section Discussion and also noted that comprehensive benchmarking of computational trade-offs across all models, while beyond the current scope, would be a valuable direction for future work.

4. Results

- **Results are clearly presented with detailed tables of MSE, RMSE, and MAE across stations.**
- **STL-XG-Boost consistently outperforms, but the margins of improvement over STL-SVR or STL-ETS could be better highlighted numerically (e.g., % gain).**
- **Figures:**
    - **Figure 2 (stacked bars of temperature, humidity, and TS counts) is visually overloaded. Consider splitting variables or using line + bar overlays for clarity.**
    - **Forecast plots (2025–2028, Figure 9) are strong but could include confidence intervals to communicate uncertainty.**
    - **Radar plot (Figure 10) is informative but unusual in meteorology—justify its use or replace with boxplots/seasonal profiles.**

Authors' response:
Thank you for such a detailed suggestion. As the forecasting accuracy measures are presented with four decimal places for clarity, percentage gains are not included in the table.

We have completely redesigned Figure 2 to improve clarity: "Figure 2: Yearly climate trends and thunderstorm frequency (1985–2024) for Sylhet (top), Sreemangal (middle), and Mymensingh (bottom). Annual TS frequency is shown as blue bars (left y-axis). Five climatic variables are overlaid as lines on separate right y-axes: average annual temperature (red line, °C), relative humidity (brown line, %), cloud cover (blue line, oktas), rainfall (green line, mm), and atmospheric pressure (purple line, hPa). This multi-panel design separates variables visually while maintaining temporal alignment."

Figure 9 (Forecast plots) and Figure 10 (Radar Plot) have been deleted. "Long-term historical and forecasted monthly TS frequency trend for Sylhet, Sreemangal, and Mymensingh (1985–2028) using univariate and exogenous models frameworks" is plotted in the new Figure 7.

5. Discussion

- **Discussion appropriately situates results in the climate change context, but could improve by:**
    - **Explicitly quantifying the policy implications (e.g., how much earlier warnings could be issued, or which adaptation strategies might be prioritized).**
    - **Linking to regional/global thunderstorm studies (India, China, Nepal) more directly to highlight comparative insights.**
    - **Addressing limitations: reliance on only 3 stations, no incorporation of reanalysis/satellite data, and lack of socio-economic outcome modelling.**

Authors' response:
Thank you for your suggestion. We have revised the Discussion section extensively, as follows:

- Policy Implications: We have added a new paragraph in the Discussion quantifying policy implications.
- Regional/Global Linkages: We have expanded the discussion of comparative insights: "Our findings both converge with and diverge from regional thunderstorm studies. In India, pre-monsoon thunderstorm forecasting has advanced through integration of thermodynamic indices and machine learning [21,87], consistent with our finding that

exogenous climatic variables enhance predictive skill. Studies in China have demonstrated the value of ensemble approaches combining multiple models [67], supporting our systematic comparison framework. Research in Nepal has highlighted the importance of orographic forcing in convective development [18], aligning with our attribution of Sylhet's elevated activity to its unique topographic setting. However, our station-level trend analysis revealing no significant intensification contrasts with some regional studies reporting widespread increases [26], suggesting that thunderstorm responses to climate change may be highly localized and sensitive to aggregation methods—a finding with important implications for regional climate adaptation planning."

- Limitations: We have strengthened the limitations section (Lines 790-806).

6. References

- **The reference list is comprehensive, covering climatology, forecasting, and machine learning.**
- **Some sources (blogs/tutorials like Analytics Vidhya) may not be acceptable for a high-impact journal—consider replacing with peer-reviewed methodological references.**
- **Add more citations on lightning mortality in Bangladesh and regional ENSO/IOD linkages.**

Authors' response:
We have thoroughly revised the reference list: Removed non-peer-reviewed sources,; Added lightning mortality citations; Added recent survey-based and media sources documenting lightning impacts in the study regions ; and Added ENSO/IOD linkages.

7. Language and Style

- **Generally clear, but some sections are overly technical for a generalist journal like PLOS ONE. Recommend:**
  - **Shortening methodological jargon in the abstract/introduction.**
  - **Ensuring figures and captions are self-contained.**
  - **Using consistent terminology (e.g., TS = thunderstorm vs TS days vs thunderstorm frequency).**

Authors' response:
We have carefully reviewed the manuscript for language and style. Thank you.

Summary Recommendation

- **Major revision recommended. The study is important, but revisions are needed in clarifying novelty, improving data transparency, justifying modelling choices, and refining figures/policy discussion.**
- **With these addressed, the manuscript could make a meaningful contribution to climate resilience and forecasting in South Asia.**

Authors' response:
We thank Reviewer #8 for the thorough and constructive review. The major revisions recommended have been addressed as summarized above. We believe these changes have substantially strengthened the manuscript's clarity, methodological transparency, and practical relevance. We are grateful for the reviewer's recognition that with these improvements, "the

manuscript could make a meaningful contribution to climate resilience and forecasting in South Asia."

Reviewer #9: Comments

The manuscript analyzes monthly thunderstorm (TS) occurrences over Sylhet, Sreemangal, and Mymensingh using long-term station data (1985–2024). Six forecasting techniques, Autoregressive Integrated Moving Average (ARIMA), Exponential Smoothing (ETS), Artificial Neural Network (ANN), Support Vector Regression (SVR), Extreme Gradient Boosting (XGBoost), and Long Short-Term Memory (LSTM), have been employed. Additionally, decomposition and Seasonal–Trend decomposition using Loess (STL) were used to adjust for seasonality.

The topic addressed is of considerable scientific and practical importance, particularly for regional policy planning and climate-related risk management. However, several substantive issues must be addressed. Specific comments are provided below for the authors' consideration.

Authors' response:
We thank Reviewer #9 for the thorough review and constructive comments. Many of the points raised have been addressed in our responses to other reviewers; we provide brief summaries below with references to corresponding manuscript revisions.

Comments:

1. The title "Climate Change and Seasonal Thunderstorm Intensification: Evidence from Bangladesh's Most Vulnerable Regions" suggests an analysis of thunderstorm intensification, but the manuscript only examines occurrence frequency. The authors should either incorporate analyses addressing TS intensity or revise the title to accurately reflect the study's actual content.

Authors' response:
We agree and have revised the title to: "Climate Change and Seasonal Thunderstorm Dynamics: Evidence from Bangladesh's Most Vulnerable Regions". This accurately reflects our focus on frequency rather than intensity.

2. The Introduction section requires substantial improvement. Its current structure lacks logical flow and does not clearly establish the research gap. The authors should (i) synthesize prior studies, (ii) identify knowledge deficiencies, and (iii) articulate the novelty and objectives of the present work more explicitly.

Authors' response:
We have substantially revised the Introduction to: (i) synthesize prior studies, (ii) identify knowledge deficiencies, and (iii) explicitly articulate novelty and objectives.

3. It is unclear how monthly-scale thunderstorm frequency predictions can be translated into early warning system applications, which typically operate on shorter timescales. The authors should elaborate on the operational relevance and potential end-use of such forecasts.

Authors' response:
We have added a new paragraph on operational integration in the Discussion section, explaining that monthly outlooks inform preparedness planning at weekly to monthly lead times, complementing shorter-term warnings.

4. Figure captions are currently embedded within the main text and not easily distinct. Captions should be clearly separated from the main body, numbered sequentially, and self-explanatory.

Authors' response:
All figure captions are now clearly separated from the main text, numbered sequentially, and fully self-explanatory.

5. The modeling framework is described only with basic mathematics. The authors should briefly explain the theoretical basis and functionality of each model (ARIMA, ETS, ANN, SVR, XGBoost, LSTM), highlighting their strengths, limitations, and suitability for the dataset and forecasting objectives.

Authors' response:
As noted in responses to Reviewer #4 (Comment 3) and Reviewer #5 (multiple comments), we have condensed textbook definitions and focused on each model's strengths, limitations, and suitability for our dataset (Section 2.2.1-2.2.2).

6. Multiple instances of placeholder text ("Click or tap here to enter text…") appear throughout the manuscript. These should be removed or replaced with appropriate content.

Authors' response:
All instances of "Click or tap here to enter text" have been removed from the final manuscript.

7. The manuscript uses basic performance metrics (MSE, MAE, MAPE, MASE, RMSE). The rationale for their selection should be justified, and the significance of each metric discussed. It is strongly recommended to include additional and more diagnostic statistics such as percentage bias (PBIAS), normalized RMSE/MSE, RSR (RMSE and standard deviation ratio), Nash-Sutcliffe efficiency coefficient (NSE), and Index of Agreement (d) to provide a more comprehensive assessment of model performance.

Authors' response:
We have justified our metric selection (MSE, RMSE, MAE, MASE) in Section 2.2.5. MASE was prioritized as it remains defined when actual values are zero—critical for winter months. Additional metrics (PBIAS, NSE, etc.) were considered but deemed redundant given the comprehensive evaluation already provided.

8. The Discussion section needs major revision. The authors should interpret the results in a scientific context, linking them to prior studies and physical processes. Furthermore, it is essential to explain why certain models outperform others, supported by data-driven reasoning.

Authors' response:
We have substantially revised and expanded the Discussion to: (i) interpret results in scientific context, (ii) link to prior studies, (iii) explain model performance differences with data-driven reasoning, and (iv) address limitations transparently.

9. The manuscript combines both pre-monsoon and monsoon thunderstorm events. Since the driving mechanisms differ substantially between these periods (e.g., pre-monsoon TS events are also influenced by cyclonic activity), the authors should clarify whether large-scale influences were identified and excluded prior to analysis.

Authors' response:
Thank you for this important observation. We agree that pre-monsoon and monsoon thunderstorms are driven by distinct mechanisms, with pre-monsoon events also influenced by cyclonic activity. While our exogenous models incorporating climate predictors were able to capture these seasonal differences, we acknowledge this as a limitation of the current study. We now recommend that future research examine these periods separately using higher-resolution data to better isolate the specific drivers of each season.

10. The methodological description and model validation procedure are insufficient. Details regarding model training, testing, and validation must be clearly provided. The authors should specify the data partitioning scheme (training vs. testing periods), justify its choice, and evaluate the statistical significance of the model predictions.

Authors' response:
We have added comprehensive details on: (i) data partitioning (80% training, 20% test), (ii) time-series cross-validation with expanding window (5 folds, 12-month horizon), and (iii) hyperparameter tuning with grid search (Tables S1-S2) (Section 2.2.4).

11. All figures require improvement in visual quality and resolution.

Authors' response:
All figures have been regenerated at 300 dpi resolution with improved clarity, labeling, and color schemes.

12. The relative humidity and temperature plots in Figure 2 are not adequately discussed. A physical interpretation should be added, and it is recommended to present anomalies with respect to the climatological mean to better capture deviations and variability.

Authors' response:
We have revised Figure 2 and expanded the discussion of Figure 2 to include physical interpretation of trends of five climatic variables (temperature, relative humidity, cloud cover, rainfall, atmospheric pressure). While anomalies would provide additional insight, we retained absolute values to maintain direct interpretability for BMD stakeholders. This is noted as a potential enhancement for future work.

13. Figures 4–6 present similar types of plots for different stations. For compactness and

easier comparison, these can be combined into a single multi-panel figure with clear sub-labels (e.g., 4a–4c).

Authors' response:
Figures 3-6 have been combined into a single multi-panel Figure 3 with clear caption for each station.

14. Clarify the method used for plotting trend lines in Figures 4–6. Are they running trends? If so, specify the window size. Alternatively, indicate whether these are the median of the seasonally adjusted trends. Statistical significance of the trends should also be reported.

Authors' response:
The trend lines in Figure 3 (previously Figures 4-6) are from STL decomposition (blue lines in left panel). Statistical significance of long-term trends is now formally reported in Table 1 using Mann-Kendall tests and Sen's slope estimates with confidence intervals.

15. The manuscript discusses only the occurrence frequency of thunderstorms, not their duration or intensity. Therefore, text sections implying intensity analysis should be condensed or revised accordingly.

Authors' response:
We have removed all text implying intensity analysis. The revised manuscript consistently refers to "thunderstorm frequency" throughout.

16. The role and significance of the WRF model in this study are unclear. The authors must clarify its purpose and how it relates to the TS occurrence prediction framework in the present study.

Authors' response:
We thank the reviewer for this clarification. The mention of the WRF model in the Introduction (Lines 123-125) serves only to acknowledge previous studies that have used numerical weather prediction models for thunderstorm simulation in Bangladesh [28,29]. It is not part of our methodology. To avoid confusion, we have revised the sentence to clearly indicate that this refers to prior work, not the current study: "Numerical models, such as the Advanced Research Weather Research and Forecasting (ARW-WRF) model, have been used in previous studies to simulate storms in Bangladesh [28,29]."

17. The conclusion should be rewritten to emphasize the major findings, contributions, and practical implications of the research, while avoiding repetition of earlier results.

Authors' response:
The Conclusion has been completely rewritten to summarize key findings with numerical results, articulate methodological contributions, and provide actionable recommendations for decision-makers.

We thank all reviewers for their comprehensive feedback, which has helped us substantially strengthen the manuscript.
