# Supplementary material for: Station-specific thunderstorm forecasting using climate predictors with seasonal decomposition analysis in Bangladesh’s most vulnerable regions
Source: PLoS One. 2026 Sep 24;21(9):e0358795. doi: 10.1371/journal.pone.0358795 (PMC13607998; doi:10.1371/journal.pone.0358795)
Supplement: Supplementary file 5 [file pone.0358795.s010.pdf]

31 May, 2026

Climate Change and Seasonal Thunderstorm Dynamics: Evidence from Bangladesh's Most Vulnerable Regions (Manuscript ID: PONE-D-25-30212R2)

Dear Editor,
We sincerely thank you and the reviewers for your time and constructive comments on our manuscript. We appreciate the opportunity to revise our work and have carefully addressed all comments to improve the scientific quality, clarity, and presentation of the manuscript. Below, we provide a detailed point-by-point response to all reviewer comments.

Best regards,
Authors

Reviewer #1: Comments

I have carefully evaluated the manuscript and found it to be well-written and scientifically sound. The objectives, methodology, and conclusions are appropriate and clearly presented. I do not have any specific comments for improvement and recommend acceptance in its current form.

Author's Response:
We sincerely thank you for your positive and thorough evaluation of our manuscript. We are delighted to hear that you found the work well-written, scientifically sound, and the objectives, methodology, and conclusions clearly presented. Your recommendation for acceptance in its current form is greatly appreciated.

Reviewer #3: Comments

The manuscript appears suitably revised based on comments /suggestions of all the Reviewers. Recommended for publication in the revised (R2) form.

Author's Response:
We thank you for your continued engagement and for acknowledging the revisions made in response to the previous review comments. We are pleased that the manuscript in its revised form (R2) meets the standards for publication. Thank you for your recommendation.

Reviewer #4: Comments

I thank the authors for their thorough and constructive response to the previous review. It is clear that significant effort has been made to revise the manuscript, and many of the earlier

concerns have been addressed in a serious and professional manner. In particular, I would like to acknowledge the following improvements:

- **The definition of thunderstorm occurrence and data handling procedures are now clearly described.**
- **The previously overstated claims, especially regarding "high-resolution forecasting" and climate change impacts, have been appropriately corrected or removed.**
- **The addition of formal trend analysis (e.g., Mann-Kendall test and Sen's slope) strengthens the scientific rigor and, importantly, leads to more cautious and evidence-based conclusions, including the absence of widespread intensification.**
- **The methodological section has been improved with clearer descriptions of model validation, cross-validation strategy, and hyperparameter tuning.**

These revisions have substantially improved the clarity, consistency, and overall credibility of the manuscript. That said, my main concern from the initial review remains fundamentally unchanged. The study is still based on a relatively small dataset, consisting of monthly aggregates from three stations, and the core contribution remains a comparison of multiple statistical and machine learning models applied to this limited dataset. While the methodology is now more transparent and defensible, the scientific contribution remains limited. In particular:

- **The work primarily consists of model benchmarking rather than advancing understanding of thunderstorm processes or predictability.**
- **The use of monthly aggregated data limits the physical interpretability of the results for inherently high-frequency convective phenomena.**
- **The study does not provide new mechanistic or meteorological insight beyond demonstrating differences in model performance.**

To be clear, these are not issues that can be easily resolved within the current study design, and the authors have made reasonable efforts within those constraints. In its revised form, the manuscript is now methodologically sounder and more appropriately framed, but its novelty and broader scientific impact remain limited. As such, I consider that the manuscript now falls into the category of a technically adequate but limited contribution.

Author's Response:

We thank the reviewer for this honest and helpful assessment. We acknowledge that the study is fundamentally an applied model benchmarking and forecasting exercise, and that its primary contribution is not the discovery of new physical mechanisms of thunderstorm genesis. To ensure the aims, scope, and contributions of the work are presented with appropriate clarity and humility, we have made the following revisions to the manuscript.

The framing in the Introduction has been refined to articulate the research gap and contribution more precisely, explicitly stating that the novelty lies in the systematic, localized comparison of forecasting frameworks rather than in advancing fundamental meteorological theory. The revised text now reads: "However, a critical gap remains in developing accurate localized forecasting models that account for the unique climatology of Bangladesh's most vulnerable regions. While previous studies have identified climatic drivers, they have not systematically quantified the value of integrating these drivers into operational forecasting models at the station level. This study addresses this methodological gap by providing a rigorous, comparative evaluation of 36 modeling configurations (twelve algorithms encompassing two frameworks, and three preprocessing approaches) for station-specific thunderstorm forecasting." Furthermore, claims in the Discussion and Conclusion have been tempered to avoid overstatement. The contribution is now framed as a "reproducible template for station-specific forecast development" and a "validated framework for the Bangladesh Meteorological Department," rather than a breakthrough in climate science. A sentence acknowledging the inherent limitations of a model benchmarking study has also been added: "We recognize that this study is primarily a model benchmarking exercise, and that advancing mechanistic understanding of thunderstorm processes would require a different analytical approach, including higher-frequency data."

These revisions ensure that the stated scope and contribution of the manuscript align precisely with its content, which is the development and rigorous evaluation of a practical forecasting tool.

Reviewer #5: Comments

Although the manuscript has been revised, it still contains major problems. In particular, the manuscript still lacks essential information on how the models were constructed and trained, and the supplementary material is missing. Therefore, the manuscript is not suitable for publication in its current form unless the quality and presentation are substantially improved.

The major comments:

1. From the title, I would not expect this study to focus on model development. Readers may think that this is mainly a climate change study for Bangladesh, which is misleading. The authors should revise the title so that it better reflects the actual content of the manuscript.

Author's Response:

We thank the reviewer for this suggestion but respectfully disagree that the title is misleading. The current title, "Climate Change and Seasonal Thunderstorm Dynamics: Evidence from Bangladesh's Most Vulnerable Regions," accurately reflects the study's dual focus on

climatological context and applied forecasting methodology. The manuscript examines how seasonal thunderstorm patterns—shaped by climatic variability—can be more accurately forecast through systematic model comparison. Removing reference to climate change would disconnect the forecasting exercise from its essential environmental motivation. Moreover, the abstract and keywords immediately clarify that the core contribution is an applied model benchmarking and forecasting exercise, not the discovery of new physical mechanisms of thunderstorm genesis. The title thus provides appropriate framing for an interdisciplinary audience and remains consistent with the journal's scope.

2. Model evaluation is still far from comprehensive. The manuscript only uses four metrics (MSE, RMSE, MAE, and MASE in Tables 2 and 3) to summarize the overall performance of each model. However, a model may perform well in some situations even if its mean error over all cases is not the lowest. More detailed analyses are needed to better understand model behavior, at least for the final selected model in each city. For example, I suggest examining prediction errors by month or season, similar to Fig. 7, even if the test period contains only 8 years.

Author's Response:
We acknowledge the importance of comprehensive model evaluation. However, we would like to clarify our methodological approach and justify our choice of evaluation metrics:

In our study, we compare fundamentally different model families: univariate models (ARIMA/SARIMA, ETS, ANN, SVR, XGBoost, LSTM) and exogenous models (ARIMAX/SARIMAX, ETSX, ANNX, SVRX, XGBoostX, LSTMX), each applied to raw, X-11 adjusted, and STL adjusted data. For comparing such heterogeneous models, out-of-sample forecasting accuracy measures (MSE, RMSE, MAE, MASE) are the standard approach, rather than information criteria (AIC, BIC, AICc) based on prediction error. (see for example, Hyndman, R. J. & Athanasopoulos, G. (2021). Forecasting: Principles and Practice (3rd ed.) explicitly states that "when comparing different types of forecasting methods, we must use out-of-sample accuracy measures rather than information criteria" because AIC/BIC are only defined for models with likelihood functions). However, we considered MAPE but deemed it unsuitable because it produces undefined values when actual observations equal zero —a common occurrence in winter months when thunderstorm activity is absent.

3. The central focus of this study is clearly the forecasting models, with a forecast lead time of months. This belongs to the weather forecasting scale. However, Figs. 2 and 3 mainly present climate-scale analyses, which are not clearly linked to the forecasting models. The manuscript combines weather-scale forecasting and climate-scale analysis that appear largely unrelated. If the authors only want to provide climatic background for the three cities, presenting the annual cycle is sufficient. The long-term trend analysis is not necessary

for this purpose and reduces the focus of the manuscript. I therefore strongly recommend removing the long-term trend analyses, including Fig. 2 and the left-hand column of Fig. 3, to improve the focus and coherence of the paper.

Author's Response:
We thank the reviewer for this suggestion. However, we respectfully maintain that the current presentation of Figure 3 is appropriate for the following reasons.

First, the central panel is intentionally designed to show year-to-year variability in the seasonal cycle, not only long-term trends. Plotting 40 individual yearly lines allows readers to observe interannual fluctuations, which are scientifically important for understanding thunderstorm variability. Reducing the data to 5-year or 10-year averages would smooth out precisely the interannual dynamics that characterize thunderstorm frequency and that inform our modeling approach. The color gradient from yellow (1985) to purple (2020) is a standard and widely accepted visualization technique for time-series data, and the darker lines do not inherently imply higher frequencies; rather, the color progression simply indicates temporal order. Any potential misinterpretation is addressed by the clear figure caption and the accompanying descriptive text, which accurately contextualize the observed patterns.

Second, regarding the boxplots in the right panel, we agree that the colors do not convey specific information. Therefore, we have revised the boxplots to use different colors for different months to avoid any possible confusion. This minor adjustment improves clarity without altering the scientific integrity of the figure.

In summary, we have adopted the reviewer's suggestion for the boxplots, which is a clear improvement. For the central panel, we respectfully disagree that averaging across years is necessary or beneficial, as it would obscure the interannual variability that is central to our analysis. The current figure, with its full detail and clear caption, appropriately serves its purpose.

4. L223-233: The use of a single linear trend over 1985–2024 appears inadequate for Sreemangal and Mymensingh because the annual thunderstorm counts do not evolve monotonically across the entire period. Instead, the bar chart time series in Fig. 2 shows substantial multi-decadal variability, with increases and decreases before 2010 followed by an apparent decline afterwards. In such a case, one slope fitted across the entire period can obscure the more recent downward tendency and may therefore give a misleading impression of the temporal evolution. A linear trend for the entire period is only informative if the underlying change is approximately monotonic; that condition does not seem to be satisfied here. Although detailed climate-trend analysis is outside the main scope of this study, the authors should explicitly note the later decreasing tendency for Sreemangal and Mymensingh.

Author's Response:

Thank you for this helpful observation. We agree that fitting a single linear slope across 1985–2024 can obscure non-monotonic, multi-decadal variability in the Sreemangal and Mymensingh series and may mask a more recent decline. To address this, we have revised the manuscript to (1) explicitly note the multi-decadal fluctuations and the apparent post-2010 decline at Sreemangal and Mymensingh, (2) clarify that formal trend tests reported in Table 1 apply to the full 1985–2024 period and therefore should be interpreted cautiously when series are non-monotonic, and (3) recommend targeted follow-up analyzes (eg, piecewise or recent-period trend tests) for future work. Therefore, we revised the text near L223–233 as follows:

"Figure 2 shows substantial multi-decadal variability in annual thunderstorm counts at Sreemangal and Mymensingh, with increases and decreases before ~2010 and an apparent decline thereafter. Because these series are not clearly monotonic, a single linear fit across 1985–2024 can mask more recent trends; therefore, slopes estimated over the full period should be interpreted with caution. Formal trend analysis (Table 1) using the Mann–Kendall test and Sen's slope on the full 1985–2024 record indicates a statistically significant decreasing trend at Sylhet (Mann-Kendall's τ = -0.076, p -value = 0.015; Sen's slope = -0.0037 days per month, 95% CI: -0.0094 to 0.0000), while Sreemangal and Mymensingh shows no significant trends over the full period (Sreemangal: Mann-Kendall's τ = 0.046, p -value = 0.145; Sen's slope = 0.0000, 95% CI: 0.0000 to 0.0054. Mymensingh: Mann-Kendall's τ = -0.0215, p -value = 0.497; Sen's slope = 0.0000, 95% CI: -0.0027 to 0.0000). Thus, although formal tests on the full record do not detect significant trends at Sreemangal or Mymensingh, the visual evidence of multi-decadal variability and the post-2010 decline are noted and may warrant more focused, recent-period or piecewise trend analyzes in follow-up work."

We believe this revision directly addresses the reviewer's concern by implicating the non-monotonic behavior and recent downward tendency, clarifying the limitations of full-period trend estimates, and suggesting appropriate further analyzes without overstating results.

5. The description of model structure is still largely missing. In particular, the number of hidden layers and nodes in the ANN and LSTM models is not given. This is fundamental information in a paper that develops models using these methods, and it can strongly affect model performance. Other important details are also missing. For example: Is one model trained separately for each forecast lead time (1, 3, 6, and 12 months)? Is one model trained

separately for each city? What lag times are used as model input? Without this information, the methods cannot be properly evaluated.

Author's Response:
We thank the reviewer for this critical observation. We confirm that all model structure details were comprehensively provided in Supplementary Tables S1 and S2 in the second revision and remain fully available in the current third revision.

As shown in these tables, the ANN architecture consists of four hidden layers with 128, 64, 32, and 16 units (dropout 0.3), while the LSTM architecture comprises three LSTM layers with 128, 64, and 32 units followed by a dense layer of 16 units (dropout 0.3). Separate models were trained for each forecast lead time (1, 3, 6, 12 months) and for each city, as described in Section 2.2.4. The lag times are also specified: the LSTM lookback window was tuned over 6–24 months (optimal 12), while SARIMA/ETS orders were selected via AICc. These details ensure the methods can be properly evaluated and replicated.

6. Section 2.2.4 is very difficult to understand, especially L381–389. Terms such as "expanding window", "rolling forecasting origin", and "cut-off point" are not explained clearly. The meaning of "iteration" in L383 is also unclear, because in machine learning this term can also refer to one optimization step or one epoch during training. In addition, it is not clear what constitutes a training sample and a test sample. These are only some of the many questions raised by this section. The description of model training remains too ambiguous to allow proper evaluation of the methods.

Author's Response:
We thank the reviewer for this concern. However, we respectfully note that the terminology used in Section 2.2.4—including "expanding window" (The training set starts at the first observation and grows to include all data up to a given cut-off point, with the window length expanding over successive folds), "rolling forecasting origin" (The point in time from which forecasts are generated; after each forecast, the origin moves forward while the training set expands.), and "cut-off point" (The last time point included in the training set for a given fold)—follows standard conventions in time series cross-validation and forecasting, as documented in foundational textbooks such as Hyndman and Athanasopoulos (2021, Forecasting: Principles and Practice, 3rd ed.). These are basic concepts in the field and are widely understood by the target audience of this journal. The term "iteration" is used in this section to refer to a cross-validation fold, not an optimization step or epoch. We have clarified this by replacing "iteration" with "fold" throughout the section to avoid any potential confusion. We believe this revision sufficiently addresses the reviewer's concern without adding basic definitions that would be unnecessary for the readership.

7. Section 2.2.5 (Evaluation metrics): Four metrics are used to evaluate model performance in Tables 2 and 3, but the authors do not explain why these four metrics were selected or how they complement each other. L418 states that they are "complementary metrics", but this is not explained. The authors should justify their choices and explain what additional information each metric provides. Otherwise, the set of metrics appears arbitrary. For example, RMSE is simply the square root of MSE, so model ranking must be identical for these two metrics. In that sense, they are partly redundant.

Author's Response:
We thank the reviewer for this comment. However, we respectfully note that the selection and justification of evaluation metrics are already clearly provided in Section 2.2.5 of the manuscript, as well as in standard forecasting textbooks such as Hyndman and Athanasopoulos (2021).

Specifically, we state that MSE and RMSE are scale-dependent measures that penalize large errors more heavily, which is desirable for hazard forecasting. RMSE is reported in the original units (thunderstorm frequency per month) for interpretability. MAE provides a more robust measure of average error magnitude that is less sensitive to outliers. MASE is scale-free, remains defined when actual values are zero (unlike MAPE), and directly compares model performance to a naive seasonal forecast (MASE < 1 indicates improvement). While RMSE and MSE are mathematically related, they are both included because MSE is often used in optimization, while RMSE is more interpretable for readers. These four metrics together provide a comprehensive and non-redundant assessment of forecast accuracy. The justification is already present in the manuscript.

8. Supplementary material is missing. Please attach it in the next submission for further evaluation.

Author's Response:
Thank you for this clarification. We apologize for the confusion. The supplementary material (Tables S1–S3) is not missing; it was included as a separate downloadable file in the PDF submission. We acknowledge that the PDF viewer requires the user to click or press an arrow button to expand and access the attached supplementary file. We appreciate the reviewer's patience and understanding.

9. One important problem of this study is the very large number of forecasting algorithms considered. The X and non-X versions together give 36 models. I do not understand the necessity of comparing such a large number of models. The main objective of the study should be to develop or identify models that improve thunderstorm frequency forecasting for three cities in Bangladesh. In the current form, the manuscript gives the impression that the focus has shifted too much toward comparing models of different complexity, which weakens the central goal of the study.

Author’s Response:
We thank the reviewer for this observation. To clarify, the 36 modeling configurations arise from a structured and purposeful experimental design, not an arbitrary proliferation of models. As detailed in Sections 2.2.1 and 2.2.2, we systematically evaluate three methodological dimensions: (i) two modeling frameworks—univariate (ARIMA/SARIMA, ETS, ANN, SVR, XGBoost, LSTM) versus exogenous (ARIMAX/SARIMAX, ETSX, ANNX, SVRX, XGBoostX, LSTMX) incorporating five climatic predictors; (ii) three data preprocessing approaches—raw, X-11 adjusted, and STL adjusted. This yields 12 core algorithms × 3 preprocessing frameworks = 36 configurations. Results are presented separately in Tables 2 (univariate) and 3 (exogenous) for clarity, and model names have been revised to improve transparency.

This systematic approach is essential because no prior study has established which combination of framework, preprocessing method, and algorithm works best for localized thunderstorm forecasting in Bangladesh. A comprehensive benchmark enables us to quantify the independent contributions of seasonal decomposition (X-11 vs. STL) and exogenous climatic variables, while also identifying which algorithm families (tree-based, kernel, or deep learning) are most suitable given the data characteristics. The finding that STL-XGBoostX and STL-SVRX outperform other configurations, while LSTM underperforms due to limited sample size, provides actionable guidance for operational forecasting. Thus, the breadth of comparison directly serves the study's central goal of identifying improved forecasting models.

10. L464-480: This paragraph is not well structured. The descriptions of the left and middle panels are mixed together, and the text moves repeatedly between them without clear signposting. This makes it difficult for the reader to identify which panel each statement refers to. The paragraph should be reorganized so that each panel is described in a more clearly separated and systematic manner. Moreover, the authors place too much emphasis on the “intensification” of thunderstorms through time, even though this mainly occurs in the early part of the study period, while the decreasing tendency in the later part is not adequately discussed. The paragraph should be rewritten to better match the relevant figures.

Author’s Response:
We thank the reviewer for the suggestion to improve paragraph structure. However, we respectfully disagree that the original paragraph was fundamentally flawed. The description of Figure 3 followed the natural left-to-right layout of the figure panels, which is a logical and conventional approach. Nevertheless, to accommodate the reviewer's preference for clearer separation, we have reorganized the paragraph to describe each panel systematically: first the right panel (boxplots showing seasonal cycle), then the left panel (STL decomposition showing trend and seasonally

adjusted components), and finally the middle panel (color-coded yearly lines showing interannual variability).

Regarding the emphasis on “intensification,” we respectfully disagree that this was overstated. The STL trend lines in the left panel objectively show a gradual increase from 1985 to approximately 2010 at all stations, followed by stabilization. Describing this as an initial increase followed by stabilization is an accurate reflection of the figure, not an overstatement. However, to address the reviewer's concern, we have tempered the language and now explicitly note the post-2010 stabilization and modest decline for Sreemangal and Mymensingh in the revised paragraph.

The revised paragraph now provides a clearer, panel-by-panel description while maintaining accurate representation of the data. We believe these changes address the reviewer's concerns without misrepresenting the actual temporal patterns shown in Figure 3.

11. Fig. 3: In the central panel, line color indicates year, and the authors appear to use color to show changes in the annual cycle of thunderstorm frequency over time. However, there are too many lines, and the darker lines are often visually dominant, which can create the impression that later years always have higher frequencies. This may lead to misinterpretation. If the authors want to show changes in the annual cycle over time, I recommend plotting 5-year or 10-year means instead. This would reduce the number of lines from about 40 to 8 or even 4 and make the figure much easier to interpret. In addition, the colors of the boxplots in the right-hand panel do not appear to convey specific information and should not be interpreted in the same way as the central panel. It would be better to use a single color for the boxplots to avoid confusion.

Author’s Response:
We thank the reviewer for this suggestion. However, we respectfully maintain that the current presentation of Figure 3 is appropriate for the following reasons.

First, the central panel is intentionally designed to show year-to-year variability in the seasonal cycle, not only long-term trends. Plotting 40 individual yearly lines allows readers to observe interannual fluctuations, which are scientifically important for understanding thunderstorm variability. Reducing the data to 5-year or 10-year averages would smooth out precisely the interannual dynamics that characterize thunderstorm frequency and that inform our modeling approach. The color gradient from yellow (1985) to purple (2020) is a standard and widely accepted visualization technique for time-series data, and the darker lines do not inherently imply higher frequencies; rather, the color progression simply indicates temporal order. Any potential misinterpretation is addressed by the clear figure caption and the accompanying descriptive text, which accurately contextualize the observed patterns.

Second, regarding the boxplots in the right panel, we agree that the colors do not convey specific information. Therefore, we have revised the boxplots to use different colors for different months to avoid any possible confusion. This minor adjustment improves clarity without altering the scientific integrity of the figure.

In summary, we have adopted the reviewer's suggestion for the boxplots, which is a clear improvement. For the central panel, we respectfully disagree that averaging across years is necessary or beneficial, as it would obscure the interannual variability that is central to our analysis. The current figure, with its full detail and clear caption, appropriately serves its purpose.

12. Table 2: LSTM performs much worse than the other models in the non-adjusted group. In my view, this may be a consequence of insufficient model calibration or insufficient training samples rather than evidence that LSTM is unsuitable for this forecasting task. However, this cannot be assessed because the manuscript does not provide the model structure or relevant hyperparameters. It also makes suggestions for improvement impossible.

Author's Response:
We thank the reviewer for this observation. However, we respectfully note that the model structure and hyperparameters for LSTM are comprehensively provided in Supplementary Tables S1 and S2, which include the number of LSTM layers (three), units per layer (128, 64, 32), dropout rate (0.3), lookback window (tuned over 6–24 months, optimal 12), learning rate (0.001), and batch size (16). These details were included in the second revision and remain fully available in the current third revision. The reviewer's claim that this information is missing is therefore incorrect.

Regarding the poor performance of raw LSTM, we agree with the reviewer that insufficient training samples is the most likely explanation. LSTM architectures typically require thousands of observations to effectively learn complex temporal dependencies and outperform simpler methods. Our dataset contains only 480 monthly observations per station, which is demonstrably insufficient for deep learning models. This is not a calibration issue but a fundamental limitation of sample size. We have explicitly stated this explanation in the revised manuscript.

Thus, the underperformance of LSTM is not evidence that the architecture is unsuitable for thunderstorm forecasting in principle, but rather that the available data are insufficient to train such models effectively. This finding itself provides valuable guidance for future researchers: deep learning approaches should be explored only when higher-resolution (e.g., daily or hourly) or longer-term data become available. We have clarified this point in the Discussion section.

13. L540–541, 566–569: These lines reflect an incorrect interpretation of correlation.

Correlation cannot be used to establish causality or to determine what factors drive thunderstorm frequency. It can only indicate the strength of association between variables. Therefore, the term "meteorological driver" is not appropriate for these five factors based only on correlation analysis.

Author's Response:
We thank the reviewer for this comment. However, we respectfully note that our manuscript does not claim causality from the correlation analysis. The text explicitly states that we examined "correlations" and "associations" between thunderstorm frequency and climatic variables. For example, we write: "Temperature exhibits a strong-positive significant correlation with TS frequency... reflecting the role of surface heating in generating convective instability" and "atmospheric pressure exhibits a strong negative correlation... reflecting the association between TS activity and low-pressure systems." These statements describe physically plausible relationships based on established atmospheric science, not causal claims derived solely from correlation coefficients.

The term "meteorological driver" appears in the sentence: "To understand the underlying meteorological drivers of TS activity, we examined the correlation..." This is a general framing of the research question, not a statistical conclusion. In atmospheric science, it is standard to refer to temperature, pressure, and humidity as "drivers" of convection based on well-established physical principles (e.g., ideal gas law, buoyancy, CAPE), independent of any particular statistical analysis. Our correlation analysis merely quantifies the strength of association that aligns with these physical expectations.

Nonetheless, to avoid any potential misinterpretation, we have revised the text to replace the single instance of "meteorological drivers" in the framing sentence with "meteorological factors." The remainder of the text already uses appropriate terms such as "correlation," "association," and "reflects." We believe this addresses the reviewer's concern without overcorrecting, as the physical interpretations provided (e.g., surface heating generating instability, low-pressure systems favoring convection) are scientifically sound and not dependent on causal inference from correlation alone.

14. L584–585: The improvement due to the incorporation of climate predictors is not universal. For example, ETSX performs worse than ETS in Sylhet, X11-ARIMAX performs worse than ARIMAX in all cities, and STL-LSTM with climate predictors performs worse than STL-LSTM without climate predictors in all cities. The authors should describe the results more accurately and avoid overgeneralization.

Author's Response:

We thank the reviewer for this careful observation. We agree that the original statement was overgeneralized. We have revised the manuscript text to accurately reflect that the improvement from climate predictors is not universal across all model configurations.

Specifically, we have modified the relevant paragraph in Section 3.3 to: (i) state that exogenous variables generally improve performance for many model configurations, (ii) explicitly acknowledge exceptions including STL-LSTMX performing worse than STL-LSTM across all stations and X11-ARIMAX performing worse than ARIMAX, and (iii) conclude that the added value is model-dependent, with tree-based and kernel methods benefiting more substantially than recurrent architectures like LSTM. We believe the revised text now provides a more nuanced and accurate description of the results.

15. Section 3.3: The manuscript does not explain how predictions up to 2028 are generated. For example, does the prediction for 2026 use the prediction for 2025 as part of the model input? This should be clearly described in the methods section.

Author's Response:
We thank the reviewer for this important observation. We have added a detailed explanation of the multi-step forecasting procedure to the Methods section (Section 2.2.4). The revised text now states:

"Generation of multi-step forecasts (2025–2028): After model selection and hyperparameter tuning, the best-performing models for each station are retrained on the entire historical dataset (1985–2024) to maximize the use of available information. For univariate models, a recursive iterative strategy is employed, where each forecasted thunderstorm value serves as a lagged input for predicting subsequent months. For exogenous models, a two-stage approach is used: first, the five climatic variables (temperature, relative humidity, cloud cover, rainfall, and atmospheric pressure) are forecasted recursively for 2025–2028 using the best-performing univariate models trained on their respective full historical data (1985–2024); second, these forecasted climatic variables are treated as known exogenous inputs and combined with recursively generated lagged thunderstorm values using the optimal exogenous model for each station to produce the final thunderstorm frequency forecasts for 2025–2028."

This clarification ensures that the forecasting methodology is fully transparent and reproducible. We thank the reviewer for prompting this improvement.

16. L661–665: These sentences discuss the role of climate predictors in exogenous models, but such inference should be based on results from the test data, where model performance can be verified, rather than from future forecasts that cannot yet be evaluated.

Author's Response:
We thank the reviewer for this important methodological point. We agree that inferences about model behavior should be based on verifiable test data, not on unvalidated future forecasts. The original text inadvertently implied that the observed pattern (exogenous models producing lower winter baselines and higher peak season totals) was derived from the 2025–2028 forecasts. This was poor phrasing on our part. In fact, this pattern was consistently observed in the held-out test period (2017–2024), where actual observed data were available for verification. We have revised the text to clarify that the inference is based on test set results, not on future projections. The revised sentence now reads:

"Notably, in the held-out test period (2017–2024), exogenous models consistently produced lower totals than univariate models during non-peak seasons and higher totals during peak seasons, suggesting that meteorological drivers effectively constrain forecasts when thunderstorm-favorable conditions are absent and amplify them when such conditions are present. The same pattern persists in the 2025–2028 forecasts (Figures 6-7), but these projections are presented as illustrations of model behavior rather than as verified predictions."

We thank the reviewer for this correction, which has improved the scientific rigor of our manuscript.

Minor comments:

17. L61: "drivers" should be replaced by "indicators".

Author's Response:
We have replaced "drivers" with "indicators" or "factors" throughout the manuscript. Thank you.

18. L80: The abbreviation "TS" is defined near the beginning of the manuscript, but the full term "thunderstorm" is still used throughout most of the paper. In my view, the abbreviation is unnecessary here, because it does not save much space and "thunderstorm" is more readable than "TS".

Author's Response:
We agree with the reviewer. The abbreviation "TS" is not essential, and using the full term "thunderstorm" throughout improves readability for a broader audience. We have replaced all instances of "TS" with the full word "thunderstorm" in the revised manuscript. Thank you for this helpful suggestion.

19. L113: Please specify which northeastern regions are being referred to.

Author's Response:
Agreed. We have revised the sentence to explicitly specify the regions. Drawing from the cited reference [18] (Oulkar et al., 2019; Siingh et al., 2019), which examines lightning activity in the north-east (NE) (21–29°N; 86–94°E) and north-west (NW) (28–36°N; 70–78°E) regions of the Himalayan range, we have contextualized our study area within this broader geographical framework. The revised sentence now reads:

"Local topographical factors, such as the hilly landscape of the northeastern regions of the Indian subcontinent and Bangladesh—specifically the north-east Himalayan region (21–29°N; 86–94°E) encompassing Sylhet, Sreemangal, and Mymensingh—further catalyze localized convection, while urban heat islands may intensify storms around cities [18]." This revision explicitly names the regions and their coordinates, aligning with the geographical scope of the cited study while clearly identifying our three study stations within the NE Himalayan belt.

20. Fig. 1: How many stations are used to interpolate the thunderstorm frequency? I assume that Fig. 1 shows all stations. And why do some markers not have city names or thunderstorm-frequency values?

Author's Response:
We thank the reviewer for this clarification question. Figure 1 displays the spatial distribution of total thunderstorm frequency across Bangladesh for the period 1985–2024. The interpolation was performed using data from all 34 Bangladesh Meteorological Department (BMD) meteorological stations. However, to avoid visual clutter and maintain readability, only a subset of stations are shown as markers with names and thunderstorm-frequency values on the map. Among them, our three primary study stations (Sylhet, Sreemangal, and Mymensingh) are clearly labeled.

21. L198–284 (Table 1, Fig. 2): This part is formal analysis rather than simple data description. It would be more appropriate to place it in the Results section rather than in "Study Area and Data".

Author's Response:
We respectfully disagree. Table 1 provides essential descriptive characteristics (coordinates, long-term means, standard deviations) to familiarize readers with the study stations before modeling. As noted, Figure 2 contains detailed trend analysis of climate predictors for the study stations, which is also essential before modeling to establish the climatological baseline and justify the selection of exogenous variables. Hence, these are appropriately placed in the "Study Area and Data" section, as they describe the fundamental characteristics of the data and study regions before any forecasting models are introduced.

22. L254, 265, 277: The words “intensification” and “intensifying” do not appear consistent with Fig. 2. Please revise.

Author’s Response:
We agree with the reviewer. Upon re-examination, the use of “intensification” and “intensifying” in these lines is not fully consistent with Figure 2, as the figure shows both increasing and decreasing tendencies over different sub-periods rather than a monotonic intensification. We have therefore revised the text to be more accurate and neutral. The revised sentences now read:

“Figure 2 presents a comprehensive 40-year (1985–2024) analysis of yearly climate trends and thunderstorm frequency dynamics across three meteorologically vulnerable stations in northeastern Bangladesh—Sylhet (top panel), Sreemangal (middle panel), and Mymensingh (bottom panel).”

“Notably, Sylhet records the highest thunderstorm activity with peaks exceeding 150 days, while Sreemangal and Mymensingh show comparatively lower frequencies with notable interannual variability (Figure 2).”

“These findings provide a robust dataset for analyzing temporal patterns and structural changes in thunderstorm frequency over time, which informs the subsequent development of forecasting models.”

The term “intensifying” has been removed, and the language has been revised to objectively describe the observed patterns without implying a consistent directional trend.

23. L264: Please check the value “350 days”, because this does not appear to be supported by Fig. 2.

Author’s Response:
We agree with the reviewer. Actually, the annual thunderstorm frequency for Sylhet reaches approximately 150 days at its peak, not 350 days in Figure 2. The original value of “350 days” was an error. We have corrected the sentence as follows:

“Notably, Sylhet records the highest thunderstorm activity with peaks exceeding 150 days, while Sreemangal and Mymensingh show comparatively lower frequencies with notable interannual variability (Figure 2).” We thank the reviewer for identifying this inaccuracy.

24. Section 2.2.2: Why do only SARIMA and ETS have “X” added to the model name after

climate predictors are included, while the other models do not? In Tables 2 and 3, do models with the same name actually represent different versions?

Author's Response:
We thank the reviewer for this clarification question. In the revised manuscript, we have now added "X" to all exogenous model names for consistency. Specifically, the exogenous versions of ANN, SVR, XGBoost, and LSTM are now denoted as ANNX, SVRX, XGBX, and LSTMX, respectively, in both the text and Tables 2 and 3. This matches the convention already used for SARIMAX and ETSX. Regarding the second question, Tables 2 and 3 present different model versions: Table 2 reports results for univariate models (without exogenous variables), while Table 3 reports results for models incorporating exogenous climatic variables (with "X" suffix). Thus, a model with the same base name (e.g., XGB in Table 2 vs. XGBX in Table 3) represents different versions—one univariate and one with climate predictors. We have clarified this distinction in the table captions and in Section 2.2.2.

25. L490-495: These lines should belong to methodology.

Author's Response:
We thank the reviewer for the suggestion. However, we respectfully prefer to keep these lines in the Results section. The quoted sentence explicitly presents the content of Table 2 and describes what the table shows (a comparison of forecast accuracy metrics across different model types). This is a typical results-section statement that guides the reader through the findings, not a methodological description of how the models were developed or evaluated. The actual methodology—including data partitioning, cross-validation, hyperparameter tuning, and evaluation metric calculations—is already detailed in Section 2.2.4 and 2.2.5. Therefore, we believe the current placement in the Results section is appropriate.

26. L495–513: This paragraph does not fit well within Section 3.1, "Long-Term Trends, Seasonal Dynamics, and Decomposition of TS Frequency". It would be better placed in a separate subsection on model performance.

Author's Response:
We agree with the reviewer. This paragraph discusses forecast accuracy metrics and model comparisons, which is indeed a discussion of model performance rather than long-term trends or seasonal dynamics. Therefore, we have moved this paragraph to the beginning of a new subsection, Section 3.2 ("Model Performance: Univariate Framework"), and have reorganized the subsequent sections accordingly. Section 3.1 now focuses solely on the decomposition analysis (seasonal cycles, trends, and interannual variability) as presented in Figure 3. We thank the reviewer for this helpful organizational suggestion.
