# Supplementary material for: Station-specific thunderstorm forecasting using climate predictors with seasonal decomposition analysis in Bangladesh’s most vulnerable regions
Source: PLoS One. 2026 Sep 24;21(9):e0358795. doi: 10.1371/journal.pone.0358795 (PMC13607998; doi:10.1371/journal.pone.0358795)
Supplement: Supplementary file 6 [file pone.0358795.s011.pdf]

10 August, 2026

Climate Change and Seasonal Thunderstorm Dynamics: Evidence from Bangladesh's Most Vulnerable Regions (Manuscript ID: PONE-D-25-30212R3)

Dear Editor,

We sincerely thank you and the reviewers for your valuable time, careful evaluation, and insightful comments on our revised manuscript. We greatly appreciate the opportunity to further improve our work. We have revised the title to more accurately reflect the manuscript's core contribution. The new title, "**Station-Specific Thunderstorm Forecasting using Climate Predictors with Seasonal Decomposition Analysis in Bangladesh's Most Vulnerable Regions**", now foregrounds the study's actual contribution (station-specific forecasting with climate predictors and seasonal decomposition) rather than implying climate-change evidence that our own trend results do not support. All other revisions described below have been made in response to the specific reviewer comments.

We have carefully considered each comment and made corresponding revisions to enhance the manuscript's scientific quality, clarity, and overall presentation. Below, we provide a detailed point-by-point response to each editorial and reviewer comment.

Best regards,
Authors

Editorial Requirements

We've checked your submission and before we can proceed, we need you to address the following issues:

1. When completing the data availability statement of the submission form, you indicated that you will make your data available on acceptance. We strongly recommend all authors decide on a data sharing plan before acceptance, as the process can be lengthy and hold up publication timelines. Please note that, though access restrictions are acceptable now, your entire data will need to be made freely accessible if your manuscript is accepted for publication. This policy applies to all data except where public deposition would breach compliance with the protocol approved by your research ethics board. If you are unable to adhere to our open data policy, please kindly revise your statement to explain your reasoning and we will seek the editor's input on an exemption. Please be assured that, once you have provided your new statement, the assessment of your exemption will not hold up the peer review process.

Author's Response:
We have revised the Data Availability Statement to clearly confirm that the full dataset is already provided as Supporting Information File S1_Dataset with our submission. This file contains the complete monthly thunderstorm frequency records and all five climatic variables (temperature,

relative humidity, cloud cover, rainfall, and atmospheric pressure) for the three stations over the 1985–2024 period. Since the data are already freely accessible to readers and reviewers, we do not require any exemption from PLOS ONE's open data policy. The updated statement reflects this and also notes that all author-generated code will be shared via a public GitHub repository upon acceptance.

2. Can you please upload an additional copy of your revised manuscript that does not contain any tracked changes or highlighting as your main article file. This will be used in the production process if your manuscript is accepted. Please amend the file type for the file showing your changes to Revised Manuscript w/tracked changes. Please follow this link for more information: http://blogs.PLOS.org/everyone/2011/05/10/how-to-submit-your-revised-manuscript/.

Author's Response:
As requested, we have uploaded two separate files to the submission system: (1) a clean version of the revised manuscript, with all highlighting removed; and (2) a tracked-changes version of the revised manuscript to facilitate the review process. We have followed the instructions provided in the PLOS ONE blog post and confirmed that both files are correctly designated in Editorial Manager.

3.We note that Figure 3 in your submission contain [map/satellite] images which may be copyrighted. All PLOS content is published under the Creative Commons Attribution License (CC BY 4.0), which means that the manuscript, images, and Supporting Information files will be freely available online, and any third party is permitted to access, download, copy, distribute, and use these materials in any way, even commercially, with proper attribution. For these reasons, we cannot publish previously copyrighted maps or satellite images created using proprietary data, such as Google software (Google Maps, Street View, and Earth). For more information, see our copyright guidelines: http://journals.plos.org/plosone/s/licenses-and-copyright.

Author's Response:
We confirm that Figure 3 does NOT contain any copyrighted or proprietary material. This figure presents statistical decompositions (STL) and monthly distribution boxplots of thunderstorm frequencies – it is entirely a data visualization, not a map or satellite image.

This figure is entirely original – it was generated using our own R code and open-source packages (ggplot2, dplyr, lubridate, forecast, seasonal, viridis, patchwork, and RColorBrewer) based on our own dataset from the Bangladesh Meteorological Department. It contains no maps, satellite images, or proprietary third-party material, and therefore requires no permission. The code will be shared via a public GitHub repository upon acceptance.

Journal Requirements

If the reviewer comments include a recommendation to cite specific previously published works, please review and evaluate these publications to determine whether they are relevant and should be cited. There is no requirement to cite these works unless the editor has indicated otherwise.

Author's Response:
We thank the Editor for this guidance. We carefully reviewed all reviewer comments and confirmed that no specific previously published works were recommended for citation. Therefore, no additional references were added in this regard.

Reviewer #1 Comments

I do not have any specific comments for improvement and recommend acceptance in its current form. Not further comment.

Author's Response:
We sincerely thank the reviewer for the positive evaluation of our manuscript and for recommending its acceptance. We greatly appreciate your time and encouraging comments.

Reviewer #3 Comments

The manuscript has been suitably revised based on the comments / suggestions of all the Reviewers. Recommended for publication in the revised (R3) form.

Author's Response:
We sincerely thank the reviewer for the positive assessment of our revised manuscript and for recognizing our efforts in addressing the previous comments. We appreciate your recommendation for publication.

Reviewer #4 Comments

I thank the authors for their R3 response. However, the revised manuscript still contains issues that were not resolved as claimed:

Comment 1: The title "Climate Change and Seasonal Thunderstorm Dynamics: Evidence from Bangladesh's Most Vulnerable Regions" implies the paper provides evidence about climate change and thunderstorm dynamics. The paper's own trend analysis, however, finds either no significant trend or a statistically significant decreasing trend at Sylhet. The manuscript's core contribution is

a forecasting model comparison, as the authors themselves state. The title must be changed to accurately reflect the paper's actual contribution (a station-specific forecasting model comparison) rather than implying climate change evidence that the paper's own results do not support.

Author's Response:
We fully agree with the reviewer's assessment. Upon careful reflection, we recognize that the original title overstates the climate change evidence presented in our study. While our paper does examine long-term trends and incorporates climatic variables as predictors, the core contribution is indeed the development and rigorous comparison of station-specific forecasting models with seasonal decomposition analysis. We have therefore revised the title to: "Station-Specific Thunderstorm Forecasting using Climate Predictors with Seasonal Decomposition Analysis in Bangladesh's Most Vulnerable Regions."

This retains the geographic and risk framing ("Bangladesh's Most Vulnerable Regions") established in earlier revisions, while accurately foregrounding the manuscript's actual, and already explicitly stated, contribution: station-specific forecasting that incorporates climate variables (temperature, humidity, cloud cover, rainfall, and pressure) as exogenous predictors, alongside the X11/STL seasonal decomposition preprocessing that is central to our modeling framework. This descriptive framing accurately reflects what the manuscript does (use climate predictors and seasonal decomposition as part of the forecasting pipeline) without implying, as the previous title did, that the manuscript's own trend results constitute evidence of climate-change-driven intensification, which the reviewer correctly notes they do not. We have also updated the keyword list, replacing "Climate change" with "Climate predictors" and "Model benchmarking", consistent with the existing "Seasonal adjustment" and "STL decomposition" keywords already reflected in the new title. We thank the reviewer for their persistence on this point, which has led to a title that is now fully consistent with our findings.

Comment 2: The trend analysis identifies a statistically significant decreasing trend at Sylhet. Yet lines 827-831 interpret the long-term Sylhet forecast (2025-2049) as projecting intensification under continued warming, with frequency rising from ~62 to ~86 days annually. These two findings directly contradict each other therefore cannot coexist without explicit mechanistic justification, which is not provided.

Author's Response:
We thank the reviewer for identifying this inconsistency. We agree that the two results were presented in unexplained tension and have revised both passages.

First, we identified a related error that the reviewer's comment prompted us to re-examine: the Figure 2 description in Section 3.1 (in the revised manuscript) previously stated "All stations

exhibit a positive trend in thunderstorm frequency (e.g., +0.52 counts/year for Sylhet)," which directly contradicted the formal Mann-Kendall result reported in the same section. This has been corrected to: "All stations exhibit substantial multi-decadal variability in thunderstorm frequency, consistent with the formal trend results reported in Table 1 rather than a uniform directional trend."

Second, the long-term forecast interpretation in Section 3.3 previously read: "…suggesting that under continued warming and favorable moisture conditions, Sylhet's already high thunderstorm activity could intensify further, with important implications for disaster preparedness and infrastructure resilience." This has been revised to: "This extrapolated increase is a statistical projection of the fitted model rather than a validated forecast or a claim about underlying physical drivers, and it should be interpreted with caution given that it runs counter to the significant historical decreasing trend detected at Sylhet over 1985–2024 (Section 3.1). We flag this discrepancy as an open question for future work combining longer forecast validation with mechanistic climate analysis, rather than resolving it here."

Third, on further internal review we identified a related passage discussing the near-term (2025–2028) seasonal forecasts that required the same correction; it now closes with: "We note that the apparent contradiction between the historical decreasing trend at Sylhet and the projected increase in later forecast years is a property of the fitted statistical model rather than a demonstrated physical mechanism; we have not established a causal link to warming in this study, and this discrepancy is flagged as an open question for future work combining longer forecast validation with mechanistic climate analysis." This ensures the manuscript does not attribute the projected increase to warming anywhere, consistent with the correction described above.

Comment 3: The opening paragraph of the Discussion still asserts "mounting evidence of a troubling rise in the frequency, duration, and intensity of localized thunderstorm", which is not supported by the manuscript's own results and also is the overstatement the authors committed to removing in R3.

Author's Response:
We thank the reviewer for catching this. This sentence was intended to be softened during the R3 revision along with other overstated claims but was inadvertently left unchanged; we apologize for the oversight and have now corrected it.

The sentence in the opening paragraph of the Discussion, previously reading "…there is mounting evidence of a troubling rise in the frequency, duration, and intensity of localized thunderstorm, especially during pre-monsoon and monsoon seasons," has been revised. On further reflection, we went beyond simply removing the overstatement and rewrote the opening to state our own trend results directly: it now reads "While cyclones and floods have historically dominated climate risk discourse, thunderstorms represent one of the most significant convective hazards in Bangladesh,

with documented impacts on public health, agriculture, and infrastructure [8,79,80]. Climate change projections and global studies suggest that warming conditions may intensify convective activity in many regions [12-15], though station-level trend analyses in Bangladesh show heterogeneous patterns that warrant continued monitoring and localized forecasting efforts. Our own analysis reveals a statistically significant decreasing trend at Sylhet, while Sreemangal and Mymensingh show no significant trends over the 1985-2024 period. Despite these heterogeneous trends, the development of accurate station-specific forecasting models remains essential for early warning and disaster preparedness in these vulnerable regions." We believe this more directly closes the loop between the Discussion's framing and our own Results, rather than merely avoiding the previous overstatement.

We thank the reviewer again for their careful, cross-round scrutiny, which has materially improved the internal consistency of the manuscript. All changes described above are highlighted in the attached revised manuscript.
