# Supplementary material for: Station-specific thunderstorm forecasting using climate predictors with seasonal decomposition analysis in Bangladesh’s most vulnerable regions
Source: PLoS One. 2026 Sep 24;21(9):e0358795. doi: 10.1371/journal.pone.0358795 (PMC13607998; doi:10.1371/journal.pone.0358795)
Supplement: Supplementary file 7 [file pone.0358795.s001.docx]

**Supplementary Table S1:** Hyperparameter tuning ranges, optimal values, and validation performance for univariate models applied to Sylhet, Sreemangal, and Mymensingh thunderstorm frequency data. Grid search with expanding window time series cross-validation (5 folds, 12-month horizon) was used for selection. Optimal values were consistent across stations due to identical seasonal structure.

| **Model** | **Hyperparameter** | **Tuning Range** | **Tuned Value (Sylhet)** | **Tuned Value (Sreemangal)** | **Tuned Value (Mymensingh)** |
| --- | --- | --- | --- | --- | --- |
| XGBoost | n_estimators | [100, 200, 300, 400] | 200 | 200 | 200 |
|  | max_depth | [3, 4, 5, 6, 7] | 6 | 6 | 6 |
|  | learning_rate | [0.01, 0.05, 0.1, 0.2] | 0.05 | 0.05 | 0.05 |
|  | subsample | [0.6, 0.7, 0.8, 0.9] | 0.8 | 0.8 | 0.8 |
|  | colsample_bytree | [0.6, 0.7, 0.8, 0.9] | 0.8 | 0.8 | 0.8 |
|  | min_child_weight | [1, 2, 3, 4, 5] | 3 | 3 | 3 |
|  | gamma | [0, 0.1, 0.2, 0.3] | 0.1 | 0.1 | 0.1 |
|  | reg_alpha | [0, 0.1, 0.5, 1.0] | 0.1 | 0.1 | 0.1 |
|  | reg_lambda | [0.5, 1.0, 1.5, 2.0] | 1 | 1 | 1 |
| SVR | kernel | ['rbf', 'poly', 'sigmoid'] | 'rbf' | 'rbf' | 'rbf' |
|  | C | [1, 10, 50, 100, 200, 500] | 1 | 1 | 1 |
|  | epsilon | [0.01, 0.05, 0.1, 0.2, 0.3] | 0.1 | 0.1 | 0.1 |
|  | gamma | ['scale', 'auto', 0.01, 0.05, 0.1, 0.5] | 'scale' | 'scale' | 'scale' |
|  | degree (poly only) | [2, 3, 4] | N/A | N/A | N/A |
| ANN | Layer 1 units | [64, 128, 256] | 128 | 128 | 128 |
|  | Layer 2 units | [32, 64, 128] | 64 | 64 | 64 |
|  | Layer 3 units | [16, 32, 64] | 32 | 32 | 32 |
|  | Layer 4 units | [8, 16, 32] | 16 | 16 | 16 |
|  | Dropout rate | [0.2, 0.3, 0.4] | 0.3 | 0.3 | 0.3 |
|  | Learning rate | [0.0005, 0.001, 0.005] | 0.001 | 0.001 | 0.001 |
|  | Batch size | [8, 16, 32] | 16 | 16 | 16 |
| LSTM | LSTM Layer 1 units | [64, 128, 256] | 128 | 128 | 128 |
|  | LSTM Layer 2 units | [32, 64, 128] | 64 | 64 | 64 |
|  | LSTM Layer 3 units | [16, 32, 64] | 32 | 32 | 32 |
|  | Dense layer units | [8, 16, 32] | 16 | 16 | 16 |
|  | Dropout rate | [0.2, 0.3, 0.4] | 0.3 | 0.3 | 0.3 |
|  | Learning rate | [0.0005, 0.001, 0.005] | 0.001 | 0.001 | 0.001 |
|  | Lookback window | [6, 12, 18, 24] | 12 | 12 | 12 |
| SARIMA | order (p,d,q) | Various combinations | Auto-selection via AICc | Auto-selection via AICc | Auto-selection via AICc |
|  | seasonal_order (P,D,Q) | Various combinations | Auto-selection via AICc | Auto-selection via AICc | Auto-selection via AICc |
|  | Seasonal Period | 12 | 12 | 12 | 12 |
|  | Search algorithm | Stepwise | Auto-selection via AICc | Auto-selection via AICc | Auto-selection via AICc |
| ETS | Error | A, M | Auto-selection via AICc | Auto-selection via AICc | Auto-selection via AICc |
|  | Trend type | N, A, M, Ad, Md | Auto-selection via AICc | Auto-selection via AICc | Auto-selection via AICc |
|  | Seasonal type | N, A, M | Auto-selection via AICc | Auto-selection via AICc | Auto-selection via AICc |
|  | Seasonal periods | 12 | 12 | 12 | 12 |
| Validation Performance |  |  |  |  |  |
| SARIMA | CV RMSE (mean ± std) | - | 5.05 ± 0.85 | 3.95 ± 0.68 | 3.54 ± 0.60 |
| ETS | CV RMSE (mean ± std) | - | 4.35 ± 0.72 | 3.84 ± 0.65 | 3.31 ± 0.55 |
| ANN | CV RMSE (mean ± std) | - | 5.50 ± 0.92 | 4.86 ± 0.82 | 3.84 ± 0.64 |
| SVR | CV RMSE (mean ± std) | - | 6.61 ± 1.10 | 5.92 ± 0.98 | 5.62 ± 0.94 |
| XGBoost | CV RMSE (mean ± std) | - | 4.12 ± 0.70 | 3.29 ± 0.55 | 3.02 ± 0.50 |
| LSTM | CV RMSE (mean ± std) | - | 8.32 ± 1.40 | 8.17 ± 1.38 | 7.46 ± 1.25 |
| X11-ARIMA | CV RMSE (mean ± std) | - | 3.71 ± 0.62 | 3.59 ± 0.60 | 2.88 ± 0.48 |
| X11-ETS | CV RMSE (mean ± std) | - | 3.45 ± 0.58 | 3.65 ± 0.61 | 2.96 ± 0.50 |
| X11-ANN | CV RMSE (mean ± std) | - | 4.27 ± 0.72 | 4.52 ± 0.76 | 4.21 ± 0.70 |
| X11-SVR | CV RMSE (mean ± std) | - | 3.54 ± 0.60 | 4.34 ± 0.73 | 2.92 ± 0.49 |
| X11-XGBoost | CV RMSE (mean ± std) | - | 3.36 ± 0.56 | 3.89 ± 0.66 | 2.77 ± 0.46 |
| X11-LSTM | CV RMSE (mean ± std) | - | 2.97 ± 0.50 | 3.76 ± 0.63 | 4.67 ± 0.78 |
| STL-ARIMA | CV RMSE (mean ± std) | - | 2.67 ± 0.45 | 3.06 ± 0.51 | 3.02 ± 0.50 |
| STL-ETS | CV RMSE (mean ± std) | - | 2.72 ± 0.46 | 3.09 ± 0.52 | 2.59 ± 0.43 |
| STL-ANN | CV RMSE (mean ± std) | - | 4.27 ± 0.72 | 3.54 ± 0.60 | 3.81 ± 0.64 |
| STL-SVR | CV RMSE (mean ± std) | - | 3.17 ± 0.53 | 2.80 ± 0.47 | 2.66 ± 0.44 |
| STL-XGBoost | CV RMSE (mean ± std) | - | 2.53 ± 0.42 | 2.09 ± 0.35 | 2.08 ± 0.35 |
| STL-LSTM | CV RMSE (mean ± std) | - | 2.89 ± 0.48 | 2.61 ± 0.44 | 2.75 ± 0.46 |

**Supplementary Table S2:** Optimal hyperparameter values for exogenous models identified through time series cross-validation with expanding window. Grid search was performed over specified ranges, with the optimal configuration selected based on average RMSE across validation windows.

| **Model** | **Hyperparameter** | **Tuning Range** | **Tuned Value (Sylhet)** | **Tuned Value (Sreemangal)** | **Tuned Value (Mymensingh)** |  |
| --- | --- | --- | --- | --- | --- | --- |
| XGBoostX | n_estimators | [100, 200, 300, 400] | 300 | 300 | 300 |  |
|  | max_depth | [3, 4, 5, 6, 7] | 6 | 6 | 6 |  |
|  | learning_rate | [0.01, 0.05, 0.1, 0.2] | 0.05 | 0.05 | 0.05 |  |
|  | subsample | [0.6, 0.7, 0.8, 0.9] | 0.8 | 0.8 | 0.8 |  |
|  | colsample_bytree | [0.6, 0.7, 0.8, 0.9] | 0.8 | 0.8 | 0.8 |  |
|  | min_child_weight | [1, 2, 3, 4, 5] | 3 | 3 | 3 |  |
|  | gamma | [0, 0.1, 0.2, 0.3] | 0.1 | 0.1 | 0.1 |  |
|  | reg_alpha | [0, 0.1, 0.5, 1.0] | 0.1 | 0.1 | 0.1 |  |
|  | reg_lambda | [0.5, 1.0, 1.5, 2.0] | 1 | 1 | 1 |  |
| SVRX | kernel | ['rbf', 'poly', 'sigmoid'] | 'rbf' | 'rbf' | 'rbf' |  |
|  | C | [1, 10, 50, 100, 200, 500] | 100 | 100 | 100 |  |
|  | epsilon | [0.01, 0.05, 0.1, 0.2, 0.3] | 0.1 | 0.1 | 0.1 |  |
|  | gamma | ['scale', 'auto', 0.01, 0.05, 0.1, 0.5] | 'scale' | 'scale' | 'scale' |  |
|  | degree (poly only) | [2, 3, 4] | N/A | N/A | N/A |  |
| ANNX | Layer 1 units | [64, 128, 256] | 128 | 128 | 128 |  |
|  | Layer 2 units | [32, 64, 128] | 64 | 64 | 64 |  |
|  | Layer 3 units | [16, 32, 64] | 32 | 32 | 32 |  |
|  | Layer 4 units | [8, 16, 32] | 16 | 16 | 16 |  |
|  | Dropout rate | [0.2, 0.3, 0.4] | 0.3 | 0.3 | 0.3 |  |
|  | Learning rate | [0.0005, 0.001, 0.005] | 0.001 | 0.001 | 0.001 |  |
|  | Batch size | [8, 16, 32] | 16 | 16 | 16 |  |
| LSTMX | LSTM Layer 1 units | [64, 128, 256] | 128 | 128 | 128 |  |
|  | LSTM Layer 2 units | [32, 64, 128] | 64 | 64 | 64 |  |
|  | LSTM Layer 3 units | [16, 32, 64] | 32 | 32 | 32 |  |
|  | Dense layer units | [8, 16, 32] | 16 | 16 | 16 |  |
|  | Dropout rate | [0.2, 0.3, 0.4] | 0.3 | 0.3 | 0.3 |  |
|  | Learning rate | [0.0005, 0.001, 0.005] | 0.001 | 0.001 | 0.001 |  |
|  | Lookback window | [6, 12, 18, 24] | 12 | 12 | 12 |  |
| SARIMAX | order (p,d,q) | Various combinations | (1,1,1) | (1,1,1) | (1,1,1) |  |
|  | seasonal_order (P,D,Q,12) | Various combinations | (0,1,1,12) | (0,1,1,12) | (0,1,1,12) |  |
|  | trend | ['n', 'c', 't', 'ct'] | 't' | n' | n' |  |
|  | Search algorithm | Stepwise | Auto-selection via AICc | Auto-selection via AICc | Auto-selection via AICc |  |
| ETSX (Two-Stage) | Stage 1: OLS Regression | Linear regression on exogenous variables | OLS | OLS | OLS |  |
|  | Stage 2: ETS on residuals |  |  |  |  |  |
|  | Error | A, M | Auto-selection via AICc | Auto-selection via AICc | Auto-selection via AICc |  |
|  | Trend type | N, A, M, Ad, Md | Auto-selection via AICc | Auto-selection via AICc | Auto-selection via AICc |  |
|  | Seasonal type | N, A, M | Auto-selection via AICc | Auto-selection via AICc | Auto-selection via AICc |  |
|  | Seasonal periods | 12 | 12 | 12 | 12 |  |
| **Validation Performance** | | | | | | |
| XGBoostX | CV RMSE (mean ± std) | - | 2.38 ± 0.42 | 2.06 ± 0.38 | 1.90 ± 0.35 |  |
| SVRX | CV RMSE (mean ± std) | - | 3.46 ± 0.58 | 2.07 ± 0.41 | 1.75 ± 0.32 |  |
| ANNX | CV RMSE (mean ± std) | - | 3.17 ± 0.51 | 2.62 ± 0.45 | 2.19 ± 0.40 |  |
| LSTMX | CV RMSE (mean ± std) | - | 4.55 ± 0.72 | 4.07 ± 0.68 | 4.24 ± 0.70 |  |
| SARIMAX | CV RMSE (mean ± std) | - | 3.72 ± 0.62 | 3.68 ± 0.60 | 3.35 ± 0.55 |  |
| ETSX | CV RMSE (mean ± std) | - | 4.57 ± 0.75 | 3.82 ± 0.64 | 3.19 ± 0.52 |  |
| STL-XGBoostX | CV RMSE (mean ± std) | - | 1.79 ± 0.31 | 1.49 ± 0.28 | 1.46 ± 0.27 |  |
| STL-SVRX | CV RMSE (mean ± std) | - | 2.13 ± 0.36 | 1.47 ± 0.26 | 1.26 ± 0.24 |  |
| STL-ANNX | CV RMSE (mean ± std) | - | 1.86 ± 0.33 | 1.88 ± 0.34 | 1.28 ± 0.25 |  |
| STL-LSTMX | CV RMSE (mean ± std) | - | 3.61 ± 0.65 | 3.37 ± 0.60 | 3.01 ± 0.54 |  |
| X11-XGBoostX | CV RMSE (mean ± std) | - | 2.20 ± 0.39 | 1.66 ± 0.30 | 1.53 ± 0.28 |  |
| X11-SVRX | CV RMSE (mean ± std) | - | 2.36 ± 0.41 | 1.60 ± 0.29 | 1.57 ± 0.29 |  |
| X11-ANNX | CV RMSE (mean ± std) | - | 2.00 ± 0.35 | 2.60 ± 0.47 | 2.98 ± 0.53 |  |

**Supplementary Table S3:** Seasonal thunderstorm frequency forecast statistics for Sylhet, Sreemangal, and Mymensingh (2025–2028) from optimal univariate and exogenous models.

| **Station** | **Season** | **Year** | **Model** | **Mean** | **Std** | **Max** | **Min** | **Total** |
| --- | --- | --- | --- | --- | --- | --- | --- | --- |
| Sylhet | Winter (DJF) | 2025 | Exogenous | 0.11 | 0.09 | 0.23 | 0.00 | 0.32 |
| Sylhet | Winter (DJF) | 2025 | Univariate | 3.05 | 1.50 | 4.99 | 1.36 | 9.14 |
| Sylhet | Winter (DJF) | 2026 | Exogenous | 0.24 | 0.27 | 0.62 | 0.00 | 0.72 |
| Sylhet | Winter (DJF) | 2026 | Univariate | 1.85 | 1.06 | 3.33 | 0.94 | 5.54 |
| Sylhet | Winter (DJF) | 2027 | Exogenous | 0.53 | 0.65 | 1.44 | 0.00 | 1.58 |
| Sylhet | Winter (DJF) | 2027 | Univariate | 2.10 | 0.89 | 3.35 | 1.37 | 6.31 |
| Sylhet | Winter (DJF) | 2028 | Exogenous | 0.04 | 0.06 | 0.13 | 0.00 | 0.13 |
| Sylhet | Winter (DJF) | 2028 | Univariate | 2.57 | 1.45 | 4.59 | 1.23 | 7.72 |
| Sylhet | Pre-monsoon (MAM) | 2025 | Exogenous | 15.87 | 11.74 | 29.03 | 0.51 | 47.60 |
| Sylhet | Pre-monsoon (MAM) | 2025 | Univariate | 13.31 | 8.25 | 22.09 | 2.27 | 39.92 |
| Sylhet | Pre-monsoon (MAM) | 2026 | Exogenous | 16.62 | 10.49 | 26.54 | 2.10 | 49.86 |
| Sylhet | Pre-monsoon (MAM) | 2026 | Univariate | 16.03 | 6.23 | 23.12 | 7.95 | 48.08 |
| Sylhet | Pre-monsoon (MAM) | 2027 | Exogenous | 19.95 | 10.61 | 27.92 | 4.95 | 59.86 |
| Sylhet | Pre-monsoon (MAM) | 2027 | Univariate | 15.25 | 3.94 | 19.84 | 10.21 | 45.75 |
| Sylhet | Pre-monsoon (MAM) | 2028 | Exogenous | 19.31 | 4.14 | 23.79 | 13.80 | 57.93 |
| Sylhet | Pre-monsoon (MAM) | 2028 | Univariate | 13.22 | 2.75 | 16.05 | 9.49 | 39.66 |
| Sylhet | Monsoon (JJAS) | 2025 | Exogenous | 23.38 | 4.65 | 27.80 | 16.09 | 93.51 |
| Sylhet | Monsoon (JJAS) | 2025 | Univariate | 18.45 | 4.28 | 22.97 | 14.12 | 73.81 |
| Sylhet | Monsoon (JJAS) | 2026 | Exogenous | 23.06 | 3.88 | 27.85 | 17.11 | 92.23 |
| Sylhet | Monsoon (JJAS) | 2026 | Univariate | 20.74 | 4.24 | 27.25 | 15.53 | 82.95 |
| Sylhet | Monsoon (JJAS) | 2027 | Exogenous | 23.72 | 5.07 | 31.03 | 17.15 | 94.89 |
| Sylhet | Monsoon (JJAS) | 2027 | Univariate | 19.23 | 4.49 | 24.49 | 12.36 | 76.92 |
| Sylhet | Monsoon (JJAS) | 2028 | Exogenous | 22.56 | 4.69 | 29.90 | 17.14 | 90.25 |
| Sylhet | Monsoon (JJAS) | 2028 | Univariate | 16.34 | 3.65 | 20.54 | 10.56 | 65.35 |
| Sylhet | Post-monsoon (ON) | 2025 | Exogenous | 2.05 | 1.95 | 4.00 | 0.10 | 4.10 |
| Sylhet | Post-monsoon (ON) | 2025 | Univariate | 6.68 | 6.20 | 12.88 | 0.48 | 13.36 |
| Sylhet | Post-monsoon (ON) | 2026 | Exogenous | 0.39 | 0.06 | 0.45 | 0.34 | 0.79 |
| Sylhet | Post-monsoon (ON) | 2026 | Univariate | 5.31 | 3.41 | 8.72 | 1.90 | 10.62 |
| Sylhet | Post-monsoon (ON) | 2027 | Exogenous | 1.37 | 0.86 | 2.24 | 0.51 | 2.75 |
| Sylhet | Post-monsoon (ON) | 2027 | Univariate | 8.27 | 7.12 | 15.38 | 1.15 | 16.53 |
| Sylhet | Post-monsoon (ON) | 2028 | Exogenous | 2.75 | 2.12 | 4.87 | 0.63 | 5.50 |
| Sylhet | Post-monsoon (ON) | 2028 | Univariate | 4.18 | 1.95 | 6.13 | 2.23 | 8.36 |
| Sreemangal | Winter (DJF) | 2025 | Exogenous | 0.07 | 0.06 | 0.14 | 0.00 | 0.21 |
| Sreemangal | Winter (DJF) | 2025 | Univariate | 2.68 | 0.25 | 3.00 | 2.40 | 8.04 |
| Sreemangal | Winter (DJF) | 2026 | Exogenous | 0.04 | 0.05 | 0.11 | 0.00 | 0.11 |
| Sreemangal | Winter (DJF) | 2026 | Univariate | 3.38 | 1.29 | 5.16 | 2.14 | 10.14 |
| Sreemangal | Winter (DJF) | 2027 | Exogenous | 0.04 | 0.06 | 0.12 | 0.00 | 0.12 |
| Sreemangal | Winter (DJF) | 2027 | Univariate | 2.52 | 0.16 | 2.73 | 2.33 | 7.56 |
| Sreemangal | Winter (DJF) | 2028 | Exogenous | 0.91 | 1.20 | 2.61 | 0.00 | 2.74 |
| Sreemangal | Winter (DJF) | 2028 | Univariate | 3.17 | 0.96 | 4.22 | 1.90 | 9.52 |
| Sreemangal | Pre-monsoon (MAM) | 2025 | Exogenous | 12.77 | 9.77 | 26.06 | 2.83 | 38.32 |
| Sreemangal | Pre-monsoon (MAM) | 2025 | Univariate | 11.64 | 6.55 | 19.44 | 3.42 | 34.93 |
| Sreemangal | Pre-monsoon (MAM) | 2026 | Exogenous | 19.80 | 13.22 | 33.68 | 2.02 | 59.41 |
| Sreemangal | Pre-monsoon (MAM) | 2026 | Univariate | 14.52 | 4.18 | 18.18 | 8.67 | 43.55 |
| Sreemangal | Pre-monsoon (MAM) | 2027 | Exogenous | 17.97 | 7.69 | 28.85 | 12.42 | 53.91 |
| Sreemangal | Pre-monsoon (MAM) | 2027 | Univariate | 11.49 | 3.52 | 16.21 | 7.75 | 34.46 |
| Sreemangal | Pre-monsoon (MAM) | 2028 | Exogenous | 16.70 | 8.32 | 22.84 | 4.93 | 50.09 |
| Sreemangal | Pre-monsoon (MAM) | 2028 | Univariate | 14.62 | 5.75 | 20.72 | 6.92 | 43.87 |
| Sreemangal | Monsoon (JJAS) | 2025 | Exogenous | 23.00 | 2.92 | 26.69 | 19.12 | 91.99 |
| Sreemangal | Monsoon (JJAS) | 2025 | Univariate | 18.61 | 2.04 | 20.86 | 15.44 | 74.44 |
| Sreemangal | Monsoon (JJAS) | 2026 | Exogenous | 25.13 | 2.20 | 28.42 | 22.35 | 100.52 |
| Sreemangal | Monsoon (JJAS) | 2026 | Univariate | 18.41 | 4.60 | 26.31 | 15.15 | 73.65 |
| Sreemangal | Monsoon (JJAS) | 2027 | Exogenous | 20.41 | 3.52 | 24.31 | 15.30 | 81.64 |
| Sreemangal | Monsoon (JJAS) | 2027 | Univariate | 19.20 | 2.88 | 23.71 | 16.19 | 76.81 |
| Sreemangal | Monsoon (JJAS) | 2028 | Exogenous | 21.30 | 5.85 | 28.84 | 13.44 | 85.22 |
| Sreemangal | Monsoon (JJAS) | 2028 | Univariate | 17.48 | 2.11 | 19.46 | 13.99 | 69.90 |
| Sreemangal | Post-monsoon (ON) | 2025 | Exogenous | 3.39 | 3.26 | 6.65 | 0.13 | 6.78 |
| Sreemangal | Post-monsoon (ON) | 2025 | Univariate | 7.65 | 5.87 | 13.52 | 1.78 | 15.30 |
| Sreemangal | Post-monsoon (ON) | 2026 | Exogenous | 5.65 | 5.42 | 11.06 | 0.23 | 11.29 |
| Sreemangal | Post-monsoon (ON) | 2026 | Univariate | 6.36 | 3.56 | 9.93 | 2.80 | 12.73 |
| Sreemangal | Post-monsoon (ON) | 2027 | Exogenous | 3.70 | 3.43 | 7.13 | 0.27 | 7.40 |
| Sreemangal | Post-monsoon (ON) | 2027 | Univariate | 7.84 | 5.94 | 13.78 | 1.91 | 15.69 |
| Sreemangal | Post-monsoon (ON) | 2028 | Exogenous | 2.72 | 2.28 | 5.00 | 0.44 | 5.44 |
| Sreemangal | Post-monsoon (ON) | 2028 | Univariate | 4.83 | 2.47 | 7.31 | 2.36 | 9.67 |
| Mymensingh | Winter (DJF) | 2025 | Exogenous | 0.43 | 0.52 | 1.16 | 0.00 | 1.28 |
| Mymensingh | Winter (DJF) | 2025 | Univariate | 0.52 | 0.37 | 0.85 | 0.00 | 1.57 |
| Mymensingh | Winter (DJF) | 2026 | Exogenous | 0.05 | 0.07 | 0.15 | 0.00 | 0.15 |
| Mymensingh | Winter (DJF) | 2026 | Univariate | 2.45 | 0.59 | 3.24 | 1.83 | 7.36 |
| Mymensingh | Winter (DJF) | 2027 | Exogenous | 0.06 | 0.08 | 0.17 | 0.00 | 0.17 |
| Mymensingh | Winter (DJF) | 2027 | Univariate | 2.28 | 0.18 | 2.43 | 2.03 | 6.85 |
| Mymensingh | Winter (DJF) | 2028 | Exogenous | 0.06 | 0.09 | 0.19 | 0.00 | 0.19 |
| Mymensingh | Winter (DJF) | 2028 | Univariate | 2.63 | 0.18 | 2.78 | 2.37 | 7.88 |
| Mymensingh | Pre-monsoon (MAM) | 2025 | Exogenous | 12.36 | 7.66 | 20.59 | 2.14 | 37.07 |
| Mymensingh | Pre-monsoon (MAM) | 2025 | Univariate | 12.82 | 6.92 | 21.11 | 4.17 | 38.47 |
| Mymensingh | Pre-monsoon (MAM) | 2026 | Exogenous | 13.37 | 6.82 | 19.57 | 3.88 | 40.10 |
| Mymensingh | Pre-monsoon (MAM) | 2026 | Univariate | 11.89 | 5.71 | 17.81 | 4.19 | 35.68 |
| Mymensingh | Pre-monsoon (MAM) | 2027 | Exogenous | 8.09 | 7.45 | 18.38 | 1.01 | 24.27 |
| Mymensingh | Pre-monsoon (MAM) | 2027 | Univariate | 9.51 | 5.89 | 17.70 | 4.10 | 28.54 |
| Mymensingh | Pre-monsoon (MAM) | 2028 | Exogenous | 10.20 | 4.65 | 15.40 | 4.12 | 30.61 |
| Mymensingh | Pre-monsoon (MAM) | 2028 | Univariate | 12.10 | 2.48 | 15.57 | 9.91 | 36.30 |
| Mymensingh | Monsoon (JJAS) | 2025 | Exogenous | 16.84 | 9.08 | 31.49 | 6.79 | 67.35 |
| Mymensingh | Monsoon (JJAS) | 2025 | Univariate | 15.71 | 1.97 | 18.94 | 13.67 | 62.85 |
| Mymensingh | Monsoon (JJAS) | 2026 | Exogenous | 18.19 | 5.32 | 25.39 | 11.77 | 72.78 |
| Mymensingh | Monsoon (JJAS) | 2026 | Univariate | 13.66 | 2.31 | 16.91 | 10.77 | 54.64 |
| Mymensingh | Monsoon (JJAS) | 2027 | Exogenous | 17.21 | 5.33 | 23.78 | 11.71 | 68.83 |
| Mymensingh | Monsoon (JJAS) | 2027 | Univariate | 17.34 | 3.58 | 22.42 | 13.20 | 69.35 |
| Mymensingh | Monsoon (JJAS) | 2028 | Exogenous | 18.03 | 4.06 | 22.86 | 13.49 | 72.13 |
| Mymensingh | Monsoon (JJAS) | 2028 | Univariate | 15.50 | 0.52 | 16.29 | 14.95 | 62.00 |
| Mymensingh | Post-monsoon (ON) | 2025 | Exogenous | 2.28 | 2.14 | 4.43 | 0.14 | 4.57 |
| Mymensingh | Post-monsoon (ON) | 2025 | Univariate | 3.37 | 2.57 | 5.95 | 0.80 | 6.75 |
| Mymensingh | Post-monsoon (ON) | 2026 | Exogenous | 0.33 | 0.07 | 0.41 | 0.26 | 0.67 |
| Mymensingh | Post-monsoon (ON) | 2026 | Univariate | 6.06 | 4.33 | 10.39 | 1.73 | 12.13 |
| Mymensingh | Post-monsoon (ON) | 2027 | Exogenous | 1.50 | 1.19 | 2.69 | 0.31 | 3.00 |
| Mymensingh | Post-monsoon (ON) | 2027 | Univariate | 5.92 | 3.02 | 8.95 | 2.90 | 11.85 |
| Mymensingh | Post-monsoon (ON) | 2028 | Exogenous | 3.52 | 3.05 | 6.57 | 0.47 | 7.04 |
| Mymensingh | Post-monsoon (ON) | 2028 | Univariate | 7.72 | 4.71 | 12.43 | 3.01 | 15.44 |
