# Supplementary material for: Station-specific thunderstorm forecasting using climate predictors with seasonal decomposition analysis in Bangladesh’s most vulnerable regions
Source: PLoS One. 2026 Sep 24;21(9):e0358795. doi: 10.1371/journal.pone.0358795 (PMC13607998; doi:10.1371/journal.pone.0358795)
Supplement: Supplementary file 8 [file pone.0358795.s006.docx]

Dear Editor,

I hope this message finds you well. I extend my sincere appreciation for providing me with the opportunity to review the manuscript. While the manuscript has undergone previous comments but there are still some modifications and clarifications that require attention. I have outlined these issues below for your consideration:

1. The study area is not well described. The study area section should provide a clear description of the geographical, environmental, climatic, and socio-economic characteristics of the study area, supported by relevant maps or figures. So that, reader can get a clear picture and idea about your research region. The authors should improve it and see other published papers for a good understanding.
2. The authors can rename the study area to “study area and data.” Give a brief description of your dataset (like stations, data resolutions, temporal resolutions, etc.).
3. Lines 161 – 164 are irrelevant in the study area section.
4. The conclusion does not represent the key findings of this research. I would encourage the authors to improve the conclusion section and explain how the findings would be helpful for the decision makers.
