# Supplementary material for: Station-specific thunderstorm forecasting using climate predictors with seasonal decomposition analysis in Bangladesh’s most vulnerable regions
Source: PLoS One. 2026 Sep 24;21(9):e0358795. doi: 10.1371/journal.pone.0358795 (PMC13607998; doi:10.1371/journal.pone.0358795)
Supplement: Supplementary file 9 [file pone.0358795.s007.docx]

Title: Climate Change and Seasonal Thunderstorm Intensification: Evidence from Bangladesh’s Most Vulnerable Regions

In this manuscript, the authors investigated the yearly and monthly variation in the frequency of thunderstorms (TS) in 3 cities of Bangladesh. They also developed 6 statistical models for forecasting monthly TS frequency during 2025-2028. The result found that the model with seasonally adjusted data obtained the best performance. Unfortunately, the manuscript contains several vital issues, especially the lack of explanation about how the models are trained. A lot of analyses are redundant and not well linked to each other. As a result, the manuscript in the current form cannot be published unless the quality of the study and manuscript is significantly improved, which should include a redesign of the study to make the focus (TS forecasting) clearer.

The major comments:

1. As explained in the introduction, the research gap is “high-resolution, localized forecasting models that account for the unique climatology of Bangladesh's most vulnerable regions.” However, only 2 of the 10 figures plus one table are related to the forecasting models. The relevant content is disproportionately insufficient. Moreover, the remaining 8 figures do not have any supporting role to pave the way for the development of the model. The analyses associated with Figs. 1-8 are the background information. If the authors insist on the same research gap, the manuscript should be reorganized to emphasize on the building, training, and evaluation of the models, and minimize irrelevant contents (including the figures and analyses).
2. Since the objective is to develop models for better forecasting of TS, the introduction does not contain relevant background information related to the topic. The first 4 paragraphs are simply generic information about TS. The authors should introduce the challenges of forecasting of monthly TS frequency in Bangladesh.
3. The authors spend a lot of time on explaining the annual cycle of the number of thunderstorm (Figs. 3-8). However, it is neither new or hidden knowledge. This information can be found in https://www.banglajol.info/index.php/BJPhy/article/view/78684, which is published in 2022. Only very little can be regarded as new information. The redundancy is excessive even if removing the seasonal variable from the training data of models is a key aspect of this study.
4. For machine learning models, it is important to describe how these models are trained. It should include the split of training, validation, and test dataset, hyperparameters, etc. The training and evaluation of ML models have profound impact of the performance of the models. However, these information are entirely missing in the manuscript. The authors must clearly describe the training and evaluation procedure of each model.
5. Because the structure, setting, and training data allocation of the machine learning and deep learning model are entirely unknown, it is impossible to know whether these models are well trained or overfitted. Although LSTM is a powerful deep learning algorithm, if it is poorly designed and insufficiently training, it is not impossible for it to get a lower score than any simple models. Therefore, it is impossible to give a fair and meaningful model evaluation.
6. The description and interpretation of all analyses and figures are insufficient. For example, in Fig. 2, only the earlier part of the time series is described, and the later part is completely ignored. I don’t know whether the authors found the later part of the time series not interesting. There is also no comparison between the results of the 3 cities. Since the 3 cities are neigboring cities, it is possible to analyze the average of them, instead of analyze them individually, especially there are not much difference in .
7. The author should explain why the seasonally adjusted data lead to better model performance.
8. The title does not align with the content. The study is clearly not exmaining climate change, although there are a few yearly time series; TS does not show intensification trend as well. The authors should write a title that trufully reflect the result of the study.
9. Figure 2: The figure is inappropriate. Quantity with different units should not share the same y-axis. The variation in relative humidity and average temperature cannot be observed due to the overly large maximum value in y. Please include only one variable in one panel.
10. Figure 2: In Sylhet, there is an observable decreasing trend since 2010. For Sreemangal, the decreasing trend is weaker than Sylhet, but the one for Mymensingh is a little bit clearer. These are important findings in the figure, but the authors omit them.
11. L355: The decreasing trend in TS frequency is obvious in the 3 cities. The authors must clearly explain how they can attribute climate change to such a decreasing trend.
12. L380-381: This statement is a purely qualitative observation. The authors need to connect it to the decreasing trend of the number of TS observed in Fig. 2.
13. The function of boxplots in Figs 4-6 overlap with Fig. 2. To reduce information redundancy, the authors should keep either boxplots of line plots. Also, the authors should also describe the difference in result between the 3 cities. Moreover, Figs 4-6 should be merged into one figure.
14. Figs 4-6: Similar to Fig 2, the monthly TS count in the 3 cities show a clear decreasing trend in the recent decade.
15. For model evaluation, the authors should not simply assess the average of all samples. More statistics should be utilized.
16. L513-518: The authors should explain why there is a discrepancy in the monsoon season definition between your result and the conventional climatological framework. Does the conventional one represent better at national level rather than city level?

Minor comments:

17. L69-70: "Damage" should not be used for "human health". “Leading to …….and risk to human health” is more appropriate. Moreover, it does not seems there is a direct relationship between thunderstorms and human health. Please add references as evidence for such a relationship.
18. The first 2 paragraphs have common topic: damage. They should be combined into one.
19. L78: Remove the word “tragic”.
20. L110-111: This paragraph is all about forecasting. However, the studies of the 2 references (28, 29) only used WRF for studying thunderstorms, but not forecasting them. These 2 references are not suitable for this paragraph. Please remove them.
21. L122: What is the forecast lead time? Days or hours?
22. L140: This subsection is actually explaining your dataset, and is irrelevant to the study domain. Please describe why you select the 3 cities and exclude the others in more details to justify your choice. Also, write a subsection specifically for describing your dataset.
23. It is difficult to distinguish the level of subsection as the number for indicating sub-section is missing. Please assign subsection number.
24. Figure 1: The font of city name are too small to be read easily. Please enlarge them.
25. L147-148: Please describe how TS events is counted in this dataset.
26. L152-164: This paragraph will be clearer if you first explain why you use the ground-based dataset, and then why you don't use the satellite dataset..
27. L163-164: This sentence is unnecessary and inappropriate for this section. Please remove.
28. L184-185: Machine learning approach is statistical in nature, so it is correct to frame all these 6 techniques as statistical methods.
29. L188: Abbreviation has been defined earlier on L176. It is unnecessary to write the term in full. The same issue exists for the subsection of all models. Please correct it.
30. L219-220: The sentence "Click or tap here to enter text" appears too many times. The authors should make sure unnecessary text are removed before submission.
31. L276-298: I suggest the authors include figures to demonstrate a few time series before and after processed by the two decomposition method. It is the best way to show the readers the difference between them, and how they work.
32. L327-329: These are information for study area. Please move it to Section 2.
33. L337-338: Although you cited a reference, it is necessary to explicitly explain why large rainfall amount can help severe storm development. It does not sound like a proper casual relationship.
34. L356: Figure 2 only shows annual value, seasonal dynamics cannot be observed. Please correct this sentence.
35. Figure 3: The x-axis should be month instead of season.
36. L406: How is the trend calculated? By moving average? If yes, it is more appropriate to call it "X-year moving average" because "trend" is more specific, usually linear.
37. Figure 8: Ridgeline density plot is not suitable when data contains very small number of samples (those winter months). Also, TS count is always positive, so the the part in the negative region is non-physical. The authors must trim it to avoid misleading the readers. Furthermore, what is the value associated with each radius in the plot?
38. Table 1: Besides showing a table, I suggest the authors to visualize the model performance using barchart. It can make the comparison of each model easier.
39. L447: What is the data period for model evaluation?
40. L450: ANN is consider as deep learning as long as the network contains at least one hidden layer. Please revise.
41. L483: What is the input variables (including lag time) and forecast lead time of the output of the model? These are important information.
42. L495-509: This paragraph describes the background of this study, so it should belong to the Introduction section.
