# Supplementary material for: Station-specific thunderstorm forecasting using climate predictors with seasonal decomposition analysis in Bangladesh’s most vulnerable regions
Source: PLoS One. 2026 Sep 24;21(9):e0358795. doi: 10.1371/journal.pone.0358795 (PMC13607998; doi:10.1371/journal.pone.0358795)
Supplement: Supplementary file 10 [file pone.0358795.s008.doc]

**1. Overall Significance**

- The manuscript addresses an important and timely topic: the intensification of thunderstorms (TS) in Bangladesh under climate change, which has major societal and economic implications.
- The use of **station-level forecasting with seasonal adjustment and machine learning** is innovative compared to many prior works relying on pooled or regional averages.
- However, the paper would benefit from clearer articulation of the *novelty*: is the innovation primarily methodological (STL-XGBoost hybrid), geographic focus (Bangladesh), or policy relevance (early warning systems)?

**2. Abstract and Introduction**

- The abstract is well-structured but somewhat **dense**. Consider simplifying technical details (e.g., naming fewer models in the abstract) and emphasizing the **key policy relevance** more strongly.
- The introduction reviews global and local literature but could expand discussion of:
  - Why thunderstorms specifically (vs. cyclones/floods) have been understudied in Bangladesh.
  - The unique socio-economic vulnerability of Sylhet, Sreemangal, and Mymensingh beyond climatology (e.g., agriculture dependence, lightning mortality).
  - How this study directly advances or contrasts with prior works using SARIMA/ARIMA in Bangladesh

**3. Methods**

- The **dataset description** (1985–2024, BMD station data) is clear. Still:
  - Clarify **data quality control**: Were missing values imputed? Were homogenization checks performed?
  - Explain whether lightning-day definitions or storm thresholds were consistent over the 40 years.
- The **seasonal adjustment section** is strong (X-11 vs STL), but more justification of **why STL was expected to perform better** would help (currently explained mostly post-results).
- The **model comparison**:
  - Six forecasting models are compared (ARIMA, ETS, ANN, SVR, XG-Boost, LSTM). However, LSTM performed very poorly (MSE > 100). Why? Was hyperparameter tuning sufficient? Could this be due to data scarcity?
  - Were cross-validation or out-of-sample tests done, or only in-sample error reporting? Clarify.
  - Report computational efficiency – were some models more resource-intensive, which could matter for operational use by BMD?

**4. Results**

- Results are clearly presented with detailed tables of MSE, RMSE, and MAE across stations.
- STL-XG-Boost consistently outperforms, but the **margins of improvement** over STL-SVR or STL-ETS could be better highlighted numerically (e.g., % gain).
- Figures:
  - Figure 2 (stacked bars of temperature, humidity, and TS counts) is visually overloaded. Consider splitting variables or using line+bar overlays for clarity.
  - Forecast plots (2025–2028, Figure 9) are strong but could include **confidence intervals** to communicate uncertainty.
  - Radar plot (Figure 10) is informative but unusual in meteorology—justify its use or replace with boxplots/seasonal profiles.

**5. Discussion**

- Discussion appropriately situates results in the climate change context, but could improve by:
  - Explicitly quantifying the **policy implications** (e.g., how much earlier warnings could be issued, or which adaptation strategies might be prioritised).
  - Linking to **regional/global thunderstorm studies** (India, China, Nepal) more directly to highlight comparative insights.
  - Addressing **limitations**: reliance on only 3 stations, no incorporation of reanalysis/satellite data, and lack of socio-economic outcome modelling.

**6. References**

- The reference list is comprehensive, covering climatology, forecasting, and machine learning.
- Some sources (blogs/tutorials like Analytics Vidhya) may not be acceptable for a high-impact journal—consider replacing with peer-reviewed methodological references.
- Add more citations on lightning mortality in Bangladesh and regional ENSO/IOD linkages.

**7. Language and Style**

- Generally clear, but some sections are overly **technical for a generalist journal like PLOS ONE**. Recommend:
  - Shortening methodological jargon in the abstract/introduction.
  - Ensuring figures and captions are self-contained.
  - Using consistent terminology (e.g., TS = thunderstorm vs TS days vs thunderstorm frequency).

**Summary Recommendation**

- **Major revision** recommended. The study is important, but revisions are needed in **clarifying novelty, improving data transparency, justifying modelling choices, and refining figures/policy discussion**.
- With these addressed, the manuscript could make a meaningful contribution to climate resilience and forecasting in South Asia.
