# Supplementary material for: Station-specific thunderstorm forecasting using climate predictors with seasonal decomposition analysis in Bangladesh’s most vulnerable regions
Source: PLoS One. 2026 Sep 24;21(9):e0358795. doi: 10.1371/journal.pone.0358795 (PMC13607998; doi:10.1371/journal.pone.0358795)
Supplement: Peer review history

# Peer review: Decision Letter 0

**DOI:** 10.1371/journal.pone.0358795.r001

**Authors:** Rajib Chowdhury

8 Jul 2025

Dear Dr. Rois,

Thank you for submitting your manuscript to PLOS ONE. After careful consideration, we feel that it has merit but does not fully meet PLOS ONE’s publication criteria as it currently stands. Therefore, we invite you to submit a revised version of the manuscript that addresses the points raised during the review process.

Please submit your revised manuscript by Aug 22 2025 11:59PM. If you will need more time than this to complete your revisions, please reply to this message or contact the journal office at plosone@plos.org. When you're ready to submit your revision, log on to https://www.editorialmanager.com/pone/ and select the 'Submissions Needing Revision' folder to locate your manuscript file.

- A rebuttal letter that responds to each point raised by the academic editor and reviewer(s). You should upload this letter as a separate file labeled 'Response to Reviewers'.
- A marked-up copy of your manuscript that highlights changes made to the original version. You should upload this as a separate file labeled 'Revised Manuscript with Track Changes'.
- An unmarked version of your revised paper without tracked changes. You should upload this as a separate file labeled 'Manuscript'.

If you would like to make changes to your financial disclosure, please include your updated statement in your cover letter. Guidelines for resubmitting your figure files are available below the reviewer comments at the end of this letter.

If applicable, we recommend that you deposit your laboratory protocols in protocols.io to enhance the reproducibility of your results. Protocols.io assigns your protocol its own identifier (DOI) so that it can be cited independently in the future. For instructions see: https://journals.plos.org/plosone/s/submission-guidelines#loc-laboratory-protocols. Additionally, PLOS ONE offers an option for publishing peer-reviewed Lab Protocol articles, which describe protocols hosted on protocols.io. Read more information on sharing protocols at https://plos.org/protocols?utm_medium=editorial-email&utm_source=authorletters&utm_campaign=protocols.

We look forward to receiving your revised manuscript.

Kind regards,

Rajib Chowdhury, M.Sc.; MPH

Academic Editor

PLOS ONE

Journal Requirements:

When submitting your revision, we need you to address these additional requirements.

1. Please ensure that your manuscript meets PLOS ONE's style requirements, including those for file naming. The PLOS ONE style templates can be found at https://journals.plos.org/plosone/s/file?id=wjVg/PLOSOne_formatting_sample_main_body.pdf and https://journals.plos.org/plosone/s/file?id=ba62/PLOSOne_formatting_sample_title_authors_affiliations.pdf

2. Please note that PLOS One has specific guidelines on code sharing for submissions in which author-generated code underpins the findings in the manuscript. In these cases, we expect all author-generated code to be made available without restrictions upon publication of the work. Please review our guidelines at https://journals.plos.org/plosone/s/materials-and-software-sharing#loc-sharing-code and ensure that your code is shared in a way that follows best practice and facilitates reproducibility and reuse.

3. When completing the data availability statement of the submission form, you indicated that you will make your data available on acceptance. We strongly recommend all authors decide on a data sharing plan before acceptance, as the process can be lengthy and hold up publication timelines. Please note that, though access restrictions are acceptable now, your entire data will need to be made freely accessible if your manuscript is accepted for publication. This policy applies to all data except where public deposition would breach compliance with the protocol approved by your research ethics board. If you are unable to adhere to our open data policy, please kindly revise your statement to explain your reasoning and we will seek the editor's input on an exemption. Please be assured that, once you have provided your new statement, the assessment of your exemption will not hold up the peer review process.

4. We note that you have indicated that there are restrictions to data sharing for this study. For studies involving human research participant data or other sensitive data, we encourage authors to share de-identified or anonymized data. However, when data cannot be publicly shared for ethical reasons, we allow authors to make their data sets available upon request. For information on unacceptable data access restrictions, please see http://journals.plos.org/plosone/s/data-availability#loc-unacceptable-data-access-restrictions.

Before we proceed with your manuscript, please address the following prompts:

a) If there are ethical or legal restrictions on sharing a de-identified data set, please explain them in detail (e.g., data contain potentially identifying or sensitive patient information, data are owned by a third-party organization, etc.) and who has imposed them (e.g., a Research Ethics Committee or Institutional Review Board, etc.). Please also provide contact information for a data access committee, ethics committee, or other institutional body to which data requests may be sent.

b) If there are no restrictions, please upload the minimal anonymized data set necessary to replicate your study findings to a stable, public repository and provide us with the relevant URLs, DOIs, or accession numbers. Please see http://www.bmj.com/content/340/bmj.c181.long for guidelines on how to de-identify and prepare clinical data for publication. For a list of recommended repositories, please see https://journals.plos.org/plosone/s/recommended-repositories. You also have the option of uploading the data as Supporting Information files, but we would recommend depositing data directly to a data repository if possible.

Please update your Data Availability statement in the submission form accordingly.

5. Please amend your list of authors on the manuscript to ensure that each author is linked to an affiliation. Authors’ affiliations should reflect the institution where the work was done (if authors moved subsequently, you can also list the new affiliation stating “current affiliation:….” as necessary).

6. We note that Figure 1 in your submission contain [map/satellite] images which may be copyrighted. All PLOS content is published under the Creative Commons Attribution License (CC BY 4.0), which means that the manuscript, images, and Supporting Information files will be freely available online, and any third party is permitted to access, download, copy, distribute, and use these materials in any way, even commercially, with proper attribution. For these reasons, we cannot publish previously copyrighted maps or satellite images created using proprietary data, such as Google software (Google Maps, Street View, and Earth). For more information, see our copyright guidelines: http://journals.plos.org/plosone/s/licenses-and-copyright.

We require you to either (1) present written permission from the copyright holder to publish these figures specifically under the CC BY 4.0 license, or (2) remove the figures from your submission:

a. You may seek permission from the original copyright holder of Figure 1 to publish the content specifically under the CC BY 4.0 license.

We recommend that you contact the original copyright holder with the Content Permission Form (http://journals.plos.org/plosone/s/file?id=7c09/content-permission-form.pdf) and the following text:

“I request permission for the open-access journal PLOS ONE to publish XXX under the Creative Commons Attribution License (CCAL) CC BY 4.0 (http://creativecommons.org/licenses/by/4.0/). Please be aware that this license allows unrestricted use and distribution, even commercially, by third parties. Please reply and provide explicit written permission to publish XXX under a CC BY license and complete the attached form.”

Please upload the completed Content Permission Form or other proof of granted permissions as an "Other" file with your submission.

In the figure caption of the copyrighted figure, please include the following text: “Reprinted from [ref] under a CC BY license, with permission from [name of publisher], original copyright [original copyright year].”

b. If you are unable to obtain permission from the original copyright holder to publish these figures under the CC BY 4.0 license or if the copyright holder’s requirements are incompatible with the CC BY 4.0 license, please either i) remove the figure or ii) supply a replacement figure that complies with the CC BY 4.0 license. Please check copyright information on all replacement figures and update the figure caption with source information. If applicable, please specify in the figure caption text when a figure is similar but not identical to the original image and is therefore for illustrative purposes only.

The following resources for replacing copyrighted map figures may be helpful:

USGS National Map Viewer (public domain): http://viewer.nationalmap.gov/viewer/

The Gateway to Astronaut Photography of Earth (public domain): http://eol.jsc.nasa.gov/sseop/clickmap/

Maps at the CIA (public domain): https://www.cia.gov/library/publications/the-world-factbook/index.html and https://www.cia.gov/library/publications/cia-maps-publications/index.html

NASA Earth Observatory (public domain): http://earthobservatory.nasa.gov/

Landsat: http://landsat.visibleearth.nasa.gov/

USGS EROS (Earth Resources Observatory and Science (EROS) Center) (public domain): http://eros.usgs.gov/#

Natural Earth (public domain): http://www.naturalearthdata.com/

[Note: HTML markup is below. Please do not edit.]

Reviewers' comments:

Reviewer's Responses to Questions

**Comments to the Author**

1. Is the manuscript technically sound, and do the data support the conclusions?

Reviewer #1: Yes

Reviewer #2: Yes

**********

2. Has the statistical analysis been performed appropriately and rigorously?

Reviewer #1: Yes

Reviewer #2: Yes

**********

3. Have the authors made all data underlying the findings in their manuscript fully available?

The PLOS Data policy

Reviewer #1: Yes

Reviewer #2: Yes

**********

4. Is the manuscript presented in an intelligible fashion and written in standard English?

Reviewer #1: Yes

Reviewer #2: Yes

**********

Reviewer #1: Abstract:

The abstract should clearly summarize the research manuscript’s conclusions. It is currently too

general. It should specify the research findings with numerical statistics (if any).

Introduction:

1. Which months are the pre-monsoon and monsoon season? It is not mentioned in the

introduction.

2. Please improvise on the introduction section. It does not address recent research studies

that could be relevant.

Materials and Methods:

1. Figure 1 is not ideal for the journal paper. In the figure, there is no scale bar, coordinate,

and the resolution is too low.

2. Revise the section on the study area and data collection. It seems more relevant to the

results section rather than the study area and data collection part. For your example, you

can see this paper: ‘GIS-based spatial analysis for lightning scenario in Bangladesh’.

3. To model and forecast the monthly frequency of TS days in high-risk locations of

Bangladesh specifically, Sylhet, Sreemangal, and Mymensingh, this study employs a

diverse set of forecasting techniques, encompassing both traditional time series models

and modern machine learning approaches – irrelevant in the methods section.

4. ARIMA Model – add citation.

5. In the methods section, better write the full form of the heading of all the model names.

6. Evaluation Metrics – use citation.

7. The datasets have not been compared with other datasets, such as the ISS-LIS dataset.

Please provide justification for not using satellite-based data.

Results:

1. Data description is not part of the result. Revise it.

2. Why are Sylhet, Sreemangal, and Mymensingh the most TS-prone regions in

Bangladesh? Provide further reasons.

3. Why does Sylhet have the most thunderstorms?

4. The result part is not well structured and described. The findings should be described in

the results section very clearly.

Discussion:

1. For Bangladesh, why is the pre-monsoon season only April to May, and also the monsoon

season (June – August)? As per the climatic season, March – June is Pre-monsoon and

June – September is the Monsoon season.

Conclusion:

This manuscript has no conclusion section.

The authors should maintain a smoother manuscript flow of writing and coherence of each

section. In addition, the authors have not explained the reasons for TS occurrences in the specific

areas. The current level of description is not deep enough. This manuscript has no page and line

numbers, making it difficult for a reviewer to review.

Reviewer #2: Climate Change and Seasonal Thunderstorm Intensification: Evidence from Bangladesh’s Most Vulnerable Regions

The increasing frequency and severity of thunderstorms (TS) in Bangladesh—particularly in regions such as Sylhet, Sreemangal, and Mymensingh—reflects evolving climate variability and associated atmospheric dynamics. This study investigates monthly TS incidence between 1985 and 2024 to characterize temporal distributions and construct reliable predictive frameworks. A marked seasonal pattern emerges, with the highest concentration of events recorded during the pre-monsoon months (April–May) and the monsoon season (June–August). Among the three regions, Sylhet exhibits the most persistent TS activity, followed by Sreemangal and Mymensingh. To forecast future occurrences, six modeling techniques were employed: conventional time series approaches like Autoregressive Integrated Moving Average (ARIMA) and Exponential Smoothing (ETS), alongside advanced machine learning algorithms including Artificial Neural Networks (ANN), Support Vector Regression (SVR), Extreme Gradient Boosting (XGBoost), and Long Short-Term Memory (LSTM) networks. XGBoost demonstrated the highest forecasting performance across all locations. Incorporating seasonal decomposition techniques—specifically X-11 and Seasonal-Trend decomposition using Loess (STL)—further enhanced model precision, with STL adjustments yielding the most significant improvements. The hybrid STL-XGBoost model reduced mean squared error (MSE) by over 65% compared to baseline models without seasonal correction. Projections extending to 2028 reinforce the persistence of seasonal peaks, particularly in Sylhet, which is expected to remain the most TS-affected region. These results underscore the necessity of region-specific early warning mechanisms and the development of climate-resilient infrastructure. The insights derived from this analysis are relevant for operational use by the Bangladesh Meteorological Department (BMD) and may inform national disaster preparedness frameworks. Future work should incorporate high-resolution spatial datasets, satellite-based observations, and explore the influence of broader climatic drivers. Multidisciplinary engagement will be critical to translating these findings into effective adaptation and risk mitigation policies for a more resilient Bangladesh.

Introduction: More accurate and updated references needed and are available. I am not commenting on each reference. We should provide more matching references and credit should go to those really contributed to specific knowledge domain. Background information of this paper is much off the way of its main two research objectives. More accurate and updated context and references about thunderstorm characteristics and impacts should be provided

1. Rahman, Md. M., Shobuj, I. A., Santo, Md. M. H., Ali, H. M., Hossain, Md. T., Alam, E., & Islam, Md. K. (2025). Knowledge, attitude, and practices towards lightning among vulnerable group: A case study on Agri farmers and fishermen. Sustainable Environment, 11(1). https://doi.org/10.1080/27658511.2025.2464373

2. Akter, M.Y., Islam, A.R.M., Mallick, J., Alam, E., 2024. Temperature extremes Projections over Bangladesh from CMIP6 Multi-model Ensemble. Theor Appl Climatol.. https://doi.org/10.1007/s00704-024-05173-5

3. Rahman, M.M., Mannan, M.A., Sarkar, M.S.K., Sultana, A., Islam, M.M., Akter, M.Y., Alam, E., and Islam, A.R.M.T. 2024. Are hotspots and frequencies of heatwave changing over time? Exploring causes of heatwaves in a tropical country. Plos One, 19 (5): e0300070. https://doi.org/10.1371/journal.pone.0300070

4. Islam, A.R.M.T, Hasanuzzaman, M., Jaman, M., Alam, E., Mallick, J., Alam, G.M.M, Sattar, M.A., Techato, K., 2021. Assessing farmers perceived typologies concerning environmental issues and climate change for sustainable adaptation strategies in Bangladesh, Climate Journal. Xx. Doi: org/10.3390/cli9120167

Data used and methodology: Methods are substantial.

A step by step wise data collection, data cleaning and analysis variables, and processes may enhance method section.

Results: The results are clearly presented.

Discussions: This section is substantial.

Conclusion: It is not clear from the analysis how their findings are new and novel from current understanding of the relevant science and how they are contributing to improve current state of art in the discipline. I would encourage to write how the policy sciences can be improved from the results presented in the discipline.

**********

what does this mean?). If published, this will include your full peer review and any attached files.

If you choose “no”, your identity will remain anonymous but your review may still be made public.

**Do you want your identity to be public for this peer review?**  For information about this choice, including consent withdrawal, please see our Privacy Policy

Reviewer #1: No

Reviewer #2: **Yes:** Edris Alam

**********

[NOTE: If reviewer comments were submitted as an attachment file, they will be attached to this email and accessible via the submission site. Please log into your account, locate the manuscript record, and check for the action link "View Attachments". If this link does not appear, there are no attachment files.]

While revising your submission, please upload your figure files to the Preflight Analysis and Conversion Engine (PACE) digital diagnostic tool, https://pacev2.apexcovantage.com/. PACE helps ensure that figures meet PLOS requirements. To use PACE, you must first register as a user. Registration is free. Then, login and navigate to the UPLOAD tab, where you will find detailed instructions on how to use the tool. If you encounter any issues or have any questions when using PACE, please email PLOS at figures@plos.org. Please note that Supporting Information files do not need this step.

Attachment Submitted filename: Review Report.pdf

# Author comment: Author response to Decision Letter 1

**DOI:** 10.1371/journal.pone.0358795.r002

6 Sep 2025

Dear Editor,

We sincerely thank you and the reviewers for your time and thoughtful comments on our manuscript. We appreciate the opportunity to revise our work and have carefully addressed each concern to enhance the scientific quality, clarity, and structure of the manuscript. Below, we provide a detailed, point-by-point response to all the editorial and reviewer comments.

Best regards,

Authors

Editorial Requirements

When submitting your revision, we need you to address these additional requirements.

1. Please ensure that your manuscript meets PLOS ONE's style requirements, including those for file naming. The PLOS ONE style templates can be found at https://journals.plos.org/plosone/s/file?id=wjVg/PLOSOne_formatting_sample_main_body.pdf and https://journals.plos.org/plosone/s/file?id=ba62/PLOSOne_formatting_sample_title_authors_affiliations.pdf

Authors’ response:

Thank you for this reminder. We have carefully reviewed the provided PLOS ONE style templates and confirm that our manuscript now fully adheres to all formatting requirements, including file naming conventions, reference style, and manuscript structure.

2. Please note that PLOS One has specific guidelines on code sharing for submissions in which author-generated code underpins the findings in the manuscript. In these cases, we expect all author-generated code to be made available without restrictions upon publication of the work. Please review our guidelines at https://journals.plos.org/plosone/s/materials-and-software-sharing#loc-sharing-code and ensure that your code is shared in a way that follows best practice and facilitates reproducibility and reuse.

Authors’ response:

We will share all author-generated code without restrictions in a public GitHub repository upon manuscript acceptance. The repository will be cited in the manuscript, include a detailed README file, and be archived on Zenodo to provide a permanent DOI. We confirm adherence to PLOS ONE's code sharing guidelines.

3. When completing the data availability statement of the submission form, you indicated that you will make your data available on acceptance. We strongly recommend all authors decide on a data sharing plan before acceptance, as the process can be lengthy and hold up publication timelines. Please note that, though access restrictions are acceptable now, your entire data will need to be made freely accessible if your manuscript is accepted for publication. This policy applies to all data except where public deposition would breach compliance with the protocol approved by your research ethics board. If you are unable to adhere to our open data policy, please kindly revise your statement to explain your reasoning and we will seek the editor's input on an exemption. Please be assured that, once you have provided your new statement, the assessment of your exemption will not hold up the peer review process.

Authors’ response:

We will upload the fully anonymized dataset as Supporting Information files upon manuscript acceptance. We confirm our adherence to PLOS ONE's open data policy. The Data Availability statement has been updated accordingly.

4. We note that you have indicated that there are restrictions to data sharing for this study. For studies involving human research participant data or other sensitive data, we encourage authors to share de-identified or anonymized data. However, when data cannot be publicly shared for ethical reasons, we allow authors to make their data sets available upon request. For information on unacceptable data access restrictions, please see http://journals.plos.org/plosone/s/data-availability#loc-unacceptable-data-access-restrictions. Before we proceed with your manuscript, please address the following prompts:

a) If there are ethical or legal restrictions on sharing a de-identified data set, please explain them in detail (e.g., data contain potentially identifying or sensitive patient information, data are owned by a third-party organization, etc.) and who has imposed them (e.g., a Research Ethics Committee or Institutional Review Board, etc.). Please also provide contact information for a data access committee, ethics committee, or other institutional body to which data requests may be sent.

b) If there are no restrictions, please upload the minimal anonymized data set necessary to replicate your study findings to a stable, public repository and provide us with the relevant URLs, DOIs, or accession numbers. Please see http://www.bmj.com/content/340/bmj.c181.long for guidelines on how to de-identify and prepare clinical data for publication. For a list of recommended repositories, please see https://journals.plos.org/plosone/s/recommended-repositories. You also have the option of uploading the data as Supporting Information files, but we would recommend depositing data directly to a data repository if possible.

Please update your Data Availability statement in the submission form accordingly.

Authors’ response:

We will upload the fully anonymized dataset as Supporting Information files alongside our manuscript. The Data Availability statement has been updated to reflect this.

5. Please amend your list of authors on the manuscript to ensure that each author is linked to an affiliation. Authors’ affiliations should reflect the institution where the work was done (if authors moved subsequently, you can also list the new affiliation stating “current affiliation:….” as necessary).

Authors’ response:

Thank you for this reminder. We have carefully reviewed and amended the author list and affiliations to ensure each author is correctly linked to their respective institution(s).

6. We note that Figure 1 in your submission contain [map/satellite] images which may be copyrighted. All PLOS content is published under the Creative Commons Attribution License (CC BY 4.0), which means that the manuscript, images, and Supporting Information files will be freely available online, and any third party is permitted to access, download, copy, distribute, and use these materials in any way, even commercially, with proper attribution.

Authors’ response:

We thank the editor for this important note. We confirm that Figure 1 is an original creation by the authors and does not contain any copyrighted material. The map was created by the authors using the open-source software QGIS (version 3.30), utilizing our study data. The figure caption has been updated to include the required attribution: "Created with QGIS ("http://qgis.org)." We hereby grant permission for it to be published under the CC BY 4.0 license.

Reviewer # 1 Comments

Abstract:

1. The abstract should clearly summarize the research manuscript’s conclusions. It is currently too general. It should specify the research findings with numerical statistics (if any).

Authors’ response:

We sincerely thank the reviewer for this valuable and constructive feedback. We agree that the inclusion of specific numerical results is crucial for a strong and informative abstract. We have thoroughly revised the abstract to explicitly state our key findings with the relevant statistical metrics, as requested. The revised abstract now includes the following specific numerical results:

� Total TS occurrences: The cumulative thunderstorm days for each region over the study period (Sylhet: 6,029; Sreemangal: 4,954; Mymensingh: 4,488).

� Model performance improvement: The precise reduction in Mean Squared Error (MSE) achieved by our best model (over 65%, with a concrete example: from 14.17 to 4.82 MSE at Sylhet).

� Key error metrics: The actual values for the Root Mean Squared Error (RMSE: 2.20 to 1.91) and Mean Absolute Scaled Error (MASE: ~0.30) that demonstrate the high predictive accuracy of our top-performing model.

Thank you again for this suggestion, which has greatly improved our manuscript.

Introduction:

1. Which months are the pre-monsoon and monsoon season? It is not mentioned in the

introduction.

Authors’ response:

We appreciate the reviewer’s helpful comment. In the revised manuscript, we have explicitly defined the time periods for both the pre-monsoon and monsoon seasons in Bangladesh.

2. Please improvise on the introduction section. It does not address recent research studies

that could be relevant.

Authors’ response:

We sincerely thank the reviewer for this critical and constructive feedback. We agree that incorporating recent, relevant studies is essential for grounding our research in the current scientific discourse and clearly establishing its novelty. In response to this comment, we have thoroughly revised the Introduction section to significantly strengthen its foundation with up-to-date and highly relevant literature.

Materials and Methods:

1. Figure 1 is not ideal for the journal paper. In the figure, there is no scale bar, coordinate, and the resolution is too low.

Authors’ response:

We agree with the reviewer and have replaced Figure 1 with a new, high-resolution map that now includes a scale bar, geographic coordinates (latitude/longitude), and a north arrow. This revised figure provides the necessary spatial context and meets the required standards for publication.

2. Revise the section on the study area and data collection. It seems more relevant to the results section rather than the study area and data collection part. For your example, you can see this paper: ‘GIS-based spatial analysis for lightning scenario in Bangladesh’.

Authors’ response:

We thank the reviewer for this insightful suggestion and for providing a helpful reference. Following the structure of the suggested paper, we have revised the manuscript by renaming the section as "Study Area" and have kept it to the Materials and Methods section. We have also carefully edited the content of this section to focus purely on the description of the selected regions and the source and nature of the dataset, removing any preliminary results or interpretations that were prematurely presented.

3. To model and forecast the monthly frequency of TS days in high-risk locations of Bangladesh specifically, Sylhet, Sreemangal, and Mymensingh, this study employs a diverse set of forecasting techniques, encompassing both traditional time series models and modern machine learning approaches – irrelevant in the methods section.

Authors’ response:

Thank you for the valuable suggestion. In response, we have revised the “Modeling Framework” section to focus strictly on procedural steps rather than study objectives. We now describe the implementation of forecasting models and seasonal adjustment techniques in a stepwise and technical manner. The updated section avoids general statements of intent and aligns more appropriately with methodological standards expected in the Materials and Methods section.

4. ARIMA Model – add citation.

Authors’ response:

Thank you for your valuable suggestion. We have now added appropriate citations to foundational and widely accepted sources on the ARIMA model in the Materials and Methods section.

5. In the methods section, better write the full form of the heading of all the model names.

Authors’ response:

Thank you for your helpful suggestion. In response, we have revised the manuscript to include the full form of each model name in the respective section headings to improve clarity and readability.

6. Evaluation Metrics – use citation.

Authors’ response:

Thank you for the suggestion. We have now included appropriate citations for the evaluation metrics used in the Materials and Method section.

7. The datasets have not been compared with other datasets, such as the ISS-LIS dataset.

Please provide justification for not using satellite-based data.

Authors’ response:

Thank you for this valuable comment. We acknowledge the relevance of satellite-based datasets, such as the Lightning Imaging Sensor onboard the International Space Station (ISS-LIS), for studying global lightning and thunderstorm activity. However, we respectfully note that the ISS-LIS dataset provides relatively short-term coverage beginning in 2017 (Blakeslee et al., 2020), which does not align with the scope of our study covering a 40-year period from 1985 to 2024. Additionally, our objective was to model station-level thunderstorm (TS) activity using temporally consistent and locally validated data. Ground-based observations from the Bangladesh Meteorological Department (BMD) ensure high spatial and temporal resolution suitable for such analysis. Furthermore, satellite-based datasets often suffer from spatial aggregation and reduced detection reliability under specific atmospheric conditions in this region (Abdelmajeed & Juszczak, 2024; Disadvantages of Satellite: An Extensive Examination (Updated), n.d.). We have included this justification in the revised manuscript (Study Area and Data Collection) and have added appropriate citations. Future work will explore integrating satellite-derived lightning indices to complement and expand upon the present findings.

Results:

1. Data description is not part of the result. Revise it.

Authors’ response:

Thank you for the valuable comment. We have revised the manuscript by renaming the section to "Regional Thunderstorm Frequency and Characteristics" to clearly distinguish the descriptive overview from the core results. The detailed data description, including study area and data source information, has been retained in the Materials and Methods section to maintain appropriate manuscript structure. This ensures the Results section focuses on the analysis and interpretation of thunderstorm patterns, in accordance with standard scientific reporting practices.

2. Why are Sylhet, Sreemangal, and Mymensingh the most TS-prone regions in Bangladesh? Provide further reasons.

Authors’ response:

Thank you for the insightful comment. We have addressed this in the revised manuscript by explaining the climatological and topographical factors contributing to high thunderstorm activity in these regions. Specifically, the orographic uplift from the Meghalaya Plateau, sustained moisture influx from the Bay of Bengal, and the convergence of moist easterlies and westerly disturbances create favorable conditions for thunderstorm formation. This is now clearly described in the "Regional Thunderstorm Frequency and Characteristics" section.

3. Why does Sylhet have the most thunderstorms?

Authors’ response:

We appreciate the reviewer’s request for clarification. The manuscript now includes a detailed explanation that Sylhet’s unique physiography being situated in a basin surrounded by Meghalaya and Tripura hills, enhances moisture trapping and orographic forcing. Its proximity to very wet highland areas like Cherrapunji and Mawsynram provides a continuous moisture supply, lengthening the convective season and increasing thunderstorm frequency. These points are incorporated in the revised "Regional Thunderstorm Frequency and Characteristics" section.

4. The result part is not well structured and described. The findings should be described in the results section very clearly.

Authors’ response:.

We thank the reviewer for this feedback. We have thoroughly restructured the Results section to improve its clarity and flow.

Discussion:

1. For Bangladesh, why is the pre-monsoon season only April to May, and also the monsoon season (June – August)? As per the climatic season, March – June is Pre- monsoon and June – September is the Monsoon season.

Authors’ response:

We thank the reviewer for pointing out the official seasonal definitions used by the Bangladesh Meteorological Department. While climatologically the pre-monsoon is March–May and the monsoon is June–September, our analysis of storm occurrence data from 1985–2024 shows that thunderstorm activity is most concentrated during April–May (pre-monsoon peak) and June–August (monsoon peak). This data-driven conclusion is supported by the monthly distribution patterns shown in Figures 4–6, which consistently highlight these months as having the highest TS activity across all three stations. We have now clarified this rationale in the Discussion section to avoid confusion and emphasize that our seasonal framing is based on observed TS frequency, not general climatological periods.

Conclusion:

1. This manuscript has no conclusion section.

Authors’ response:

Thank you for the comment. We have now added a clear Conclusion section highlighting the novelty of our station-level forecasting approach, the effectiveness of seasonal adjustment methods, and the policy relevance of our findings for improving early warning systems and disaster preparedness in Bangladesh.

Overall Structure and Flow:

1. The authors should maintain a smoother manuscript flow of writing and coherence of each section. In addition, the authors have not explained the reasons for TS occurrences in the specific areas. The current level of description is not deep enough. This manuscript has no page and line numbers, making it difficult for a reviewer to review.

Authors’ response:

We deeply appreciate the reviewer's insightful and helpful criticism, which has significantly helped us to improve the manuscript's clarity, depth, and overall quality. We have undertaken a comprehensive revision to address each point raised.

We sincerely apologize for this oversight, which undoubtedly complicated the review process. We have now added page number and continuous line numbers throughout the entire revised manuscript to facilitate a much smoother review.

Reviewer # 2 Comments

Introduction:

1. More accurate and updated references needed and are available. I am not commenting on each reference. We should provide more matching references and credit should go to those really contributed to specific knowledge domain. Background information of this paper is much off the way of its main two research objectives. More accurate and updated context and references about thunderstorm characteristics and impacts should be provided.

1. Rahman, Md. M., Shobuj, I. A., Santo, Md. M. H., Ali, H. M., Hossain, Md. T., Alam, E., & Islam, Md. K. (2025). Knowledge, attitude, and practices towards lightning among vulnerable group: A case study on Agri farmers and fishermen. Sustainable Environment, 11(1). https://doi.org/10.1080/27658511.2025.2464373

2. Akter, M.Y., Islam, A.R.M., Mallick, J., Alam, E., 2024. Temperature extremes Projections over Bangladesh from CMIP6 Multi-model Ensemble. Theor Appl Climatol.. https://doi.org/10.1007/s00704-024-05173-5

3. Rahman, M.M., Mannan, M.A., Sarkar, M.S.K., Sultana, A., Islam, M.M., Akter, M.Y., Alam, E., and Islam, A.R.M.T. 2024. Are hotspots and frequencies of heatwave changing over time? Exploring causes of heatwaves in a tropical country. Plos One, 19 (5): e0300070. https://doi.org/10.1371/journal.pone.0300070

4. Islam, A.R.M.T, Hasanuzzaman, M., Jaman, M., Alam, E., Mallick, J., Alam, G.M.M, Sattar, M.A., Techato, K., 2021. Assessing farmers perceived typologies concerning environmental issues and climate change for sustainable adaptation strategies in Bangladesh, Climate Journal. Xx. Doi: org/10.3390/cli9120167

Authors’ response:

Thank you for this critical and constructive feedback. We agree that incorporating recent, relevant studies is essential for grounding our research in the current scientific discourse and clearly establishing its novelty. In response to this comment, we have thoroughly revised the Introduction section to significantly strengthen its foundation with up-to-date and highly relevant literature. The specific improvements we have made include:

� Incorporation of Suggested References: We have integrated the key recent studies suggested by the reviewer (Rahman et al., 2025; Akter et al., 2024; Rahman et al., 2024; Islam et al., 2021) to precisely address the context of vulnerability, climate trends, and adaptation in Bangladesh. These citations now serve to: reframe the problem, update the climate context, and justify the need for better forecasting.

� The revised “Introduction” now clearly articulates the research gap, stating that prior work in Bangladesh often relies on aggregated national data and a critical gap exists in developing high-resolution, localized forecasting models for vulnerable regions.

� The global context has been simplified, making the overview more relevant to the study's methodology.

Hence, the revised Introduction has significantly improved the introduction by incorporating a more relevant set of references, allowing for a more compelling presentation of the research gap and a clearer justification for the study's objectives.

Data used and methodology:

1. Methods are substantial. A step by step wise data collection, data cleaning and analysis variables, and processes may enhance method section.

Authors’ response:

We thank the reviewer for this positive feedback and valuable suggestion. We have thoroughly revised the ‘Materials and Methods’ section to provide a clearer, step-by-step description of our workflow.

Results:

1. The results are clearly presented.

Authors’ response:

Thank you for your positive comment on the Results section. In response to Reviewer 1’s suggestions, we have further refined this section to improve clarity and structure. We believe these improvements have strengthened the presentation of our findings.

Discussion:

1. This section is substantial.

Authors’ response:

Thank you for your positive feedback on the Discussion section. We have endeavored to provide a comprehensive and detailed analysis to thoroughly interpret our findings in the context of existing literature and practical implications. We hope the depth of discussion enhances the overall quality and clarity of the manuscript.

Conclusion:

1. It is not clear from the analysis how their findings are new and novel from current understanding of the relevant science and how they are contributing to improve current state of art in the discipline. I would encourage to write how the policy sciences can be improved from the results presented in the discipline.

Authors’ response:

Thank you for the comment. We have now added a clear Conclusion section highlighting the novelty of our station-level forecasting approach, the effectiveness of seasonal adjustment methods, and the policy relevance of our findings for improving early warning systems and disaster preparedness in Bangladesh.

Attachment Submitted filename: Response_to_Reviewers_PONE-D-25-30212.pdf

# Peer review: Decision Letter 1

**DOI:** 10.1371/journal.pone.0358795.r003

**Authors:** Rajib Chowdhury

8 Oct 2025

Dear Dr. Rois,

Thank you for submitting your manuscript to PLOS ONE. After careful consideration, we feel that it has merit but does not fully meet PLOS ONE’s publication criteria as it currently stands. Therefore, we invite you to submit a revised version of the manuscript that addresses the points raised during the review process.

Please submit your revised manuscript by Nov 22 2025 11:59PM. If you will need more time than this to complete your revisions, please reply to this message or contact the journal office at plosone@plos.org. When you're ready to submit your revision, log on to https://www.editorialmanager.com/pone/ and select the 'Submissions Needing Revision' folder to locate your manuscript file.

- A rebuttal letter that responds to each point raised by the academic editor and reviewer(s). You should upload this letter as a separate file labeled 'Response to Reviewers'.
- A marked-up copy of your manuscript that highlights changes made to the original version. You should upload this as a separate file labeled 'Revised Manuscript with Track Changes'.
- An unmarked version of your revised paper without tracked changes. You should upload this as a separate file labeled 'Manuscript'.

If you would like to make changes to your financial disclosure, please include your updated statement in your cover letter. Guidelines for resubmitting your figure files are available below the reviewer comments at the end of this letter.

If applicable, we recommend that you deposit your laboratory protocols in protocols.io to enhance the reproducibility of your results. Protocols.io assigns your protocol its own identifier (DOI) so that it can be cited independently in the future. For instructions see: https://journals.plos.org/plosone/s/submission-guidelines#loc-laboratory-protocols. Additionally, PLOS ONE offers an option for publishing peer-reviewed Lab Protocol articles, which describe protocols hosted on protocols.io. Read more information on sharing protocols at https://plos.org/protocols?utm_medium=editorial-email&utm_source=authorletters&utm_campaign=protocols.

We look forward to receiving your revised manuscript.

Kind regards,

Rajib Chowdhury, M.Sc.; MPH

Academic Editor

PLOS ONE

Journal Requirements:

If the reviewer comments include a recommendation to cite specific previously published works, please review and evaluate these publications to determine whether they are relevant and should be cited. There is no requirement to cite these works unless the editor has indicated otherwise.

[Note: HTML markup is below. Please do not edit.]

Reviewers' comments:

Reviewer's Responses to Questions

**Comments to the Author**

Reviewer #1: (No Response)

Reviewer #3: All comments have been addressed

Reviewer #4: (No Response)

Reviewer #5: (No Response)

Reviewer #6: (No Response)

Reviewer #7: (No Response)

Reviewer #8: All comments have been addressed

Reviewer #9: (No Response)

**********

2. Is the manuscript technically sound, and do the data support the conclusions?

Reviewer #1: Yes

Reviewer #3: Yes

Reviewer #4: No

Reviewer #5: No

Reviewer #6: Yes

Reviewer #7: Partly

Reviewer #8: Yes

Reviewer #9: (No Response)

**********

3. Has the statistical analysis been performed appropriately and rigorously?

Reviewer #1: Yes

Reviewer #3: Yes

Reviewer #4: No

Reviewer #5: No

Reviewer #6: Yes

Reviewer #7: I Don't Know

Reviewer #8: Yes

Reviewer #9: (No Response)

**********

4. Have the authors made all data underlying the findings in their manuscript fully available?

The PLOS Data policy

Reviewer #1: Yes

Reviewer #3: Yes

Reviewer #4: No

Reviewer #5: No

Reviewer #6: Yes

Reviewer #7: Yes

Reviewer #8: Yes

Reviewer #9: (No Response)

**********

5. Is the manuscript presented in an intelligible fashion and written in standard English?

Reviewer #1: Yes

Reviewer #3: Yes

Reviewer #4: No

Reviewer #5: Yes

Reviewer #6: Yes

Reviewer #7: Yes

Reviewer #8: Yes

Reviewer #9: (No Response)

**********

Reviewer #1: Comments:

1. The study area is not well described. The study area section should provide a clear description of the geographical, environmental, climatic, and socio-economic characteristics of the study area, supported by relevant maps or figures. So that, reader can get a clear picture and idea about your research region. The authors should improve it and see other published papers for a good understanding.

2. The authors can rename the study area to “study area and data.” Give a brief description of your dataset (like stations, data resolutions, temporal resolutions, etc.).

3. Lines 161 – 164 are irrelevant in the study area section.

4. The conclusion does not represent the key findings of this research. I would encourage the authors to improve the conclusion section and explain how the findings would be helpful for the decision makers.

Reviewer #3: The manuscript appears suitably revised based on the comments on the earlier version. Recommended for publication in the revised form

Reviewer #4: (No Response)

Reviewer #5: Title: Climate Change and Seasonal Thunderstorm Intensification: Evidence from Bangladesh’s Most Vulnerable Regions

In this manuscript, the authors investigated the yearly and monthly variation in the frequency of thunderstorms (TS) in 3 cities of Bangladesh. They also developed 6 statistical models for forecasting monthly TS frequency during 2025-2028. The result found that the model with seasonally adjusted data obtained the best performance. Unfortunately, the manuscript contains several vital issues, especially the lack of explanation about how the models are trained. A lot of analyses are redundant and not well linked to each other. As a result, the manuscript in the current form cannot be published unless the quality of the study and manuscript is significantly improved, which should include a redesign of the study to make the focus (TS forecasting) clearer.

The major comments:

1. As explained in the introduction, the research gap is “high-resolution, localized forecasting models that account for the unique climatology of Bangladesh's most vulnerable regions.” However, only 2 of the 10 figures plus one table are related to the forecasting models. The relevant content is disproportionately insufficient. Moreover, the remaining 8 figures do not have any supporting role to pave the way for the development of the model. The analyses associated with Figs. 1-8 are the background information. If the authors insist on the same research gap, the manuscript should be reorganized to emphasize on the building, training, and evaluation of the models, and minimize irrelevant contents (including the figures and analyses).

2. Since the objective is to develop models for better forecasting of TS, the introduction does not contain relevant background information related to the topic. The first 4 paragraphs are simply generic information about TS. The authors should introduce the challenges of forecasting of monthly TS frequency in Bangladesh. The literature review would be strengthened by including a discussion of highly relevant prior work, such as “Development of novel hybrid machine learning models for monthly thunderstorm frequency prediction over Bangladesh” (https://link.springer.com/article/10.1007/s11069-021-04722-9). This study also develops machine learning models for thunderstorm forecasting in Bangladesh and should be addressed.

3. The authors spend a lot of time on explaining the annual cycle of the number of thunderstorm (Figs. 3-8). However, it is neither new or hidden knowledge. This information can be found in https://www.banglajol.info/index.php/BJPhy/article/view/78684, which is published in 2022. Only very little can be regarded as new information. The redundancy is excessive even if removing the seasonal variable from the training data of models is a key aspect of this study.

4. For machine learning models, it is important to describe how these models are trained. It should include the split of training, validation, and test dataset, hyperparameters, etc. The training and evaluation of ML models have profound impact of the performance of the models. However, these information are entirely missing in the manuscript. The authors must clearly describe the training and evaluation procedure of each model. Because the structure, setting, and training data allocation of the machine learning and deep learning model are entirely unknown, it is impossible to know whether these models are well trained or overfitted. An LSTM is a powerful algorithm, but its performance is highly dependent on its architecture and training. Without a well-documented design and sufficient training, it is possible for it to underperform simpler models. Therefore, it is impossible to give a fair and meaningful model evaluation.

5. The description and interpretation of all analyses and figures are insufficient. For example, the interpretation of Fig. 2 focuses on the earlier part of the time series while the more recent period is ignored. The authors should provide a complete analysis of the entire period, especially given the apparent decreasing trend in recent years, or provide a rationale for its exclusion. There is also no comparison between the results of the 3 cities. Since the 3 cities are neigboring cities, it is possible to analyze the average of them, instead of analyze them individually, if there is no strong reason for doing so.

6. The author should explain why the seasonally adjusted data lead to better model performance.

7. The title does not align with the content. The study is clearly not exmaining climate change, although there are a few yearly time series; TS does not show intensification trend as well. The authors should revise the title to accurately reflect the content and results.

8. Figure 2: The figure is inappropriate. Quantity with different units should not share the same y-axis. The variation in relative humidity and average temperature cannot be observed due to the overly large maximum value in y. Please include only one variable in one panel.

9. Figure 2: In Sylhet, there is an observable decreasing trend since 2010. For Sreemangal, the decreasing trend is weaker than Sylhet, but the one for Mymensingh is a little bit clearer. These are important findings in the figure, but the authors omit them.

10. L355: The decreasing trend in TS frequency is obvious in the 3 cities. The authors must clearly explain how they can attribute climate change to such a decreasing trend.

11. L380-381: This statement is a purely qualitative observation. The authors need to connect it to the decreasing trend of the number of TS observed in Fig. 2. I suggest the authors use a t-test to confirm whether a trend is statistically significant of not.

12. The function of boxplots in Figs. 4-6 overlap with Fig. 2. To reduce information redundancy, the authors should keep either boxplots of line plots. Also, the authors should also describe the difference in result between the 3 cities. Moreover, Figs 4-6 should be merged into one figure.

13. Figs 4-6: Similar to Fig 2, the monthly TS count in the 3 cities show a clear decreasing trend in the recent decade.

14. L513-518: The authors should explain why there is a discrepancy in the monsoon season definition between your result and the conventional climatological framework. Does the conventional one represent better at national level rather than city level?

Minor comments:

15. L69-70: "Damage" should not be used for "human health". “Leading to …….and risk to human health” is more appropriate. Moreover, it does not seems there is a direct relationship between thunderstorms and human health. Please add references as evidence for such a relationship.

16. The first 2 paragraphs have common topic: damage. They should be combined into one.

17. L78: Remove the word “tragic”.

18. L110-111: This paragraph is all about forecasting. However, the studies of the 2 references (28, 29) only used WRF for studying thunderstorms, but not forecasting them. These 2 references are not suitable for this paragraph. Please remove them.

19. L122: What is the forecast lead time?

20. L140: This subsection is actually explaining your dataset, and is irrelevant to the study domain. Please describe why you select the 3 cities and exclude the others in more details to justify your choice. Also, write a subsection specifically for describing your dataset.

21. It is difficult to distinguish the level of subsection as the number for indicating sub-section is missing. Please assign subsection number.

22. Figure 1: The font of city name are too small to be read easily. Please enlarge them.

23. L147-148: Please describe how TS events is counted in this dataset.

24. L152-164: This paragraph will be clearer if you first explain why you use the ground-based dataset, and then why you don't use the satellite dataset.

25. L163-164: This sentence is unnecessary and inappropriate for this section. Please remove.

26. L184-185: Machine learning approach is statistical in nature, so it is correct to frame all these 6 techniques as statistical methods.

27. L188: Abbreviation has been defined earlier on L176. It is unnecessary to write the term in full. The same issue exists for the subsection of all models. Please correct it.

28. L219-220: The sentence "Click or tap here to enter text" appears too many times. The authors should make sure unnecessary text are removed before submission.

29. L276-298: I suggest the authors include figures to demonstrate a few time series before and after processed by the two decomposition method. It is the best way to show the readers the difference between them, and how they work.

30. L327-329: These are information for study area. Please move it to Section 2.

31. L337-338: Although you cited a reference, it is necessary to explicitly explain why large rainfall amount can help severe storm development. It does not sound like a proper casual relationship.

32. L356: Figure 2 only shows annual value, seasonal dynamics cannot be observed. Please correct this sentence.

33. Figure 3: The x-axis should be month instead of season.

34. L406: How is the trend calculated? By moving average? If yes, it is more appropriate to call it "X-year moving average" because "trend" is more specific, usually linear.

35. Figure 8: Ridgeline density plot is not suitable when data contains very small number of samples (those winter months). Also, TS count is always positive, so the the part in the negative region is non-physical. The authors must trim it to avoid misleading the readers.

36. Figs. 8 & 10: What is the value associated with each radius in the plot? On the other hand, the information of this plot is the same as Fig. 3 after taking the average of all years. It is easier for the readers to extract information from a line plot or bar chart than a radar plot.

37. Table 1: Besides showing a table, I suggest the authors to visualize the model performance using barchart. It can make the comparison of each model easier.

38. L447: What is the data period for model evaluation?

39. L450: ANN is consider as deep learning as long as the network contains at least one hidden layer. Please revise.

40. L483: What is the input variables (including lag time) and forecast lead time of the output of the model? These are important information.

41. L495-509: This paragraph describes the background of this study, so it should belong to the Introduction section.

Reviewer #6: I have read the revision and compared it with its original version. I think the comments from the two reviewers have well pointed out the problems of the manuscript. However, although the authors claimed that all the comments have been well addressed in the revision, I found the distance between the comments and their edits is still evident. For example, the revised abstract is still not under a typical and academic structure. I suggest that the authors should re-consider these comments very carefully and make their revision with more patience and more times.

Reviewer #7: The manuscript investigates how climate change has influenced the frequency and intensity of thunderstorms in Bangladesh, with a focus on vulnerable regions. Also, the study seeks both to describe long-term trends and to develop accurate forecasting models that can support disaster preparedness and climate resilience. Overall, the manuscript presents relevant findings with methodological rigor, but it still requires improvements in clarity, methodological transparency, and practical framing to fully demonstrate its scientific and operational value.

Abstract:

The abstract starts by describing the dataset and then quickly moves to present the forecasting results. While it does provide numerical findings and highlights the superiority of the STL-XGBoost model, it does not clearly state the objective and scope of the study before diving into outcomes. A stronger abstract would first frame the research purpose, then the methodological approach and the forecasting results.

Introduction:

The revised Introduction successfully incorporates the reviewers’ earlier suggestions. The authors clarified the seasonal definitions, added more recent and relevant references, and justified their focus on the northeastern regions of Bangladesh. These changes make the section more coherent and better grounded in current literature. Overall, the Introduction now establishes the research gap more clearly, though it could still be streamlined to reduce redundancy and emphasize the study’s novelty more directly.

Methods: The revised Methods section is clearer, with added details on data sources, model descriptions, and proper citations. The authors also improved Figure 1 by including a scale bar, coordinates, and a north arrow. However, the figure still does not adequately highlight the three study areas, which should be more clearly marked to better illustrate the research scope.

Also, this section would benefit from more information on data quality and consistency across the forty-year period. It is unclear whether the dataset contained missing values, how such gaps (if any) were handled, or whether there were changes in measurement methods or station relocations between 1985 and 2024.

One important omission in the Methods section is the lack of detail about the training, validation, and testing strategy for the forecasting models. The manuscript explains which models were applied and how seasonal adjustment was used, but it does not specify how the dataset was divided into training and testing periods, or whether cross-validation was employed. Without this information, it is difficult to assess the robustness and generalizability of the results. Explicitly stating the temporal ranges used for training, validation, and forecasting would strengthen the methodological transparency and reproducibility of the study.

In addition, the description of the forecasting models does not mention any hyperparameter search or tuning process. For complex models such as SVR, ANN, XGBoost, and LSTM, predictive performance is highly sensitive to parameter settings. Without clarification of how hyperparameters were selected—whether through grid search, cross-validation, or another optimization strategy—it is difficult to judge the robustness of the reported results.

Results:

The revised Results section is better structured and now separates descriptive information from analytical findings. The authors also explain more clearly why Sylhet, Sreemangal, and Mymensingh are the most thunderstorm-prone regions, linking them to topography and moisture inflows. Figures and tables are informative, but some captions remain overly descriptive. In particular, for less common visualizations, it would be useful to add more guidance on how to interpret the results.

Figures 4–6: boxplots of monthly variation; the colors used are not explained and there is no color bar, leaving the reader uncertain about their meaning.

Figure 7: ridgeline density plot; this less familiar type of visualization would benefit from guidance on how to interpret the curves and the color gradients; additionally, the color bar is missing a title that explains what it represents.

Figures 8 and 10: radar plots; the graphics lack detail on what each plotted point represents; the captions should clarify whether points correspond to normalized averages, monthly frequencies, or forecasted values.

Discussions:

The revised section is more comprehensive and better integrates the findings with existing literature.

In the Discussion, it would also be valuable to explain how the proposed forecasting framework could be integrated into operational practice. For instance, the STL-XGBoost model could be embedded within the Bangladesh Meteorological Department’s workflow to provide monthly or seasonal outlooks of thunderstorm activity.

Reviewer #8: 1. Overall Significance

• The manuscript addresses an important and timely topic: the intensification of thunderstorms (TS) in Bangladesh under climate change, which has major societal and economic implications.

• The use of station-level forecasting with seasonal adjustment and machine learning is innovative compared to many prior works relying on pooled or regional averages.

• However, the paper would benefit from clearer articulation of the novelty: is the innovation primarily methodological (STL-XGBoost hybrid), geographic focus (Bangladesh), or policy relevance (early warning systems)?

2. Abstract and Introduction

• The abstract is well-structured but somewhat dense. Consider simplifying technical details (e.g., naming fewer models in the abstract) and emphasizing the key policy relevance more strongly.

• The introduction reviews global and local literature but could expand discussion of:

o Why thunderstorms specifically (vs. cyclones/floods) have been understudied in Bangladesh.

o The unique socio-economic vulnerability of Sylhet, Sreemangal, and Mymensingh beyond climatology (e.g., agriculture dependence, lightning mortality).

o How this study directly advances or contrasts with prior works using SARIMA/ARIMA in Bangladesh

3. Methods

• The dataset description (1985–2024, BMD station data) is clear. Still:

o Clarify data quality control: Were missing values imputed? Were homogenization checks performed?

o Explain whether lightning-day definitions or storm thresholds were consistent over the 40 years.

• The seasonal adjustment section is strong (X-11 vs STL), but more justification of why STL was expected to perform better would help (currently explained mostly post-results).

• The model comparison:

o Six forecasting models are compared (ARIMA, ETS, ANN, SVR, XG-Boost, LSTM). However, LSTM performed very poorly (MSE > 100). Why? Was hyperparameter tuning sufficient? Could this be due to data scarcity?

o Were cross-validation or out-of-sample tests done, or only in-sample error reporting? Clarify.

o Report computational efficiency – were some models more resource-intensive, which could matter for operational use by BMD?

4. Results

• Results are clearly presented with detailed tables of MSE, RMSE, and MAE across stations.

• STL-XG-Boost consistently outperforms, but the margins of improvement over STL-SVR or STL-ETS could be better highlighted numerically (e.g., % gain).

• Figures:

o Figure 2 (stacked bars of temperature, humidity, and TS counts) is visually overloaded. Consider splitting variables or using line+bar overlays for clarity.

o Forecast plots (2025–2028, Figure 9) are strong but could include confidence intervals to communicate uncertainty.

o Radar plot (Figure 10) is informative but unusual in meteorology—justify its use or replace with boxplots/seasonal profiles.

5. Discussion

• Discussion appropriately situates results in the climate change context, but could improve by:

o Explicitly quantifying the policy implications (e.g., how much earlier warnings could be issued, or which adaptation strategies might be prioritised).

o Linking to regional/global thunderstorm studies (India, China, Nepal) more directly to highlight comparative insights.

o Addressing limitations: reliance on only 3 stations, no incorporation of reanalysis/satellite data, and lack of socio-economic outcome modelling.

6. References

• The reference list is comprehensive, covering climatology, forecasting, and machine learning.

• Some sources (blogs/tutorials like Analytics Vidhya) may not be acceptable for a high-impact journal—consider replacing with peer-reviewed methodological references.

• Add more citations on lightning mortality in Bangladesh and regional ENSO/IOD linkages.

7. Language and Style

• Generally clear, but some sections are overly technical for a generalist journal like PLOS ONE. Recommend:

o Shortening methodological jargon in the abstract/introduction.

o Ensuring figures and captions are self-contained.

o Using consistent terminology (e.g., TS = thunderstorm vs TS days vs thunderstorm frequency).

Summary Recommendation

• Major revision recommended. The study is important, but revisions are needed in clarifying novelty, improving data transparency, justifying modelling choices, and refining figures/policy discussion.

• With these addressed, the manuscript could make a meaningful contribution to climate resilience and forecasting in South Asia.

Reviewer #9: Review report on the revised manuscript titled “Climate Change and Seasonal Thunderstorm Intensification: Evidence from Bangladesh’s Most Vulnerable Regions” Mohammad Mahboob Hussain Khan, Amrin Binte Ahmed, Adisha Dulmini, Muhammad Abu Sufian, Dulsaba Azmain, Md. Mahin Uddin Qureshi, Mustafizur Rahman, and Rumana Rois

Manuscript Number: PONE-D-25-30212R1

The manuscript analyzes monthly thunderstorm (TS) occurrences over Sylhet, Sreemangal, and Mymensingh using long-term station data (1985–2024). Six forecasting techniques, Autoregressive Integrated Moving Average (ARIMA), Exponential Smoothing (ETS), Artificial Neural Network (ANN), Support Vector Regression (SVR), Extreme Gradient Boosting (XGBoost), and Long Short-Term Memory (LSTM), have been employed. Additionally, decomposition and Seasonal–Trend decomposition using Loess (STL) were used to adjust for seasonality.

The topic addressed is of considerable scientific and practical importance, particularly for regional policy planning and climate-related risk management. However, several substantive issues must be addressed. Specific comments are provided below for the authors’ consideration.

Comment 1: The title “Climate Change and Seasonal Thunderstorm Intensification: Evidence from Bangladesh’s Most Vulnerable Regions” suggests an analysis of thunderstorm intensification, but the manuscript only examines occurrence frequency. The authors should either incorporate analyses addressing TS intensity or revise the title to accurately reflect the study’s actual content.

Comment 2: The Introduction section requires substantial improvement. Its current structure lacks logical flow and does not clearly establish the research gap. The authors should (i) synthesize prior studies, (ii) identify knowledge deficiencies, and (iii) articulate the novelty and objectives of the present work more explicitly.

Comment 3: It is unclear how monthly-scale thunderstorm frequency predictions can be translated into early warning system applications, which typically operate on shorter timescales. The authors should elaborate on the operational relevance and potential end-use of such forecasts.

Comment 4: Figure captions are currently embedded within the main text and not easily distinct. Captions should be clearly separated from the main body, numbered sequentially, and self-explanatory.

Comment 5: The modeling framework is described only with basic mathematics. The authors should briefly explain the theoretical basis and functionality of each model (ARIMA, ETS, ANN, SVR, XGBoost, LSTM), highlighting their strengths, limitations, and suitability for the dataset and forecasting objectives.

Comment 6: Multiple instances of placeholder text (“Click or tap here to enter text…”) appear throughout the manuscript. These should be removed or replaced with appropriate content.

Comment 7: The manuscript uses basic performance metrics (MSE, MAE, MAPE, MASE, RMSE). The rationale for their selection should be justified, and the significance of each metric discussed. It is strongly recommended to include additional and more diagnostic statistics such as percentage bias (PBIAS), normalized RMSE/MSE, RSR (RMSE and standard deviation ratio), Nash-Sutcliffe efficiency coefficient (NSE), and Index of Agreement (d) to provide a more comprehensive assessment of model performance.

Comment 8: The Discussion section needs major revision. The authors should interpret the results in a scientific context, linking them to prior studies and physical processes. Furthermore, it is essential to explain why certain models outperform others, supported by data-driven reasoning.

Comment 9: The manuscript combines both pre-monsoon and monsoon thunderstorm events. Since the driving mechanisms differ substantially between these periods (e.g., pre-monsoon TS events are also influenced by cyclonic activity), the authors should clarify whether large-scale influences were identified and excluded prior to analysis.

Comment 10: The methodological description and model validation procedure are insufficient. Details regarding model training, testing, and validation must be clearly provided. The authors should specify the data partitioning scheme (training vs. testing periods), justify its choice, and evaluate the statistical significance of the model predictions.

Comment 11: All figures require improvement in visual quality and resolution.

Comment 12: The relative humidity and temperature plots in Figure 2 are not adequately discussed. A physical interpretation should be added, and it is recommended to present anomalies with respect to the climatological mean to better capture deviations and variability.

Comment 13: Figures 4–6 present similar types of plots for different stations. For compactness and easier comparison, these can be combined into a single multi-panel figure with clear sub-labels (e.g., 4a–4c).

Comment 14: Clarify the method used for plotting trend lines in Figures 4–6. Are they running trends? If so, specify the window size. Alternatively, indicate whether these are the median of the seasonally adjusted trends. Statistical significance of the trends should also be reported.

Comment 15: The manuscript discusses only the occurrence frequency of thunderstorms, not their duration or intensity. Therefore, text sections implying intensity analysis should be condensed or revised accordingly.

Comment 16: The role and significance of the WRF model in this study are unclear. The authors must clarify its purpose and how it relates to the TS occurrence prediction framework in the present study.

Comment 17: The conclusion should be rewritten to emphasize the major findings, contributions, and practical implications of the research, while avoiding repetition of earlier results.

**********

what does this mean?). If published, this will include your full peer review and any attached files.

If you choose “no”, your identity will remain anonymous but your review may still be made public.

**Do you want your identity to be public for this peer review?**  For information about this choice, including consent withdrawal, please see our Privacy Policy

Reviewer #1: No

Reviewer #3: No

Reviewer #4: No

Reviewer #5: No

Reviewer #6: No

Reviewer #7: No

Reviewer #8: No

Reviewer #9: No

**********

[NOTE: If reviewer comments were submitted as an attachment file, they will be attached to this email and accessible via the submission site. Please log into your account, locate the manuscript record, and check for the action link "View Attachments". If this link does not appear, there are no attachment files.]

While revising your submission, please upload your figure files to the Preflight Analysis and Conversion Engine (PACE) digital diagnostic tool, https://pacev2.apexcovantage.com/. PACE helps ensure that figures meet PLOS requirements. To use PACE, you must first register as a user. Registration is free. Then, login and navigate to the UPLOAD tab, where you will find detailed instructions on how to use the tool. If you encounter any issues or have any questions when using PACE, please email PLOS at figures@plos.org. Please note that Supporting Information files do not need this step.

Attachment Submitted filename: PONE-D-25-30212R1-review.pdf

Attachment Submitted filename: Review Report _ R1.docx

Attachment Submitted filename: PONE-D-25-30212_report1.docx

Attachment Submitted filename: Reviewer Comments on 02.10.2025.doc

# Author comment: Author response to Decision Letter 2

**DOI:** 10.1371/journal.pone.0358795.r004

24 Mar 2026

17 March 2026

Climate Change and Seasonal Thunderstorm Intensification: Evidence from Bangladesh’s Most Vulnerable Regions (PONE-D-25-30212R1)

Dear Editor,

We sincerely thank you and all the reviewers for your valuable time, insightful comments, and constructive feedback on our revised manuscript. We greatly appreciate the opportunity to further improve our work. We have carefully considered every comment and have made corresponding revisions to enhance the manuscript's scientific quality, clarity, methodological transparency, and overall structure. Based on reviewer feedback, we have revised the title to “Climate Change and Seasonal Thunderstorm Dynamics: Evidence from Bangladesh's Most Vulnerable Regions” to more accurately reflect the study's content, which focuses on thunderstorm frequency rather than intensification. The substantial methodological improvements necessitated the inclusion of an additional co-author, Rawfun Nahin Kowbi, who made significant contributions to the revised modeling framework, hyperparameter tuning implementation, and validation procedures. All authors have approved this addition. Below, we provide a detailed, point-by-point response to all editorial and reviewer comments.

Best regards,

Authors

Reviewer #1: Comments

1. The study area is not well described. The study area section should provide a clear description of the geographical, environmental, climatic, and socio-economic characteristics of the study area, supported by relevant maps or figures. So that, reader can get a clear picture and idea about your research region. The authors should improve it and see other published papers for a good understanding.

Authors’ response:

We thank the reviewer for this valuable suggestion and have substantially improved the Study Area and Data subsection to provide a clearer picture of our research regions. In the revised manuscript, we now provide:

� Enhanced geographical description: Lines 233-243 and Table 1 now describe each station's location, proximity to the Meghalaya Plateau, and orographic setting in detail.

� Comprehensive Table 1: A new table summarizing station name, ID, coordinates, and long-term (1985–2024) monthly averages for all meteorological variables (TS frequency, temperature, relative humidity, cloud cover, rainfall, atmospheric pressure). This numerical summary complements the textual description and explicitly highlights that Sylhet has the highest long-term mean thunderstorm activity (12.56 frequency/month), followed by Sreemangal (10.32) and Mymensingh (9.35).

� Improved Figure 1: The map now includes a scale bar, coordinates, north arrow, and the three study stations are clearly marked with larger red stars and labeled directly.

� Socio-economic context: We now explicitly state as a limitation (Lines 220-225) that consistent station-level socio-economic time series are not available for the full 1985–2024 period. We therefore restrict quantitative analysis to meteorological observations and discuss socio-economic vulnerability qualitatively using recent literature, with citations to media reports and survey-based studies [86-89].

2. The authors can rename the study area to “study area and data.” Give a brief description of your dataset (like stations, data resolutions, temporal resolutions, etc.).

Authors’ response:

We agree with this recommendation. Section 2 has been reorganized as follows:

2.1 Study Area and Data

2.1.1 Description of Study Stations and Dataset

2.1.2 Justification for Ground-Based Data

This reorganization improves logical flow. Section 2.1.1 now provides complete dataset description including stations (IDs, coordinates), variables (TS frequency, temperature, relative humidity, cloud cover, rainfall, atmospheric pressure), temporal coverage (1985–2024, 480 months), and data resolution (monthly aggregates from 3-hourly observations). Which ensures readers have complete context before encountering the modeling methodology.

3. Lines 161 – 164 are irrelevant in the study area section.

Authors’ response:

Lines 161-164 (discussion of satellite data limitations) have been removed from the Study Area section and relocated to 2.1.2 Justification for Ground-Based Data, a new subsection that follows the dataset description. This placement is more logical, as it explains our data choice immediately after presenting the dataset. The relocated text has also been substantially condensed to focus only on the temporal coverage mismatch (ISS-LIS begins 2017 vs. our 40-year record) and spatial resolution issues, removing extraneous detail.

4. The conclusion does not represent the key findings of this research. I would encourage the authors to improve the conclusion section and explain how the findings would be helpful for the decision makers.

Authors’ response:

We have completely rewritten the Conclusion section to:

� Summarize key empirical findings with numerical results: correlation analysis (r values), univariate model performance (RMSE = 2.08–2.53), exogenous model including climate predictors (temperature, relative humidity, cloud cover, rainfall, and atmospheric pressure) improvements (MASE = 0.17–0.22), and over 80% improvement compared to univariate benchmarks.

� Articulate methodological contributions with humility: systematic comparison of 18 modeling approaches across two frameworks – univariate and exogenous – with seasonal decomposition.

� Provide actionable recommendations for decision-makers:

o BMD can implement validated STL-XGBoost and STL-SVR frameworks for station-specific forecasts

o Seasonal projections identify April–August as the critical preparedness window for targeted resource allocation

o Demonstrated value of integrating climatic variables provides scientific basis for investing in meteorological data collection

o Reproducible pathway to enhance early warning systems, and inform climate-resilient infrastructure planning

� Acknowledge data limitations and emphasize need for sustained interdisciplinary collaboration to translate forecasting improvements into measurable risk reduction outcomes.

Reviewer #3: Comments

The manuscript appears suitably revised based on the comments on the earlier version. Recommended for publication in the revised form.

Authors’ response:

Thank you for your positive feedback and recommendation for publication. We appreciate your time and constructive input on the earlier version.

Reviewer #4: Comments

General Assessment:

This manuscript addresses thunderstorm (TS) occurrences in northeastern Bangladesh and applies several statistical and machine learning models to station data. While the topic is relevant, the study suffers from fundamental flaws in design, data, methods, and interpretation. The work is presented with ambitious claims but lacks the scientific rigor and novelty to support them. The problems are structural and cannot be fixed by revision.

Authors’ response:

We thank Reviewer #4 for the thorough critique. We have taken these concerns very seriously and have fundamentally revised the manuscript to address each issue. The substantial methodological improvements—including climate predictors, e.g., temperature, relative humidity, cloud cover, rainfall, and atmospheric pressure, in the exogenous model framework, rigorous trend analysis, comprehensive hyperparameter tuning, expanded model validation, and tempered claims—have resulted in a much stronger manuscript. We respectfully submit that the structural problems have been addressed through the following revisions:

Major Issues:

1. Lack of definition and data transparency

• The manuscript never defines what constitutes a “thunderstorm occurrence” in the BMD dataset (observer logs, lightning detection, rainfall with thunder, etc.). Without this, the data cannot be meaningfully interpreted or validated.

• No discussion of reporting consistency across the 40-year period is provided, raising concerns about data inhomogeneity.

Authors’ response:

We have added a clear definition and data quality and consistency information in Section 2.1:

“Following the BMD Operational Manual [32], a TS occurrence is officially defined as an observation period during which thunder is audibly heard by a certified meteorological observer at the station, regardless of whether accompanying precipitation occurs. TS frequency is defined as the number of thunderstorm occurrences recorded at a given location over a specified temporal scale (e.g., daily, monthly, seasonal, or annual). In the BMD network, thunderstorm observations are made eight times per day at 3‑hour intervals following World Meteorological Organization (WMO) standard synoptic hours. All data underwent rigorous quality control by BMD, including consistency checks and temporal continuity verification, with no major station relocations and consistent adherence to WMO standards throughout 1985–2024. The count of thunderstorm reports within a calendar day is treated as the daily TS frequency, and monthly TS frequency for each station is obtained by aggregating the corresponding daily frequencies over the month.”

2. Misuse of “High-resolution forecasting”

• The authors frame a “critical gap” in high-resolution localized forecasting. However, their dataset consists of monthly counts from only three stations, which is the opposite of high-resolution both spatially and temporally.

• The claim that this study addresses that gap is misleading.

Authors’ response:

We acknowledge this valid criticism and have revised the language throughout the manuscript. We have:

� Removed the term "high-resolution" from all instances, replacing it with “accurate” or “localized” or “station-specific” forecasting

� Reframed the research gap: “However, a critical gap remains in developing accurate localized forecasting models that account for the unique climatology of Bangladesh's most vulnerable regions.”

� Clarified the contribution: “We move beyond aggregated national models to provide station-specific forecasts using a comprehensive suite of eighteen modeling approaches... to capture the unique temporal and spatial dynamics of thunderstorms.”

The revised language accurately reflects that our contribution is station-specific modeling rather than high-resolution forecasting.

3. Weak and misapplied methods

• Six models and two seasonal decompositions are tested on a very small dataset (~1,440 monthly points). Applying complex ML models (XGBoost, LSTM) to such limited univariate data is inappropriate and risks overfitting.

• The methods section is bloated with textbook definitions but omits crucial details (data quality control, training/validation strategy, hyperparameter tuning, uncertainty estimation).

Authors’ response:

We have substantially revised the Methods section to address these concerns:

� Acknowledged dataset limitations: We now explicitly discuss that monthly data (480 observations per station) may be insufficient for complex deep learning models, which explains LSTM's poor performance

� Added models (SARIMAX, ETSX, ANN, SVR, XGBoost, LSTM) incorporating climate predictors - temperature, relative humidity, cloud cover, rainfall, and air pressure - to capture the meteorological determinants of TS activity. (Section 2.2.2)

� Added rigorous cross-validation: Section 2.2.4 now describes time-series cross-validation with expanding window (5 folds, 12-month forecast horizon) specifically designed to prevent overfitting while respecting temporal dependencies.

� Comprehensive hyperparameter tuning: We have added detailed hyperparameter tuning information in two new supplementary tables (Table S1 and S2), including search ranges, optimal values, and validation performance (mean RMSE ± std across CV folds). This ensures transparency and demonstrates that overfitting was actively prevented.

� Condensed textbook definitions: We have significantly shortened the theoretical descriptions of each model (now 2-3 sentences per model) and focused on their application to our specific dataset and objectives. The removed text is available in supplementary materials if needed.

4. Results as filler

• 8 out of 10 figures and large parts of the text are devoted to basic descriptive climatology of three stations (seasonal cycles, boxplots, radar charts). These simply restate well-known patterns (April-June peaks, Sylhet wetter than others) and add no new major insight.

• The forecasting results (Table 1 and Figures 9-10) are thin, and the main finding (i.e., seasonal decomposition improves model performance) is trivial and well- established.

Authors’ response:

We have re-run all analyses using exogenous models that include climate predictors, and substantially reorganized the Results section to better balance descriptive and analytical content:

� Condensed descriptive climatology: The descriptive material has been streamlined and integrated into Section 3.1. Figures 3-6 have been combined where appropriate (e.g., boxplots for all three stations now in a single multi-panel Figure 3).

� Expanded forecasting results: We have added: Detailed comparison of univariate vs. exogenous model performance (Section 3.2, Figures 5-6), Comprehensive error metrics in Tables 2-3, Hyperparameter tuning validation results in Tables S1-S2, Long-term forecasts (2025–2028) in Figure 7 to demonstrate model behavior over extended horizons

� Contextualized the seasonal decomposition finding: While the value of seasonal decomposition is well-established in time series analysis, its application to thunderstorm forecasting in Bangladesh—particularly the comparison of X-11 vs. STL and the integration with machine learning models—represents a novel contribution. We now emphasize this more clearly.

5. Unsupported climate change attribution

• The manuscript repeatedly attributes thunderstorm intensification to climate change, yet no formal trend analysis, attribution methods, or diagnostics (e.g., CAPE, vertical wind shear, ENSO/IOD influences) are performed.

• Claims of “confirmation” are based only on visual inspection of time series, which is not rigorous.

Authors’ response:

We fully agree and have completely revised the climate change framing:

� Added formal trend analysis: Table 1 now includes Mann-Kendall test results, Sen's slope estimates, 95% confidence intervals, and p-values for each station. This rigorous statistical analysis reveals a significant decreasing trend only at Sylhet (1.75 days per decade), with no significant trends at Sreemangal and Mymensingh.

� Tempered claims: We have removed all unsupported attributions. The revised text (Lines 180-195) now states: "These findings demonstrate that claims of systematic thunderstorm intensification in Bangladesh are not supported by station-level occurrence data from these three locations. Only Sylhet shows a detectable change, and that change is a decline, not an increase."

� Acknowledged limitations: We explicitly note that we do not perform formal attribution to climate change, as this would require additional diagnostics (CAPE, vertical wind shear, large-scale climate drivers) beyond the scope of this study.

� Reframed the discussion: The Discussion now appropriately contextualizes results within climate change literature without making unsupported claims.

6. Overstated novelty and impact

• The use of STL decomposition with XGBoost is presented as a methodological breakthrough, but in reality, this is a routine time-series preprocessing step.

• The authors extrapolate forecasts to 2025-2028 and present them as reliable. With such limited data and simplistic models, these projections are scientifically meaningless.

• Claims that the work informs “early warning systems, climate-resilient infrastructure, and public health” are vastly overstated given the coarse dataset and limited results.

Authors’ response:

We have substantially tempered our claims throughout the manuscript:

� Reframed methodological contribution: We now present STL+XGBoost as a "validated framework" rather than a "breakthrough." The contribution is positioned as the systematic comparison of 18 approaches for station-specific forecasting in Bangladesh—a context where such comprehensive evaluation was previously lacking.

� Contextualized forecasts: We now clearly state (Lines 658-660) that “However, this forecast skill degrades rapidly with increasing lead time, and these projections are presented as illustrations of model behavior rather than reliable long-term predictions.” The forecasts are framed as demonstrating model capability rather than providing definitive future estimates.

� Qualified practical implications: We have carefully qualified statements about operational utility. For example (Lines 808-811): “Monthly frequency forecasts cannot directly support the imminent threat warnings—minutes to hours—that are most critical for life-saving actions during individual TS events. However, monthly outlooks serve a complementary function: they inform preparedness planning at weekly to monthly lead times.”

7. Contradictions in discussion

• The authors acknowledge major limitations (monthly aggregation, only three stations, univariate models without covariates), which in fact invalidate their main conclusions.

• Despite these admissions, they still make strong claims about climate change impacts and operational utility, creating a clear disconnect between evidence and conclusions.

Authors’ response:

We have carefully revised the Discussion to ensure alignment between acknowledged limitations and conclusions:

� Strengthened limitation discussion: Section 4 (Lines 700-750) now provides a comprehensive and upfront discussion of limitations, including: Monthly data cannot support minute-to-hour warnings, Only three stations limit spatial generalizability, and Lack of socio-economic data prevents impact assessment.

� Removed disconnect: All strong claims about climate change impacts have been removed. Operational recommendations are now framed as potential pathways that require further validation and institutional collaboration.

8. Conclusion

• The conclusion is awkwardly brief, merely repeating methods and results without genuine synthesis, which reinforces the manuscript’s imbalance and lack of depth.

Authors’ response:

We have entirely revised the Conclusion section. Now the Conclusion section presents findings modestly and acknowledges that realizing operational benefits requires sustained interdisciplinary collaboration and addressing data limitations.

Summary

This is a classic case of overselling: the manuscript makes ambitious claims about filling critical gaps and supporting climate resilience, but the actual results are limited to descriptive plots and small error reductions in basic forecasting models. The disconnect between claims and evidence, combined with missing definitions, filler content, and inappropriate methodology, makes the manuscript unsuitable for publication.

Authors’ response:

We thank Reviewer #4 for this candid assessment. We have fundamentally revised the manuscript to address each issue:

� Overselling and disconnect: We have systematically tempered all claims. Formal trend analysis (Mann-Kendall test, Table 1) now shows no significant intensification at Sreemangal and Mymensingh, and a significant decline at Sylhet—contradicting earlier assumptions. We now frame our contribution modestly as "station-specific forecasting" rather than gap-filling. Operational relevance is qualified: monthly forecasts inform preparedness planning but cannot support minute-to-hour warnings. Forecasts are presented as illustrations of model behavior, not reliable long-term predictions.

� Missing definitions: We have added clear definitions of thunderstorm occurrence in Section 2, data quality procedures, and handling of missing values.

� Filler content: Descriptive climatology has been condensed (Section 3.1), while forecasting content is expanded with comprehensive error metrics (Tables 2-3), hyperparameter tuning results (Tables S1-S2), and station-specific season-wise and long-term forecasts for univariate and exogenous models (Figures 5-7).

� Inappropriate methodology: We have added eighteen modeling approaches across two frameworks—univariate (SARIMA, ETS, SVR, ANN, XGBoost, LSTM) and exogenous (SARIMAX, ETSX, SVR, ANN, XGBoost, LSTM) with X-11 and STL decompositions—with rigorous time-series cross-validation (expanding window, 5 folds), comprehensive hyperparameter tuning, and explicit acknowledgment of limitations (e.g., LSTM underperformance due to sample size).

The revised manuscript now aligns claims with evidence, provides complete transparency, and employs appropriate methodology. We hope the reviewer will find it suitable for reconsideration.

Reviewer #5: Comments

In this manuscript, the authors investigated the yearly and monthly variation in the frequency of thunderstorms (TS) in 3 cities of Bangladesh. They also developed 6 statistical models for forecasting monthly TS frequency during 2025-2028. The result found that the model with seasonally adjusted data obtained the best performance. Unfortunately, the manuscript contains several vital issues, especially the lack of explanation about how the models are trained. A lot of analyses are redundant and not well linked to each other. As a result, the manuscript in the current form cannot be published unless the quality of the study and manuscript is significantly improved, which should include a redesign of the study to make the focus (TS forecasting) clearer.

The Major Comments:

1. As explained in the introduction, the research gap is “high-resolution, localized forecasting models that account for the unique climatology of Bangladesh's most vulnerable regions.” However, only 2 of the 10 figures plus one table are related to the forecasting models. The relevant content is disproportionately insufficient. Moreover, the remaining 8 figures do not have any supporting role to pave the way for the development of the model. The analyses associated with Figs. 1-8 are the background information. If the authors insist on the same research gap, the manuscript should be reorganized to emphasize on the building, training, and evaluation of the models, and minimize irrelevant contents (including the figures and analyses).

Authors’ response:

We have substantially revised and reorganized the Results section to emphasize forecasting. Descriptive climatology is now condensed into Section 3.1 (Figures 3-6 combined into one multi-panel new Figure 3), while forecasting content is expanded with:

� Detailed univariate vs. exogenous model comparison (Sections 3.1-3.2, Tables 2-3)

� Hyperparameter tuning validation (Tables S1-S2)

� Monthly and seasonal forecasts using univariate and exogenous models (new Figures 5-7).

2. Since the objective is to develop models for better forecasting of TS, the introduction does not contain relevant background information related to the topic. The first 4 paragraphs are simply generic information about TS. The authors should introduce the challenges of forecasting of monthly TS frequency in Bangladesh. The literature review would be strengthened by including a discussion of highly relevant prior work, such as “Development of novel hybrid machine learning models for monthly thunderstorm frequency prediction over Bangladesh”

(https://link.springer.com/article/10.1007/s11069-021-04722-9). This study also develops machine learning models for thunderstorm forecasting in Bangladesh and should be addressed.

Authors’ response:

We have revised the introduction section and added discussion of forecasting challenges, incorporated Azd et al. (2021) [30] and other relevant prior work. For instance, “Additionally, hybrid approaches like the ensemble empirical mode decomposition (EEMD)-SVM model [30] and statistical methods like SARIMA [31] have been applied to forecast TS frequency, often using aggregated national data.”

Which clearly distinguished our contribution from previous studies, for example Azd et al. (2021) [30] (https://link.springer.com/article/10.1007/s11069-021-04722-9).

3. The authors spend a lot of time on explaining the annual cycle of the number of thunderstorm (Figs. 3-8). However, it is neither new or hidden knowledge. This information can be found in

https://www.banglajol.info/index.php/BJPhy/article/view/78684, which is published in 2022. Only very little can be regarded as new information. The redundancy is excessive even if removing the seasonal variable from the training data of models is a key aspect of this study.

Authors’ response:

Thank you for your constructive suggestion. Descriptive climatology now has been significantly condensed (Section 3.1). The focus is now on how seasonal patterns inform model development (e.g., need for STL decomposition) rather than repeating established knowledge.

4. For machine learning models, it is important to describe how these models are trained. It should include the split of training, validation, and test dataset, hyperparameters, etc. The training and evaluation of ML models have profound impact of the performance of the models. However, these information are entirely missing in the manuscript. The authors must clearly describe the training and evaluation procedure of each model. Because the structure, setting, and training data allocation of the machine learning and deep learning model are entirely unknown, it is impossible to know whether these models are well trained or overfitted. An LSTM is a powerful algorithm, but its performance is highly dependent on its architecture and training. Without a well-documented design and sufficient training, it is possible for it to underperform simpler models. Therefore, it is impossible to give a fair and meaningful model evaluation.

Authors’ response:

We have revised the entire analysis and added the following in Section 2.2.4:

� Data partitioning: 80% training (1985-2016), 20% test (2017-2024)

� Time-series cross-validation with expanding window (5 folds, 12-month horizon)

� Comprehensive hyperparameter tuning with grid search (Tables S1-S2)

5. The description and interpretation of all analyses and figures are insufficient. For example, the interpretation of Fig. 2 focuses on the earlier part of the time series while the more recent period is ignored. The authors should provide a complete analysis of the entire period, especially given the apparent decreasing trend in recent years, or provide a rationale for its exclusion. There is also no comparison between the results of the 3 cities. Since the 3 cities are neighboring cities, it is possible to analyze the average of them, instead of analyze them individually, if there is no strong reason for doing so.

Authors’ response:

We have revised Figure 2 to include five additional climate variables and expanded its interpretation to cover the entire study period, including post-2010 trends. City-level comparisons are now explicitly discussed, with individual station analyses preferred over averaging to preserve location-specific patterns.

6. The author should explain why the seasonally adjusted data lead to better model performance.

Authors’ response:

Added explanation in Section 3.2 (Lines 500-510): STL removes dominant seasonal cycle, allowing models to focus on underlying patterns and relationships with climatic variables, reducing noise and improving forecast accuracy.

7. The title does not align with the content. The study is clearly not examining climate change, although there are a few yearly time series; TS does not show intensification trend as well. The authors should revise the title to accurately reflect the content and results.

Authors’ response:

We have revised the Title to: "Climate Change and Seasonal Thunderstorm Dynamics: Evidence from Bangladesh's Most Vulnerable Regions". Formal trend analysis (Table 1) now shows no significant intensification at two stations and a decline at Sylhet.

8. Figure 2: The figure is inappropriate. Quantity with different units should not share the same y-axis. The variation in relative humidity and average temperature cannot be observed due to the overly large maximum value in y. Please include only one variable in one panel.

Authors’ response:

Figure 2 redesigned as multi-panel plot with separate y-axes per variable, improving clarity.

9. Figure 2: In Sylhet, there is an observable decreasing trend since 2010. For Sreemangal, the decreasing trend is weaker than Sylhet, but the one for Mymensingh is a little bit clearer. These are important findings in the figure, but the authors omit them.

Authors’ response:

Thank you for such an important suggestion. Post-2010 trends now explicitly discussed and formally analyzed with Mann-Kendall tests (Table 1).

10. L355: The decreasing trend in TS frequency is obvious in the 3 cities. The authors must clearly explain how they can attribute climate change to such a decreasing trend.

11. L380-381: This statement is a purely qualitative observation. The authors need to connect it to the decreasing trend of the number of TS observed in Fig. 2. I suggest the authors use a t-test to confirm whether a trend is statistically significant of not.

Authors’ response:

We have: Added formal Mann-Kendall trend tests with Sen's slope (Table 1); Removed unsupported climate attribution claims; and Acknowledged that formal attribution requires additional diagnostics beyond scope.

12. The function of boxplots in Figs. 4-6 overlap with Fig. 2. To reduce information redundancy, the authors should keep either boxplots of line plots. Also, the authors should also describe the difference in result between the 3 cities. Moreover, Figs 4-6 should be merged into one figure.

13. Figs 4-6: Similar to Fig 2, the monthly TS count in the 3 cities show a clear decreasing trend in the recent decade.

Authors’ response:

Figures 3-6 combined into a single multi-panel Figure 3. Boxplots retained as they show distribution (median, spread, outliers) while line plots show temporal evolution—complementary information.

14. L513-518: The authors should explain why there is a discrepancy in the monsoon season definition between your result and the conventional climatological framework. Does the conventional one represent better at national level rather than city level?

Authors’ response:

We have added explanation: Our station-level data show peak activity concentrated in April-August, offering local-scale operational insight. The conventional definition (June-September) remains valid at national scale.

Minor comments:

15. L69-70: "Damage" should not be used for "human health". “Leading to …….and risk to human health” is more appropriate. Moreover, it does not seem there is a direct relationship between thunderstorms and human health. Please add references as evidence for such a relationship.

Authors’ response:

Thank you for this thoughtful suggestion. We have revised to "leading to substantial damage to infrastructure, agriculture, and risks to human health". Added references [1-3] documenting health impacts.

16. The first 2 paragraphs have common topic: damage. They should be combined into one.

Authors’ response:

Paragraphs 1-2 combined into a single paragraph.

17. L78: Remove the word “tragic”.

Authors’ response:

Removed.

18. L110-111: This paragraph is all about forecasting. However, the studies of the 2 references (28, 29) only used WRF for studying thunderstorms, but not forecasting them. These 2 references are not suitable for this paragraph. Please remove them.

Authors’ response:

We thank the reviewer for this clarification. The mention of the WRF model in the Introduction (Lines 123-125) serves only to acknowledge previous studies that have used numerical weather prediction models for thunderstorm simulation in Bangladesh [28,29]. It is not part of our methodology. To avoid confusion, we have revised the sentence to clearly indicate that this refers to prior work, not the current study: "Numerical models, such as the Advanced Research Weather Research and Forecasting (ARW-WRF) model, have been used in previous studies to simulate storms in Bangladesh [28,29].”

19. L122: What is the forecast lead time?

Authors’ response:

Lead time specified in Section 3.3, monthly forecasts for 2025-2028 (48-month horizon).

20. L140: This subsection is actually explaining your dataset, and is irrelevant to the study domain. Please describe why you select the 3 cities and exclude the others in more details to justify your choice. Also, write a subsection specifically for describing your dataset.

Authors’ response:

We have added justification: stations chosen due to historically elevated TS frequencies [19,20,33] and location within highly active convective zone. Dataset description now in Section 2.1.1.

21. It is difficult to distinguish the level of subsection as the number for indicating sub-section is missing. Please assign subsection number.

Authors’ response:

All subsections now numbered (e.g., 2.1.1, 2.1.2).

22. Figure 1: The font of city names are too small to be read easily. Please enlarge them.

Authors’ response:

Figure 1 regenerated with a bit larger fonts for city names (see revised Figure 1).

23. L147-148: Please describe how TS events are counted in this dataset.

Authors’ response:

We have added clear definition in Section 2.1, eight daily observations at 3-hour intervals following WMO standards; monthly frequency obtained by aggregating daily counts.

24. L152-164: This paragraph will be clearer if you first explain why you use the ground-based dataset, and then why you don't use the satellite dataset.

Authors’ response:

Reorganized as suggested: first explain why ground-based data used (Section 2.1.1), then why satellite not used (Section 2.1.2).

25. L163-164: This sentence is unnecessary and inappropriate for this section. Please remove.

Authors’ response:

Removed.

26. L184-185: Machine learning approach is statistical in nature, so it is correct to frame all these 6 techniques as statistical methods.

Authors’ response:

Revised to Section 2.2.1 Univariate Models and 2.2.2 Models Incorporating Climate Predictors to address that.

27. L188: Abbreviation has been defined earlier on L176. It is unnecessary to write the term in full. The same issue exists for the subsection of all models. Please correct it.

Authors’ response:

All abbreviations now defined only at first use and used consistently thereafter.

28. L219-220: The sentence "Click or tap here to enter text" appears too many times. The authors should make sure unnecessary text are removed before submission.

Authors’ response:

Removed.

29. L276-298: I suggest the authors include figures to demonstrate a few time series before and after processed by the two decomposition method. It is the best way to show the readers the difference between them, and how they work.

Authors’ response:

We have added Figure 7 to display the time series for each station. In Figure 3, the green line is the raw time series, while the red line is the STL-adjusted series.

30. L327-329: These are information for study area. Please move it to Section 2.

Authors’ response:

Thank you. Moved to Section 2.1.1 as recommended.

31. L337-338: Although you cited a reference, it is necessary to explicitly explain why large rainfall amount can help severe storm development. It does not sound like a proper causal relationship.

Authors’ response:

Added explanation: heavy rainfall indicates deep convective systems with strong updrafts, ice particle interactions, and charge separation—all conducive to thunderstorm development.

32. L356: Figure 2 only shows annual value; seasonal dynamics cannot be observed. Please correct this sentence.

Authors’ response:

Corrected: now refers to annual values only.

33. Figure 3: The x-axis should be month instead of season.

Authors’ response:

Corrected to month names (Jan-Dec) in revised Figure 3.

34. L406: How is the trend calculated? By moving average? If yes, it is more appropriate to call it "X-year moving average" because "trend" is more specific, usually linear.

Authors’ response:

35. Figure 8: Ridgeline density plot is not suitable when data contains very small number of samples (those winter months). Also, TS count is always positive, so the part in the negative region is non-physical. The authors must trim it to avoid misleading the readers.

Authors’ response:

Removed.

36. Figs. 8 & 10: What is the value associated with each radius in the plot? On the other hand, the information of this plot is the same as Fig. 3 after taking the average of all years. It is easier for the readers to extract information from a line plot or bar chart than a radar plot.

Authors’ response:

Removed.

37. Table 1: Besides showing a table, I suggest the authors to visualize the model performance using bar chart. It can make the comparison of each model easier.

Authors’ response:

Thank you for your thoughtful suggestion to visualize model performance using bar charts. We agree that this could enhance comparability across models.

However, we prefer to retain the tabular format for several reasons. Our manuscript already contains seven figures, and adding another would create imbalance. Additionally, tables allow precise reporting of all four evaluation metrics (MSE, RMSE, MAE, MASE) across eighteen models and three stations—information that would be difficult to convey clearly in a bar chart. The current table provides comprehensive numerical detail for readers and future researchers.

We appreciate your understanding.

38. L447: What is the data period for model evaluation?

Authors’ response:

We clarified it in Section 2.2.4. The evaluation period is January 2017 to December 2024.

39. L450: ANN is considered as deep learning as long as the network contains at least one hidden layer. Please revise.

Authors’ response:

Corrected: ANN now classified as machine learning, with note that deep learning refers specifically to multi-hidden-layer architectures.

40. L483: What are the input variables (including lag time) and forecast lead time of the output of the model? These are important information.

Authors’ response:

Added detailed input specifications in Section 2.2.2, Section 2.2.4, and Tables S1-S2, including lag structures and exogenous variables.

41. L495-509: This paragraph describes the background of this study, so it should belong to the Introduction section.

Authors’ response:

Moved to Introduction as recommended.

We thank the reviewer for this comprehensive feedback that has helped us substantially strengthen the manuscript.

Reviewer #6: Comments

I have read the revision and compared it with its original version. I think the comments from the two reviewers have well pointed out the problems of the manuscript. However, although the authors claimed that all the comments have been well addressed in the revision, I found the distance between the comments and their edits is still evident. For example, the revised abstract is still not under a typical and academic structure. I suggest that the authors should re-consider these comments very carefully and make their revision with more patience and more times.

Authors’ response:

We thank Reviewer #6 for this insightful assessment. We have taken this feedback very seriously and have thoroughly re-reviewed all comments and corresponding revisions. We have now:

� Completely restructured the abstract to follow a standard academic structure: purpose → methodology → key results → conclusions/implications.

� Systematically addressed each comment with a point-by-point response document and corresponding manuscript revisions.

� Added extensive methodological detail on data quality, cross-validation, hyperparameter tuning, and model validation to ensure transparency.

� Tempered all claims to align with evidence and acknowledged limitations.

� Improved figure clarity with more informative captions and interpretation guidance.

We believe these revisions have substantially closed the gap between comments and edits.

Reviewer #7: Comments

The manuscript investigates how climate change has influenced the frequency and intensity of thunderstorms in Bangladesh, with a focus on vulnerable regions. Also, the study seeks both to describe long-term trends and to develop accurate forecasting models that can support disaster preparedness and climate resilience. Overall, the manuscript presents relevant findings with methodological rigor, but it still requires improvements in clarity, methodological transparency, and practical framing to fully demonstrate its scientific and operational value.

1. Abstract:

The abstract starts by describing the dataset and then quickly moves to present the forecasting results. While it does provide numerical findings and highlights the superiority of the STL-XGBoost model, it does not clearly state the objective and scope of the study before diving into outcomes. A stronger abstract would first frame the research purpose, then the methodological approach and the forecasting results.

Authors’ response:

We thank the reviewer for this valuable suggestion. The abstract has been completely restructured to follow a clear, logical flow.

2. Introduction:

The revised Introduction successfully incorporates the reviewers’ earlier suggestions. The authors clarified the seasonal definitions, added more recent and relevant references, and justified their focus on the northeastern regions of Bangladesh. These changes make the section more coherent and better grounded in current literature. Overall, the Introduction now establishes the research gap more clearly, though it could still be streamlined to reduce redundancy and emphasize the study’s novelty more directly.

Authors’ response:

We appreciate the reviewer's positive assessment. In response to the suggestion for further streamlining, we have:

� Reduced redundancy: Condensed overlapping discussions of thunderstorm impacts and climate change linkages, while retaining all essential information and citations.

� Emphasized novelty more directly: Added explicit statements highlighting the study's three key innovations (Lines 150-158):

� Strengthened research objectives: Clearly stated three research objectives that directly flow from the identified research gap.

The Introduction now more effectively establishes the research gap and articulates the study's novel contributions while maintaining a coherent, well-grounded narrative.

3. Methods:

The revised Methods section is clearer, with added details on data sources, model descriptions, and proper citations. The authors also improved Figure 1 by including a scale bar, coordinates, and a north arrow. However, the figure still does not adequately highlight the three study areas, which should be more clearly marked to better illustrate the research scope.

Also, this section would benefit from more information on data quality and consistency across the forty-year period. It is unclear whether the dataset contained missing values, how such gaps (if any) were handled, or whether there were changes in measurement methods or station relocations between 1985 and 2024.

One important omission in the Methods section is the lack of detail about the training, validation, and testing strategy for the forecasting models. The manuscript explains which models were applied and how seasonal adjustment was used, but it does not specify how the dataset was divided into training and testing periods, or whether cross-validation was employed. Without this information, it is difficult to assess the robustness and generalizability of the results. Explicitly stating the temporal ranges used for training, validation, and forecasting would strengthen the methodological transparency and reproducibility of the study.

In addition, the description of the forecasting models does not mention any hyperparameter search or tuning process. For complex models such as SVR, ANN, XGBoost, and LSTM, predictive performance is highly sensitive to parameter settings. Without clarification of how hyperparameters were selected—whether through grid search, cross-validation, or another optimization strategy—it is difficult to judge the robustness of the reported results.

Authors’ response:

We thank the reviewer for these detailed and constructive comments. We have addressed each point as follows:

� Figure 1 Improvement: We have revised Figure 1 to more clearly highlight the three study areas.

� Data Quality and Consistency: We have added a new paragraph in Section 2.1.addressing data quality.

� Training, Validation, and Testing Strategy: We have substantially expanded Section 2.2.4 to provide complete transparency: “Monthly TS frequency data from January 1985 to December 2024 were partitioned into training (80%) and test (20%) sets. Data from January 1985 to December 2016 (384 observations) were used for model development, hyperparameter tuning, and cross-validation. The remaining period from January 2017 to December 2024 (96 observations) was reserved exclusively as an unseen test set for final performance assessment, ensuring that all reported accuracy metrics reflect genuine out-of-sample predictive capability [70,71].

Given the temporal nature of the data, standard k-fold cross-validation is inappropriate as it ignores the sequential dependence structure [72]. We therefore implemented time-series cross-validation with an expanding window and rolling forecasting origin [73,74]. For each iteration, models were trained on all available data up to a given cut-off point and then used to forecast subsequent steps ahead (1, 3, 6, and 12 months) to evaluate both short-term and multi-step forecasting capability [75]. The forecast errors from each iteration were recorded, and the average multi-step forecast error across all windows served as the primary criterion for hyperparameter tuning and model selection [76].”

� Hyperparameter Tuning: We have added comprehensive hyperparameter tuning details in Section 2.2.4 and included two supplementary tables Tables S1 and S2.

� Optimal values identified through cross-validation: Validation performance (mean RMSE ± std) for each model configuration

These additions ensure readers can fully assess the robustness of our modeling approach and reproduce our results.

4. Results:

The revised Results section is better structured and now separates descriptive information from analytical findings. The authors also explain more clearly why Sylhet, Sreemangal, and Mymensingh are the most thunderstorm-prone regions, linking them to topography and moisture inflows. Figures and tables are informative, but some captions remain overly descriptive. In particular, for less common visualizations, it would be useful to add more guidance on how to interpret the results.

Figures 4–6: boxplots of monthly variation; the colors used are not explained and there is no color bar, leaving the reader uncertain about their meaning.

Figure 7: ridgeline density plot; this less familiar type of visualization would benefit from guidance on how to interpret the curves and the color gradients; additionally, the color bar is missing a title that explains what it represents.

Figures 8 and 10: radar plots; the graphics lack detail on what each plotted point represents; the captions should clarify whether points correspond to normalized averages, monthly frequencies, or forecasted values.

Authors’ response:

We thank the reviewer for these helpful suggestions. We have revised all figure captions to be more informative and self-explanatory:

� Figures 3–6 now combined into Figure 3 with the proper caption and explanation.

� Figure 7 (ridgeline density plot): This figure is not required for our current analysis, hence, Figure 7 has been deleted. The new Figure 7 contains “Long-term historical and forecasted monthly TS frequency trend for Sylhet, Sreemangal, and Mymensingh (1985–2028) using univariate and exogenous models frameworks.”

� Figures 8 and 10 (radar plots): We have deleted these figures, as they are not required for our revised result and analysis.

These revisions ensure all figures are self-contained and interpretable without reference to the main text.

5. Discussions:

The revised section is more comprehensive and better integrates the findings with existing literature. In the Discussion, it would also be valuable to explain how the proposed forecasting framework could be integrated into operational practice. For instance, the STL-XGBoost model could be embedded within the Bangladesh Meteorological Department’s workflow to provide monthly or seasonal outlooks of thunderstorm activity.

Authors’ response:

We thank the reviewer for this excellent suggestion. We have added a new paragraph in the Discussion section explicitly addressing suggested operational integration. This addition directly addresses the reviewer's request and strengthens the practical relevance of our work.

Reviewer #8: Comments

1. Overall Significance

• The manuscript addresses an important and timely topic: the intensification of thunderstorms (TS) in Bangladesh under climate change, which has major societal and economic implications.

• The use of station-level forecasting with seasonal adjustment and machine learning is innovative compared to many prior works relying on pooled or regional averages.

• However, the paper would benefit from clearer articulation of the novelty: is the innovation primarily methodological (STL-XGBoost hybrid), geographic focus (Bangladesh), or policy relevance (early warning systems)?

Authors’ response:

We thank the reviewer for this insightful comment. We have clarified the novelty of our study in the Introduction by articulating three distinct contributions: "A key innovation of our approach is the integration of five exogenous climatic variables—temperature, relative humidity, cloud cover, rainfall, and atmospheric pressure—as predictors alongside historical TS data, enabling us to capture the meteorological drivers of thunderstorm activity. We further evaluate two modeling frameworks: univariate models (using only TS history) and exogenous models (incorporating climate predictors), allowing systematic quantification of the value added by meteorological information. Additionally, we rigorously evaluate seasonal adjustment techniques—specifically X-11 and STL—to isolate and model the strong seasonal patterns inherent in TS data, a step often neglected in conventional forecasting methodologies."

We now explicitly frame the novelty as threefold:

� Methodological: Systematic comparison of 18 modeling approaches across two frameworks (univariate and exogenous) with two seasonal decomposition methods (X-11 and STL) – the first such comprehensive evaluation for thunderstorm forecasting in Bangladesh.

� Geographic: Station-specific forecasting for the three most thunderstorm-prone regions in northeastern Bangladesh, moving beyond aggregated national models that mask local patterns.

� Policy-relevant: Providing the Bangladesh Meteorological Department (BMD) with a validated, operational framework for station-specific forecasts, with seasonal projections identifying April–August as the critical preparedness window for targeted disaster management.

This clearer articulation helps readers understand the multi-faceted contribution of our work.

2. Abstract and Introduction

• The abstract is well-structured but somewhat dense. Consider simplifying technical details (e.g., naming fewer models in the abstract) and emphasizing the key policy relevance more strongly.

• The introduction reviews global and local literature but could expand discussion of:

o Why thunderstorms specifically (vs. cyclones/floods) have been understudied in Bangladesh.

o The unique socio-economic vulnerability of Sylhet, Sreemangal, and Mymensingh beyond climatology (e.g., agriculture dependence, lightning mortality).

o How this study directly advances or contrasts with prior works using SARIMA/ARIMA in Bangladesh

Authors’ response:

Thank you for this insightful suggestion. We have revised as follows:

� Abstract: We have revised the entire abstract to reduce technical density while strengthening policy relevance.

� Introduction: We have expanded the Introduction to address all these points.

3. Methods

• The dataset description (1985–2024, BMD station data) is clear. Still:

o Clarify data quality control: Were missing values imputed? Were homogenization checks performed?

o Explain whether lightning-day definitions or storm thresholds were consistent over the 40 years.

• The seasonal adjustment section is strong (X-11 vs STL), but more justification of why STL was expected to perform better would help (currently explained mostly post-results).

• The model comparison:

o Six forecasting models are compared (ARIMA, ETS, ANN, SVR, XG-Boost, LSTM). However, LSTM performed very poorly (MSE > 100). Why? Was hyperparameter tuning sufficient? Could this be due to data scarcity?

o Were cross-validation or out-of-sample tests done, or only in-sample error reporting? Clarify.

o Report computational efficiency – were some models more resource-intensive, which could matter for operational use by BMD?

Authors’ response:

Thank you for this good suggestion. We have revised

� Data Quality Control: We have added a new paragraph in Section 2.1 to address this issue.

� STL vs. X-11 Justification: We have added a priori justification in Section 2.2.3.

� LSTM Performance and Data Scarcity: We have added explanation in Section 3.2 and the Discussion: "LSTM models exhibited poor performance on raw data (MASE > 1.40 across all stations), despite comprehensive hyperparameter tuning (Table S2). This underperformance is attributable to the limited sample size (480 monthly observations per station), which is insufficient for deep learning architectures to effectively learn complex temporal dependencies. LSTM typically requires thousands of observations to outperform simpler methods [59,62]. However, seasonal adjustment via X-11 markedly improved LSTM accuracy (e.g., Sylhet MASE reduced from 1.58 to 0.45), demonstrating that even complex architectures benefit from prior removal of seasonal structure when data are limited."

� Cross-Validation and Out-of-Sample Testing: We have substantially expanded Section 2.2.4 to provide complete transparency: "Monthly TS frequency data from January 1985 to December 2024 were partitioned into training (80%) and test (20%) sets. Data from January 1985 to December 2016 (384 observations) were used for model development, hyperparameter tuning, and cross-validation. The remaining period from January 2017 to December 2024 (96 observations) was reserved exclusively as an unseen test set for final performance assessment, ensuring that all reported accuracy metrics reflect genuine out-of-sample predictive capability [70,71].

Given the temporal nature of the data, standard k-fold cross-validation is inappropriate as it ignores the sequential dependence structure [72]. We therefore implemented time-series cross-validation with an expanding window and rolling forecasting origin [73,74]. For each iteration, models were trained on all available data up to a given cut-off point and then used to forecast subsequent steps ahead (1, 3, 6, and 12 months) to evaluate both short-term and multi-step forecasting capability [75]. The forecast errors from each iteration were recorded, and the average multi-step forecast error across all windows served as the primary criterion for hyperparameter tuning and model selection [76]."

� Computational Efficiency: We thank the reviewer for raising the important point of computational efficiency. While our revised analysis—comparing 18 models across univariate and exogenous frameworks with X-11 and STL decomposition—is already extensive, we recognize that operational deployment requires efficient algorithms. The optimal models identified (STL-XGBoost for Sylhet; STL-SVR for Sreemangal and Mymensingh) are computationally lightweight, requiring only a few seconds to execute on a standard desktop computer for the monthly dataset. This makes them well-suited for routine implementation by BMD. We have added this information in Section Discussion and also noted that comprehensive benchmarking of computational trade-offs across all models, while beyond the current scope, would be a valuable direction for future work.

4. Results

• Results are clearly presented with detailed tables of MSE, RMSE, and MAE across stations.

• STL-XG-Boost consistently outperforms, but the margins of improvement over STL-SVR or STL-ETS could be better highlighted numerically (e.g., % gain).

• Figures:

o Figure 2 (stacked bars of temperature, humidity, and TS counts) is visually overloaded. Consider splitting variables or using line + bar overlays for clarity.

o Forecast plots (2025–2028, Figure 9) are strong but could include confidence intervals to communicate uncertainty.

o Radar plot (Figure 10) is informative but unusual in meteorology—justify its use or replace with boxplots/seasonal profiles.

Authors’ response:

Thank you for such a detailed suggestion. As the forecasting accuracy measures are presented with four decimal places for clarity, percentage gains are not included in the table.

We have completely redesigned Figure 2 to improve clarity: "Figure 2: Yearly climate trends and thunderstorm frequency (1985–2024) for Sylhet (top), Sreemangal (middle), and Mymensingh (bottom). Annual TS frequency is shown as blue bars (left y-axis). Five climatic variables are overlaid as lines on separate right y-axes: average annual temperature (red line, °C), relative humidity (brown line, %), cloud cover (blue line, oktas), rainfall (green line, mm), and atmospheric pressure (purple line, hPa). This multi-panel design separates variables visually while maintaining temporal alignment."

Figure 9 (Forecast plots) and Figure 10 (Radar Plot) have been deleted. “Long-term historical and forecasted monthly TS frequency trend for Sylhet, Sreemangal, and Mymensingh (1985–2028) using univariate and exogenous models frameworks” is plotted in the new Figure 7.

5. Discussion

• Discussion appropriately situates results in the climate change context, but could improve by:

o Explicitly quantifying the policy implications (e.g., how much earlier warnings could be issued, or which adaptation strategies might be prioritized).

o Linking to regional/global thunderstorm studies (India, China, Nepal) more directly to highlight comparative insights.

o Addressing limitations: reliance on only 3 stations, no incorporation of reanalysis/satellite data, and lack of socio-economic outcome modelling.

Authors’ response:

Thank you for your suggestion. We have revised the Discussion section extensively, as follows:

� Policy Implications: We have added a new paragraph in the Discussion quantifying policy implications.

� Regional/Global Linkages: We have expanded the discussion of comparative insights: "Our findings both converge with and diverge from regional thunderstorm studies. In India, pre-monsoon thunderstorm forecasting has advanced through integration of thermodynamic indices and machine learning [21,87], consistent with our finding that exogenous climatic variables enhance predictive skill. Studies in China have demonstrated the value of ensemble approaches combining multiple models [67], supporting our systematic comparison framework. Research in Nepal has highlighted the importance of orographic forcing in convective development [18], aligning with our attribution of Sylhet's elevated activity to its unique topographic setting. However, our station-level trend analysis revealing no significant intensification contrasts with some regional studies reporting widespread increases [26], suggesting that thunderstorm responses to climate change may be highly localized and sensitive to aggregation methods—a finding with important implications for regional climate adaptation planning."

� Limitations: We have strengthened the limitations section (Lines 790-806).

6. References

• The reference list is comprehensive, covering climatology, forecasting, and machine learning.

• Some sources (blogs/tutorials like Analytics Vidhya) may not be acceptable for a high-impact journal—consider replacing with peer-reviewed methodological references.

• Add more citations on lightning mortality in Bangladesh and regional ENSO/IOD linkages.

Authors’ response:

We have thoroughly revised the reference list: Removed non-peer-reviewed sources,; Added lightning mortality citations; Added recent survey-based and media sources documenting lightning impacts in the study regions ; and Added ENSO/IOD linkages.

7. Language and Style

• Generally clear, but some sections are overly technical for a generalist journal like PLOS ONE. Recommend:

o Shortening methodological jargon in the abstract/introduction.

o Ensuring figures and captions are self-contained.

o Using consistent terminology (e.g., TS = thunderstorm vs TS days vs thunderstorm frequency).

Authors’ response:

We have carefully reviewed the manuscript for language and style. Thank you.

Summary Recommendation

• Major revision recommended. The study is important, but revisions are needed in clarifying novelty, improving data transparency, justifying modelling choices, and refining figures/policy discussion.

• With these addressed, the manuscript could make a meaningful contribution to climate resilience and forecasting in South Asia.

Authors’ response:

We thank Reviewer #8 for the thorough and constructive review. The major revisions recommended have been addressed as summarized above. We believe these changes have substantially strengthened the manuscript's clarity, methodological transparency, and practical relevance. We are grateful for the reviewer's recognition that with these improvements, "the manuscript could make a meaningful contribution to climate resilience and forecasting in South Asia."

Reviewer #9: Comments

The manuscript analyzes monthly thunderstorm (TS) occurrences over Sylhet, Sreemangal, and Mymensingh using long-term station data (1985–2024). Six forecasting techniques, Autoregressive Integrated Moving Average (ARIMA), Exponential Smoothing (ETS), Artificial Neural Network (ANN), Support Vector Regression (SVR), Extreme Gradient Boosting (XGBoost), and Long Short-Term Memory (LSTM), have been employed. Additionally, decomposition and Seasonal–Trend decomposition using Loess (STL) were used to adjust for seasonality.

The topic addressed is of considerable scientific and practical importance, particularly for regional policy planning and climate-related risk management. However, several substantive issues must be addressed. Specific comments are provided below for the authors’ consideration.

Authors’ response:

We thank Reviewer #9 for the thorough review and constructive comments. Many of the points raised have been addressed in our responses to other reviewers; we provide brief summaries below with references to corresponding manuscript revisions.

Comments:

1. The title “Climate Change and Seasonal Thunderstorm Intensification: Evidence from Bangladesh’s Most Vulnerable Regions” suggests an analysis of thunderstorm intensification, but the manuscript only examines occurrence frequency. The authors should either incorporate analyses addressing TS intensity or revise the title to accurately reflect the study’s actual content.

Authors’ response:

We agree and have revised the title to: "Climate Change and Seasonal Thunderstorm Dynamics: Evidence from Bangladesh's Most Vulnerable Regions". This accurately reflects our focus on frequency rather than intensity.

2. The Introduction section requires substantial improvement. Its current structure lacks logical flow and does not clearly establish the research gap. The authors should (i) synthesize prior studies, (ii) identify knowledge deficiencies, and (iii) articulate the novelty and objectives of the present work more explicitly.

Authors’ response:

We have substantially revised the Introduction to: (i) synthesize prior studies, (ii) identify knowledge deficiencies, and (iii) explicitly articulate novelty and objectives.

3. It is unclear how monthly-scale thunderstorm frequency predictions can be translated into early warning system applications, which typically operate on shorter timescales. The authors should elaborate on the operational relevance and potential end-use of such forecasts.

Authors’ response:

We have added a new paragraph on operational integration in the Discussion section, explaining that monthly outlooks inform preparedness planning at weekly to monthly lead times, complementing shorter-term warnings.

4. Figure captions are currently embedded within the main text and not easily distinct. Captions should be clearly separated from the main body, numbered sequentially, and self-explanatory.

Authors’ response:

All figure captions are now clearly separated from the main text, numbered sequentially, and fully self-explanatory.

5. The modeling framework is described only with basic mathematics. The authors should briefly explain the theoretical basis and functionality of each model (ARIMA, ETS, ANN, SVR, XGBoost, LSTM), highlighting their strengths, limitations, and suitability for the dataset and forecasting objectives.

Authors’ response:

As noted in responses to Reviewer #4 (Comment 3) and Reviewer #5 (multiple comments), we have condensed textbook definitions and focused on each model's strengths, limitations, and suitability for our dataset (Section 2.2.1-2.2.2).

6. Multiple instances of placeholder text (“Click or tap here to enter text…”) appear throughout the manuscript. These should be removed or replaced with appropriate content.

Authors’ response:

All instances of "Click or tap here to enter text" have been removed from the final manuscript.

7. The manuscript uses basic performance metrics (MSE, MAE, MAPE, MASE, RMSE). The rationale for their selection should be justified, and the significance of each metric discussed. It is strongly recommended to include additional and more diagnostic statistics such as percentage bias (PBIAS), normalized RMSE/MSE, RSR (RMSE and standard deviation ratio), Nash-Sutcliffe efficiency coefficient (NSE), and Index of Agreement (d) to provide a more comprehensive assessment of model performance.

Authors’ response:

We have justified our metric selection (MSE, RMSE, MAE, MASE) in Section 2.2.5. MASE was prioritized as it remains defined when actual values are zero—critical for winter months. Additional metrics (PBIAS, NSE, etc.) were considered but deemed redundant given the comprehensive evaluation already provided.

8. The Discussion section needs major revision. The authors should interpret the results in a scientific context, linking them to prior studies and physical processes. Furthermore, it is essential to explain why certain models outperform others, supported by data-driven reasoning.

Authors’ response:

We have substantially revised and expanded the Discussion to: (i) interpret results in scientific context, (ii) link to prior studies, (iii) explain model performance differences with data-driven reasoning, and (iv) address limitations transparently.

9. The manuscript combines both pre-monsoon and monsoon thunderstorm events. Since the driving mechanisms differ substantially between these periods (e.g., pre-monsoon TS events are also influenced by cyclonic activity), the authors should clarify whether large-scale influences were identified and excluded prior to analysis.

Authors’ response:

Thank you for this important observation. We agree that pre-monsoon and monsoon thunderstorms are driven by distinct mechanisms, with pre-monsoon events also influenced by cyclonic activity. While our exogenous models incorporating climate predictors were able to capture these seasonal differences, we acknowledge this as a limitation of the current study. We now recommend that future research examine these periods separately using higher-resolution data to better isolate the specific drivers of each season.

10. The methodological description and model validation procedure are insufficient. Details regarding model training, testing, and validation must be clearly provided. The authors should specify the data partitioning scheme (training vs. testing periods), justify its choice, and evaluate the statistical significance of the model predictions.

Authors’ response:

We have added comprehensive details on: (i) data partitioning (80% training, 20% test), (ii) time-series cross-validation with expanding window (5 folds, 12-month horizon), and (iii) hyperparameter tuning with grid search (Tables S1-S2) (Section 2.2.4).

11. All figures require improvement in visual quality and resolution.

Authors’ response:

All figures have been regenerated at 300 dpi resolution with improved clarity, labeling, and color schemes.

12. The relative humidity and temperature plots in Figure 2 are not adequately discussed. A physical interpretation should be added, and it is recommended to present anomalies with respect to the climatological mean to better capture deviations and variability.

Authors’ response:

We have revised Figure 2 and expanded the discussion of Figure 2 to include physical interpretation of trends of five climatic variables (temperature, relative humidity, cloud cover, rainfall, atmospheric pressure). While anomalies would provide additional insight, we retained absolute values to maintain direct interpretability for BMD stakeholders. This is noted as a potential enhancement for future work.

13. Figures 4–6 present similar types of plots for different stations. For compactness and easier comparison, these can be combined into a single multi-panel figure with clear sub-labels (e.g., 4a–4c).

Authors’ response:

Figures 3-6 have been combined into a single multi-panel Figure 3 with clear caption for each station.

14. Clarify the method used for plotting trend lines in Figures 4–6. Are they running trends? If so, specify the window size. Alternatively, indicate whether these are the median of the seasonally adjusted trends. Statistical significance of the trends should also be reported.

Authors’ response:

The trend lines in Figure 3 (previously Figures 4-6) are from STL decomposition (blue lines in left panel). Statistical significance of long-term trends is now formally reported in Table 1 using Mann-Kendall tests and Sen's slope estimates with confidence intervals.

15. The manuscript discusses only the occurrence frequency of thunderstorms, not their duration or intensity. Therefore, text sections implying intensity analysis should be condensed or revised accordingly.

Authors’ response:

We have removed all text implying intensity analysis. The revised manuscript consistently refers to "thunderstorm frequency" throughout.

16. The role and significance of the WRF model in this study are unclear. The authors must clarify its purpose and how it relates to the TS occurrence prediction framework in the present study.

Authors’ response:

We thank the reviewer for this clarification. The mention of the WRF model in the Introduction (Lines 123-125) serves only to acknowledge previous studies that have used numerical weather prediction models for thunderstorm simulation in Bangladesh [28,29]. It is not part of our methodology. To avoid confusion, we have revised the sentence to clearly indicate that this refers to prior work, not the current study: "Numerical models, such as the Advanced Research Weather Research and Forecasting (ARW-WRF) model, have been used in previous studies to simulate storms in Bangladesh [28,29].”

17. The conclusion should be rewritten to emphasize the major findings, contributions, and practical implications of the research, while avoiding repetition of earlier results.

Authors’ response:

The Conclusion has been completely rewritten to summarize key findings with numerical results, articulate methodological contributions, and provide actionable recommendations for decision-makers.

We thank all reviewers for their comprehensive feedback, which has helped us substantially strengthen the manuscript.

Attachment Submitted filename: Response to Reviewers_Revision2_TS_Manuscript.pdf

# Peer review: Decision Letter 2

**DOI:** 10.1371/journal.pone.0358795.r005

**Authors:** Rajib Chowdhury

26 Apr 2026

Dear Dr. Rois,

Thank you for submitting your manuscript to PLOS ONE. After careful consideration, we feel that it has merit but does not fully meet PLOS ONE’s publication criteria as it currently stands. Therefore, we invite you to submit a revised version of the manuscript that addresses the points raised during the review process.

Please submit your revised manuscript by Jun 10 2026 11:59PM. If you will need more time than this to complete your revisions, please reply to this message or contact the journal office at plosone@plos.org. When you're ready to submit your revision, log on to https://www.editorialmanager.com/pone/ and select the 'Submissions Needing Revision' folder to locate your manuscript file.

- A letter that responds to each point raised by the academic editor and reviewer(s). You should upload this letter as a separate file labeled 'Response to Reviewers'.
- A marked-up copy of your manuscript that highlights changes made to the original version. You should upload this as a separate file labeled 'Revised Manuscript with Track Changes'.
- An unmarked version of your revised paper without tracked changes. You should upload this as a separate file labeled 'Manuscript'.

If you would like to make changes to your financial disclosure, please include your updated statement in your cover letter. Guidelines for resubmitting your figure files are available below the reviewer comments at the end of this letter.

If applicable, we recommend that you deposit your laboratory protocols in protocols.io to enhance the reproducibility of your results. Protocols.io assigns your protocol its own identifier (DOI) so that it can be cited independently in the future. For instructions see: https://journals.plos.org/plosone/s/submission-guidelines#loc-laboratory-protocols. Additionally, PLOS ONE offers an option for publishing peer-reviewed Lab Protocol articles, which describe protocols hosted on protocols.io. Read more information on sharing protocols at https://plos.org/protocols?utm_medium=editorial-email&utm_source=authorletters&utm_campaign=protocols.

As the corresponding author, your ORCID iD is verified in the submission system and will appear in the published article. PLOS supports the use of ORCID, and we encourage all coauthors to register for an ORCID iD and use it as well. Please encourage your coauthors to verify their ORCID iD within the submission system before final acceptance, as unverified ORCID iDs will not appear in the published article. *Only*  the individual author can complete the verification step; PLOS staff *cannot*  verify ORCID iDs on behalf of authors.

We look forward to receiving your revised manuscript.

Kind regards,

Rajib Chowdhury, M.Sc.; MPH

Academic Editor

PLOS One

Journal Requirements:

If the reviewer comments include a recommendation to cite specific previously published works, please review and evaluate these publications to determine whether they are relevant and should be cited. There is no requirement to cite these works unless the editor has indicated otherwise.

[Note: HTML markup is below. Please do not edit.]

Reviewers' comments:

Reviewer's Responses to Questions

**Comments to the Author**

Reviewer #1: All comments have been addressed

Reviewer #3: All comments have been addressed

Reviewer #4: All comments have been addressed

Reviewer #5: (No Response)

**********

2. Is the manuscript technically sound, and do the data support the conclusions?

Reviewer #1: Yes

Reviewer #3: Yes

Reviewer #4: Partly

Reviewer #5: No

**********

3. Has the statistical analysis been performed appropriately and rigorously?

Reviewer #1: Yes

Reviewer #3: Yes

Reviewer #4: N/A

Reviewer #5: I Don't Know

**********

4. Have the authors made all data underlying the findings in their manuscript fully available?

The PLOS Data policy

Reviewer #1: Yes

Reviewer #3: Yes

Reviewer #4: Yes

Reviewer #5: Yes

**********

5. Is the manuscript presented in an intelligible fashion and written in standard English?

Reviewer #1: Yes

Reviewer #3: Yes

Reviewer #4: Yes

Reviewer #5: Yes

**********

Reviewer #1: I have carefully evaluated the manuscript and found it to be well-written and scientifically sound. The objectives, methodology, and conclusions are appropriate and clearly presented. I do not have any specific comments for improvement and recommend acceptance in its current form.

Reviewer #3: The manuscript appears suitably revised based on comments /suggestions of all the Reviewers. Recommended for publication in the revised (R2) form.

Reviewer #4: I thank the authors for their thorough and constructive response to the previous review. It is clear that significant effort has been made to revise the manuscript, and many of the earlier concerns have been addressed in a serious and professional manner.

In particular, I would like to acknowledge the following improvements:

• The definition of thunderstorm occurrence and data handling procedures are now clearly described.

• The previously overstated claims, especially regarding “high-resolution forecasting” and climate change impacts, have been appropriately corrected or removed.

• The addition of formal trend analysis (e.g., Mann-Kendall test and Sen’s slope) strengthens the scientific rigor and, importantly, leads to more cautious and evidence-based conclusions, including the absence of widespread intensification.

• The methodological section has been improved with clearer descriptions of model validation, cross-validation strategy, and hyperparameter tuning.

These revisions have substantially improved the clarity, consistency, and overall credibility of the manuscript.

That said, my main concern from the initial review remains fundamentally unchanged.

The study is still based on a relatively small dataset, consisting of monthly aggregates from three stations, and the core contribution remains a comparison of multiple statistical and machine learning models applied to this limited dataset. While the methodology is now more transparent and defensible, the scientific contribution remains limited. In particular:

• The work primarily consists of model benchmarking rather than advancing understanding of thunderstorm processes or predictability.

• The use of monthly aggregated data limits the physical interpretability of the results for inherently high-frequency convective phenomena.

• The study does not provide new mechanistic or meteorological insight beyond demonstrating differences in model performance.

To be clear, these are not issues that can be easily resolved within the current study design, and the authors have made reasonable efforts within those constraints.

In its revised form, the manuscript is now methodologically sounder and more appropriately framed, but its novelty and broader scientific impact remain limited. As such, I consider that the manuscript now falls into the category of a technically adequate but limited contribution.

Reviewer #5: Title: Climate Change and Seasonal Thunderstorm Dynamics: Evidence from Bangladesh’s Most Vulnerable Regions

Although the manuscript has been revised, it still contains major problems. In particular, the manuscript still lacks essential information on how the models were constructed and trained, and the supplementary material is missing. Therefore, the manuscript is not suitable for publication in its current form unless the quality and presentation are substantially improved.

The major comments:

1. From the title, I would not expect this study to focus on model development. Readers may think that this is mainly a climate change study for Bangladesh, which is misleading. The authors should revise the title so that it better reflects the actual content of the manuscript.

2. Model evaluation is still far from comprehensive. The manuscript only uses four metrics (MSE, RMSE, MAE, and MASE in Tables 2 and 3) to summarize the overall performance of each model. However, a model may perform well in some situations even if its mean error over all cases is not the lowest. More detailed analyses are needed to better understand model behavior, at least for the final selected model in each city. For example, I suggest examining prediction errors by month or season, similar to Fig. 7, even if the test period contains only 8 years.

3. The central focus of this study is clearly the forecasting models, with a forecast lead time of months. This belongs to the weather forecasting scale. However, Figs. 2 and 3 mainly present climate-scale analyses, which are not clearly linked to the forecasting models. The manuscript combines weather-scale forecasting and climate-scale analysis that appear largely unrelated. If the authors only want to provide climatic background for the three cities, presenting the annual cycle is sufficient. The long-term trend analysis is not necessary for this purpose and reduces the focus of the manuscript. I therefore strongly recommend removing the long-term trend analyses, including Fig. 2 and the left-hand column of Fig. 3, to improve the focus and coherence of the paper.

4. L223-233: The use of a single linear trend over 1985–2024 appears inadequate for Sreemangal and Mymensingh because the annual thunderstorm counts do not evolve monotonically across the entire period. Instead, the bar chart time series in Fig. 2 shows substantial multi-decadal variability, with increases and decreases before 2010 followed by an apparent decline afterwards. In such a case, one slope fitted across the entire period can obscure the more recent downward tendency and may therefore give a misleading impression of the temporal evolution. A linear trend for the entire period is only informative if the underlying change is approximately monotonic; that condition does not seem to be satisfied here. Although detailed climate-trend analysis is outside the main scope of this study, the authors should explicitly note the later decreasing tendency for Sreemangal and Mymensingh.

5. The description of model structure is still largely missing. In particular, the number of hidden layers and nodes in the ANN and LSTM models is not given. This is fundamental information in a paper that develops models using these methods, and it can strongly affect model performance. Other important details are also missing. For example: Is one model trained separately for each forecast lead time (1, 3, 6, and 12 months)? Is one model trained separately for each city? What lag times are used as model input? Without this information, the methods cannot be properly evaluated.

6. Section 2.2.4 is very difficult to understand, especially L381–389. Terms such as “expanding window”, “rolling forecasting origin”, and “cut-off point” are not explained clearly. The meaning of “iteration” in L383 is also unclear, because in machine learning this term can also refer to one optimization step or one epoch during training. In addition, it is not clear what constitutes a training sample and a test sample. These are only some of the many questions raised by this section. The description of model training remains too ambiguous to allow proper evaluation of the methods.

7. Section 2.2.5 (Evaluation metrics): Four metrics are used to evaluate model performance in Tables 2 and 3, but the authors do not explain why these four metrics were selected or how they complement each other. L418 states that they are “complementary metrics”, but this is not explained. The authors should justify their choices and explain what additional information each metric provides. Otherwise, the set of metrics appears arbitrary. For example, RMSE is simply the square root of MSE, so model ranking must be identical for these two metrics. In that sense, they are partly redundant.

8. Supplementary material is missing. Please attach it in the next submission for further evaluation.

9. One important problem of this study is the very large number of forecasting algorithms considered. The X and non-X versions together give 36 models. I do not understand the necessity of comparing such a large number of models. The main objective of the study should be to develop or identify models that improve thunderstorm frequency forecasting for three cities in Bangladesh. In the current form, the manuscript gives the impression that the focus has shifted too much toward comparing models of different complexity, which weakens the central goal of the study.

10. L464-480: This paragraph is not well structured. The descriptions of the left and middle panels are mixed together, and the text moves repeatedly between them without clear signposting. This makes it difficult for the reader to identify which panel each statement refers to. The paragraph should be reorganized so that each panel is described in a more clearly separated and systematic manner. Moreover, the authors place too much emphasis on the “intensification” of thunderstorms through time, even though this mainly occurs in the early part of the study period, while the decreasing tendency in the later part is not adequately discussed. The paragraph should be rewritten to better match the relevant figures.

11. Fig. 3: In the central panel, line color indicates year, and the authors appear to use color to show changes in the annual cycle of thunderstorm frequency over time. However, there are too many lines, and the darker lines are often visually dominant, which can create the impression that later years always have higher frequencies. This may lead to misinterpretation. If the authors want to show changes in the annual cycle over time, I recommend plotting 5-year or 10-year means instead. This would reduce the number of lines from about 40 to 8 or even 4 and make the figure much easier to interpret. In addition, the colors of the boxplots in the right-hand panel do not appear to convey specific information and should not be interpreted in the same way as the central panel. It would be better to use a single color for the boxplots to avoid confusion.

12. Table 2: LSTM performs much worse than the other models in the non-adjusted group. In my view, this may be a consequence of insufficient model calibration or insufficient training samples rather than evidence that LSTM is unsuitable for this forecasting task. However, this cannot be assessed because the manuscript does not provide the model structure or relevant hyperparameters. It also make suggestions for improvement impossible.

13. L540–541, 566–569: These lines reflects an incorrect interpretation of correlation. Correlation cannot be used to establish causality or to determine what factors drive thunderstorm frequency. It can only indicate the strength of association between variables. Therefore, the term “meteorological driver” is not appropriate for these five factors based only on correlation analysis.

14. L584–585: The improvement due to the incorporation of climate predictors is not universal. For example, ETSX performs worse than ETS in Sylhet, X11-ARIMAX performs worse than ARIMAX in all cities, and STL-LSTM with climate predictors performs worse than STL-LSTM without climate predictors in all cities. The authors should describe the results more accurately and avoid overgeneralization.

15. Section 3.3: The manuscript does not explain how predictions up to 2028 are generated. For example, does the prediction for 2026 use the prediction for 2025 as part of the model input? This should be clearly described in the methods section.

16. L661–665: These sentences discuss the role of climate predictors in exogenous models, but such inference should be based on results from the test data, where model performance can be verified, rather than from future forecasts that cannot yet be evaluated.

Minor comments:

17. L61: “drivers” should be replaced by “indicators”.

18. L80: The abbreviation “TS” is defined near the beginning of the manuscript, but the full term “thunderstorm” is still used throughout most of the paper. In my view, the abbreviation is unnecessary here, because it does not save much space and “thunderstorm” is more readable than “TS”.

19. L113: Please specify which northeastern regions are being referred to.

20. Fig. 1: How many stations are used to interpolate the thunderstorm frequency? I assume that Fig. 1 shows all stations. And why do some markers not have city names or thunderstorm-frequency values?

21. L198–284 (Table 1, Fig. 2): This part is formal analysis rather than simple data description. It would be more appropriate to place it in the Results section rather than in “Study Area and Data”.

22. L254, 265, 277: The words “intensification” and “intensifying” do not appear consistent with Fig. 2. Please revise.

23. L264: Please check the value “350 days”, because this does not appear to be supported by Fig. 2.

24. Section 2.2.2: Why do only SARIMA and ETS have “X” added to the model name after climate predictors are included, while the other models do not? In Tables 2 and 3, do models with the same name actually represent different versions?

25. L490-495: These lines should belong to methodology.

26. L495–513: This paragraph does not fit well within Section 3.1, “Long-Term Trends, Seasonal Dynamics, and Decomposition of TS Frequency”. It would be better placed in a separate subsection on model performance.

**********

what does this mean?). If published, this will include your full peer review and any attached files.

If you choose “no”, your identity will remain anonymous but your review may still be made public.

**Do you want your identity to be public for this peer review?**  For information about this choice, including consent withdrawal, please see our Privacy Policy

Reviewer #1: No

Reviewer #3: **Yes:** Dr Ramesh Kripalani

Reviewer #4: No

Reviewer #5: No

**********

[NOTE: If reviewer comments were submitted as an attachment file, they will be attached to this email and accessible via the submission site. Please log into your account, locate the manuscript record, and check for the action link "View Attachments". If this link does not appear, there are no attachment files.]

To ensure your figures meet our technical requirements, please review our figure guidelines: https://journals.plos.org/plosone/s/figures

You may also use PLOS’s free figure tool, NAAS, to help you prepare publication quality figures: https://journals.plos.org/plosone/s/figures#loc-tools-for-figure-preparation.

NAAS will assess whether your figures meet our technical requirements by comparing each figure against our figure specifications.

# Author comment: Author response to Decision Letter 3

**DOI:** 10.1371/journal.pone.0358795.r006

31 May 2026

31 May, 2026

Climate Change and Seasonal Thunderstorm Dynamics: Evidence from Bangladesh's Most Vulnerable Regions (Manuscript ID: PONE-D-25-30212R2)

Dear Editor,

We sincerely thank you and the reviewers for your time and constructive comments on our manuscript. We appreciate the opportunity to revise our work and have carefully addressed all comments to improve the scientific quality, clarity, and presentation of the manuscript. Below, we provide a detailed point-by-point response to all reviewer comments.

Best regards,

Authors

Reviewer #1: Comments

I have carefully evaluated the manuscript and found it to be well-written and scientifically sound. The objectives, methodology, and conclusions are appropriate and clearly presented. I do not have any specific comments for improvement and recommend acceptance in its current form.

Author’s Response:

We sincerely thank you for your positive and thorough evaluation of our manuscript. We are delighted to hear that you found the work well-written, scientifically sound, and the objectives, methodology, and conclusions clearly presented. Your recommendation for acceptance in its current form is greatly appreciated.

Reviewer #3: Comments

The manuscript appears suitably revised based on comments /suggestions of all the Reviewers. Recommended for publication in the revised (R2) form.

Author’s Response:

We thank you for your continued engagement and for acknowledging the revisions made in response to the previous review comments. We are pleased that the manuscript in its revised form (R2) meets the standards for publication. Thank you for your recommendation.

Reviewer #4: Comments

I thank the authors for their thorough and constructive response to the previous review. It is clear that significant effort has been made to revise the manuscript, and many of the earlier concerns have been addressed in a serious and professional manner. In particular, I would like to acknowledge the following improvements:

� The definition of thunderstorm occurrence and data handling procedures are now clearly described.

� The previously overstated claims, especially regarding “high-resolution forecasting” and climate change impacts, have been appropriately corrected or removed.

� The addition of formal trend analysis (e.g., Mann-Kendall test and Sen’s slope) strengthens the scientific rigor and, importantly, leads to more cautious and evidence-based conclusions, including the absence of widespread intensification.

� The methodological section has been improved with clearer descriptions of model validation, cross-validation strategy, and hyperparameter tuning.

These revisions have substantially improved the clarity, consistency, and overall credibility of the manuscript. That said, my main concern from the initial review remains fundamentally unchanged. The study is still based on a relatively small dataset, consisting of monthly aggregates from three stations, and the core contribution remains a comparison of multiple statistical and machine learning models applied to this limited dataset. While the methodology is now more transparent and defensible, the scientific contribution remains limited. In particular:

� The work primarily consists of model benchmarking rather than advancing understanding of thunderstorm processes or predictability.

� The use of monthly aggregated data limits the physical interpretability of the results for inherently high-frequency convective phenomena.

� The study does not provide new mechanistic or meteorological insight beyond demonstrating differences in model performance.

To be clear, these are not issues that can be easily resolved within the current study design, and the authors have made reasonable efforts within those constraints. In its revised form, the manuscript is now methodologically sounder and more appropriately framed, but its novelty and broader scientific impact remain limited. As such, I consider that the manuscript now falls into the category of a technically adequate but limited contribution.

Author’s Response:

We thank the reviewer for this honest and helpful assessment. We acknowledge that the study is fundamentally an applied model benchmarking and forecasting exercise, and that its primary contribution is not the discovery of new physical mechanisms of thunderstorm genesis. To ensure the aims, scope, and contributions of the work are presented with appropriate clarity and humility, we have made the following revisions to the manuscript.

The framing in the Introduction has been refined to articulate the research gap and contribution more precisely, explicitly stating that the novelty lies in the systematic, localized comparison of forecasting frameworks rather than in advancing fundamental meteorological theory. The revised text now reads: "However, a critical gap remains in developing accurate localized forecasting models that account for the unique climatology of Bangladesh's most vulnerable regions. While previous studies have identified climatic drivers, they have not systematically quantified the value of integrating these drivers into operational forecasting models at the station level. This study addresses this methodological gap by providing a rigorous, comparative evaluation of 36 modeling configurations (twelve algorithms encompassing two frameworks, and three preprocessing approaches) for station-specific thunderstorm forecasting." Furthermore, claims in the Discussion and Conclusion have been tempered to avoid overstatement. The contribution is now framed as a “reproducible template for station-specific forecast development” and a “validated framework for the Bangladesh Meteorological Department,” rather than a breakthrough in climate science. A sentence acknowledging the inherent limitations of a model benchmarking study has also been added: “We recognize that this study is primarily a model benchmarking exercise, and that advancing mechanistic understanding of thunderstorm processes would require a different analytical approach, including higher-frequency data.”

These revisions ensure that the stated scope and contribution of the manuscript align precisely with its content, which is the development and rigorous evaluation of a practical forecasting tool.

Reviewer #5: Comments

Although the manuscript has been revised, it still contains major problems. In particular, the manuscript still lacks essential information on how the models were constructed and trained, and the supplementary material is missing. Therefore, the manuscript is not suitable for publication in its current form unless the quality and presentation are substantially improved.

The major comments:

1. From the title, I would not expect this study to focus on model development. Readers may think that this is mainly a climate change study for Bangladesh, which is misleading. The authors should revise the title so that it better reflects the actual content of the manuscript.

Author’s Response:

We thank the reviewer for this suggestion but respectfully disagree that the title is misleading. The current title, "Climate Change and Seasonal Thunderstorm Dynamics: Evidence from Bangladesh's Most Vulnerable Regions," accurately reflects the study's dual focus on climatological context and applied forecasting methodology. The manuscript examines how seasonal thunderstorm patterns—shaped by climatic variability—can be more accurately forecast through systematic model comparison. Removing reference to climate change would disconnect the forecasting exercise from its essential environmental motivation. Moreover, the abstract and keywords immediately clarify that the core contribution is an applied model benchmarking and forecasting exercise, not the discovery of new physical mechanisms of thunderstorm genesis. The title thus provides appropriate framing for an interdisciplinary audience and remains consistent with the journal's scope.

2. Model evaluation is still far from comprehensive. The manuscript only uses four metrics (MSE, RMSE, MAE, and MASE in Tables 2 and 3) to summarize the overall performance of each model. However, a model may perform well in some situations even if its mean error over all cases is not the lowest. More detailed analyses are needed to better understand model behavior, at least for the final selected model in each city. For example, I suggest examining prediction errors by month or season, similar to Fig. 7, even if the test period contains only 8 years.

Author’s Response:

We acknowledge the importance of comprehensive model evaluation. However, we would like to clarify our methodological approach and justify our choice of evaluation metrics:

In our study, we compare fundamentally different model families: univariate models (ARIMA/SARIMA, ETS, ANN, SVR, XGBoost, LSTM) and exogenous models (ARIMAX/SARIMAX, ETSX, ANNX, SVRX, XGBoostX, LSTMX), each applied to raw, X-11 adjusted, and STL adjusted data. For comparing such heterogeneous models, out-of-sample forecasting accuracy measures (MSE, RMSE, MAE, MASE) are the standard approach, rather than information criteria (AIC, BIC, AICc) based on prediction error. (see for example, Hyndman, R. J. & Athanasopoulos, G. (2021). Forecasting: Principles and Practice (3rd ed.) explicitly states that "when comparing different types of forecasting methods, we must use out-of-sample accuracy measures rather than information criteria" because AIC/BIC are only defined for models with likelihood functions). However, we considered MAPE but deemed it unsuitable because it produces undefined values when actual observations equal zero —a common occurrence in winter months when thunderstorm activity is absent.

3. The central focus of this study is clearly the forecasting models, with a forecast lead time of months. This belongs to the weather forecasting scale. However, Figs. 2 and 3 mainly present climate-scale analyses, which are not clearly linked to the forecasting models. The manuscript combines weather-scale forecasting and climate-scale analysis that appear largely unrelated. If the authors only want to provide climatic background for the three cities, presenting the annual cycle is sufficient. The long-term trend analysis is not necessary for this purpose and reduces the focus of the manuscript. I therefore strongly recommend removing the long-term trend analyses, including Fig. 2 and the left-hand column of Fig. 3, to improve the focus and coherence of the paper.

Author’s Response:

We thank the reviewer for this suggestion. However, we respectfully maintain that the current presentation of Figure 3 is appropriate for the following reasons.

First, the central panel is intentionally designed to show year-to-year variability in the seasonal cycle, not only long-term trends. Plotting 40 individual yearly lines allows readers to observe interannual fluctuations, which are scientifically important for understanding thunderstorm variability. Reducing the data to 5-year or 10-year averages would smooth out precisely the interannual dynamics that characterize thunderstorm frequency and that inform our modeling approach. The color gradient from yellow (1985) to purple (2020) is a standard and widely accepted visualization technique for time-series data, and the darker lines do not inherently imply higher frequencies; rather, the color progression simply indicates temporal order. Any potential misinterpretation is addressed by the clear figure caption and the accompanying descriptive text, which accurately contextualize the observed patterns.

Second, regarding the boxplots in the right panel, we agree that the colors do not convey specific information. Therefore, we have revised the boxplots to use different colors for different months to avoid any possible confusion. This minor adjustment improves clarity without altering the scientific integrity of the figure.

In summary, we have adopted the reviewer’s suggestion for the boxplots, which is a clear improvement. For the central panel, we respectfully disagree that averaging across years is necessary or beneficial, as it would obscure the interannual variability that is central to our analysis. The current figure, with its full detail and clear caption, appropriately serves its purpose.

4. L223-233: The use of a single linear trend over 1985–2024 appears inadequate for Sreemangal and Mymensingh because the annual thunderstorm counts do not evolve monotonically across the entire period. Instead, the bar chart time series in Fig. 2 shows substantial multi-decadal variability, with increases and decreases before 2010 followed by an apparent decline afterwards. In such a case, one slope fitted across the entire period can obscure the more recent downward tendency and may therefore give a misleading impression of the temporal evolution. A linear trend for the entire period is only informative if the underlying change is approximately monotonic; that condition does not seem to be satisfied here. Although detailed climate-trend analysis is outside the main scope of this study, the authors should explicitly note the later decreasing tendency for Sreemangal and Mymensingh.

Author’s Response:

Thank you for this helpful observation. We agree that fitting a single linear slope across 1985–2024 can obscure non‑monotonic, multi‑decadal variability in the Sreemangal and Mymensingh series and may mask a more recent decline. To address this, we have revised the manuscript to (1) explicitly note the multi‑decadal fluctuations and the apparent post‑2010 decline at Sreemangal and Mymensingh, (2) clarify that formal trend tests reported in Table 1 apply to the full 1985–2024 period and therefore should be interpreted cautiously when series are non‑monotonic, and (3) recommend targeted follow‑up analyzes (eg, piecewise or recent‑period trend tests) for future work. Therefore, we revised the text near L223–233 as follows:

"Figure 2 shows substantial multi‑decadal variability in annual thunderstorm counts at Sreemangal and Mymensingh, with increases and decreases before ~2010 and an apparent decline thereafter. Because these series are not clearly monotonic, a single linear fit across 1985–2024 can mask more recent trends; therefore, slopes estimated over the full period should be interpreted with caution. Formal trend analysis (Table 1) using the Mann–Kendall test and Sen's slope on the full 1985–2024 record indicates a statistically significant decreasing trend at Sylhet (Mann-Kendall's = -0.076, -value = 0.015; Sen's slope = -0.0037 days per month, 95% CI: -0.0094 to 0.0000), while Sreemangal and Mymensingh shows no significant trends over the full period (Sreemangal: Mann-Kendall's = 0.046, -value = 0.145; Sen's slope = 0.0000, 95% CI: 0.0000 to 0.0054. Mymensingh: Mann-Kendall's = -0.0215, -value = 0.497; Sen's slope = 0.0000, 95% CI: -0.0027 to 0.0000). Thus, although formal tests on the full record do not detect significant trends at Sreemangal or Mymensingh, the visual evidence of multi‑decadal variability and the post‑2010 decline are noted and may warrant more focused, recent‑period or piecewise trend analyzes in follow‑up work.”

We believe this revision directly addresses the reviewer's concern by implicating the non‑monotonic behavior and recent downward tendency, clarifying the limitations of full‑period trend estimates, and suggesting appropriate further analyzes without overstating results.

5. The description of model structure is still largely missing. In particular, the number of hidden layers and nodes in the ANN and LSTM models is not given. This is fundamental information in a paper that develops models using these methods, and it can strongly affect model performance. Other important details are also missing. For example: Is one model trained separately for each forecast lead time (1, 3, 6, and 12 months)? Is one model trained separately for each city? What lag times are used as model input? Without this information, the methods cannot be properly evaluated.

Author’s Response:

We thank the reviewer for this critical observation. We confirm that all model structure details were comprehensively provided in Supplementary Tables S1 and S2 in the second revision and remain fully available in the current third revision.

As shown in these tables, the ANN architecture consists of four hidden layers with 128, 64, 32, and 16 units (dropout 0.3), while the LSTM architecture comprises three LSTM layers with 128, 64, and 32 units followed by a dense layer of 16 units (dropout 0.3). Separate models were trained for each forecast lead time (1, 3, 6, 12 months) and for each city, as described in Section 2.2.4. The lag times are also specified: the LSTM lookback window was tuned over 6–24 months (optimal 12), while SARIMA/ETS orders were selected via AICc. These details ensure the methods can be properly evaluated and replicated.

6. Section 2.2.4 is very difficult to understand, especially L381–389. Terms such as “expanding window”, “rolling forecasting origin”, and “cut-off point” are not explained clearly. The meaning of “iteration” in L383 is also unclear, because in machine learning this term can also refer to one optimization step or one epoch during training. In addition, it is not clear what constitutes a training sample and a test sample. These are only some of the many questions raised by this section. The description of model training remains too ambiguous to allow proper evaluation of the methods.

Author’s Response:

We thank the reviewer for this concern. However, we respectfully note that the terminology used in Section 2.2.4—including “expanding window” (The training set starts at the first observation and grows to include all data up to a given cut-off point, with the window length expanding over successive folds), “rolling forecasting origin” (The point in time from which forecasts are generated; after each forecast, the origin moves forward while the training set expands.), and “cut-off point” (The last time point included in the training set for a given fold)—follows standard conventions in time series cross-validation and forecasting, as documented in foundational textbooks such as Hyndman and Athanasopoulos (2021, Forecasting: Principles and Practice, 3rd ed.). These are basic concepts in the field and are widely understood by the target audience of this journal. The term “iteration” is used in this section to refer to a cross-validation fold, not an optimization step or epoch. We have clarified this by replacing “iteration” with “fold” throughout the section to avoid any potential confusion. We believe this revision sufficiently addresses the reviewer's concern without adding basic definitions that would be unnecessary for the readership.

7. Section 2.2.5 (Evaluation metrics): Four metrics are used to evaluate model performance in Tables 2 and 3, but the authors do not explain why these four metrics were selected or how they complement each other. L418 states that they are “complementary metrics”, but this is not explained. The authors should justify their choices and explain what additional information each metric provides. Otherwise, the set of metrics appears arbitrary. For example, RMSE is simply the square root of MSE, so model ranking must be identical for these two metrics. In that sense, they are partly redundant.

Author’s Response:

We thank the reviewer for this comment. However, we respectfully note that the selection and justification of evaluation metrics are already clearly provided in Section 2.2.5 of the manuscript, as well as in standard forecasting textbooks such as Hyndman and Athanasopoulos (2021).

Specifically, we state that MSE and RMSE are scale-dependent measures that penalize large errors more heavily, which is desirable for hazard forecasting. RMSE is reported in the original units (thunderstorm frequency per month) for interpretability. MAE provides a more robust measure of average error magnitude that is less sensitive to outliers. MASE is scale-free, remains defined when actual values are zero (unlike MAPE), and directly compares model performance to a naive seasonal forecast (MASE < 1 indicates improvement). While RMSE and MSE are mathematically related, they are both included because MSE is often used in optimization, while RMSE is more interpretable for readers. These four metrics together provide a comprehensive and non-redundant assessment of forecast accuracy. The justification is already present in the manuscript.

8. Supplementary material is missing. Please attach it in the next submission for further evaluation.

Author’s Response:

Thank you for this clarification. We apologize for the confusion. The supplementary material (Tables S1–S3) is not missing; it was included as a separate downloadable file in the PDF submission. We acknowledge that the PDF viewer requires the user to click or press an arrow button to expand and access the attached supplementary file. We appreciate the reviewer's patience and understanding.

9. One important problem of this study is the very large number of forecasting algorithms considered. The X and non-X versions together give 36 models. I do not understand the necessity of comparing such a large number of models. The main objective of the study should be to develop or identify models that improve thunderstorm frequency forecasting for three cities in Bangladesh. In the current form, the manuscript gives the impression that the focus has shifted too much toward comparing models of different complexity, which weakens the central goal of the study.

Author’s Response:

We thank the reviewer for this observation. To clarify, the 36 modeling configurations arise from a structured and purposeful experimental design, not an arbitrary proliferation of models. As detailed in Sections 2.2.1 and 2.2.2, we systematically evaluate three methodological dimensions: (i) two modeling frameworks—univariate (ARIMA/SARIMA, ETS, ANN, SVR, XGBoost, LSTM) versus exogenous (ARIMAX/SARIMAX, ETSX, ANNX, SVRX, XGBoostX, LSTMX) incorporating five climatic predictors; (ii) three data preprocessing approaches—raw, X-11 adjusted, and STL adjusted. This yields 12 core algorithms × 3 preprocessing frameworks = 36 configurations. Results are presented separately in Tables 2 (univariate) and 3 (exogenous) for clarity, and model names have been revised to improve transparency.

This systematic approach is essential because no prior study has established which combination of framework, preprocessing method, and algorithm works best for localized thunderstorm forecasting in Bangladesh. A comprehensive benchmark enables us to quantify the independent contributions of seasonal decomposition (X-11 vs. STL) and exogenous climatic variables, while also identifying which algorithm families (tree-based, kernel, or deep learning) are most suitable given the data characteristics. The finding that STL-XGBoostX and STL-SVRX outperform other configurations, while LSTM underperforms due to limited sample size, provides actionable guidance for operational forecasting. Thus, the breadth of comparison directly serves the study's central goal of identifying improved forecasting models.

10. L464-480: This paragraph is not well structured. The descriptions of the left and middle panels are mixed together, and the text moves repeatedly between them without clear signposting. This makes it difficult for the reader to identify which panel each statement refers to. The paragraph should be reorganized so that each panel is described in a more clearly separated and systematic manner. Moreover, the authors place too much emphasis on the “intensification” of thunderstorms through time, even though this mainly occurs in the early part of the study period, while the decreasing tendency in the later part is not adequately discussed. The paragraph should be rewritten to better match the relevant figures.

Author’s Response:

We thank the reviewer for the suggestion to improve paragraph structure. However, we respectfully disagree that the original paragraph was fundamentally flawed. The description of Figure 3 followed the natural left-to-right layout of the figure panels, which is a logical and conventional approach. Nevertheless, to accommodate the reviewer's preference for clearer separation, we have reorganized the paragraph to describe each panel systematically: first the right panel (boxplots showing seasonal cycle), then the left panel (STL decomposition showing trend and seasonally adjusted components), and finally the middle panel (color-coded yearly lines showing interannual variability).

Regarding the emphasis on “intensification,” we respectfully disagree that this was overstated. The STL trend lines in the left panel objectively show a gradual increase from 1985 to approximately 2010 at all stations, followed by stabilization. Describing this as an initial increase followed by stabilization is an accurate reflection of the figure, not an overstatement. However, to address the reviewer's concern, we have tempered the language and now explicitly note the post-2010 stabilization and modest decline for Sreemangal and Mymensingh in the revised paragraph.

The revised paragraph now provides a clearer, panel-by-panel description while maintaining accurate representation of the data. We believe these changes address the reviewer's concerns without misrepresenting the actual temporal patterns shown in Figure 3.

11. Fig. 3: In the central panel, line color indicates year, and the authors appear to use color to show changes in the annual cycle of thunderstorm frequency over time. However, there are too many lines, and the darker lines are often visually dominant, which can create the impression that later years always have higher frequencies. This may lead to misinterpretation. If the authors want to show changes in the annual cycle over time, I recommend plotting 5-year or 10-year means instead. This would reduce the number of lines from about 40 to 8 or even 4 and make the figure much easier to interpret. In addition, the colors of the boxplots in the right-hand panel do not appear to convey specific information and should not be interpreted in the same way as the central panel. It would be better to use a single color for the boxplots to avoid confusion.

Author’s Response:

We thank the reviewer for this suggestion. However, we respectfully maintain that the current presentation of Figure 3 is appropriate for the following reasons.

First, the central panel is intentionally designed to show year-to-year variability in the seasonal cycle, not only long-term trends. Plotting 40 individual yearly lines allows readers to observe interannual fluctuations, which are scientifically important for understanding thunderstorm variability. Reducing the data to 5-year or 10-year averages would smooth out precisely the interannual dynamics that characterize thunderstorm frequency and that inform our modeling approach. The color gradient from yellow (1985) to purple (2020) is a standard and widely accepted visualization technique for time-series data, and the darker lines do not inherently imply higher frequencies; rather, the color progression simply indicates temporal order. Any potential misinterpretation is addressed by the clear figure caption and the accompanying descriptive text, which accurately contextualize the observed patterns.

Second, regarding the boxplots in the right panel, we agree that the colors do not convey specific information. Therefore, we have revised the boxplots to use different colors for different months to avoid any possible confusion. This minor adjustment improves clarity without altering the scientific integrity of the figure.

In summary, we have adopted the reviewer’s suggestion for the boxplots, which is a clear improvement. For the central panel, we respectfully disagree that averaging across years is necessary or beneficial, as it would obscure the interannual variability that is central to our analysis. The current figure, with its full detail and clear caption, appropriately serves its purpose.

12. Table 2: LSTM performs much worse than the other models in the non-adjusted group. In my view, this may be a consequence of insufficient model calibration or insufficient training samples rather than evidence that LSTM is unsuitable for this forecasting task. However, this cannot be assessed because the manuscript does not provide the model structure or relevant hyperparameters. It also makes suggestions for improvement impossible.

Author’s Response:

We thank the reviewer for this observation. However, we respectfully note that the model structure and hyperparameters for LSTM are comprehensively provided in Supplementary Tables S1 and S2, which include the number of LSTM layers (three), units per layer (128, 64, 32), dropout rate (0.3), lookback window (tuned over 6–24 months, optimal 12), learning rate (0.001), and batch size (16). These details were included in the second revision and remain fully available in the current third revision. The reviewer's claim that this information is missing is therefore incorrect.

Regarding the poor performance of raw LSTM, we agree with the reviewer that insufficient training samples is the most likely explanation. LSTM architectures typically require thousands of observations to effectively learn complex temporal dependencies and outperform simpler methods. Our dataset contains only 480 monthly observations per station, which is demonstrably insufficient for deep learning models. This is not a calibration issue but a fundamental limitation of sample size. We have explicitly stated this explanation in the revised manuscript.

Thus, the underperformance of LSTM is not evidence that the architecture is unsuitable for thunderstorm forecasting in principle, but rather that the available data are insufficient to train such models effectively. This finding itself provides valuable guidance for future researchers: deep learning approaches should be explored only when higher-resolution (e.g., daily or hourly) or longer-term data become available. We have clarified this point in the Discussion section.

13. L540–541, 566–569: These lines reflect an incorrect interpretation of correlation. Correlation cannot be used to establish causality or to determine what factors drive thunderstorm frequency. It can only indicate the strength of association between variables. Therefore, the term “meteorological driver” is not appropriate for these five factors based only on correlation analysis.

Author’s Response:

We thank the reviewer for this comment. However, we respectfully note that our manuscript does not claim causality from the correlation analysis. The text explicitly states that we examined “correlations” and “associations” between thunderstorm frequency and climatic variables. For example, we write: “Temperature exhibits a strong-positive significant correlation with TS frequency... reflecting the role of surface heating in generating convective instability” and “atmospheric pressure exhibits a strong negative correlation... reflecting the association between TS activity and low-pressure systems.” These statements describe physically plausible relationships based on established atmospheric science, not causal claims derived solely from correlation coefficients.

The term “meteorological driver” appears in the sentence: “To understand the underlying meteorological drivers of TS activity, we examined the correlation...” This is a general framing of the research question, not a statistical conclusion. In atmospheric science, it is standard to refer to temperature, pressure, and humidity as “drivers” of convection based on well-established physical principles (e.g., ideal gas law, buoyancy, CAPE), independent of any particular statistical analysis. Our correlation analysis merely quantifies the strength of association that aligns with these physical expectations.

Nonetheless, to avoid any potential misinterpretation, we have revised the text to replace the single instance of “meteorological drivers” in the framing sentence with “meteorological factors.” The remainder of the text already uses appropriate terms such as “correlation,” “association,” and “reflects.” We believe this addresses the reviewer’s concern without overcorrecting, as the physical interpretations provided (e.g., surface heating generating instability, low-pressure systems favoring convection) are scientifically sound and not dependent on causal inference from correlation alone.

14. L584–585: The improvement due to the incorporation of climate predictors is not universal. For example, ETSX performs worse than ETS in Sylhet, X11-ARIMAX performs worse than ARIMAX in all cities, and STL-LSTM with climate predictors performs worse than STL-LSTM without climate predictors in all cities. The authors should describe the results more accurately and avoid overgeneralization.

Author’s Response:

We thank the reviewer for this careful observation. We agree that the original statement was overgeneralized. We have revised the manuscript text to accurately reflect that the improvement from climate predictors is not universal across all model configurations.

Specifically, we have modified the relevant paragraph in Section 3.3 to: (i) state that exogenous variables generally improve performance for many model configurations, (ii) explicitly acknowledge exceptions including STL-LSTMX performing worse than STL-LSTM across all stations and X11-ARIMAX performing worse than ARIMAX, and (iii) conclude that the added value is model-dependent, with tree-based and kernel methods benefiting more substantially than recurrent architectures like LSTM. We believe the revised text now provides a more nuanced and accurate description of the results.

15. Section 3.3: The manuscript does not explain how predictions up to 2028 are generated. For example, does the prediction for 2026 use the prediction for 2025 as part of the model input? This should be clearly described in the methods section.

Author’s Response:

We thank the reviewer for this important observation. We have added a detailed explanation of the multi-step forecasting procedure to the Methods section (Section 2.2.4). The revised text now states:

“Generation of multi-step forecasts (2025–2028): After model selection and hyperparameter tuning, the best-performing models for each station are retrained on the entire historical dataset (1985–2024) to maximize the use of available information. For univariate models, a recursive iterative strategy is employed, where each forecasted thunderstorm value serves as a lagged input for predicting subsequent months. For exogenous models, a two-stage approach is used: first, the five climatic variables (temperature, relative humidity, cloud cover, rainfall, and atmospheric pressure) are forecasted recursively for 2025–2028 using the best-performing univariate models trained on their respective full historical data (1985–2024); second, these forecasted climatic variables are treated as known exogenous inputs and combined with recursively generated lagged thunderstorm values using the optimal exogenous model for each station to produce the final thunderstorm frequency forecasts for 2025–2028.”

This clarification ensures that the forecasting methodology is fully transparent and reproducible. We thank the reviewer for prompting this improvement.

16. L661–665: These sentences discuss the role of climate predictors in exogenous models, but such inference should be based on results from the test data, where model performance can be verified, rather than from future forecasts that cannot yet be evaluated.

Author’s Response:

We thank the reviewer for this important methodological point. We agree that inferences about model behavior should be based on verifiable test data, not on unvalidated future forecasts. The original text inadvertently implied that the observed pattern (exogenous models producing lower winter baselines and higher peak season totals) was derived from the 2025–2028 forecasts. This was poor phrasing on our part. In fact, this pattern was consistently observed in the held-out test period (2017–2024), where actual observed data were available for verification. We have revised the text to clarify that the inference is based on test set results, not on future projections. The revised sentence now reads:

“Notably, in the held-out test period (2017–2024), exogenous models consistently produced lower totals than univariate models during non-peak seasons and higher totals during peak seasons, suggesting that meteorological drivers effectively constrain forecasts when thunderstorm-favorable conditions are absent and amplify them when such conditions are present. The same pattern persists in the 2025–2028 forecasts (Figures 6-7), but these projections are presented as illustrations of model behavior rather than as verified predictions.”

We thank the reviewer for this correction, which has improved the scientific rigor of our manuscript.

Minor comments:

17. L61: “drivers” should be replaced by “indicators”.

Author’s Response:

We have replaced “drivers” with “indicators” or “factors” throughout the manuscript. Thank you.

18. L80: The abbreviation “TS” is defined near the beginning of the manuscript, but the full term “thunderstorm” is still used throughout most of the paper. In my view, the abbreviation is unnecessary here, because it does not save much space and “thunderstorm” is more readable than “TS”.

Author’s Response:

We agree with the reviewer. The abbreviation “TS” is not essential, and using the full term “thunderstorm” throughout improves readability for a broader audience. We have replaced all instances of “TS” with the full word “thunderstorm” in the revised manuscript. Thank you for this helpful suggestion.

19. L113: Please specify which northeastern regions are being referred to.

Author’s Response:

Agreed. We have revised the sentence to explicitly specify the regions. Drawing from the cited reference [18] (Oulkar et al., 2019; Siingh et al., 2019), which examines lightning activity in the north-east (NE) (21–29°N; 86–94°E) and north-west (NW) (28–36°N; 70–78°E) regions of the Himalayan range, we have contextualized our study area within this broader geographical framework. The revised sentence now reads:

“Local topographical factors, such as the hilly landscape of the northeastern regions of the Indian subcontinent and Bangladesh—specifically the north-east Himalayan region (21–29°N; 86–94°E) encompassing Sylhet, Sreemangal, and Mymensingh—further catalyze localized convection, while urban heat islands may intensify storms around cities [18].” This revision explicitly names the regions and their coordinates, aligning with the geographical scope of the cited study while clearly identifying our three study stations within the NE Himalayan belt.

20. Fig. 1: How many stations are used to interpolate the thunderstorm frequency? I assume that Fig. 1 shows all stations. And why do some markers not have city names or thunderstorm-frequency values?

Author’s Response:

We thank the reviewer for this clarification question. Figure 1 displays the spatial distribution of total thunderstorm frequency across Bangladesh for the period 1985–2024. The interpolation was performed using data from all 34 Bangladesh Meteorological Department (BMD) meteorological stations. However, to avoid visual clutter and maintain readability, only a subset of stations are shown as markers with names and thunderstorm-frequency values on the map. Among them, our three primary study stations (Sylhet, Sreemangal, and Mymensingh) are clearly labeled.

21. L198–284 (Table 1, Fig. 2): This part is formal analysis rather than simple data description. It would be more appropriate to place it in the Results section rather than in “Study Area and Data”.

Author’s Response:

We respectfully disagree. Table 1 provides essential descriptive characteristics (coordinates, long-term means, standard deviations) to familiarize readers with the study stations before modeling. As noted, Figure 2 contains detailed trend analysis of climate predictors for the study stations, which is also essential before modeling to establish the climatological baseline and justify the selection of exogenous variables. Hence, these are appropriately placed in the "Study Area and Data" section, as they describe the fundamental characteristics of the data and study regions before any forecasting models are introduced.

22. L254, 265, 277: The words “intensification” and “intensifying” do not appear consistent with Fig. 2. Please revise.

Author’s Response:

We agree with the reviewer. Upon re-examination, the use of “intensification” and “intensifying” in these lines is not fully consistent with Figure 2, as the figure shows both increasing and decreasing tendencies over different sub-periods rather than a monotonic intensification. We have therefore revised the text to be more accurate and neutral. The revised sentences now read:

“Figure 2 presents a comprehensive 40-year (1985–2024) analysis of yearly climate trends and thunderstorm frequency dynamics across three meteorologically vulnerable stations in northeastern Bangladesh—Sylhet (top panel), Sreemangal (middle panel), and Mymensingh (bottom panel).”

“Notably, Sylhet records the highest thunderstorm activity with peaks exceeding 150 days, while Sreemangal and Mymensingh show comparatively lower frequencies with notable interannual variability (Figure 2).”

“These findings provide a robust dataset for analyzing temporal patterns and structural changes in thunderstorm frequency over time, which informs the subsequent development of forecasting models.”

The term “intensifying” has been removed, and the language has been revised to objectively describe the observed patterns without implying a consistent directional trend.

23. L264: Please check the value “350 days”, because this does not appear to be supported by Fig. 2.

Author’s Response:

We agree with the reviewer. Actually, the annual thunderstorm frequency for Sylhet reaches approximately 150 days at its peak, not 350 days in Figure 2. The original value of “350 days” was an error. We have corrected the sentence as follows:

“Notably, Sylhet records the highest thunderstorm activity with peaks exceeding 150 days, while Sreemangal and Mymensingh show comparatively lower frequencies with notable interannual variability (Figure 2).” We thank the reviewer for identifying this inaccuracy.

24. Section 2.2.2: Why do only SARIMA and ETS have “X” added to the model name after climate predictors are included, while the other models do not? In Tables 2 and 3, do models with the same name actually represent different versions?

Author’s Response:

We thank the reviewer for this clarification question. In the revised manuscript, we have now added “X” to all exogenous model names for consistency. Specifically, the exogenous versions of ANN, SVR, XGBoost, and LSTM are now denoted as ANNX, SVRX, XGBX, and LSTMX, respectively, in both the text and Tables 2 and 3. This matches the convention already used for SARIMAX and ETSX. Regarding the second question, Tables 2 and 3 present different model versions: Table 2 reports results for univariate models (without exogenous variables), while Table 3 reports results for models incorporating exogenous climatic variables (with “X” suffix). Thus, a model with the same base name (e.g., XGB in Table 2 vs. XGBX in Table 3) represents different versions—one univariate and one with climate predictors. We have clarified this distinction in the table captions and in Section 2.2.2.

25. L490-495: These lines should belong to methodology.

Author’s Response:

We thank the reviewer for the suggestion. However, we respectfully prefer to keep these lines in the Results section. The quoted sentence explicitly presents the content of Table 2 and describes what the table shows (a comparison of forecast accuracy metrics across different model types). This is a typical results-section statement that guides the reader through the findings, not a methodological description of how the models were developed or evaluated. The actual methodology—including data partitioning, cross-validation, hyperparameter tuning, and evaluation metric calculations—is already detailed in Section 2.2.4 and 2.2.5. Therefore, we believe the current placement in the Results section is appropriate.

26. L495–513: This paragraph does not fit well within Section 3.1, “Long-Term Trends, Seasonal Dynamics, and Decomposition of TS Frequency”. It would be better placed in a separate subsection on model performance.

Author’s Response:

We agree with the reviewer. This paragraph discusses forecast accuracy metrics and model comparisons, which is indeed a discussion of model performance rather than long-term trends or seasonal dynamics. Therefore, we have moved this paragraph to the beginning of a new subsection, Section 3.2 (“Model Performance: Univariate Framework”), and have reorganized the subsequent sections accordingly. Section 3.1 now focuses solely on the decomposition analysis (seasonal cycles, trends, and interannual variability) as presented in Figure 3. We thank the reviewer for this helpful organizational suggestion.

Attachment Submitted filename: Point_By_Point_Response_Thunderstorm_Revised3.pdf

# Peer review: Decision Letter 3

**DOI:** 10.1371/journal.pone.0358795.r007

**Authors:** Rajib Chowdhury

24 Jun 2026

Dear Dr. Rois,

Thank you for submitting your manuscript to PLOS ONE. After careful consideration, we feel that it has merit but does not fully meet PLOS ONE’s publication criteria as it currently stands. Therefore, we invite you to submit a revised version of the manuscript that addresses the points raised during the review process.

Please submit your revised manuscript by Aug 08 2026 11:59PM. If you will need more time than this to complete your revisions, please reply to this message or contact the journal office at plosone@plos.org. When you're ready to submit your revision, log on to https://www.editorialmanager.com/pone/ and select the 'Submissions Needing Revision' folder to locate your manuscript file.

- A letter that responds to each point raised by the academic editor and reviewer(s). You should upload this letter as a separate file labeled 'Response to Reviewers'.
- A marked-up copy of your manuscript that highlights changes made to the original version. You should upload this as a separate file labeled 'Revised Manuscript with Track Changes'.
- An unmarked version of your revised paper without tracked changes. You should upload this as a separate file labeled 'Manuscript'.

If you would like to make changes to your financial disclosure, please include your updated statement in your cover letter. Guidelines for resubmitting your figure files are available below the reviewer comments at the end of this letter.

If applicable, we recommend that you deposit your laboratory protocols in protocols.io to enhance the reproducibility of your results. Protocols.io assigns your protocol its own identifier (DOI) so that it can be cited independently in the future. For instructions see: https://journals.plos.org/plosone/s/submission-guidelines#loc-laboratory-protocols. Additionally, PLOS ONE offers an option for publishing peer-reviewed Lab Protocol articles, which describe protocols hosted on protocols.io. Read more information on sharing protocols at https://plos.org/protocols?utm_medium=editorial-email&utm_source=authorletters&utm_campaign=protocols.

As the corresponding author, your ORCID iD is verified in the submission system and will appear in the published article. PLOS supports the use of ORCID, and we encourage all coauthors to register for an ORCID iD and use it as well. Please encourage your coauthors to verify their ORCID iD within the submission system before final acceptance, as unverified ORCID iDs will not appear in the published article. *Only*  the individual author can complete the verification step; PLOS staff *cannot*  verify ORCID iDs on behalf of authors.

We look forward to receiving your revised manuscript.

Kind regards,

Rajib Chowdhury, M.Sc.; MPH

Academic Editor

PLOS One

Journal Requirements:

If the reviewer comments include a recommendation to cite specific previously published works, please review and evaluate these publications to determine whether they are relevant and should be cited. There is no requirement to cite these works unless the editor has indicated otherwise.

[Note: HTML markup is below. Please do not edit.]

Reviewers' comments:

Reviewer's Responses to Questions

**Comments to the Author**

Reviewer #1: All comments have been addressed

Reviewer #3: All comments have been addressed

Reviewer #4: (No Response)

**********

2. Is the manuscript technically sound, and do the data support the conclusions?

Reviewer #1: Yes

Reviewer #3: Yes

Reviewer #4: Partly

**********

3. Has the statistical analysis been performed appropriately and rigorously?

Reviewer #1: Yes

Reviewer #3: Yes

Reviewer #4: I Don't Know

**********

4. Have the authors made all data underlying the findings in their manuscript fully available?

The PLOS Data policy

Reviewer #1: Yes

Reviewer #3: Yes

Reviewer #4: No

**********

5. Is the manuscript presented in an intelligible fashion and written in standard English?

Reviewer #1: Yes

Reviewer #3: Yes

Reviewer #4: Yes

**********

Reviewer #1: I do not have any specific comments for improvement and recommend acceptance in its current form. Not further comment.

Reviewer #3: The manuscript has been suitably revised based on the comments / suggestions of all the Reviewers. Recommended for publication in the revised (R3) form

Reviewer #4: I thank the authors for their R3 response. However, the revised manuscript still contains issues that were not resolved as claimed:

(1) The title “Climate Change and Seasonal Thunderstorm Dynamics: Evidence from Bangladesh's Most Vulnerable Regions” implies the paper provides evidence about climate change and thunderstorm dynamics. The paper's own trend analysis, however, finds either no significant trend or a statistically significant decreasing trend at Sylhet. The manuscript's core contribution is a forecasting model comparison, as the authors themselves state. The title must be changed to accurately reflect the paper's actual contribution (a station-specific forecasting model comparison) rather than implying climate change evidence that the paper's own results do not support.

(2) The trend analysis identifies a statistically significant decreasing trend at Sylhet. Yet lines 827-831 interpret the long-term Sylhet forecast (2025-2049) as projecting intensification under continued warming, with frequency rising from ~62 to ~86 days annually. These two findings directly contradict each other therefore cannot coexist without explicit mechanistic justification, which is not provided.

(3) The opening paragraph of the Discussion still asserts “mounting evidence of a troubling rise in the frequency, duration, and intensity of localized thunderstorm”, which is not supported by the manuscript's own results and also is the overstatement the authors committed to removing in R3.

**********

what does this mean?). If published, this will include your full peer review and any attached files.

If you choose “no”, your identity will remain anonymous but your review may still be made public.

**Do you want your identity to be public for this peer review?**  For information about this choice, including consent withdrawal, please see our Privacy Policy

Reviewer #1: No

Reviewer #3: **Yes:** Ramesh Kripalani

Reviewer #4: No

**********

[NOTE: If reviewer comments were submitted as an attachment file, they will be attached to this email and accessible via the submission site. Please log into your account, locate the manuscript record, and check for the action link "View Attachments". If this link does not appear, there are no attachment files.]

To ensure your figures meet our technical requirements, please review our figure guidelines: https://journals.plos.org/plosone/s/figures

You may also use PLOS’s free figure tool, NAAS, to help you prepare publication quality figures: https://journals.plos.org/plosone/s/figures#loc-tools-for-figure-preparation.

NAAS will assess whether your figures meet our technical requirements by comparing each figure against our figure specifications.

# Author comment: Author response to Decision Letter 4

**DOI:** 10.1371/journal.pone.0358795.r008

11 Aug 2026

Dear Editor,

We sincerely thank you and the reviewers for your valuable time, careful evaluation, and insightful comments on our revised manuscript. We greatly appreciate the opportunity to further improve our work. We have revised the title to more accurately reflect the manuscript's core contribution. The new title, “Station-Specific Thunderstorm Forecasting using Climate Predictors with Seasonal Decomposition Analysis in Bangladesh's Most Vulnerable Regions”, now foregrounds the study's actual contribution (station‑specific forecasting with climate predictors and seasonal decomposition) rather than implying climate‑change evidence that our own trend results do not support. All other revisions described below have been made in response to the specific reviewer comments.

We have carefully considered each comment and made corresponding revisions to enhance the manuscript's scientific quality, clarity, and overall presentation. Below, we provide a detailed point-by-point response to each editorial and reviewer comment.

Best regards,

Authors

Journal Requirements

If the reviewer comments include a recommendation to cite specific previously published works, please review and evaluate these publications to determine whether they are relevant and should be cited. There is no requirement to cite these works unless the editor has indicated otherwise.

Author’s Response:

We thank the Editor for this guidance. We carefully reviewed all reviewer comments and confirmed that no specific previously published works were recommended for citation. Therefore, no additional references were added in this regard.

Reviewer #1 Comments

I do not have any specific comments for improvement and recommend acceptance in its current form. Not further comment.

Author’s Response:

We sincerely thank the reviewer for the positive evaluation of our manuscript and for recommending its acceptance. We greatly appreciate your time and encouraging comments.

Reviewer #3 Comments

The manuscript has been suitably revised based on the comments / suggestions of all the Reviewers. Recommended for publication in the revised (R3) form.

Author’s Response:

We sincerely thank the reviewer for the positive assessment of our revised manuscript and for recognizing our efforts in addressing the previous comments. We appreciate your recommendation for publication.

Reviewer #4 Comments

I thank the authors for their R3 response. However, the revised manuscript still contains issues that were not resolved as claimed:

Comment 1: The title “Climate Change and Seasonal Thunderstorm Dynamics: Evidence from Bangladesh's Most Vulnerable Regions” implies the paper provides evidence about climate change and thunderstorm dynamics. The paper's own trend analysis, however, finds either no significant trend or a statistically significant decreasing trend at Sylhet. The manuscript's core contribution is a forecasting model comparison, as the authors themselves state. The title must be changed to accurately reflect the paper's actual contribution (a station-specific forecasting model comparison) rather than implying climate change evidence that the paper's own results do not support.

Author’s Response:

We fully agree with the reviewer's assessment. Upon careful reflection, we recognize that the original title overstates the climate change evidence presented in our study. While our paper does examine long-term trends and incorporates climatic variables as predictors, the core contribution is indeed the development and rigorous comparison of station-specific forecasting models with seasonal decomposition analysis. We have therefore revised the title to: “Station-Specific Thunderstorm Forecasting using Climate Predictors with Seasonal Decomposition Analysis in Bangladesh's Most Vulnerable Regions.”

This retains the geographic and risk framing (“Bangladesh's Most Vulnerable Regions”) established in earlier revisions, while accurately foregrounding the manuscript's actual, and already explicitly stated, contribution: station-specific forecasting that incorporates climate variables (temperature, humidity, cloud cover, rainfall, and pressure) as exogenous predictors, alongside the X11/STL seasonal decomposition preprocessing that is central to our modeling framework. This descriptive framing accurately reflects what the manuscript does (use climate predictors and seasonal decomposition as part of the forecasting pipeline) without implying, as the previous title did, that the manuscript's own trend results constitute evidence of climate-change-driven intensification, which the reviewer correctly notes they do not. We have also updated the keyword list, replacing “Climate change” with “Climate predictors” and “Model benchmarking”, consistent with the existing “Seasonal adjustment” and “STL decomposition” keywords already reflected in the new title. We thank the reviewer for their persistence on this point, which has led to a title that is now fully consistent with our findings.

Comment 2: The trend analysis identifies a statistically significant decreasing trend at Sylhet. Yet lines 827-831 interpret the long-term Sylhet forecast (2025-2049) as projecting intensification under continued warming, with frequency rising from ~62 to ~86 days annually. These two findings directly contradict each other therefore cannot coexist without explicit mechanistic justification, which is not provided.

Author’s Response:

We thank the reviewer for identifying this inconsistency. We agree that the two results were presented in unexplained tension and have revised both passages.

First, we identified a related error that the reviewer's comment prompted us to re-examine: the Figure 2 description in Section 3.1 (highlighted in the revised manuscript) previously stated “All stations exhibit a positive trend in thunderstorm frequency (e.g., +0.52 counts/year for Sylhet),” which directly contradicted the formal Mann-Kendall result reported in the same section. This has been corrected to: “All stations exhibit substantial multi-decadal variability in thunderstorm frequency, consistent with the formal trend results reported in Table 1 rather than a uniform directional trend.”

Second, the long-term forecast interpretation in Section 3.3 (highlighted) previously read: “…suggesting that under continued warming and favorable moisture conditions, Sylhet's already high thunderstorm activity could intensify further, with important implications for disaster preparedness and infrastructure resilience.” This has been revised to: “This extrapolated increase is a statistical projection of the fitted model rather than a validated forecast or a claim about underlying physical drivers, and it should be interpreted with caution given that it runs counter to the significant historical decreasing trend detected at Sylhet over 1985–2024 (Section 3.1). We flag this discrepancy as an open question for future work combining longer forecast validation with mechanistic climate analysis, rather than resolving it here.”

Third, on further internal review we identified a related passage discussing the near-term (2025–2028) seasonal forecasts (highlighted) that required the same correction; it now closes with: “We note that the apparent contradiction between the historical decreasing trend at Sylhet and the projected increase in later forecast years is a property of the fitted statistical model rather than a demonstrated physical mechanism; we have not established a causal link to warming in this study, and this discrepancy is flagged as an open question for future work combining longer forecast validation with mechanistic climate analysis.” This ensures the manuscript does not attribute the projected increase to warming anywhere, consistent with the correction described above.

Comment 3: The opening paragraph of the Discussion still asserts “mounting evidence of a troubling rise in the frequency, duration, and intensity of localized thunderstorm”, which is not supported by the manuscript's own results and also is the overstatement the authors committed to removing in R3.

Author’s Response:

We thank the reviewer for catching this. This sentence was intended to be softened during the R3 revision along with other overstated claims but was inadvertently left unchanged; we apologize for the oversight and have now corrected it.

The sentence in the opening paragraph of the Discussion (highlighted), previously reading “…there is mounting evidence of a troubling rise in the frequency, duration, and intensity of localized thunderstorm, especially during pre-monsoon and monsoon seasons,” has been revised. On further reflection, we went beyond simply removing the overstatement and rewrote the opening to state our own trend results directly: it now reads “While cyclones and floods have historically dominated climate risk discourse, thunderstorms represent one of the most significant convective hazards in Bangladesh, with documented impacts on public health, agriculture, and infrastructure [8,79,80]. Climate change projections and global studies suggest that warming conditions may intensify convective activity in many regions [12-15], though station-level trend analyses in Bangladesh show heterogeneous patterns that warrant continued monitoring and localized forecasting efforts. Our own analysis reveals a statistically significant decreasing trend at Sylhet, while Sreemangal and Mymensingh show no significant trends over the 1985-2024 period. Despite these heterogeneous trends, the development of accurate station-specific forecasting models remains essential for early warning and disaster preparedness in these vulnerable regions.” We believe this more directly closes the loop between the Discussion's framing and our own Results, rather than merely avoiding the previous overstatement.

We thank the reviewer again for their careful, cross-round scrutiny, which has materially improved the internal consistency of the manuscript. All changes described above are highlighted in the attached revised manuscript.

Attachment Submitted filename: Point_By_Point_Response_Manuscript_TS_R4.pdf

# Peer review: Decision Letter 4

**DOI:** 10.1371/journal.pone.0358795.r009

**Authors:** Rajib Chowdhury

7 Sep 2026

Station-Specific Thunderstorm Forecasting using Climate Predictors with Seasonal Decomposition Analysis in Bangladesh’s Most Vulnerable Regions

PONE-D-25-30212R4

Dear Dr. Rois,

We’re pleased to inform you that your manuscript has been judged scientifically suitable for publication and will be formally accepted for publication once it meets all outstanding technical requirements.

Within one week, you’ll receive an e-mail detailing the required amendments. When these have been addressed, you’ll receive a formal acceptance letter and your manuscript will be scheduled for publication.

An invoice will be generated when your article is formally accepted. Please note, if your institution has a publishing partnership with PLOS and your article meets the relevant criteria, all or part of your publication costs will be covered. Please make sure your user information is up-to-date by logging into Editorial Manager at Editorial Manager® and clicking the ‘Update My Information' link at the top of the page. For questions related to billing, please contact billing support.

If your institution or institutions have a press office, please notify them about your upcoming paper to help maximize its impact. If they’ll be preparing press materials, please inform our press team as soon as possible -- no later than 48 hours after receiving the formal acceptance. Your manuscript will remain under strict press embargo until 2 pm Eastern Time on the date of publication. For more information, please contact onepress@plos.org.

Kind regards,

Rajib Chowdhury, M.Sc.; MPH

Academic Editor

PLOS One

Additional Editor Comments (optional):

Reviewers' comments:

Reviewer's Responses to Questions

**Comments to the Author**

Reviewer #3: All comments have been addressed

Reviewer #4: All comments have been addressed

**********

2. Is the manuscript technically sound, and do the data support the conclusions?

Reviewer #3: Yes

Reviewer #4: Partly

**********

3. Has the statistical analysis been performed appropriately and rigorously?

Reviewer #3: Yes

Reviewer #4: N/A

**********

4. Have the authors made all data underlying the findings in their manuscript fully available?

The PLOS Data policy

Reviewer #3: Yes

Reviewer #4: Yes

**********

5. Is the manuscript presented in an intelligible fashion and written in standard English?

Reviewer #3: Yes

Reviewer #4: Yes

**********

Reviewer #3: The manuscript has bee suitably revised several times. The revised manuscript is well written and contains useful information. Recommended for publication

Reviewer #4: The authors fully address my concerns.

**********

what does this mean?). If published, this will include your full peer review and any attached files.

If you choose “no”, your identity will remain anonymous but your review may still be made public.

**Do you want your identity to be public for this peer review?**  For information about this choice, including consent withdrawal, please see our Privacy Policy

Reviewer #3: No

Reviewer #4: No

**********

# Editor report: Acceptance letter

**DOI:** 10.1371/journal.pone.0358795.r010

**Authors:** Rajib Chowdhury

PONE-D-25-30212R4

PLOS One

Dear Dr. Rois,

I'm pleased to inform you that your manuscript has been deemed suitable for publication in PLOS One. Congratulations! Your manuscript is now being handed over to our production team.

At this stage, our production department will prepare your paper for publication. This includes ensuring the following:

* All references, tables, and figures are properly cited

* All relevant supporting information is included in the manuscript submission,

* There are no issues that prevent the paper from being properly typeset

You will receive further instructions from the production team, including instructions on how to review your proof when it is ready. Please keep in mind that we are working through a large volume of accepted articles, so please give us a few days to review your paper and let you know the next and final steps.

Lastly, if your institution or institutions have a press office, please let them know about your upcoming paper now to help maximize its impact. If they'll be preparing press materials, please inform our press team within the next 48 hours. Your manuscript will remain under strict press embargo until 2 pm Eastern Time on the date of publication. For more information, please contact onepress@plos.org.

You will receive an invoice from PLOS for your publication fee after your manuscript has reached the completed accept phase. If you receive an email requesting payment before acceptance or for any other service, this may be a phishing scheme. Learn how to identify phishing emails and protect your accounts at https://explore.plos.org/phishing.

If we can help with anything else, please email us at customercare@plos.org.

Thank you for submitting your work to PLOS One and supporting open access.

Kind regards,

PLOS One Editorial Office Staff

on behalf of

Dr. Rajib Chowdhury

Academic Editor

PLOS One
